# Discovery of Novel Tetrahydro-β-carboline Containing Aminopeptidase N Inhibitors as Cancer Chemosensitizers

**DOI:** 10.3389/fonc.2022.894842

**Published:** 2022-05-23

**Authors:** Xiaoyan Xing, Fahui Li, Yajie Hu, Lin Zhang, Qian Hui, Hongyu Qin, Qixiao Jiang, Wenyan Jiang, Chunyan Fang, Lei Zhang

**Affiliations:** ^1^ Department of Pharmacology, School of Pharmacy, Weifang Medical University, Weifang, China; ^2^ Department of Medicinal Chemistry, School of Pharmacy, Weifang Medical University, Weifang, China; ^3^ Department of Toxicology, School of Public Health, Qingdao University, Qingdao, China

**Keywords:** aminopeptidase N, inhibitor, cancer, chemosensitizer, tetrahydro-β-carboline

## Abstract

Aminopeptidase N (APN, CD13) is closely associated with the development and progression of cancer. Previous studies suggested APN as a biomarker for cancer stem cells. APN inhibitors have been intensively evaluated as chemosensitizers for cancer treatments. In the present study, tetrahydro-*β*-carboline scaffold was introduced to the structure of APN inhibitors. The synthesized compounds showed potent enzyme inhibitory activities compared with Bestatin, an approved APN inhibitor, in cell-based enzymatic assay. In combination with chemotherapeutic drugs, representative APN inhibitor molecules **D12**, **D14** and **D16** significantly improved the antiproliferative potency of anticancer drugs in the *in vitro* tests. Further mechanistic studies revealed that the anticancer effects of these drug combinations are correlated with decreased APN expression, increased ROS level, and induction of cell apoptosis. The spheroid-formation assay and colony-formation assay results showed effectiveness of Paclitaxel-APN inhibitor combination against breast cancer stem cell growth. The combined drug treatment led to reduced mRNA expression of OCT-4, SOX-2 and Nanog in the cancer stem cells tested, suggesting the reduced stemness of the cells. In the *in vivo* study, the selected APN inhibitors, especially **D12**, exhibited improved anticancer activity in combination with Paclitaxel compared with Bestatin. Collectively, potent APN inhibitors were discovered, which could be used as lead compounds for tumor chemo-sensitization and cancer stem cell-based therapies.

## Introduction

Aminopeptidase N (APN, also known as CD13) is a widely expressed type II membrane bound metalloprotease (1). It plays important roles in various cellular processes including cell migration, survival (2), viral uptake (3), angiogenesis (4), and autophagy (5). Overexpression of APN have been demonstrated in various cancers such as breast (6), ovarian (7), colorectal (8) and hepatocellular carcinoma (9). It is reported that the serum level of APN is correlated with tumor size, lymph node metastasis, and tumor metastasis (10). Therefore, serum APN expression and activity could be utilized as diagnostic and prognostic biomarkers for different types of cancers.

Inhibition of APN has been intensively evaluated for the treatment of cancers (11). A number of APN inhibitors have been designed and synthesized for targeting various cancer cellular events, including cell migration, cell growth and tumor angiogenesis (12). According to the chemical structures, different kinds of APN inhibitors have been developed, such as antibodies, peptides, and nonpeptide small molecules (13). Bestatin, a natural product extracted from *Streptomyces olivoreticuli*, is an approved APN inhibitor (14).

Overexpression of APN played important roles in the resistance to anticancer agents, anti-apoptosis of cancer cells, as well as relapse of cancers (5, 15). APN inhibitors, such as Bestatin, have been extensively studied for their abilities in enhancing radiation sensitivity and chemo-sensitivity in different types of cancers (16, 17). Moreover, APN has been identified as a functional marker for cancer stem cells (CSCs) in human cancers (18). Inhibition of APN suppressed the self-renewal and tumor-initiative abilities of CSCs (19). It is indicated that APN is a novel therapeutic target for the treatment of cancer in combination with traditional chemotherapy. Development of APN inhibitors for the enhancement of chemotherapy sensitivity could be a new strategy in cancer treatment.

**Figure 1:**
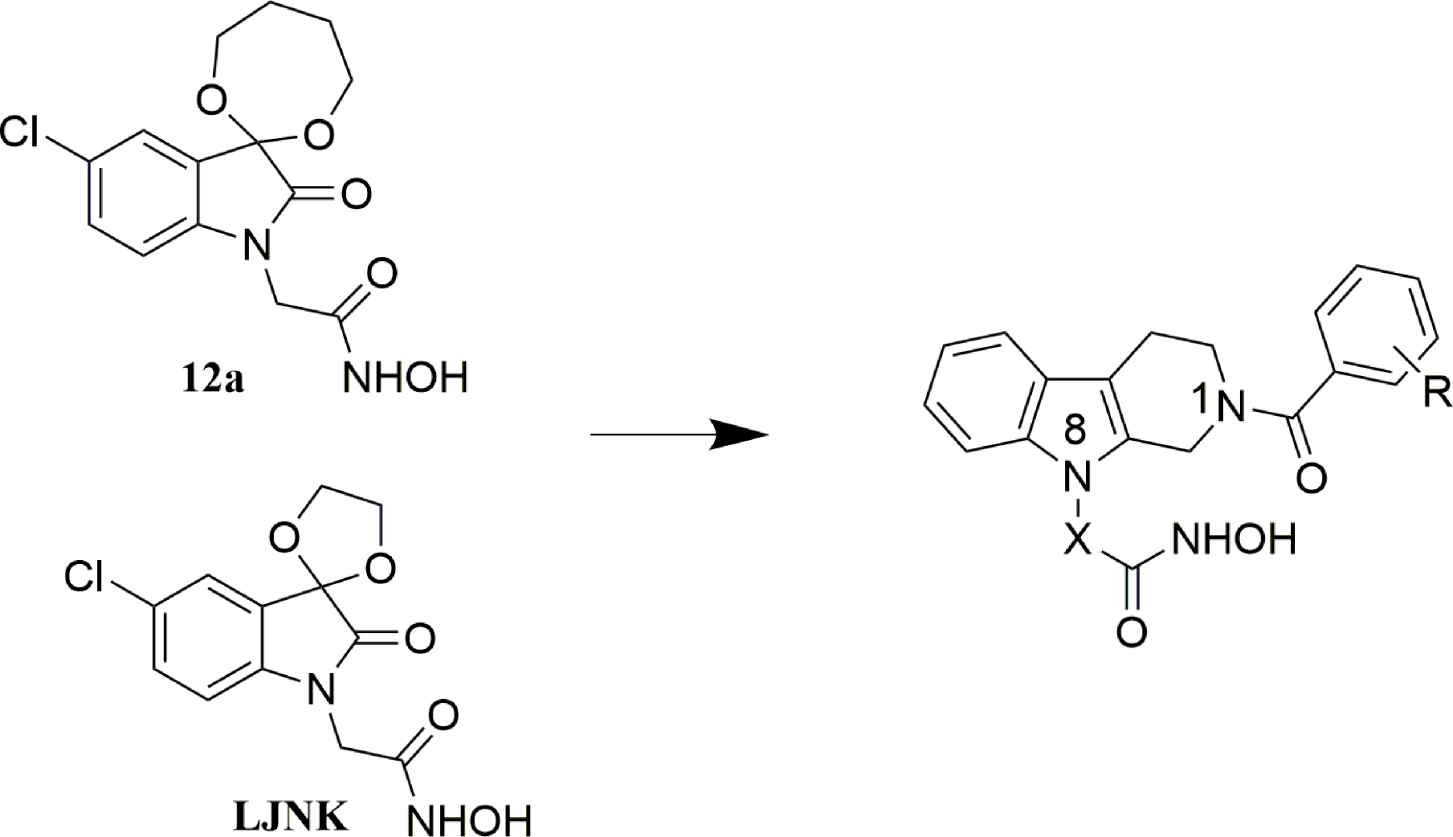
Design of tetrahydro-β-carboline containing APN inhibitors (X = -CH_2_-, -CH_2_C_6_H_4_-).

In our previous work, indoline-2,3-dione containing APN inhibitors have been developed with anticancer activities (20, 21). However, further development of these compounds was terminated due to lack of *in vivo* potency. To develop novel APN inhibitors as chemosensitizers, structural modification was performed on the existing compounds. Generally, a zinc binding group is needed for the chelation of zinc ion, which leads to the binding of inhibitors to the active site of APN. Fragments with various sizes and physicochemical properties were used to occupy different binding pockets in the active site. A linker is used to connect the former pharmacophores. To improve the binding affinity of target molecules in the active site, tetrahydro-*β*-carboline was utilized as a core fragment in the design of novel APN inhibitors (**Figure 1**). Substitutions were introduced by condensing different benzoyl chlorides to the *N1*-position in the carboline ring, and hydroxamic acid group with different linkers was introduced to *N8*-position as zinc binding group. Short linkers are usually selected in the design of APN inhibitors. In the present study, the contribution of both fatty acid and aromatic linkers was evaluated in the designed APN inhibitors. The synthesized molecules were evaluated using the enzymatic inhibition screening, *in vitro* antiproliferative test, breast CSC- based study and *in vivo* anticancer test.

### Chemistry

**Scheme 1:**
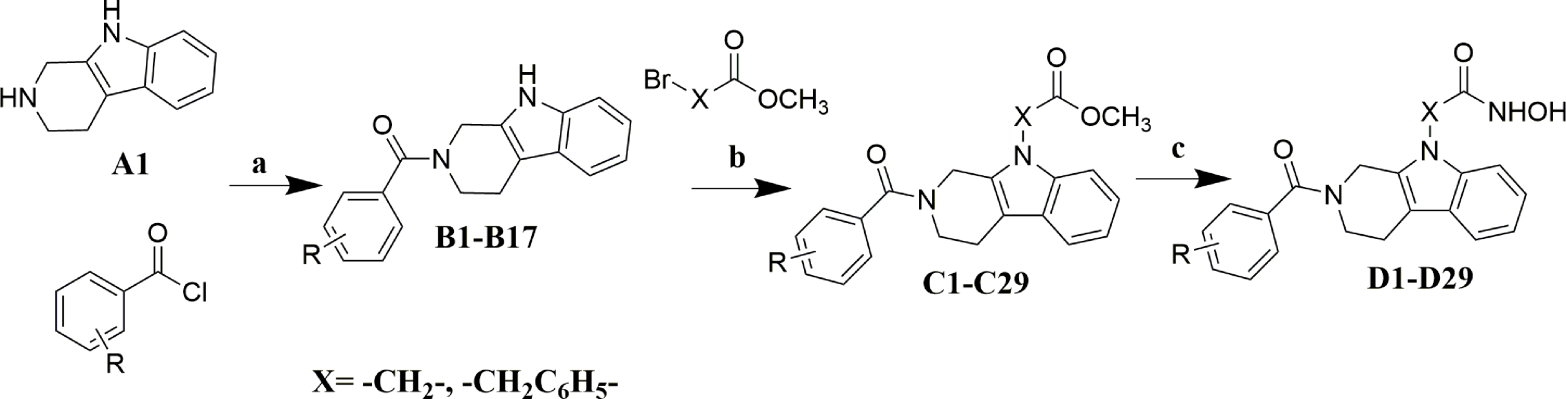
Reagents and conditions: **(A)** Et_3_N, DCM, 0°C; **(B)** Cs_2_CO_3_, DMF, 0°C; **(C)** NH_2_OK, MeOH, rt.

The synthesis of target compounds was described in **Scheme 1**. The target molecules were prepared by utilizing commercially available 2,3,4,9-tetrahydro-1H-pyrido[3,4-b]indole (**A1**) as the starting material. Firstly, compound **A1** was condensed with substituted benzoyl chlorides to obtain intermediate **B1**-**B17**. Secondly, key intermediate **C1**-**C29** were obtained by coupling of intermediate **B1**-**B17** with methyl bromoacetate or methyl 4-(bromomethyl)benzoate. At last, target molecules **D1**-**D29** were synthesized by treatment of intermediate **C1**-**C29** with NH_2_OK in methanol.

## Results and Discussions

### Enzyme Inhibitory Activity of Synthesized Molecules

The substituted carboline structures are widely utilized in the design of anticancer molecules, such as histone deacetylase (HDAC6) inhibitors (22), kinesin spindle protein inhibitors, breast cancer resistance protein (ABCG2) inhibitors (23), phosphodiesterase 5 inhibitors (24), transforming growth factor beta (TGFβ) signaling pathway inhibitors (25), and bromodomain and extra terminal proteins (BET) inhibitors (26). The carboline moiety played important roles in the site occupation and hydrophobic interaction of the ligand-receptor complexes. On the other hand, the hydrophobic pocket in the active site of APN requires hydrophobic and bulky fragments for efficient binding. Therefore, in the current study, the tetrahydro-*β*-carboline scaffold was utilized for the occupation of target compounds to the hydrophobic pocket of APN.

**Figure 2:**
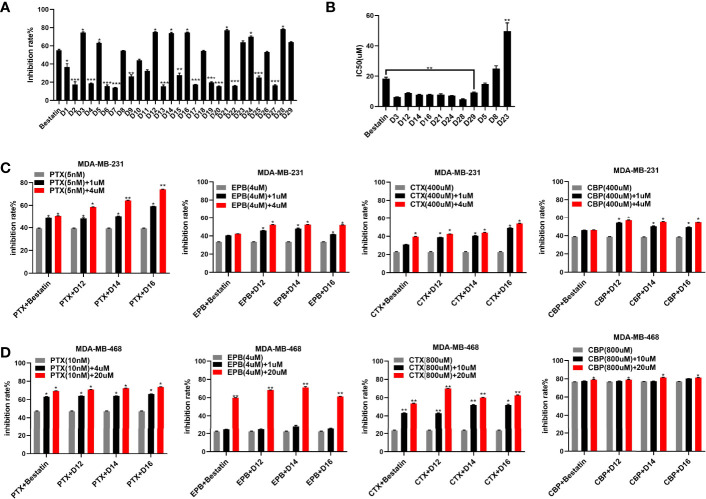
Enzyme inhibitory and antiproliferative activities of the derived APN inhibitors. a: APN enzyme inhibitory rates **(A)** of the synthesized compounds; **(B)** IC_50_ values of selected compounds; **(C)** antiproliferative activities of representative compounds in combination with chemotherapeutic drugs against MDA-MB-231 cells; **(D)** antiproliferative activities of representative compounds in combination with chemotherapeutic drugs against MDA-MB-468 cells; *P ≤ 0.05, **P ≤ 0.01, ***P ≤ 0.001.

In the enzyme inhibitory assay, K562-CD13 monoclonal cells were used as APN enzyme source as described in our previous work (27). The derived target molecules were firstly screened at the concentration of 30 µM; then, compounds with inhibitory rate higher than 55% were further investigated for the IC_50_ value (**Table 1** and **Figures 2A, B**). Notably, molecules with a short linker (CH_2_) between hydroxamic acid group and tetrahydro-*β*-carboline group exhibited better inhibitory activities than compounds with an aromatic linker (CH_2_C_6_H_4_). Several compounds exhibited increased activity compared with Bestatin (IC_50_ value 18.33 µM), such as **D3** (IC_50_ value 6.24 µM), **D14** (IC_50_ value 7.74 µM), **D16** (IC_50_ value 7.90 µM), **D21** (IC_50_ value 7.82 µM), **D24** (IC_50_ value 7.13 µM), and **D28** (IC_50_ value 4.85 µM). Comparing with **D3**, introduction of R groups by para substitution on the phenyl ring led to decreased activity as revealed in **Table 1**. Nevertheless, the fluorine substitution on the ortho and meta positions can maintain the inhibitory potency, as seen in **D12**, **D14**, **D24** and **D28**. From the present structure activity relationship analysis, substitution on the ortho position of the phenyl ring could be beneficial to the inhibitory activity, and further structural modification could be performed by targeting this site.

**Table 1:** Structures and enzyme inhibitory activities of the derived APN inhibitors.

Compound	X	R	Inhibitory rate (%)^a^	IC_50_ (µM)^a^
D1	CH_2_	-CF_3_(p)	35.0±1.51	ND
D2	CH_2_C_6_H_4_	-CF_3_(p)	13.3±3.25	ND
D3	CH_2_	-H	74.4±6.42	6.24±0.43
D4	CH_2_C_6_H_4_	-H	19.4±4.53	ND
D5	CH_2_	-CF_3_(o)	62.9±8.72	14.9±1.03
D6	CH_2_C_6_H_4_	-CF_3_(o)	15.3±3.66	ND
D7	CH_2_C_6_H_4_	-OCH_3_(o)	17.9±0.84	ND
D8	CH_2_	-OCH_3_(p)	56.7±0.42	25.0±3.32
D9	CH_2_C_6_H_4_	-OCH_3_(p)	24.0±0.25	ND
D10	CH_2_	-F(p)	42.5±1.42	ND
D11	CH_2_C_6_H_4_	-F(p)	35.0±4.57	ND
D12	CH_2_	-F(m)	74.9±4.35	8.92±0.38
D13	CH_2_C_6_H_4_	-F(m)	13.2±0.40	ND
D14	CH_2_	-F(o)	74.3±6.16	7.74±0.33
D15	CH_2_C_6_H_4_	-F(o)	28.7±2.75	ND
D16	CH_2_	-CH_3_(o)	74.5±4.66	7.9±0.27
D17	CH_2_C_6_H_4_	-CH_3_(o)	23.6±4.51	ND
D18	CH_2_	-CH_2_CH_3_(p)	53.2±2.08	ND
D19	CH_2_C_6_H_4_	-CH_2_CH_3_(p)	19.5±4.22	ND
D20	CH_2_C_6_H_4_	-Cl(o)	19.1±4.17	ND
D21	CH_2_	-Br(m)	78.2±5.43	7.82±1.05
D22	CH_2_C_6_H_4_	-Br(m)	19.2±3.70	ND
D23	CH_2_C_6_H_4_	-2,4-2F	65.0±5.97	49.74±9.29
D24	CH_2_	-3,5-2F	71.3±4.25	7.13±0.13
D25	CH_2_C_6_H_4_	-3,5-2F	27.9±5.85	ND
D26	CH_2_	-( CH_2_)_2_CH_3_(p)	53.1±7.31	ND
D27	CH_2_C_6_H_4_	-( CH_2_)_2_CH_3_(p)	20.4±5.78	ND
D28	CH_2_	-2,5-2F	78.0±3.95	4.85±0.55
D29	CH_2_	-2-Cl-6-F	60.0±4.95	9.23±0.67
Bestatin			53.9±0.77	18.33±1.68

a Each value is the mean of at least three experiments.

ND, Not determined.

### Tumor Chemo-Sensitization Ability of Selected APN Inhibitors

APN has been revealed to be overexpressed in breast cancer and the APN expression is correlated with resistance to anticancer drugs, such as doxorubicin (EPB), in breast cancer cells (6, 28). The combination of APN inhibitor and paclitaxel (PTX), has been employed for the treatment of human breast cancer. In the current study, molecules with good APN inhibitory potency (**D12**, **D14** and **D16)** were selected and used in combination with several chemotherapeutic drugs, such as PTX, EPB, cyclophosphamide (CTX) and carboplatin (CBP) to investigate its inhibitory effect on breast cancer. Human triple negative breast cancer cell (TNBC) lines MDA-MB-231 and MDA-MB-468 were utilized in the *in vitro* studies. The results revealed that the tested compounds improve the antiproliferative activities of chemotherapeutic drugs against both cell lines with superior potency than the approved APN inhibitor Bestatin (**Figures 2C, D**). It was also observed that the synthesized molecules enhanced chemotherapeutic drug sensitivity in a dose dependent manner. Therefore, further analysis was performed to examine the synergistic effects of the derived APN inhibitors and chemotherapeutic drugs.

### APN Expression, ROS Level, and Apoptosis Analysis

**Figure 3:**
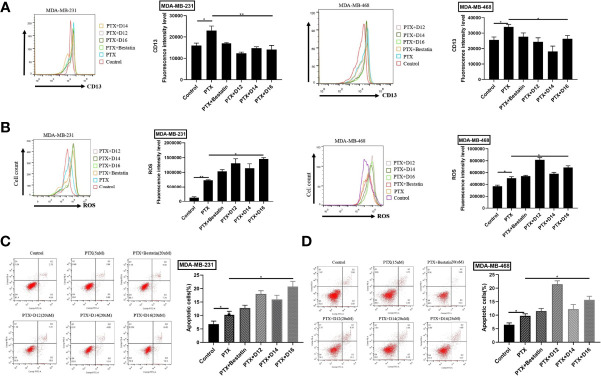
Combination of APN inhibitors with PTX decreased the expression CD13 (APN), increased ROS level, and promoted cell apoptosis. **(A)** CD13 (APN) expression regulated by various PTX-APN inhibitor combinations; **(B)** ROS level monitored by various PTX-APN inhibitor combinations; **(C)** MDA-MB-231 cell apoptosis induced by different PTX-APN inhibitor combinations; **(D)** MDA-MB-468 cell apoptosis induced by different PTX-APN inhibitor combinations; *P ≤ 0.05, **P ≤ 0.01.

PTX exhibited the highest inhibitory potency against both cell lines among the tested chemotherapeutic drugs. Moreover, APN inhibitor-induced chemo-sensitization had been frequently reported for PTX (17, 29). Therefore, the combination of PTX-APN inhibitor was selected in the subsequent studies. Enhanced APN expression was detected after treatment with the PTX relative to the untreated control, suggesting that PTX treatment up-regulated APN expression in both MDA-MB-231 and MDA-MB-468 cells (**Figure 3A**). Treatment with **D12**, **D14**, **D16** or Bestatin in combination with PTX decreased APN expression in both cell lines compared with paclitaxel treatment alone. Compared to the control group, increased ROS levels were observed in both breast cancer cell lines following PTX treatment (**Figure 3B**). Co-treatment with selected APN inhibitors resulted in further increase of intracellular ROS levels than PTX treatment alone. The apoptotic results revealed that treatment of the tested breast cancer cell lines with APN inhibitors promoted the apoptosis-inducing effect of PTX (**Figures 3C, D**). Compared with Bestatin, the synthesized compounds **D12**, **D14** and **D16** in combination with PTX treatment induced higher apoptotic rate. These results suggested that the selected APN inhibitors can sensitize TNBC cells to PTX by decreasing APN expression, increasing ROS level and induction of apoptosis.

### Cancer Stemness Inhibitory Test

**Figure 4:**
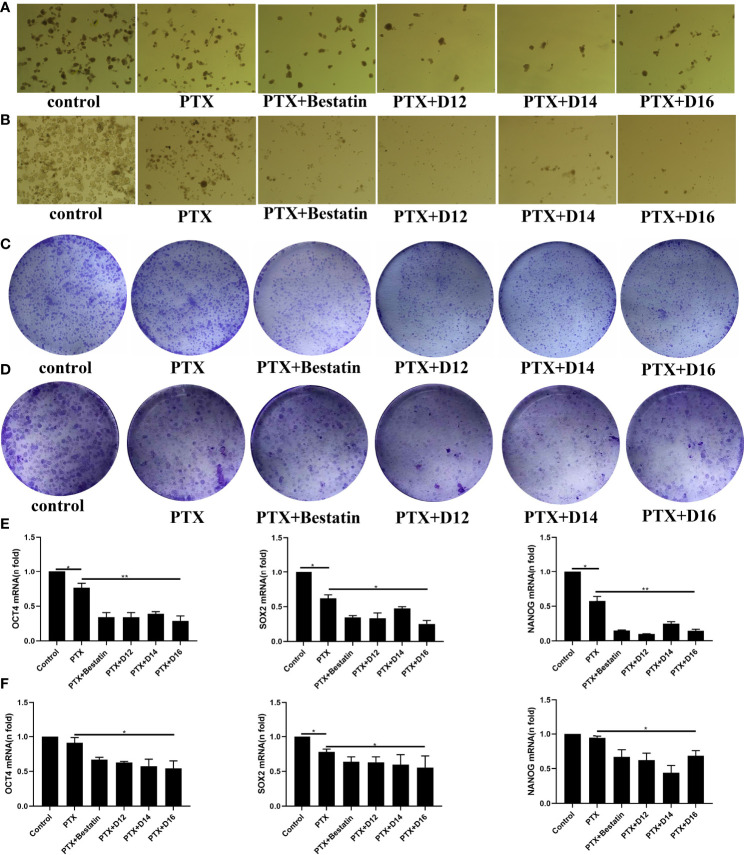
Effects of PTX-APN inhibitor combinations on spheroid and colony formation of tested TNBC cells, and on mRNA expression levels of OCT-4, SOX-2 and Nanog in the tested TNBC stem cells. **(A)** MDA-MB-231 cell spheroid formation affected by different PTX-APN inhibitor combinations; **(B)** MDA-MB-468 cell spheroid formation affected by different PTX-APN inhibitor combinations; **(C)** MDA-MB-231 cell colony formation affected by different PTX-APN inhibitor combinations; **(D)** MDA-MB-468 cell colony formation affected by different PTX-APN inhibitor combinations; **(E)** regulation of mRNA expression levels of OCT-4, SOX-2 and Nanog by different PTX-APN inhibitor combinations in MDA-MB-231 cells; **(F)** regulation of mRNA expression levels of OCT-4, SOX-2 and Nanog by different PTX-APN inhibitor combinations in MDA-MB-468 cells; *P ≤ 0.05, **P ≤ 0.01.

Cancer stem cells (CSCs) play an important role in the resistance of cancer cells to chemotherapeutic drugs. APN has been reported to be a therapeutic target in human cancer stem cells (18). Therefore, the CSC based test was performed in the present study. Colony-formation assay and spheroid-formation assay are two commonly used methods to assess the capacity of CSCs *in vitro*. In the spheroid-formation assay, MDA-MB-231 and MDA-MB-468 cells were dispersed into single cells, inoculated into low-adherence 6-well plates, and added with stem cell sphere medium. The stem cell spheres became visible after 5-6 days incubation. Stem cell sphere medium was added with 1nM of PTX in combination with 1 µM of **D12**, **D14**, **D16** or Bestatin. After 7 days of incubation, significant decrease in the number of tumor spheres formed in both MDA-MB-231 and MDA-MB-468 cells was observed in the drug combination groups comparing with the control and PTX alone groups (**Figures 4A, B**).To evaluate the effect of PTX in combination with APN inhibitors on the colony formation ability of TNBC cells, MDA-MB-231 and MDA-MB-468 cells were inoculated with 3000 cells per well in a 6-well plate. After treating with 0.2 nM of PTX combined with 1µM of various APN inhibitors for 14 days, the drug combination groups exhibited reduced number of colonies compared with the control and single PTX treatment groups (**Figures 4C, D**). It is known that mRNA levels of the stem cell biomarkers OCT-4, SOX-2 and Nanog are associated with the characteristics of TNBC stem cells (30). Therefore, RT-PCR was performed on the TNBC cells to detect the mRNA levels of these markers. The result showed that the mRNA expression levels of OCT-4, SOX-2 and Nanog in the TNBC stem cell spheres significantly decreased in the combined treatment groups compared with the control and single PTX groups (**Figures 4E, F**). These results suggested that the synthesized APN inhibitors (**D12**, **D14**, **D16**) could inhibit the stemness of TNBC cells when combined with PTX, with a higher potency than Bestatin.

### *In vivo* Anticancer Study

**Figure 5:**
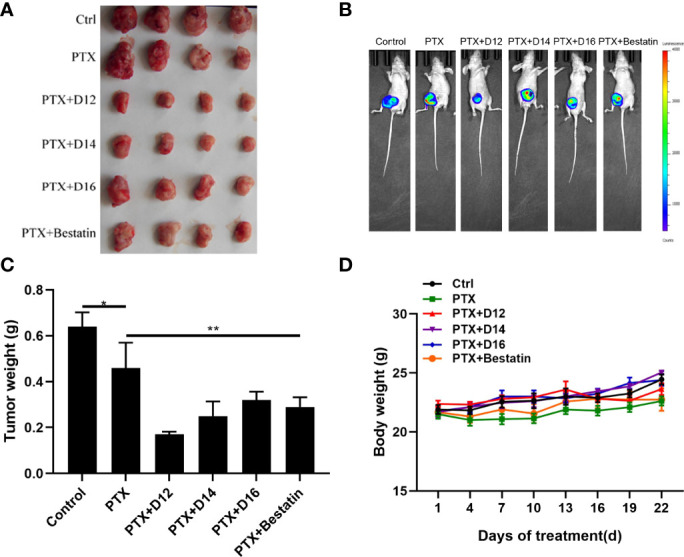
*In vivo* anticancer effects of the PTX-APN inhibitor combinations. **(A)** Dissected tumor tissues taken from mice after administration of various drug combinations; **(B)** representative *in vivo* imaging of tumors; **(C)** Tumor weight plot of different administration groups; **(D)** mice body weight plot of different administration groups; Data were taken as mean ± SEM (n = 4), *P ≤ 0.05, **P ≤ 0.01.

To evaluate the *in vivo* anticancer potency of the combination of PTX-APN inhibitor, cancer xenograft model was established by inoculation of luciferase- expressing MDA-MB-231 cells to female nude mice. About 1×10^7^ MDA-MB-231 cells were injected subcutaneously into the right fat pad of the fourth mammary gland of the mice. The tumor growth status of mice in each group was observed with a small animal imaging device. Significant tumor size variations were observed among different groups (**Figures 5A, B**). Bestatin and the APN inhibitors (**D12**, **D14**, **D16**) improved the anticancer effect of PTX compared with the single PTX administration group (**Figure 5C**). Among the tested molecules, compound **D12** in combination with PTX showed the best performance with the smallest tumor size, with an inhibitory rate of 54.02% compared with **D14** (inhibitory rate of 31.32%), **D16** (inhibitory rate of 14.15%) and Bestatin (inhibitory rate of 10.63%). It was also observed that the tested compounds inhibit the tumor growth without causing the loss in body weight (**Figure 5D**). Moreover, no clear signs of toxicity in liver and spleen were detected in the sacrificed mice. These results demonstrated that the derived APN inhibitors (especially **D12**) enhanced the anticancer effect of chemotherapeutic drug (PTX) compared with Bestatin in the *in vivo* animal model.

## Conclusion

Chemotherapy is the standard treatment for various types of cancers, such as TNBC. However, adverse effects and development of multidrug resistance has led to the search for new drugs or combinatory drug regimens for cancer therapy. APN inhibitors have been investigated as chemosensitizers and used to enhance the efficacy of standard chemotherapy. In order to develop potent chemosensitizers for the cancer treatment, novel APN inhibitors with tetrahydro-β-carboline structure were designed and synthesized in the present study. The chemo-sensitization potential of the derived molecules was evaluated.

Based on the enzymatic assay, the synthesized compounds inhibit the bioactivity of APN with various potency. Several molecules with high enzyme inhibitory activities were selected for further anticancer analysis. In the *in vitro* study, compound **D12**, **D14** and **D16** synergistically improved the antiproliferative activity of chemotherapeutic drugs, such as PTX, EPB, CTX and CBP. PTX, with the highest inhibitory potency against the tested MDA-MB-231 and MDA-MB-468 cells, were evaluated in combination with representative APN inhibitors. It is revealed that the addition of APN inhibitors decreases APN expression level, enhances intracellular ROS production, and improves apoptotic rate in the TNBC cells. The subsequent TNBC stem cell-based studies showed that introduction of APN inhibitors (**D12**, **D14** and **D16**) decreased the tumor sphere and colony formation abilities in the stem cells. The mRNA expression of stem cell markers OCT-4, SOX-2 and Nanog were decreased by combination of derived APN inhibitors to PTX. In the *in vivo* nude mouse model, the selected APN inhibitors, especially **D12**, improved the anticancer activity of PTX, as suggested by the observed smaller tumor size. Collectively, potent APN inhibitors were discovered, which can be used as lead compounds for the development of chemo-sensitizers in cancer treatment.

## Materials and Methods

All commercially available starting materials, reagents and solvents were used without further purification. All reactions were monitored by TLC with 0.25 mm silica gel plates (60GF-254). UV light and ferric chloride were used to visualize the spots. ^1^H NMR and ^13^C NMR spectra were recorded on a Bruker DRX spectrometer at 500 MHz, using TMS as an internal standard. High-resolution mass spectra was performed in Weifang Medical University. A Thermo UltiMate 3000 High performance liquid chromatograph was used to measure the purity of the derived molecules, and all the target compounds achieved > 95% purity.

Preparation of **B1** and its analogues: Derivatives **B2-B17** were prepared as described for **B1** (see below).

(1,3,4,9-tetrahydro-2*H*-pyrido[3,4-*b*]indol-2-yl)(4-(trifluoromethyl)phenyl)methanone(**B1**).
To a solution of **A1** (0.50 g, 2.9 mmol) in DCM 4-(trifluoromethyl)-benzoylchlorid (0.73 g, 3.5 mmol) and the followed Et_3_N (0.88 g, 8.7 mmol) were added dropwise at 0°C, and the mixture was stirred for 4 h. Then, the solvent was evaporated with the residue being taken up in EtOAc. The EtOAc solution was washed with saturated citric acid, NaHCO_3_ and saturated brine solution, dried over MgSO_4_, and evaporated under vacuum. The desired compound **B1** (0.87 g, 87%) was derived by crystallization in EtOAc as white powder. HRMS m/z: 345.12012[M+H]^+^. ^1^H-NMR (400 MHz, DMSO). δ 10.93 (s, 1H), 7.86 (d, *J* = 8.0 Hz, 2H), 7.70 (d, *J* = 8.0 Hz, 2H), 7.40 (d, *J* = 8.0 Hz, 1H), 7.35-7.25 (m, 1H), 7.08-7.04 (m, 1H), 7.00-6.96 (m, 1H), 4.86-4.55 (m, 2H), 4.02-3,60 (m, 2H), 2.82-2.75 (m, 2H).
Phenyl(1,3,4,9-tetrahydro-2*H*-pyrido[3,4-*b*]indol-2-yl)methanone (**B2**).
HRMS m/z: 277.13254[M+H]^+^. ^1^H-NMR (400 MHz, DMSO). δ 10.94 (s, 1H), 7.48 (d, *J* = 4.0 Hz, 4H), 7.40 (d, *J* = 8.0 Hz, 1H), 7.34-7.26 (m, 1H), 7.05-7.04 (m, 1H), 6.99-6.96 (m, 1H), 4.83-4.60 (m, 2H), 3.99-3,63 (m, 2H), 2.75 (s, 2H).
(1,3,4,9-tetrahydro-2*H*-pyrido[3,4-*b*]indol-2-yl)(2-(trifluoromethyl)phenyl)methanone (**B3**).
HRMS m/z: 345.11850[M+H]^+^. ^1^H-NMR (400 MHz, DMSO).δ 10.54 (s, 1H), 7.90-7.86 (m, 1H), 7.83-7.77 (m, 1H), 7.72-7.68 (m, 1H), 7.55-7.49 (m, 1H), 7.41-7.23 (m, 2H), 7.08-7.01 (m, 1H), 6.99-6.95 (m, 1H), 4.87 (dd, *J* = 94.0, 16.8 Hz, 1H), 4.41-4.22 (m, 1H), 3.47-3.42 (m, 2H), 2.81-2.62 (m, 2H).
(2-methoxyphenyl)(1,3,4,9-tetrahydro-2*H*-pyrido[3,4-*b*]indol-2-yl)methanone (**B4**).
HRMS m/z: 307.14319[M+H]^+^. ^1^H-NMR (400 MHz, DMSO). δ 10.61 (s, 1H), 7.46-7.32 (m, 3H), 7.25-7.20 (m, 1H), 7.18-6.95 (m, 3H), 4.84-4.37 (m, 2H), 3.80 (s, 2H), 3.72 (s, 1H), 3.50-3.45 (m, 2H), 2.79-2.62 (m, 2H).
(4-methoxyphenyl)(1,3,4,9-tetrahydro-2*H*-pyrido[3,4-*b*]indol-2-yl)methanone (**B5**).
HRMS m/z: 307.14322[M+H]^+^. ^1^H-NMR (400 MHz, DMSO). δ 10.84 (s, 1H), 7.46-7.45 (m, 2H), 7.44-7.39 (m, 1H), 7.30 (d, *J* = 8.0 Hz, 1H), 4.74 (s, 2H), 3.89-3.72 (m, 5H), 2.79-2.76 (m, 2H).
(4-fluorophenyl)(1,3,4,9-tetrahydro-2*H*-pyrido[3,4-*b*]indol-2-yl)methanone (**B6**).
HRMS m/z: 295.12253[M+H]^+^. ^1^H-NMR (400 MHz, DMSO). δ 10.94 (s, 1H), 7.57-7.53 (m, 2H), 7.40 (d, *J* = 8.0 Hz, 1H), 7.34-7.30 (m, 3H), 7.07-7.03 (m, 1H), 6.99-6.96 (m, 1H), 4.82-4.61 (m, 2H), 3.98-3.63 (m, 2H), 2.77 (s, 2H).
(3-fluorophenyl)(1,3,4,9-tetrahydro-2*H*-pyrido[3,4-*b*]indol-2-yl)methanone (**B7**).
HRMS m/z: 295.12314[M+H]^+^. ^1^H-NMR (400 MHz, DMSO). δ 10.92 (s, 1H), 7.57-7.51 (m, 1H), 7.41-7.39 (m, 1H), 7.36-7.30 (m, 4H), 7.07-7.04 (m, 1H), 6.99-6.96 (m, 1H), 4.83-4.59 (m, 2H), 3.99-3.62 (m, 2H), 2.75 (s, 2H).
(2-fluorophenyl)(1,3,4,9-tetrahydro-2*H*-pyrido[3,4-*b*]indol-2-yl)methanone (**B8**).
HRMS m/z: 295.12253[M+H]^+^. ^1^H-NMR (400 MHz, DMSO). δ 10.96 (s, 1H), 7.58-7.53 (m, 1H), 7.49-7.25 (m, 5H), 7.07-7.04 (m, 1H), 6.99-6.96 (m, 1H), 4.89-4.50 (m, 2H), 4.03-3.56 (m, 2H), 2.82-2.69 (m, 2H).
(1,3,4,9-tetrahydro-2*H*-pyrido[3,4-*b*]indol-2-yl)(o-tolyl)methanone (**B9**).
HRMS m/z: 291.14819[M+H]^+^. ^1^H-NMR (400 MHz, DMSO). δ 10.94 (s, 1H), 7.43-7.17 (m, 6H), 7.07-7.01 (m, 1H), 6.99-6.95 (m, 1H), 4.90 (d, *J* = 72.0 Hz, 2H), 4.40-4.24 (m, 2H), 3.82-3.47 (m, 2H), 2.82-2.63 (m, 2H), 2.27 (s, 2H), 2.15 (s, 1H).
(4-ethylphenyl)(1,3,4,9-tetrahydro-2*H*-pyrido[3,4-*b*]indol-2-yl)methanone (**B10**).
HRMS m/z: 305.16403[M+H]^+^. ^1^H-NMR (400 MHz, DMSO). δ 10.90 (s, 1H), 7.41-7.38 (m, 3H), 7.33-7.31 (m, 3H), 7.06-7.03 (m, 1H), 6.99-6.95 (m, 1H), 4.80-4.64 (m, 2H), 3.97-3.65 (m, 2H), 2.77 (s, 2H), 2.67 (q, *J* = 7.6 Hz, 2H),1.22 (t, *J* = 8.0 Hz, 3H).
(2-chlorophenyl)(1,3,4,9-tetrahydro-2*H*-pyrido[3,4-*b*]indol-2-yl)methanone (**B11**).
HRMS m/z: 311.09207[M+H]^+^. ^1^H-NMR (400 MHz, DMSO). δ 10.94 (s, 1H), 7.59-7.57 (m, 1H), 7.55-7.24 (m, 5H), 7.07-7.04 (m, 1H), 6.99-6.95 (m, 1H), 4.94-4.39 (m, 2H), 4.13-3.49 (m, 2H), 2.82-2.68 (m, 2H).
(3-bromophenyl)(1,3,4,9-tetrahydro-2*H*-pyrido[3,4-*b*]indol-2-yl)methanone (**B12**).
HRMS m/z: 353.02856[M+H]^+^. ^1^H-NMR (400 MHz, DMSO). δ 10.92 (s, 1H), 7.71-7.70 (m, 1H), 7.69 (s, 1H), 7.66-7.41 (m, 3H), 7.39-7.26 (m, 1H), 7.07-7.03 (m, 1H), 6.99-6.95 (m, 1H), 4.83-4.59 (m, 2H), 3.98-3.62 (m, 2H), 2.75 (s, 2H).
(2,4-difluorophenyl)(1,3,4,9-tetrahydro-2*H*-pyrido[3,4-*b*]indol-2-yl)methanone (**B13**).
HRMS m/z: 313.11172[M+H]^+^. ^1^H-NMR (400 MHz, DMSO). δ 10.93 (s, 1H), 7.58-7.50 (m, 1H), 7.44-7.19 (m, 4H), 7.08-7.02 (m, 1H), 6.99-6.95 (m, 1H), 4.87-4.50 (m, 2H), 4.02-3.56 (m, 2H), 2.81-2.69 (m, 2H).
(3,5-difluorophenyl)(1,3,4,9-tetrahydro-2*H*-pyrido[3,4-*b*]indol-2-yl)methanone (**B14**).
HRMS m/z: 313.11337[M+H]^+^. ^1^H-NMR (400 MHz, DMSO). δ 10.93 (s, 1H), 7.44-7.26 (m, 5H), 7.08-7.02 (m, 1H), 6.99-6.96 (m, 1H), 4.83-4.59 (m, 2H), 3.98-3.59 (m, 2H), 2.80-2.75 (m, 2H).
(4-propylphenyl)(1,3,4,9-tetrahydro-2*H*-pyrido[3,4-*b*]indol-2-yl)methanone (**B15**).
HRMS m/z: 319.17862[M+H]^+^. ^1^H-NMR (400 MHz, DMSO). δ 10.94 (s, 1H), 7.41-7.37 (m, 3H), 7.31-7.29 (m, 3H), 7.06-7.03 (m, 1H), 6.99-6.95 (m, 1H), 4.81-4.62 (m, 2H), 3.97-3.33 (m, 2H), 2.76 (s, 2H), 2.61 (t, *J* = 8.0 Hz, 2H), 1.67-1.85 (m, 2H), 0.93 (t, *J* = 4.0 Hz, 3H).
(2,5-difluorophenyl)(1,3,4,9-tetrahydro-2*H*-pyrido[3,4-*b*]indol-2-yl)methanone (**B16**).
HRMS m/z: 313.11273[M+H]^+^. ^1^H-NMR (400 MHz, DMSO). δ 10.97 (s, 1H), 7.43-7.38 (m, 4H), 7.35-7.27 (m, 1H), 7.08-7.03 (m, 1H), 7.00-6.96 (m, 1H), 4.88-4.52 (m, 2H), 4.04-3.57 (m, 2H), 2.76 (s, 2H), 2.82-2.71 (m, 2H).
(2-chloro-6-fluorophenyl)(1,3,4,9-tetrahydro-2*H*-pyrido[3,4-*b*]indol-2-yl)methanone (**B17**).
HRMS m/z: 329.08441[M+H]^+^. ^1^H-NMR (400 MHz, DMSO). δ 10.61 (s, 1H), 7.59-7.52 (m, 1H), 7.49-7.27 (m, 4H), 7.09-7.03 (m, 1H), 7.00-6.96 (m, 1H), 4.94-4.45 (m, 2H), 4.11-3.55 (m, 2H), 2.83-2.69 (m, 2H).

Preparation of **C1** and its analogues: Derivatives **C2-C29** were prepared as described for **C1** (see below).

Methyl 2-(2-(4-(trifluoromethyl)benzoyl)-1,2,3,4-tetrahydro-9*H*-pyrido[3,4-*b*]indol-9- yl)acetate (**C1**).
The solution of Cs_2_CO_3_ (1.17 g, 3.6 mmol) and **B1** (0.40 g, 1.2 mmol) in DMF was firstly stirred under Ar_2_ and 0°C. After 30 min, methyl bromoacetate was added. The reaction solution was stirred at room temperature for 4 h. Then, the solvent was evaporated under vacuum. The residue was extracted with EtOAc (30 mL), washed with saturated NaHCO_3_ (3×30 mL), saturated brine solution (3×30 mL), dried over MgSO_4_, and evaporated under vacuum. The desired compound **C1** (0.33 g, 66%) was derived by crystallization in EtOAc as powder. HRMS m/z: 417.14145[M+H]^+^. ^1^H-NMR (400 MHz, DMSO). δ 7.87 (d, *J* = 8.0 Hz, 2H), 7.73-7.65 (m, 2H), 7.46-7.37 (m, 2H), 7.14-7.11 (m, 1H), 7.07-7.03 (m, 1H), 5.15-4.93 (m, 2H), 4.83-4.54 (m, 2H), 4.00-3.50 (m, 2H), 3.71 (s, 3H), 2.78 (s, 2H).
Methyl 4-((2-(4-(trifluoromethyl)benzoyl)-1,2,3,4-tetrahydro-9*H*-pyrido[3,4-*b*]indol- 9-yl)methyl)benzoate (**C2**).
HRMS m/z: 493.17252[M+H]^+^. ^1^H-NMR (400 MHz, DMSO). δ 7.92 (d, *J* = 8.0 Hz, 1H), 7.86 (d, *J* = 8.0 Hz, 1H), 7.74-7.57 (m, 3H), 7.50-7.45 (m, 3H), 7.22-6.84 (m, 4H), 5.56-5.32 (m, 2H), 4.80-4.44 (m, 2H), 4.00 (s, 1H), 3.83 (s, 3H), 3.59 (s, 1H), 2.87-2.81 (m, 2H).
Methyl 2-(2-benzoyl-1,2,3,4-tetrahydro-9*H*-pyrido[3,4-*b*]indol-9-yl)acetate (**C3**).
HRMS m/z: 349.15399[M+H]^+^. ^1^H-NMR (400 MHz, DMSO). δ 7.50-7.40 (m, 7H), 7.14-7.11 (m, 1H), 7.07-7.04 (m, 1H), 5.15-4.92 (m, 2H), 4.80-4.60 (m, 2H), 3.98-3.51 (m, 5H), 2.80 (s, 2H).
Methyl 2-(2-benzoyl-1,2,3,4-tetrahydro-9*H*-pyrido[3,4-*b*]indol-9-yl)acetate (**C4**).
HRMS m/z: 425.18454[M+H]^+^. ^1^H-NMR (400 MHz, DMSO). δ 7.91-7.49 (m, 2H), 7.47-7.44 (m, 5H), 7.22 (s, 3H), 7.11 (t, *J* = 8.0 Hz, 1H), 7.05 (t, *J* = 8.0 Hz, 1H), 6.92 (s, 1H), 5.55-5.29 (m, 2H), 4.77-4.49 (m, 2H), 4.97-4.62 (m, 2H), 3.83 (s, 3H), 2.81 (s, 2H).
Methyl 2-(2-(2-(trifluoromethyl)benzoyl)-1,2,3,4-tetrahydro-9*H*-pyrido[3,4-*b*]indol-9- yl)acetate (**C5**).
HRMS m/z: 417.13935[M+H]^+^. ^1^H-NMR (400 MHz, DMSO). δ 7.86-7.67 (m, 3H), 7.58-7.56 (m, 1H), 7.48-7.33 (m, 2H), 7.14-7.10 (m, 1H), 7.09-7.03 (m, 1H), 5.15-4.67 (m, 4H), 4.38-3.86 (m, 1H), 3.72 (s, 2H), 3.49-3.36 (m, 2H), 2.83-2.61 (m, 2H).
Methyl 4-((2-(2-(trifluoromethyl)benzoyl)-1,2,3,4-tetrahydro-9*H*-pyrido[3,4-*b*]indol- 9-yl)methyl)benzoate (**C6**).
HRMS m/z: 493.17227[M+H]^+^. ^1^H-NMR (400 MHz, DMSO). δ 7.91-7.85 (m, 2H), 7.79-7.67 (m, 2H), 7.57-7.32 (m, 4H), 7.21 (d, *J* = 8.0 Hz, 1H), 7.12 (d, *J* = 8.0 Hz, 1H), 7.07-7.03 (m, 1H), 6.85 (d, *J* = 8.0 Hz, 1H), 5.57-5.35 (m, 2H), 5.07 (dd, *J* = 64.0, 16.0 Hz, 1H), 4.69-4.35 (m, 2H), 4.07-3.88 (m, 2H), 3.83 (s, 2H), 3.47-3.40 (m, 2H), 2.73-2.67 (m, 2H).
Methyl 4-((2-(2-methoxybenzoyl)-1,2,3,4-tetrahydro-9*H*-pyrido[3,4-*b*]indol-9-yl) methyl)benzoate (**C7**).
HRMS m/z: 455.19534[M+H]^+^. ^1^H-NMR (400 MHz, DMSO). δ 7.94-7.73 (m, 2H), 7.50-7.21 (m, 5H), 7.14-7.00 (m, 4H), 6.93-6.81 (m, 1H), 5.55-5.21 (m, 2H), 4.83-4.32 (m, 2H), 4.23-4.16 (m, 1H), 3.86-3.62 (m, 6H), 3.46 (d, *J* = 8.0 Hz, 1H), 2.80-2.69 (m, 2H).
methyl 2-(2-(4-methoxybenzoyl)-1,2,3,4-tetrahydro-9*H*-pyrido[3,4-*b*]indol-9-yl) acetate (**C8**).
HRMS m/z: 379.16446[M+H]^+^. ^1^H-NMR (400 MHz, DMSO). δ 7.45 (d, *J* = 8.0 Hz, 2H), 7.39 (d, *J* = 8.0 Hz, 1H), 7.13-7.12 (m, 1H), 7.06-7.01 (m, 2H), 5.08 (s, 2H), 4.72 (s, 2H), 3.82 (s, 3H), 3.66 (s, 2H), 2.81 (s, 2H).
Methyl 4-((2-(4-methoxybenzoyl)-1,2,3,4-tetrahydro-9*H*-pyrido[3,4-*b*]indol-9-yl) methyl)benzoate (**C9**).
HRMS m/z: 455.19592[M+H]^+^. ^1^H-NMR (400 MHz, DMSO). δ 7.87 (s, 2H), 7.50-7.40 (m, 4H), 7.13-6.98 (m, 6H), 5.51 (s, 2H), 4.68 (s, 2H), 3.83 (s, 3H), 3.80-3.79 (m, 5H), 2.84-2.83 (m, 2H).
Methyl 2-(2-(4-fluorobenzoyl)-1,2,3,4-tetrahydro-9*H*-pyrido[3,4-*b*]indol-9-yl)acetate (**C10**).
HRMS m/z: 367.14304[M+H]^+^. ^1^H-NMR (400 MHz, DMSO). δ 7.56 (s, 2H), 7.46-7.40 (m, 2H), 7.32 (t, *J* = 8.0 Hz, 2H), 7.14-7.10 (m, 1H), 7.07-7.03 (m, 1H), 5.15-4.94 (m, 2H), 4.78-4.60 (m, 2H), 3.95-3.62 (m, 5H), 2.80 (s, 2H).
Methyl 4-((2-(4-fluorobenzoyl)-1,2,3,4-tetrahydro-9*H*-pyrido[3,4-*b*]indol-9-yl)methyl) benzoate (**C11**).
HRMS m/z: 465.15723[M+Na]^+^. ^1^H-NMR (400 MHz, DMSO). δ 7.91-7.75 (m, 2H), 7.55 (s, 1H), 7.50-7.45 (m, 2H),7.31-7.20 (m, 2H), 7.13-7.04 (m, 4H), 6.92 (s, 1H), 5.55-5.32 (m, 2H), 4.76-4.48 (m, 2H), 3.95 (s, 1H), 3.83 (s, 3H), 3.62 (s, 1H), 2.82 (s, 2H).
Methyl 2-(2-(3-fluorobenzoyl)-1,2,3,4-tetrahydro-9*H*-pyrido[3,4-*b*]indol-9-yl)acetate (**C12**).
HRMS m/z: 367.14236[M+H]^+^. ^1^H-NMR (400 MHz, DMSO). δ 7.54 (d, *J* = 8.0 Hz, 1H), 7.45 (d, *J* = 8.0 Hz, 1H), 7.41-7.32 (m, 4H), 7.14-7.10 (m, 1H), 7.07-7.03 (m, 1H), 5.13-4.92 (m, 2H), 4.80-4.56 (m, 2H), 3.97-3.52 (m, 5H), 2.79 (s, 2H).
Methyl 4-((2-(3-fluorobenzoyl)-1,2,3,4-tetrahydro-9*H*-pyrido[3,4-*b*]indol-9-yl)methyl) benzoate (**C13**).
HRMS m/z: 443.17584[M+H]^+^. ^1^H-NMR (400 MHz, DMSO). δ 7.93-7.73 (m, 2H), 7.55-7.32 (m, 5H), 7.21-7.04 (m, 4H), 6.92 (s, 1H), 5.56-5.31 (m, 2H), 4.77-4.47 (m, 2H), 3.96 (s, 1H), 3.83 (s, 3H), 3.60 (s, 1H), 2.81 (s, 2H).
Methyl 2-(2-(2-fluorobenzoyl)-1,2,3,4-tetrahydro-9*H*-pyrido[3,4-*b*]indol-9-yl)acetate (**C14**).
HRMS m/z: 367.14447[M+H]^+^. ^1^H-NMR (400 MHz, DMSO). δ 7.55 (d, *J* = 8.0 Hz, 1H), 7.46 (d, *J* = 8.0 Hz, 1H), 7.41-7.34 (m, 4H), 7.14-7.11 (m, 1H), 7.07-7.04 (m, 1H), 5.16-4.94 (m, 2H), 4.81-4.57 (m, 2H), 3.97-3.53 (m, 5H), 2.79 (s, 2H).
Methyl 4-((2-(2-fluorobenzoyl)-1,2,3,4-tetrahydro-9*H*-pyrido[3,4-*b*]indol-9-yl)methyl) benzoate (**C15**).
HRMS m/z: 443.17517[M+H]^+^. ^1^H-NMR (400 MHz, DMSO). δ 7.93-7.75 (m, 2H), 7.57-7.45 (m, 3H), 7.34-7.32 (m, 2H), 7.22-7.04 (m, 4H), 6.93 (s, 1H), 5.56-5.32 (m, 2H), 4.77-4.47 (m, 2H), 3.97 (s, 1H), 3.83 (s, 3H), 3.61 (s, 1H), 2.81 (s, 2H).
Methyl 2-(2-(2-methylbenzoyl)-1,2,3,4-tetrahydro-9*H*-pyrido[3,4-*b*]indol-9-yl)acetate (**C16**).
HRMS m/z: 363.16928[M+H]^+^. ^1^H-NMR (400 MHz, DMSO). δ 7.48-7.23 (m, 6H), 7.15-7.10 (m, 1H), 7.09-7.02 (m, 1H), 5.15-4.28 (m, 2H), 5.01-4.72 (m, 2H), 3.72-3.47 (m, 5H), 2.82-2.66 (m, 2H), 2.09 (s, 3H).
Methyl 4-((2-(2-methylbenzoyl)-1,2,3,4-tetrahydro-9*H*-pyrido[3,4-*b*]indol-9-yl) methyl)benzoate (**C17**).
HRMS m/z: 439.20081[M+H]^+^. ^1^H-NMR (400 MHz, DMSO). δ 7.91-7.73 (m, 2H), 7.51-7.45 (m, 2H), 7.37-7.21 (m, 4H), 7.15-6.85 (m, 4H), 5.56-5.17 (m, 2H), 5.01-4.13 (m, 2H), 3.88 (s, 1H), 3.84 (t, *J* = 4.0 Hz, 2H), 3.75-3.47 (m, 2H), 2.84-2.69 (m, 2H), 2.22 (s, 2H), 1.19-1.16 (m, 1H).
Methyl 2-(2-(4-ethylbenzoyl)-1,2,3,4-tetrahydro-9*H*-pyrido[3,4-*b*]indol-9-yl)acetate (**C18**).
HRMS m/z: 377.18558[M+H]^+^. ^1^H-NMR (400 MHz, DMSO). δ 7.45 (d, *J* = 8.0 Hz, 1H), 7.40 (d, *J* = 8.0 Hz, 2H), 7.32 (d, *J* = 8.0 Hz, 2H), 7.13-7.10 (m, 1H), 7.07-7.03 (m, 1H), 5.13-4.96 (m, 2H), 4.95-4.76 (m, 2H), 3.70-3.64 (m, 5H), 2.79 (s, 2H), 2.69 (q, *J* = 7.6 Hz, 2H), 1.22 (t, *J* = 8.0 Hz, 3H).
Methyl 4-((2-(4-ethylbenzoyl)-1,2,3,4-tetrahydro-9*H*-pyrido[3,4-*b*]indol-9-yl)methyl) benzoate (**C19**).
HRMS m/z: 453.21667[M+H]^+^. ^1^H-NMR (400 MHz, DMSO). δ 7.91-7.76 (m, 2H), 7.50-7.45 (m, 2H), 7.38-7.20 (m, 4H), 7.14-6.90 (m, 4H), 5.55-5.32 (m, 2H), 4.75-4.55 (m, 2H), 3.95-3.65 (m, 5H), 2.82 (s, 2H), 2.65 (s, 2H), 1.20 (s, 3H).
Methyl 4-((2-(2-chlorobenzoyl)-1,2,3,4-tetrahydro-9*H*-pyrido[3,4-*b*]indol-9-yl)methyl) benzoate (**C20**).
HRMS m/z: 481.12839[M+H]^+^. ^1^H-NMR (400 MHz, DMSO). δ 7.94-7.73 (m, 2H), 7.58 (d, *J* = 8.0 Hz, 1H), 7.49-7.43 (m, 4H), 7.28-6.90 (m, 5H), 5.57 (s, 2H), 5.37-4.18 (m, 3H), 3.88 (s, 1H), 3.83 (s, 2H), 3.49-3.46 (m, 2H), 2.85-2.73 (m, 2H).
Methyl 2-(2-(2-bromobenzoyl)-1,2,3,4-tetrahydro-9*H*-pyrido[3,4-*b*]indol-9-yl)acetate (**C21**).
HRMS m/z: 449.04633[M+Na]^+^. ^1^H-NMR (400 MHz, DMSO). δ 7.72-7.61 (m, 2H), 7.49-7.40 (m, 4H), 7.14-7.10 (m, 1H), 7.07-7.03 (m, 1H), 5.15-4.94 (m, 2H), 4.80-4.56 (m, 2H), 3.96-3.54 (m, 5H), 2.78 (s, 2H).
methyl 4-((2-(3-bromobenzoyl)-1,2,3,4-tetrahydro-9*H*-pyrido[3,4-*b*]indol-9-yl)methyl) benzoate (**C22**).
HRMS m/z: 503.09595[M+H]^+^. ^1^H-NMR (400 MHz, DMSO). δ 7.93-7.65 (m, 3H), 7.53-7.45 (m, 4H), 7.25-7.19 (m, 2H), 7.13-7.10 (m, 1H), 7.05 (d, *J* = 8.0 Hz, 1H), 6.95 (s, 1H), 5.56-5.31 (m, 2H), 4.76-4.47 (m, 2H), 3.95-3.60 (m, 5H), 2.81 (s, 2H).
Methyl 4-((2-(2,4-difluorobenzoyl)-1,2,3,4-tetrahydro-9*H*-pyrido[3,4-*b*]indol-9-yl) methyl)benzoate (**C23**).
HRMS m/z: 461.16650[M+H]^+^. ^1^H-NMR (400 MHz, DMSO). δ 7.93-7.75 (m, 2H), 7.58-7.40 (m, 3H), 7.31 (q, *J* = 8.0 Hz, 1H), 7.24-7.20 (m, 2H), 7.14-7.03 (m, 2H), 6.98-6.92 (m, 1H), 5.55-5.35 (m, 2H), 4.81-4.36 (m, 2H), 3.99 (s, 1H), 3.85 (s, 1H), 3.82 (s, 2H), 3.58-3.55 (m, 1H), 2.84-2.76 (m, 2H).
Methyl 2-(2-(3,5-difluorobenzoyl)-1,2,3,4-tetrahydro-9*H*-pyrido[3,4-*b*]indol-9-yl) acetate (**C24**).
HRMS m/z: 385.13513[M+H5]^+^. ^1^H-NMR (400 MHz, DMSO). δ 7.46-7.40 (m, 3H), 7.35-7.26 (m, 2H), 7.18-7.10 (m, 1H), 7.07-7.03 (m, 1H), 5.15-4.96 (m, 2H), 4.80-4.55 (m, 2H), 3.96-3.56 (m, 5H), 2.78 (s, 2H).
Methyl 4-((2-(3,5-difluorobenzoyl)-1,2,3,4-tetrahydro-9*H*-pyrido[3,4-*b*]indol-9-yl) methyl)benzoate (**C25**).
HRMS m/z: 461.16635[M+H]^+^. ^1^H-NMR (400 MHz, DMSO). δ 7.93-7.74 (m, 2H), 7.50-7.38 (m, 2H), 7.25-6.94 (m, 7H), 5.56-5.34 (m, 2H), 4.76-4.43 (m, 2H), 3.95 (s, 1H), 3.83 (s, 3H), 3.59 (s, 1H), 2.80 (s, 2H).
Methyl 2-(2-(4-propylbenzoyl)-1,2,3,4-tetrahydro-9H-pyrido[3,4-b]indol-9-yl)acetate (**C26**).
HRMS m/z: 391.20087[M+H]^+^. ^1^H-NMR (400 MHz, DMSO). δ 7.46-7.39 (m, 4H), 7.31-7.29 (m, 2H), 7.13-7.10 (m, 1H), 7.06-7.03 (m, 1H), 5.13-4.95 (m, 2H), 4.87-4.77 (m, 2H), 3.91-3.52 (m, 5H), 2.79 (s, 2H), 2.63-2.60 (m, 2H), 1.67-1.58 (m, 2H), 0.92 (t, *J* = 4.0 Hz, 3H).
Methyl 4-((2-(4-propylbenzoyl)-1,2,3,4-tetrahydro-9*H*-pyrido[3,4-*b*]indol-9-yl) methyl)benzoate (**C27**).
HRMS m/z: 467.23260[M+H]^+^. ^1^H-NMR (400 MHz, DMSO). δ 7.91-7.76 (m, 2H), 7.47 (dd, *J* = 8.0, 7.6 Hz, 2H), 7.37-7.20 (m, 4H), 7.13-7.03 (m, 3H), 6.88 (s, 1H), 5.55-5.31 (m, 2H), 4.74-4.54 (m, 2H), 3.95 (s, 1H), 3.83 (s, 3H), 3.64 (s, 1H), 2.82 (s, 2H), 2.60 (s, 2H), 1.60 (s, 2H), 0.90 (s, 3H).
Methyl 2-(2-(2,5-difluorobenzoyl)-1,2,3,4-tetrahydro-9*H*-pyrido[3,4-*b*]indol-9-yl) acetate (**C28**).
HRMS m/z: 385.13501[M+H5]^+^. ^1^H-NMR (400 MHz, DMSO). δ 7.46-7.29 (m, 5H), 7.14-7.09 (m, 1H), 7.07-7.03 (m, 1H), 5.15-4.96 (m, 2H), 4.85-4.48 (m, 2H), 4.01-3.71 (m, 3H), 3.59-3.53 (m, 2H), 2.82-2.74 (m, 2H).
Methyl 2-(2-(2-chloro-6-fluorobenzoyl)-1,2,3,4-tetrahydro-9*H*-pyrido[3,4-*b*]indol-9- yl)acetate (**C29**).
HRMS m/z: 401.10547[M+H5]^+^. ^1^H-NMR (400 MHz, DMSO). δ 7.58-7.35 (m, 5H), 7.15-7.09 (m, 1H), 7.07-7.03 (m, 1H), 5.16-4.43 (m, 2H), 4.97-4.84 (m, 2H), 4.07-3.72 (m, 3H), 3.57-3.52 (m, 2H), 2.84-2.70 (m, 2H).

Preparation of **D1**and its’ analogues: **D2-D29**

*N*-hydroxy-2-(2-(4-(trifluoromethyl)benzoyl)-1,2,3,4-tetrahydro-9*H*-pyrido[3,4-*b*]indol-9-yl)acetamide (**D1**).
Compound **C1** (0.14 g, 0.6 mmol) was dissolved in 14 mL of NH_2_OK methanol solution. After 2 h of stiring, the solvent was evaporated under vacuum. The residue was acidified with saturated citric acid, and then extracted with EtOAc for 3 times. The organic layers were combined, washed with brine and dried over MgSO_4_. The desired compound **D1** (0.33 g, 54%) was derived by crystallization in EtOAc as white powder. Purity of 98.51%. HRMS (AP-ESI) m/z calcd for C_21_H_18_F_3_N_3_O_3_ [M+H]^+^ 417.13003, found: 418.13672[M+H]^+^. ^1^H-NMR (400 MHz, DMSO). δ 10.91 (d, *J* = 60.28 Hz, 1H), 9.02 (d, *J* = 46.6 Hz, 1H), 7.87 (d, *J* = 7.6 Hz, 2H), 7.72 (d, *J* = 7.6 Hz, 2H), 7.45-7.34 (m, 2H), 7.13 (t, *J* = 8.0 Hz, 1H), 7.03 (t, *J* = 8.0 Hz, 1H), 5.06-4.81 (m, 2H), 4.71-4.47 (m, 2H), 4.00-3.58 (m, 2H), 2.78 (s, 2H). ^13^C-NMR (400 MHz, DMSO). δ 169.26, 164.80, 137.52, 132.47, 128.12, 126.73, 126.14, 123.16, 121.54, 119.60, 118.22, 115.33, 109.91, 107.50, 45.78, 44.13, 21.92 ppm. HPLC (λ = 210 nm): t_R =_ 15.343 _min_ (CH_3_CN/H_2_O, 45:55).
*N*-hydroxy-4-((2-(4-(trifluoromethyl)benzoyl)-1,2,3,4-tetrahydro-9*H*-pyrido[3,4-*b*]indol-9-yl)methyl)benzamide (**D2**).
Purity of 96.32%. HRMS (AP-ESI) m/z calcd for C_27_H_22_F_3_N_3_O_3_ [M+H]^+^ 493.16133, found: 494.16742[M+H]^+^. ^1^H-NMR (400 MHz, DMSO). δ 11.28 (s, 1H), 9.07 (s, 1H), 7.87-7.60 (m, 5H), 7.58-7.39 (m, 3H), 7.15-6.82 (m, 4H), 5.51-5.25 (m, 2H), 4.83-4.54 (m, 2H), 4.02-3.59 (m, 2H), 2.87-2.81 (m, 2H). ^13^C-NMR (400 MHz, DMSO). δ 169.29, 164.33, 141.75, 140.72, 137.06, 132.29, 131.78, 130.09, 128.13, 127.85, 126.75 (d, *J* = 12.6 Hz), 126.43, 126.08, 121.83, 119.66, 118.43, 110.25, 107.83, 60.23, 46.17, 45.73, 21.95 ppm HPLC (λ = 210 nm): t_R =_ 15.497 _min_ (CH_3_CN/H_2_O, 45:55).
2-(2-benzoyl-1,2,3,4-tetrahydro-9*H*-pyrido[3,4-*b*]indol-9-yl)-*N*-hydroxyacetamide (**D3**).
Purity of 95.50%. HRMS (AP-ESI) m/z calcd for C_20_H_19_N_3_O_3_ [M+H]^+^ 349.14264, found: 350.14944[M+H]^+^. ^1^H-NMR (400 MHz, DMSO). δ 10.96 (s, 1H), 9.03 (s, 1H), 7.49-7.40 (m, 7H), 7.12 (t, *J* = 7.6 Hz, 1H), 7.03 (t, *J* = 7.2 Hz, 1H), 5.04-4.70 (m, 4H), 3.97-3.62 (m, 2H), 2.78 (s, 2H). ^13^C-NMR (400 MHz, DMSO). δ 168.76, 164.80, 137.28, 132.74, 13018, 129.03, 127.22, 126.76, 121.49, 119.57, 118.21, 109.88, 107.42, 45.92, 44.10, 22.03 ppm. HPLC (λ = 210 nm): t_R =_ 14.070 _min_ (CH_3_CN/H_2_O, 45:55).
4-((2-benzoyl-1,2,3,4-tetrahydro-9*H*-pyrido[3,4-*b*]indol-9-yl)methyl)-*N*-hydroxybenzamide (**D4**).
Purity of 97.87%. HRMS (AP-ESI) m/z calcd for C_26_H_23_N_3_O_3_ [M+H]^+^ 425.17394, found: 426.1805[M+H]^+^. ^1^H-NMR (400 MHz, DMSO). δ 11.15 (s, 1H), 9.02 (s, 1H), 7.68-7.29 (m, 9H), 7.14-6.85 (m, 4H), 5.50-5.22 (m, 2H), 4.79-4.56 (m, 2H), 3.98-3.63 (m, 2H), 2.81 (m, 2H). ^13^C-NMR (400 MHz, DMSO). δ 170.69, 164.34, 141.78, 137.04, 136.62, 132.20 (d, *J* = 28.1 Hz), 130.15, 129.00, 127.76, 127.20, 126.83, 126.72, 121.79, 119.63, 118.43, 110.21, 46.14, 45.79, 22.04 ppm. HPLC (λ = 210 nm): t_R =_ 15.087 _min_ (CH_3_CN/H_2_O, 45:55).
*N*-hydroxy-2-(2-(2-(trifluoromethyl)benzoyl)-1,2,3,4-tetrahydro-9*H*-pyrido[3,4-*b*]indol-9-yl)acetamide (**D5**).
Purity of 99.27%. HRMS (AP-ESI) m/z calcd for C_21_H_18_F_3_N_3_O_3_ [M+H]^+^ 417.13003, found: 418.13388[M+H]^+^. ^1^H-NMR (400 MHz, DMSO). δ 10.99 (s, 1H), 9.09 (s, 1H), 7.88 (d, *J* = 8.0 Hz, 1H), 7.80 (t, *J* = 8.0 Hz, 1H), 7.74-7.67 (m, 1H), 7.58-7.57 (m, 1H), 7.47-7.33 (m, 2H), 7.12(q, *J* = 8.0 Hz, 1H), 7.03 (t, *J* = 8.0 Hz, 1H), 5.15-5.06 (m, 1H), 4.78-4.72 (m, 2H), 4.60-4.14 (m, 1H), 3.86-3.38 (m, 2H), 2.82-2.60 (m, 2H). ^13^C-NMR (400 MHz, DMSO). δ 167.62, 164.75, 137.29, 135.43, 133.47 (d, *J* = 14.9 Hz), 132.19, 130.08 (d, *J* = 13.2 Hz), 127.76 (d, *J* = 12.1 Hz), 127.12 (d, *J* = 3.4 Hz), 126.65, 125.85-125.55 (m), 122.93, 121.54, 119.58, 118.27 (d, *J* = 11.6 Hz), 109.93, 107.20, 60.24, 45.31, 44.13, 21.46 ppm. HPLC (λ = 210 nm): t_R =_ 14.690 _min_ (CH_3_CN/H_2_O, 45:55).
*N*-hydroxy-4-((2-(2-(trifluoromethyl)benzoyl)-1,2,3,4-tetrahydro-9*H*-pyrido[3,4-*b*]indol-9-yl)methyl)benzamide (**D6**).
Purity of 97.92%. HRMS (AP-ESI) m/z calcd for C_27_H_22_F_3_N_3_O_3_ [M+H]^+^ 493.16133, found: 494.16602[M+H]^+^. ^1^H-NMR (400 MHz, DMSO). δ 11.18 (s, 1H), 9.05 (d, *J* = 9.6 Hz, 1H), 7.88-7.71 (m, 4H), 7.69-7.52 (m, 2H), 7.48-7.32 (m, 2H), 7.16-6.78 (m, 4H), 5.52-4.36 (m, 4H), 4.21-3.41 (m, 2H), 2.85-2.63 (m, 2H). ^13^C-NMR (400 MHz, DMSO). δ 167.59, 164.40, 141.54 (d, *J* = 24.0 Hz), 137.06 (d, *J* = 5.0 Hz), 135.01 (d, *J* = 35.6 Hz), 133.38 (d, *J* = 24.5 Hz), 132.17 (d, *J* = 35.0 Hz), 131.40 (d, *J* = 20.0 Hz), 129.99(d, *J* = 32.3 Hz), 127.82 (d, *J* = 5.4 Hz), 127.46 (d, *J* = 25.4 Hz), 127.11 (d, *J* = 4.3 Hz), 126.88, 126.63(d, *J* = 5.2 Hz), 126.40, 125.87-125.55 (m), 121.84, 119.63 (d, *J* = 5.8 Hz), 118.46 (d, *J* = 7.5 Hz), 110.18 (d, *J* = 15.1 Hz), 108.1, 107.66, 60.23, 46.08 (d, *J* = 17.8 Hz), 45.06 (d, *J* = 34.0 Hz), 21.19 (d, *J* = 59.0 Hz) ppm. HPLC (λ = 210 nm): t_R =_ 16.023 _min_ (CH_3_CN/H_2_O, 45:55).
*N*-hydroxy-4-((2-(2-methoxybenzoyl)-1,2,3,4-tetrahydro-9*H*-pyrido[3,4-*b*]indol-9-yl)methyl)benzamide (**D7**).
Purity of 98.27%. HRMS (AP-ESI) m/z calcd for C_27_H_25_N_3_O_4_ [M+H]^+^ 455.18451, found: 456.19095[M+H]^+^. ^1^H-NMR (400 MHz, DMSO). δ 11.15 (s, 1H), 9.01 (s, 1H), 7.70-7.54 (m, 2H), 7.50-7.30 (m, 3H), 7.22 (d, *J* = 8.0 Hz, 1H), 7.16-7.01 (m, 5H), 6.97-6.85 (m, 1H), 5.49-5.19 (m, 1H), 4.85-4.28 (m, 2H), 4.15-3.62 (m, 2H), 3.80-3.62 (m, 3H), 3.47 (d, *J* = 8.0 Hz, 2H), 2.80-2.69 (m, 2H). ^13^C-NMR (400 MHz, DMSO). δ 167.87 (d, *J* = 37.5 Hz), 164.28 (d, *J* = 26.1 Hz), 155.29 (d, *J* = 34.8 Hz), 141.54 (d, *J* = 47.9 Hz), 137.02 (d, *J* = 7.6 Hz), 132.26 (d, *J* = 22.1 Hz), 132.03, 130.91, 128.05, 127.80, 126.92, 126.68 (d, *J* = 6.0 Hz), 126.26, 121.74, 121.16, 119.58, 118.41, 111.89, 110.15 (d, *J* = 5.9 Hz), 108.46, 107.93, 60.24, 55.92, 46.17, 44.95, 21.58 ppm. HPLC (λ = 210 nm): t_R =_ 14.790 _min_ (CH_3_CN/H_2_O, 45:55).
*N*-hydroxy-2-(2-(2-methoxybenzoyl)-1,2,3,4-tetrahydro-9*H*-pyrido[3,4-*b*]indol-9-yl) acetamide (**D8**).
Purity of 98.39%. HRMS (AP-ESI) m/z calcd for C_21_H_21_N_3_O_4_ [M+H]^+^ 379.15321, found: 380.15985[M+H]^+^. ^1^H-NMR (400 MHz, DMSO). δ 10.92 (s, 1H), 9.02 (s, 1H), 7.47-7.43 (m, 3H), 7.38-7.31 (m, 1H), 7.12(t, *J* = 7.6 Hz, 1H), 7.05-7.01 (m, 3H), 4.83-4.65 (m, 4H), 3.82 (s, 3H), 3.73 (s, 2H), 2.80 (s, 2H). ^13^C-NMR (400 MHz, DMSO). δ 170.53, 164.69, 160.83, 140.43, 137.22, 134.46, 132.92, 129.43, 128.58, 126.78, 123.40, 121.46, 119.55, 118.21, 114.23, 109.85, 107.74, 60.23, 55.74, 44.09, 21.87, 14.53 ppm. HPLC (λ = 210 nm): t_R =_ 13.877 _min_ (CH_3_CN/H_2_O, 45:55).
*N*-hydroxy-4-((2-(4-methoxybenzoyl)-1,2,3,4-tetrahydro-9*H*-pyrido[3,4-*b*]indol-9-yl)methyl)benzamide (**D9**).
Purity of 95.09%. HRMS (AP-ESI) m/z calcd for C_27_H_25_N_3_O_4_ [M+H]^+^ 455.18451, 456.19104[M+H]^+^. ^1^H-NMR (400 MHz, DMSO). δ 11.15 (s, 1H), 9.01 (s, 1H), 7.65 (s, 2H), 7.50-7.43 (m, 4H), 7.15-6.99 (m, 6H), 5.45 (s, 2H), 4.72 (s, 2H), 3.81-3.73 (m, 5H), 2.83 (m, 2H). ^13^C-NMR (400 MHz, DMSO). δ 171.74, 164.36, 160.79, 141.68, 137.03, 132.25, 129.35, 128.40, 127.74, 126.75, 121.77, 119.62, 118.43, 114.17, 110.19, 108.11, 55.71, 46.11, 44.52, 21.95 ppm. HPLC (λ = 210 nm): t_R =_ 14.917 _min_ (CH_3_CN/H_2_O, 45:55).
2-(2-(4-fluorobenzoyl)-1,2,3,4-tetrahydro-9*H*-pyrido[3,4-*b*]indol-9-yl)-*N*-hydroxyacetamide (**D10**).
Purity of 95.65%. HRMS (AP-ESI) m/z calcd for C_20_H_18_FN_3_O_3_ [M+H]^+^ 367.13322, found: 368.13782[M+H]^+^. ^1^H-NMR (400 MHz, DMSO). δ 10.98 (s, 1H), 9.08 (s, 1H), 7.58-7.56 (m, 2H), 7.45-7.40 (m, 2H), 7.34-7.32 (m, 2H), 7.12 (t, *J* = 7.6 Hz, 1H), 7.05-7.02 (m, 1H), 5.05-4.87 (m, 2H), 4.76-4.48 (m, 2H), 3.96-3.62 (m, 2H), 2.79 (s, 2H). ^13^C-NMR (400 MHz, DMSO). δ 169.76, 164.56 (d, *J* = 36.6 Hz), 161.92, 137.22, 133.07, 129.93, 126.75, 121.51, 119.58, 118.22, 115.99 (d, *J* = 21.7 Hz), 109.87, 107.45.96, 44.10, 21.98 ppm. HPLC (λ = 210 nm): t_R =_ 14.390 _min_ (CH_3_CN/H_2_O, 45:55).
4-((2-(4-fluorobenzoyl)-1,2,3,4-tetrahydro-9*H*-pyrido[3,4-*b*]indol-9-yl)methyl)-*N*-hydroxybenzamide(**D11**).
Purity of 98.60%. HRMS (AP-ESI) m/z calcd for C_26_H_22_FN_3_O_3_ [M+H]^+^ 443.16452, found: 444.16885[M+H]^+^. ^1^H-NMR (400 MHz, DMSO). δ 11.18 (s, 1H), 9.04 (s, 1H), 7.69-7.55 (m, 3H), 7.49-7.31 (m, 4H), 7.13-7.03 (m, 3H), 9.04 (s, 2H), 5.50-5.25 (m, 2H), 4.78-4.57 (m, 2H), 3.97-3.63 (m, 2H), 2.82 (s, 2H). ^13^C-NMR (400 MHz, DMSO). δ 169.79, 164.33, 161.87, 141.76, 137.05, 132.63 (d, *J* = 67.1 Hz), 132.00, 129.88 (d, *J* = 8.5 Hz), 127.77, 126.79, 126.71, 121.81, 119.64, 118.43, 115.96 (d, *J* = 21.4 Hz), 110.21, 107.92, 60.24, 46.12, 21.62 (d, *J* = 76.9 Hz), 14.56 ppm. HPLC (λ = 210 nm): t_R =_ 15.263 _min_ (CH_3_CN/H_2_O, 45:55).
2-(2-(3-fluorobenzoyl)-1,2,3,4-tetrahydro-9*H*-pyrido[3,4-*b*]indol-9-yl)-*N*-hydroxyacetamide (**D12**).
Purity of 97.51%. HRMS (AP-ESI) m/z calcd for C_20_H_18_FN_3_O_3_ [M+H]^+^ 367.13322, found: 368.13693[M+H]^+^. ^1^H-NMR (400 MHz, DMSO). δ 10.95 (s, 1H), 9.04 (s, 1H), 7.54 (s, 1H), 7.45-7.32 (m, 5H), 7.12 (t, *J* = 7.2, 1H), 7.05-7.02 (m, 1H), 5.05-4.46 (m, 4H), 3.97-3.60(m, 2H), 2.78 (s, 2H). ^13^C-NMR (400 MHz, DMSO). δ 169.12, 164.73, 162.39 (d, *J* = 245.2 Hz), 139.06, 137.23, 132.52, 131.31, 126.73, 123.25, 121.51, 119.58, 118.23, 117.04 (d, *J* = 19.46 Hz), 114.30 (d, *J* = 22.17 Hz), 109.86, 107.44, 45.78, 44.10, 21.92 ppm. HPLC (λ = 210 nm): t_R =_ 14.407 _min_ (CH_3_CN/H_2_O, 45:55).
4-((2-(3-fluorobenzoyl)-1,2,3,4-tetrahydro-9*H*-pyrido[3,4-*b*]indol-9-yl)methyl)-*N*-hydroxybenzamide(**D13**).
Purity of 96.92%. HRMS (AP-ESI) m/z calcd for C_26_H_22_FN_3_O_3_ [M+H]^+^ 443.16452, found: 444.17139[M+H]^+^. ^1^H-NMR (400 MHz, DMSO).δ 11.15 (s, 1H), 9.01 (s, 1H), 7.69-7.55 (m, 3H), 7.53-7.45 (m, 2H), 7.34-7.20 (m, 3H), 7.19-7.03 (m, 2H), 6.88 (s, 2H), 5.50-5.24 (m, 2H), 4.79-4.54 (m, 2H), 3.98-3.61 (m, 2H), 2.81 (s, 2H). ^13^C-NMR (400 MHz, DMSO). δ 169.14, 164.38, 162.35 (d, *J* = 246.2 Hz), 141.78, 138.90, 137.04, 132.31, 131.85, 131.29, 127.76, 126.84, 126.69, 123.26, 121.81, 119.63, 118.45, 117.03 (d, *J* = 21.0 Hz), 114.28 (d, *J* = 23.2 Hz), 110.21, 107.92, 60.24, 45.93 (d, *J* = 41.3 Hz), 21.60 (d, *J* = 71.3 Hz), 14.56 ppm. HPLC (λ = 210 nm): t_R =_ 15.183 _min_ (CH_3_CN/H_2_O, 45:55).
2-(2-(2-fluorobenzoyl)-1,2,3,4-tetrahydro-9*H*-pyrido[3,4-*b*]indol-9-yl)-*N*-hydroxyacetamide (**D14**).
Purity of 98.35%. HRMS (AP-ESI) m/z calcd for C_20_H_18_FN_3_O_3_ [M+H]^+^ 367.13322, found: HRMS m/z: 368.13776[M+H]^+^. ^1^H-NMR (400 MHz, DMSO). δ 10.87 (d, *J* =63.3 Hz, 1H), 8.99 (d, *J* = 48.9 Hz, 1H), 7.58-7.28 (m, 6H), 7.14-7.09 (m, 1H), 7.05-7.01 (m, 1H), 5.04-4.44 (m, 4H), 4.00-3.54 (m, 2H), 2.81 (s, 2H). ^13^C-NMR (400 MHz, DMSO). δ 165.55, 164.74, 158.19 (d, *J* = 245.3 Hz), 137.25, 132.40, 132.01 (d, *J* = 8.2 Hz), 129.17, 126.64, 125.53 (d, *J* = 3.0 Hz), 124.86 (d, *J* = 18.1 Hz), 121.52, 119.58, 118.20, 116.38 (d, *J* = 21.0 Hz), 109.93, 107.28, 45.32, 44.13, 21.93 ppm. HPLC (λ = 210 nm): t_R =_ 14.300 _min_ (CH_3_CN/H_2_O, 45:55).
4-((2-(2-fluorobenzoyl)-1,2,3,4-tetrahydro-9*H*-pyrido[3,4-*b*]indol-9-yl)methyl)-*N*-hydroxybenzamide (**D15**).
Purity of 98.13%. HRMS (AP-ESI) m/z calcd for C_26_H_22_FN_3_O_3_ [M+H]^+^ 443.16452, found: 444.16870[M+H]^+^. ^1^H-NMR (400 MHz, DMSO). δ 11.17 (s, 1H), 9.04 (d, *J* = 13.6 Hz, 1H), 7.70-7.52 (m, 3H), 7.51-7.31 (m, 4H), 7.26-6.83 (m, 5H), 5.50-5.24 (m, 2H), 4.86-4.43 (m, 2H), 4.04-3.55 (m, 2H), 2.84-2.75 (m, 2H). ^13^C-NMR (400 MHz, DMSO). δ 165.58, 164.40, 158.18 (d, *J* = 244.2 Hz), 141.54 (d, *J* =41.4 Hz), 137.06, 132.36, 132.03 (d, *J* = 8.0 Hz), 131.74, 128.98 (d, *J* = 30.4 Hz), 127.73 (d, *J* = 15.9 Hz), 126.90, 126.56 (d, *J* = 15.6 Hz), 125.52-124.65 (m), 121.84, 119.65, 118.47 (d, *J* = 8.1 Hz), 116.38 (d, *J* = 21.0 Hz), 110.20 (d, *J* = 8.7 Hz), 108.50, 107.76, 60.24, 46.12 (d, *J* = 14.4 Hz), 44.92 (d, *J* = 70.6 Hz), 21.52(d, *J* = 89.7 Hz) ppm. HPLC (λ = 210 nm): t_R =_ 15.110 _min_ (CH_3_CN/H_2_O, 45:55).
*N*-hydroxy-2-(2-(2-methylbenzoyl)-1,2,3,4-tetrahydro-9*H*-pyrido[3,4-*b*]indol-9-yl)acetamide (**D16**).
Purity of 95.42%. HRMS (AP-ESI) m/z calcd for C_21_H_21_N_3_O_3_ [M+H]^+^ 363.15829, found: 364.16382[M+H]^+^. ^1^H NMR (400 MHz, DMSO). δ 10.90 (d, *J* = 71.4 Hz, 1H), 9.02 (d, *J* = 54.2 Hz, 1H), 7.47-7.16 (m, 6H), 7.14-7.11 (m, 1H), 7.05-7.01 (m, 1H), 5.11-4.71 (m, 3H), 4.51-4.39 (m, 1H), 3.73-3.47 (m, 2H), 2.82-2.66 (m, 2H), 2.25-2.13 (m, 3H). ^13^C-NMR (400 MHz, DMSO). δ 170.08, 164.75, 137.20 (d, *J* = 11.8 Hz), 134.22, 132.70, 130.73, 129.23, 126.70, 126.41, 125.95, 121.48, 119.55, 118.19, 109.91, 107.26, 44.85, 44.13, 21.92, 21.20, 19.06 ppm. HPLC (λ = 210 nm): t_R =_ 14. 923 min (CH_3_CN/H_2_O, 45:55).
*N*-hydroxy-4-((2-(2-methylbenzoyl)-1,2,3,4-tetrahydro-9*H*-pyrido[3,4-*b*]indol-9-yl) methyl)benzamide (**D17**).
Purity of 95.30%. HRMS (AP-ESI) m/z calcd for C_27_H_25_N_3_O_3_ [M+H]^+^ 439.18959, found: 440.19635[M+H]^+^. ^1^H-NMR (400 MHz, DMSO). δ 11.20 (s, 1H), 9.03 (d, *J* = 9.2 Hz, 1H), 7.71-7.56 (m, 2H), 7.51-7.44 (m, 2H), 7.37-7.22 (m, 3H), 7.20-7.00 (m, 3H), 6.80-6.78 (d, *J* = 8.0 HZ, 2H), 5.51-5.02 (m, 3H), 4.98 -4.20 (m, 2H), 3.74-3.48 (m, 2H), 2.84-2.68 (m, 2H), 2.23 (s, 2H). ^13^C-NMR (400 MHz, DMSO). δ 170.10, 164.38, 141.52 (d, *J* = 53.3 H), 137.03 (d, *J* = 4.4 Hz), 136.68, 133.92 (d, *J* = 62.5 Hz), 132.33, 132.04 (d, *J* = 5.3 HZ), 130.63 (d, *J* = 16.1 HZ), 129.11 (d, *J* = 26.9 HZ), 127.73 (d, *J* = 14.4 HZ), 126.78 (d, *J* = 18.4 Hz), 126.37 (d, *J* = 7.1 Hz), 125.75 (d, *J* = 46.3 Hz), 121.82 (d, *J* = 7.1 Hz), 119.61, 118.46 (d, *J* = 11.4 Hz), 110.18 (d, *J* = 7. Hz), 108.48, 107.71, 46.09 (d, *J* = 17.7 Hz), 44.81, 44.42, 21.53 (d, *J* = 83.4 Hz), 18.84 (d, *J* = 41.1 Hz) ppm. HPLC (λ = 210 nm): t_R =_ 15.820 _min_ (CH_3_CN/H_2_O, 45:55).
2-(2-(4-ethylbenzoyl)-1,2,3,4-tetrahydro-9*H*-pyrido[3,4-*b*]indol-9-yl)-*N*-hydroxyacetamide (**D18**).
Purity of 97.92%. HRMS (AP-ESI) m/z calcd for C_22_H_23_N_3_O_3_ [M+H]^+^ 377.17394, found: 378.18045[M+H]^+^. ^1^H-NMR (400 MHz, DMSO). δ 10.96 (s, 1H), 9.06 (s, 1H), 7.45-7.41 (m, 4H), 7.34-7.32 (d, *J* = 7.6 Hz, 2H), 7.14-7.11 (m, 1H), 7.06-7.02 (m, 1H), 5.04-4.70 (m, 4H), 3.95-3.64 (m, 2H), 2.79 (s, 2H), 2.68 (dd, *J* = 15.2, 7.6 Hz, 2H), 1.23 (t, *J* = 7.6 Hz, 3H). ^13^C-NMR (400 MHz, DMSO). δ 170.74, 164.72, 146.06, 137.23, 134.03, 132.81, 128.32, 127.47, 126.77, 121.47, 119.56, 118.21, 109.87, 107.45.90, 44.10, 28.49, 22.04, 15.87 ppm. HPLC (λ = 210 nm): t_R =_ 15.897 _min_ (CH_3_CN/H_2_O, 45:55).
4-((2-(4-ethylbenzoyl)-1,2,3,4-tetrahydro-9*H*-pyrido[3,4-*b*]indol-9-yl)methyl)-*N*-hydroxybenzamide (**D19**).
Purity of 98.88%. HRMS (AP-ESI) m/z calcd for C_28_H_27_N_3_O_3_ [M+H]^+^ 453.20524, found: 454.21204[M+H]^+^. ^1^H-NMR (400 MHz, DMSO). δ 11.15 (s, 1H), 9.01 (s, 1H), 7.67 (s, 2H), 7.50-7.46 (m, 2H), 7.44-7.39 (m, 3H), 7.13-6.85 (m, 5H), 5.50-5.26 (m, 2H), 4.77-4.61 (m, 2H), 3.97-3.65 (m, 2H), 2.82 (s, 2H), 2.68-2.65 (m, 2H), 1.21-1.16 (m, 3H). ^13^C-NMR (400 MHz, DMSO). δ 170.82, 164.38, 146.03, 141.74, 137.04, 133.91, 132.14, 128.29, 127.75, 127.41, 126.73, 121.78, 119.62, 118.42, 110.20, 108.02, 60.24, 46.12, 28.46, 22.08, 21.24, 15.82 ppm. HPLC (λ = 210 nm): t_R =_ 17.653 _min_ (CH_3_CN/H_2_O, 45:55).
4-((2-(2-chlorobenzoyl)-1,2,3,4-tetrahydro-9*H*-pyrido[3,4-*b*]indol-9-yl)methyl)-*N*-hydroxybenzamide (**D20**).
Purity of 99.27%. HRMS (AP-ESI) m/z calcd for C_26_H_22_ClN_3_O_3_ [M+H]^+^ 459.13497, found: 460.13953[M+H]^+^. ^1^H-NMR (400 MHz, DMSO) δ 11.16 (s, 1H), 9.03 (d, *J* = 10.3 Hz, 1H), 7.69 (d, *J* = 8.0 HZ, 1H), 7.59-7.55 (m, 1H), 7.52-7.42 (m, 4H), 7.34-7.21 (m, 3H), 7.16-7.02 (m, 2H), 6.83 (d, *J* = 8.0 HZ, 1H), 5.51-5.15 (m, 2H), 4.96-4.25 (m, 2H), 3.87-3.48 (m, 2H), 2.85-2.73 (m, 2H). ^13^C-NMR (400 MHz, DMSO). δ 167.07, 166.66, 137.06, 136.19 (d, *J* = 37.9 Hz), 131.71, 130.96 (d, *J* = 31.6 Hz), 129.67 (d, *J* = 21.1 Hz), 128.27 (d, *J* = 15.8 Hz), 127.71 (d, *J* = 16.3 Hz), 126.90, 126.54 (d, *J* = 15.9 Hz), 121.83, 119.64, 118.46 (d, *J* = 8.5 Hz), 110.17 (d, *J* = 12.5 Hz), 108.43, 107.74, 46.18, 44.60 (d, *J* = 65.3 Hz), 21.90, 21.10 ppm. HPLC (λ = 210 nm): t_R =_ 14.690 _min_ (CH_3_CN/H_2_O, 45:55).
2-(2-(3-bromobenzoyl)-1,2,3,4-tetrahydro-9*H*-pyrido[3,4-*b*]indol-9-yl)-*N*-hydroxyacetamide (**D21**).
Purity of 97.53%. HRMS (AP-ESI) m/z calcd for C_20_H_18_BrN_3_O_3_ [M+H]^+^ 427.05315, found: 428.05792[M+H]^+^. ^1^H-NMR (400 MHz, DMSO). δ 10.92 (d, *J* = 47.3 Hz, 1H), 9.03 (d, *J* = 38.0 Hz, 1H), 7.76-7.67 (m, 2H), 7.49-7.34 (m, 4H), 7.14-7.11 (m, 1H), 7.05-7.02 (m, 1H), 5.05-4.77 (m, 2H), 4.70-4.47 (m, 2H), 3.96-3.60 (m, 2H), 2.77 (s, 2H). ^13^C-NMR (400 MHz, DMSO). δ 139.22, 138.25, 133.01, 131.39, 130.10, 126.72, 122.43, 121.52, 119.59, 118.25, 109.92, 107.34, 55.77, 44.11, 21.95 ppm. HPLC (λ = 210 nm): t_R =_ 15.120 _min_ (CH_3_CN/H_2_O, 45:55).
4-((2-(3-bromobenzoyl)-1,2,3,4-tetrahydro-9*H*-pyrido[3,4-*b*]indol-9-yl)methyl)-*N*-hydroxybenzamide (**D22**).
Purity of 97.45%. HRMS (AP-ESI) m/z calcd for C_26_H_22_BrN_3_O_3_ [M+H]^+^ 503.08445, found: 504.08893[M+H]^+^. ^1^H-NMR (400 MHz, DMSO). δ 11.15 (s, 1H), 9.01 (s, 1H), 7.68 (d, J = 8.4 HZ, 3H), 7.59 (s, 1H), 7.49-7.26 (m, 4H), 7.14-6.91 (m, 4H), 5.50-5.25 (m, 2H), 4.79-4.55 (m, 2H), 3.97-3.61 (m, 2H), 2.81 (s, 2H). ^13^C-NMR (400 MHz, DMSO). δ 168.91, 164.39, 141.83, 138.94, 137.04, 132.97, 132.31, 131.83, 129.89, 127.77, 126.82, 126.68, 126.12, 122.26, 121.82, 119.63, 118.45, 110.21, 107.87, 108, 46.14, 45.77, 21.95 ppm. HPLC (λ = 210 nm): t_R =_ 16.097 _min_ (CH_3_CN/H_2_O, 45:55).
4-((2-(2,4-difluorobenzoyl)-1,2,3,4-tetrahydro-9*H*-pyrido[3,4-*b*]indol-9-yl)methyl)-*N*-hydroxybenzamide (**D23**).
Purity of 95.67%. HRMS (AP-ESI) m/z calcd for C_26_H_21_F_2_N_3_O_3_ [M+H]^+^ 461.15510, found: HRMS m/z: 462.16037[M+H]^+^. ^1^H-NMR (400 MHz, DMSO). δ 11.16 (s, 1H), 9.02 (s, 1H), 7.70-7.53 (m, 2H), 7.50-7.33 (m, 3H), 7.24-7.03 (m, 4H), 6.98-6.65 (m, 1H), 5.54-5.28 (m, 2H), 4.84-4.34 (m, 2H), 3.39-3.56 (m, 2H), 2.84 (m, 2H). ^13^C-NMR (400 MHz, DMSO). δ 164.66 (d, *J* = 42.5 Hz), 161.99 (d, *J* = 12.3 Hz), 159.90 (d, *J* = 12.3 Hz), 157.44 (d, *J* = 12.7 Hz), 141.60 (d, *J* = 26.1 Hz), 137.06, 132.25 (d, *J* = 18.6 Hz), 131.68, 127.71(d, *J* = 18.6 Hz), 126.89, 126.55 (d, *J* = 15.0 Hz), 121.85, 121.32 (d, *J* = 18.5 Hz), 119.66, 118.48 (d, *J* = 7.8 Hz), 112.74 (d, *J* = 17.5 Hz), 110.23, 108.46, 107.76, 105.05 (d, *J* = 17.4 Hz), 46.09 (d, *J* = 18.2 Hz), 45.34, 44.61, 21.51 (d, *J* = 94.3 Hz) ppm. HPLC (λ = 210 nm): t_R =_ 16.067 _min_ (CH_3_CN/H_2_O, 45:55).
2-(2-(3,5-difluorobenzoyl)-1,2,3,4-tetrahydro-9*H*-pyrido[3,4-*b*]indol-9-yl)-*N*-hydroxyacetamide (**D24**).
Purity of 95.49%. HRMS (AP-ESI) m/z calcd for C_20_H_17_F_2_N_3_O_3_ [M+H]^+^ 385.12380, found: 386.12924[M+H]^+^. ^1^H-NMR (400 MHz, DMSO). δ 10.92 (d, *J* = 50.0 Hz, 1H), 9.03 (d, *J* = 39.1 Hz, 1H), 7.45-7.33 (m, 3H), 7.27-7.26 (m, 2H), 7.12 (t, *J* = 7.2 Hz, 1H), 7.05-7.02 (m, 1H), 5.05-4.78 (m, 2H), 4.70-4.49 (m, 2H), 3.96-3.59 (m, 2H), 2.77 (s, 2H). ^13^C-NMR (400 MHz, DMSO). δ 167.91, 164.75, 161.51, 137.23, 137.50 (d, *J* = 40.0 Hz), 130.75, 126.70, 121.53, 119.59, 118.27, 110.71 (d, *J* = 26.5 Hz), 109.87, 107.48, 45.70, 44.11, 21.83 ppm. HPLC (λ = 210 nm): t_R =_ 14.477 _min_ (CH_3_CN/H_2_O, 45:55).
4-((2-(3,5-difluorobenzoyl)-1,2,3,4-tetrahydro-9*H*-pyrido[3,4-*b*]indol-9-yl)methyl)-*N*-hydroxybenzamide (**D25**).
Purity of 95.87%. HRMS (AP-ESI) m/z calcd for C_26_H_21_F_2_N_3_O_3_ [M+H]^+^ 461.15510, found: 462.15964[M+H]^+^. ^1^H-NMR (400 MHz, DMSO). δ 11.16 (s, 1H), 9.01 (s, 1H), 7.70-7.57 (m, 2H), 7.49-7.40 (m, 3H), 7.26-6.93 (s, 2H), 7.24-7.03 (m, 4H), 5.50-5.27 (m, 2H), 4.79-4.54 (m, 2H), 3.97-3.59 (m, 2H), 2.80 (s, 2H). ^13^C-NMR (400 MHz, DMSO). δ 167.94, 164.37, 164.01 (d, *J* = 11.5 Hz), 161.54 (d, *J* = 12.0 Hz),141.77, 140.15, 137.06, 132.33, 131.62, 127.78, 126.85, 126.67, 121.83, 119.64, 118.46, 110.71 (d, *J* = 26.6 Hz), 110.21, 107.97, 105.60, 105.50 (d, *J* = 51.5 Hz), 60.24, 45.91 (d, *J* = 47.8 Hz), 21.55 (d, *J* = 64.1 Hz), 14.55 ppm. HPLC (λ = 210 nm): t_R =_ 15. 933 min (CH_3_CN/H_2_O, 45:55).
*N*-hydroxy-2-(2-(4-propylbenzoyl)-1,2,3,4-tetrahydro-9*H*-pyrido[3,4-*b*]indol-9-yl)acetamide (**D26**).
Purity of 98.35%. HRMS (AP-ESI) m/z calcd for C_23_H_25_N_3_O_3_ [M+H]^+^ 391.1859, found: 392.19434[M+H]^+^. ^1^H-NMR (400 MHz, DMSO). δ 10.97 (s, 1H), 9.07 (s, 1H), 7.45-7.40 (m, 4H), 7.31-7.30 (m, 2H), 7.12 (t, *J* = 8.0 Hz, 1H), 7.05-7.02 (m, 1H), 5.04-4.85 (m, 2H), 4.69-4.47 (m, 2H), 3.94-3.63 (m, 2H), 2.78 (s, 2H), 2.62 (t, *J* = 8.0 Hz, 2H), 1.68-1.58 (m, 2H), 0.92 (t, *J* = 8.0 Hz, 3H). ^13^C-NMR (400 MHz, DMSO). δ 170.81, 164.72, 144.47, 137.23, 134.05, 132.81, 128.88, 127.38, 126.77, 121.46, 119.55, 118.21, 109.87, 107.46, 60.23, 45.89, 44.10, 37.52, 24.37, 21.62 (d, *J* = 76.9 Hz), 14.14 ppm. HPLC (λ = 210 nm): t_R =_ 16.550 _min_ (CH_3_CN/H_2_O, 45:55).
*N*-hydroxy-4-((2-(4-propylbenzoyl)-1,2,3,4-tetrahydro-9*H*-pyrido[3,4-*b*]indol-9-yl)methyl)benzamide (**D27**).
Purity of 99.31%. HRMS (AP-ESI) m/z calcd for C_29_H_29_N_3_O_3_ [M+H]^+^ 467.22089, found: 468.22525[M+H]^+^. ^1^H-NMR (400 MHz, DMSO). δ 11.15 (s, 1H), 9.01 (s, 1H), 7.67 (s, 2H), 7.49-7.43 (s, 2H), 7.38-7.29 (m, 3H), 7.12-6.85 (m, 5H), 5.49-5.24 (m, 2H), 4.77-4.59 (m, 2H), 3.96-3.64 (m, 2H), 2.82 (s, 2H), 2.60 (s, 2H), 1.60 (s, 2H), 0.90 (s, 3H). ^13^C-NMR (400 MHz, DMSO). δ 170.82, 164.34, 144.44, 141.75, 137.03, 133.93, 132.27, 132.13, 128.85, 127.75, 127.31, 126.78, 121.78, 119.62, 118.43, 110.19, 107.74, 60.24, 45.99 (d, *J* = 24.6 Hz),37.48, 24.34, 21.65 (d, *J* = 83.3 Hz), 14.56, 14.11 ppm. HPLC (λ = 210 nm): t_R =_ 17.960 _min_ (CH_3_CN/H_2_O, 45:55).
2-(2-(2,5-difluorobenzoyl)-1,2,3,4-tetrahydro-9*H*-pyrido[3,4-*b*]indol-9-yl)-*N*-hydroxyacetamide (**D28**).
Purity of 95.86%. HRMS (AP-ESI) m/z calcd for C_20_H_17_F_2_N_3_O_3_ [M+H]^+^ 385.12380, found: 386.13004[M+H]^+^. ^1^H-NMR (400 MHz, DMSO). δ 10.91 (d, *J* = 56.9 Hz, 1H), 9.03 (d, *J* = 43.9 Hz, 1H), 7.47-7.35 (m, 5H), 7.15-7.10 (m, 1H), 7.04 (t, *J* = 8.0 Hz, 1H), 5.05-4.83 (m, 2H), 4.71-4.48 (m, 2H), 4.00-3.57 (m, 2H), 2.74 (s, 2H). ^13^C-NMR (400 MHz, DMSO). δ 164.73, 164.31 (d, *J* = 29.9 Hz), 158.63 (d, *J* = 242.3 Hz), 154.36 (d, *J* = 240.8 Hz), 137.25, 132.19, 126.63, 126.20 (d, *J* = 10.8 Hz), 121.56, 118.28 (d, *J* = 8.8 Hz), 115.73 (d, *J* = 25.8 Hz), 109.89, 107.97, 107.33, 45.34, 44.13, 21.89, 21.06 ppm. HPLC (λ = 210 nm): t_R =_ 14.470 _min_ (CH_3_CN/H_2_O, 45:55).
2-(2-(2-chloro-6-fluorobenzoyl)-1,2,3,4-tetrahydro-9H-pyrido[3,4-b]indol-9-yl)-N-hydroxyacetamide (**D29**).
Purity of 99.09%. HRMS (AP-ESI) m/z calcd for C_20_H_17_ClFN_3_O_3_ [M+H]^+^ 401.09425, found: 402.09875[M+H]^+^. ^1^H NMR (400 MHz, DMSO). δ 10.91 (d, *J* = 65.9, 1H), 9.03 (dd, *J* = 50.4, 1H), 7.60-7.56 (m, 1H), 7.54-7.32 (m, 4H), 7.15-7.10 (m, 1H), 7.03 (t, *J* = 7.6 Hz, 1H), 4.99 (dd, *J* = 42.0, 16.8 Hz, 2H), 4.71-4.47 (m, 2H), 4.04-3.54 (m, 2H), 2.82-2.67 (m, 2H). ^13^C-NMR (400 MHz, DMSO). δ 164.73, 162.29, 158.58 (d, *J* = 247.4 Hz), 137.29, 132.25 (d, *J* = 9.6 Hz), 132.08, 131.01 (d, *J* = 6.1 Hz), 126.45 (d, *J* = 21.6 Hz), 124.78 (d, *J* = 23.3 Hz), 121.61, 119.62, 118.25, 115.50 (d, *J* = 21.2 Hz), 109.86 (d, *J* = 14.6 Hz), 107.91, 44.92, 44.17, 22.07, 21.15 ppm. HPLC (λ = 210 nm): t_R =_ 16.050 _min_ (CH_3_CN/H_2_O, 45:55).

### Cell Culture

Human K562 myeloid lymphoblastoma cell line, human triple-negative breast cancer cell lines MDA-MB-231 and MDA-MB-468 were received from the Cell Bank of Shanghai (Shanghai in China) and maintained in (RPMI)-1640 supplemented with 10% fetal calf serum (FCS). The cells were incubated at 37°C in a humidified atmosphere containing 5% CO_2_. Ubenimex (Cat. B8385) and 2′, 7′-Dichlorofluorescin diaceta (DCFH-DA) were purchased from Sigma. Paclitaxel (PTX, Cat. SP8020) and doxorubicin (Epirubicin Hydrochloride, Cat. IE 0640) were purchased from Solarbio. Carboplatin (CBP, Cat.C-0219027) and Cyclophosphamide (CTX, Cat.C-0232450) were purchased from HEOWNS. All the compounds were dissolved in sterile dimethyl sulfoxide (DMSO) to constitute 20 mM stock agents and the aliquots were stored at -20°C.

### Enzyme Activity Screening Assay

K562 cells with overexpression of APN were added into PBS for ultrasonic crushing, centrifuged for 10 minutes, and supernatant was taken as enzyme source and added into 96-well plate. Different concentrations of compounds were added subsequently, and 1.6 mM L-leucine-p-nitro anilide was used as substrate. After 30 minutes of reaction, the APN enzyme activity was measured at 405 nm absorbance. The inhibition rate of APN activity was analyzed by the formula (Od control OD tested)/Od control 100%.

### MTT Assay

MDA-MB-231 and MDA-MB-468 cells were added into 96-well plates (5X103 cells/well) and treated with PTX, PTX+Bestatin, or PTX+ various compounds at 37°C, 5% CO_2_ for 48h. Then, 10μL of MTT solution (5 mg/mL) were added to each well. After incubation for 4h, the medium was carefully removed, and 100 μL DMSO was added to each well and fully dissolved in vibration for 10min. The optical density (OD) at 490 nm and 630 nm were measured with a microplate Spectrophotometer (M5, MD). IC_50_ values were obtained from at least 3 independent experiments.

### Flow Cytometry of APN

The cells were seeded in 6-well plates (4 × 105 cells/well) and treated with tested compounds for 48 h. Then, cells were collected into 1.5 mL EP tubes, washed twice with PBS, and centrifuged at 2500rpm for 5minutes. Cells were resuspended with 100μlof PBS, then 2μL/tube of APN antibody was added, followed by incubation in dark for 30 minutes at 4°C. Then the cells were subjected to flow cytometry (FACSAria II, Becton-Dickinson) for the expression of APN.

### Cellular Reactive Oxygen Species Detection

The cells were seeded in 6-well plates (4 × 10^5^ cells/well) and treated with tested compounds for 48 h. Then, cells were resuspended with 500 μL of serum-free culture medium; after addition of10μMof DCFH-DA, cells were returned to the incubator for 30min. Finally, the cells were resuspended with PBS and subjected to flow cytometry.

### Apoptosis Analysis

The cells were seeded in 6-well plates (3 × 10^5^ cells/well) and treated with various compounds for 48 h. Then, cells were collected and washed twice with cold PBS. Annexin V-FITC staining solution was then added to the cells, and cells were incubated at 2-8°C for 15 minutes under dark conditions. Then, PI staining solution was added by gentle mix at 2-8°C. After 5 minutes of incubation under dark conditions, cells were tested with flow cytometry immediately.

### Colony-Formation Assay

3000 cells were suspended in 2 mL medium, and seeded into the wells of six-well plate. After 12 hours of incubation, cells were treated with various compounds. Until each colony had more than 50 cells, this experiment terminated. Cells were washed twice with PBS and fixed with methanol for 10minutes. Then cells were stained with 1% crystal violet for 5minutes. Finally, colonies were washed and pictures were taken under a microscope (IX81, Olympus).

### Spheroid-Formation Assay

MDA-MB-231 cells and MDA-MB-468 cells in logarithmic growth phase were collected, stem cell sphere culture medium without serum (containing 50X B-27Supplement,100X N-2 Supplement,20 μg/L epidermal growth factor (EGF), 20µg/L insulin-like growth factor (IGF-1), 10 μg/L fibroblast growth factor (b-FGF), 20X Knock Out SR, 2mM L-Glutamine, 5mg/L Heparin sodium, 1X PENICILLIN-STREPTOMYCIN and DMEM: F12 (1:1) medium was added for the re-suspension and fully blown into single-cell suspension. The cells were diluted to 5,000/2 mL, with stem cell sphere culture medium, and cultured in a 37°C, 5% CO_2_ cell incubator. The number of stem cell spheres were observed every day. After about five days, stem cell microspheres could be visually confirmed, then various compounds were added every two days for ten days.

### Quantitative Real-Time Reverse Transcription Polymerase Chain Reaction

Total RNA was extracted with TRIzol reagent, and the RNA concentration was detected by a Nano Drop instrument (2000C, NanoDrop). Single-strand cDNA was reverse-transcribed with a Hi Fi Script cDNA Synthesis Kit (CWBIO, China) following the manufacturer-provided instruction. The cDNA was amplified with a SYBR Green mixture (CWBIO, China) and gene-specific primers (**Table 2**). The primers used for QRT-PCR were OCT-4, SOX-2 and Nanog. The relative gene expression was calculated with the 2^-△△Ct^ method.

**Table 2:** Primers in the RT-PCR study.

Gene	Forward (5’-3’)	Reverse (5’-3’)
SOX2	GACTTCACATGTCCCAGCACTA	CTCTTTTGCACCCCTCCCATT
NANOG	CCCCAGCCTTTACTCTTCCTA	CCAGGTTGAATTGTTCCAGGTC
OCT4	GAGAAGGATGTGGTCCGAGT	GTGCATAGTCGCTGCTTGAT

### *In vivo* Imaging of Mice

Female Nude mice aged 3 weeks were purchased from Hunan SJA Experimental Animal Company and raised under SPF aseptic conditions. About 1×10^7^ luciferase overexpressing MDA-MB-231 cells were injected subcutaneously into the right fat pad of the fourth mammary gland of each mouse. When the tumor volume reached100 mm^3^, the mice were randomly divided into six groups (n = 4): PBS group, PTX group, PTX+Bestatin group, PTX+**D12** group, PTX+**D14** group, PTX+**D16** group. PBS group animals received PBS *via* intraperitoneal injection, while 10 mg/kg/3 days of PTX was administered to animals in the PTX group. 20 mg/kg/d of Bestatin, **D12**, **D14**, **D16**were administered in combination to10 mg/kg/3 days of PTX to the corresponding mice. After 3 weeks of administration, each mouse was intraperitoneally injected with 200uL of fluorescein substrate (d-fluorescein potassium salt, 150mg/kg), and then anesthetized with isoflurane. *In vivo* fluorescence imaging system (IVIS Spectrum, PerkinElmer) and SlideBook4.0 software were utilized for the result analysis. After the imaging analysis, mice were sacrificed and dissected for the detection of tumor size and calculation of liver/spleen indexes. All animal experiments were approved by the Animal Care and Use Committee of Weifang Medical University.

## Statistical Analysis

All statistical analyses were performed with GraphPad Prism statistical software package. Quantitative data were presented as mean ± standard error of the mean (SEM). Statistical analysis was performed by one-way analysis of variance (ANOVA). When ANOVA returns significant results, post-hoc least significant different (LSD) tests were used be compare among the groups. P < 0.05 was considered statistically significant in all experiments.

## Data Availability Statement

The original contributions presented in the study are included in the article/**Supplementary Material**. Further inquiries can be directed to the corresponding authors.

## Ethics Statement

The animal study was reviewed and approved by Animal Care and Use Committee of Weifang Medical University (ACUC-WFMU).

## Author Contributions

LEZ designed the project. XX and YH performed the enzymatic screening. FL synthesized the molecules. LIZ, QH and HQ performed the *in vitro* and *in vivo* anticancer experiments. QJ, CF and WJ analyzed the data and wrote the manuscript. All authors contributed to the article and approved the submitted version.

## Funding

This work was supported by Science and technology support plan for youth innovation in universities of Shandong Province (Grant No. 2019KJM001), Natural Foundation of Shandong Province (Youth Found, Grant No. ZR2019QH005), National Natural Science Foundation of China (Youth Found, Grant No. 81803343).

## Conflict of Interest

The authors declare that the research was conducted in the absence of any commercial or financial relationships that could be construed as a potential conflict of interest.

## Publisher’s Note

All claims expressed in this article are solely those of the authors and do not necessarily represent those of their affiliated organizations, or those of the publisher, the editors and the reviewers. Any product that may be evaluated in this article, or claim that may be made by its manufacturer, is not guaranteed or endorsed by the publisher.
