# Supplementary material for: Discovery of Novel Tetrahydro-β-carboline Containing Aminopeptidase N Inhibitors as Cancer Chemosensitizers
Source: Front Oncol. 2022 May 23;12:894842. doi: 10.3389/fonc.2022.894842 (PMC9168271; doi:10.3389/fonc.2022.894842)
Supplement: Supplementary file 1 [file DataSheet_1.pdf]

Discovery of Novel Tetrahydro-β-carboline Containing Aminopeptidase N Inhibitors as Cancer Chemosensitizers

Xiaoyan Xing [a†], Fahui Li[b†], Yajie Hu[b†], Lin Zhang[b], Qian Hui[b], Hongyu Qin[b], Qixiao Jiang[c], Wenyan Jiang[b], Chunyan Fang[a]* and Lei Zhang[b]*

[a] *Department of Pharmacology, School of Pharmacy, Weifang Medical University, Weifang, Shandong, China;*

[b] *Department of Medicinal Chemistry, School of Pharmacy, Weifang Medical University, Weifang, Shandong, China;*

[c] *Department of Toxicology, School of Public Health, Qingdao University, Qingdao, Shandong, China.*

[†]*These authors have contributed equally to this work.*

Author for correspondence: Chunyan Fang, 13853660344@163.com; Lei Zhang, Tel./fax: +86-536-8462014, E-mail: leizhangchemical@gmail.com.

Contents

HRMS and ^{1}H-NMR spectrum of **B1**......6

HRMS and ^{1}H-NMR spectrum of **B2**......7

HRMS and ^{1}H-NMR spectrum of **B3**......8

HRMS and ^{1}H-NMR spectrum of **B4**......9

HRMS and ^{1}H-NMR spectrum of **B5**......10

HRMS and ^{1}H-NMR spectrum of **B6**......11

HRMS and ^{1}H-NMR spectrum of **B7**......12

HRMS and ^{1}H-NMR spectrum of **B8**......13

HRMS and ^{1}H-NMR spectrum of **B9**......14

HRMS and ^{1}H-NMR spectrum of **B10**......15

HRMS and ^{1}H-NMR spectrum of **B11**......16

HRMS and ^{1}H-NMR spectrum of **B12**......17

HRMS and ^{1}H-NMR spectrum of **B13**......18

HRMS and ^{1}H-NMR spectrum of **B14**......19

HRMS and ^{1}H-NMR spectrum of **B15**......20

HRMS and ^{1}H-NMR spectrum of **B16**......21

HRMS and ^{1}H-NMR spectrum of **B17**......22

HRMS and ^{1}H-NMR spectrum of **C1**......23

HRMS and ^{1}H-NMR spectrum of **C2**......24

HRMS and ^{1}H-NMR spectrum of **C3**......25

HRMS and ^{1}H-NMR spectrum of **C4**......26

HRMS and ^{1}H-NMR spectrum of **C5**......27

HRMS and ^{1}H-NMR spectrum of **C6**......28

HRMS and ^{1}H-NMR spectrum of **C7**..29

HRMS and ^{1}H-NMR spectrum of **C8**..30

HRMS and ^{1}H-NMR spectrum of **C9**..31

HRMS and ^{1}H-NMR spectrum of **C10**..32

HRMS and ^{1}H-NMR spectrum of **C11**..33

HRMS and ^{1}H-NMR spectrum of **C12**..34

HRMS and ^{1}H-NMR spectrum of **C13**..35

HRMS and ^{1}H-NMR spectrum of **C14**..36

HRMS and ^{1}H-NMR spectrum of **C15**..37

HRMS and ^{1}H-NMR spectrum of **C16**..38

HRMS and ^{1}H-NMR spectrum of **C17**..39

HRMS and ^{1}H-NMR spectrum of **C18**..40

HRMS and ^{1}H-NMR spectrum of **C19**..41

HRMS and ^{1}H-NMR spectrum of **C20**..42

HRMS and ^{1}H-NMR spectrum of **C21**..43

HRMS and ^{1}H-NMR spectrum of **C22**..44

HRMS and ^{1}H-NMR spectrum of **C23**..45

HRMS and ^{1}H-NMR spectrum of **C24**..46

HRMS and ^{1}H-NMR spectrum of **C25**..47

HRMS and ^{1}H-NMR spectrum of **C26**..48

HRMS and ^{1}H-NMR spectrum of **C27**..49

HRMS and ^{1}H-NMR spectrum of **C28**..50

HRMS and ^{1}H-NMR spectrum of **C29**..51

HRMS and ^{1}H-NMR spectrum of **D1**..52

^{13}C-NMR and HPLC spectrum of **D1**........53

HRMS and ^{1}H-NMR spectrum of **D2**........54

^{13}C-NMR and HPLC spectrum of **D2**........55

HRMS and ^{1}H-NMR spectrum of **D3**........56

^{13}C-NMR and HPLC spectrum of **D3**........57

HRMS and ^{1}H-NMR spectrum of **D4**........58

^{13}C-NMR and HPLC spectrum of **D4**........59

HRMS and ^{1}H-NMR spectrum of **D5**........60

^{13}C-NMR and HPLC spectrum of **D5**........61

HRMS and ^{1}H-NMR spectrum of **D6**........62

^{13}C-NMR and HPLC spectrum of **D6**........63

HRMS and ^{1}H-NMR spectrum of **D7**........64

^{13}C-NMR and HPLC spectrum of **D7**........65

HRMS and ^{1}H-NMR spectrum of **D8**........66

^{13}C-NMR and HPLC spectrum of **D8**........67

HRMS and ^{1}H-NMR spectrum of **D9**........68

^{13}C-NMR and HPLC spectrum of **D9**........69

HRMS and ^{1}H-NMR spectrum of **D10**........70

^{13}C-NMR and HPLC spectrum of **D10**........71

HRMS and ^{1}H-NMR spectrum of **D11**........72

^{13}C-NMR and HPLC spectrum of **D11**........73

HRMS and ^{1}H-NMR spectrum of **D12**........74

^{13}C-NMR and HPLC spectrum of **D12**........75

HRMS and ^{1}H-NMR spectrum of **D13**........76

^{13}C-NMR and HPLC spectrum of **D13**..77

HRMS and ^{1}H-NMR spectrum of **D14**..78

^{13}C-NMR and HPLC spectrum of **D14**..79

HRMS and ^{1}H-NMR spectrum of **D15**..80

^{13}C-NMR and HPLC spectrum of **D15**..81

HRMS and ^{1}H-NMR spectrum of **D16**..82

^{13}C-NMR and HPLC spectrum of **D16**..83

HRMS and ^{1}H-NMR spectrum of **D17**..84

^{13}C-NMR and HPLC spectrum of **D17**..85

HRMS and ^{1}H-NMR spectrum of **D18**..86

^{13}C-NMR and HPLC spectrum of **D18**..87

HRMS and ^{1}H-NMR spectrum of **D19**..88

^{13}C-NMR and HPLC spectrum of **D19**..89

HRMS and ^{1}H-NMR spectrum of **D20**..90

^{13}C-NMR and HPLC spectrum of **D20**..91

HRMS and ^{1}H-NMR spectrum of **D21**..92

^{13}C-NMR and HPLC spectrum of **D21**..93

HRMS and ^{1}H-NMR spectrum of **D22**..94

^{13}C-NMR and HPLC spectrum of **D22**..95

HRMS and ^{1}H-NMR spectrum of **D23**..96

^{13}C-NMR and HPLC spectrum of **D23**..97

HRMS and ^{1}H-NMR spectrum of **D24**..98

^{13}C-NMR and HPLC spectrum of **D24**..99

HRMS and ^{1}H-NMR spectrum of **D25**..100

^{13}C-NMR and HPLC spectrum of **D25**......101

HRMS and ^{1}H-NMR spectrum of **D26**......102

^{13}C-NMR and HPLC spectrum of **D26**......103

HRMS and ^{1}H-NMR spectrum of **D27**......104

^{13}C-NMR and HPLC spectrum of **D27**......105

HRMS and ^{1}H-NMR spectrum of **D28**......106

^{13}C-NMR and HPLC spectrum of **D28**......107

HRMS and ^{1}H-NMR spectrum of **D29**......108

^{13}C-NMR and HPLC spectrum of **D29**......109

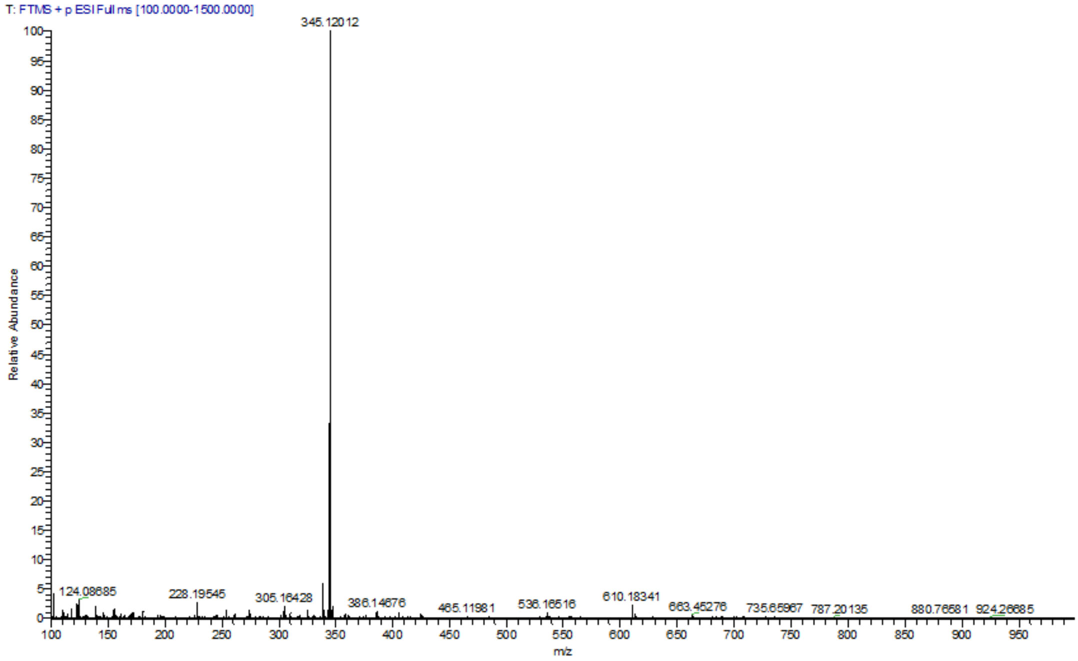

HRMS spectrum of **B1**

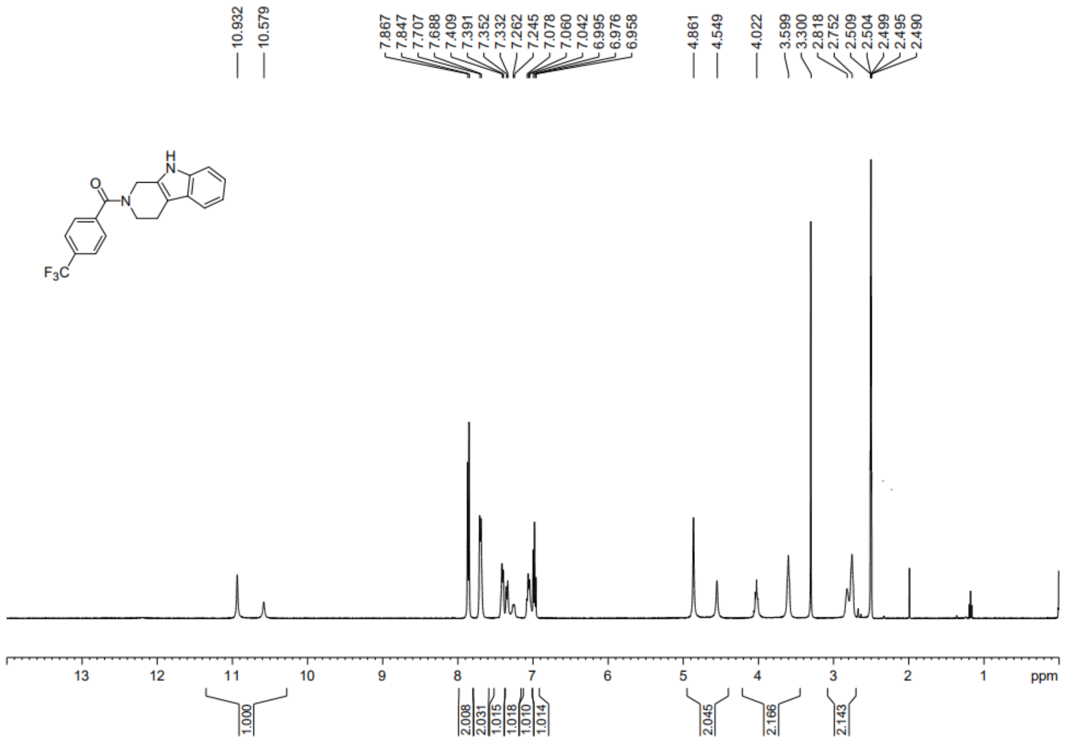

^{1}H NMR spectrum of **B1**

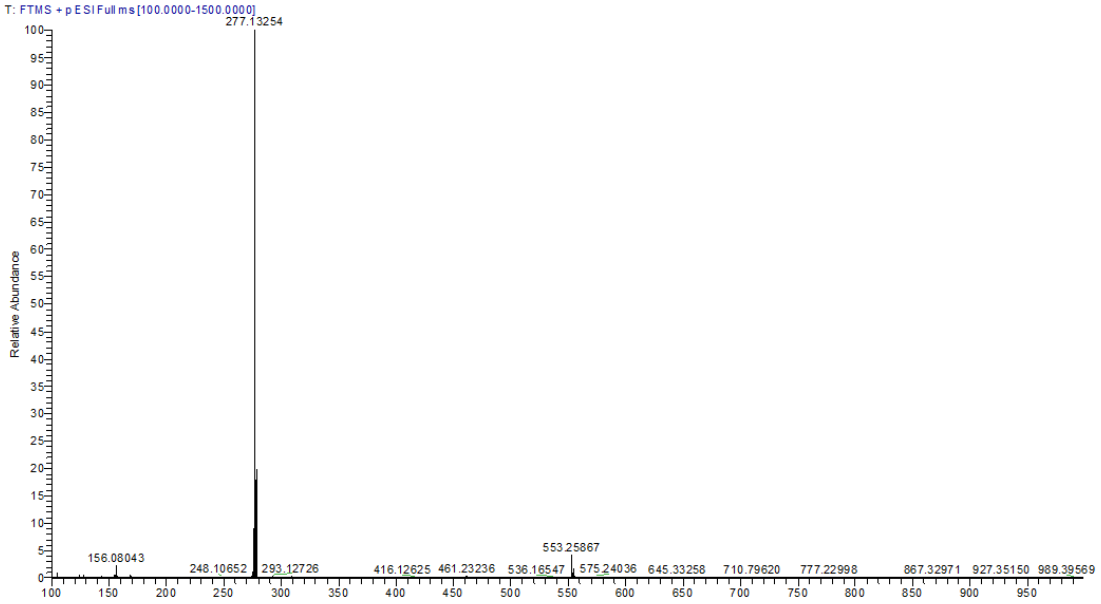

HRMS spectrum of **B2**

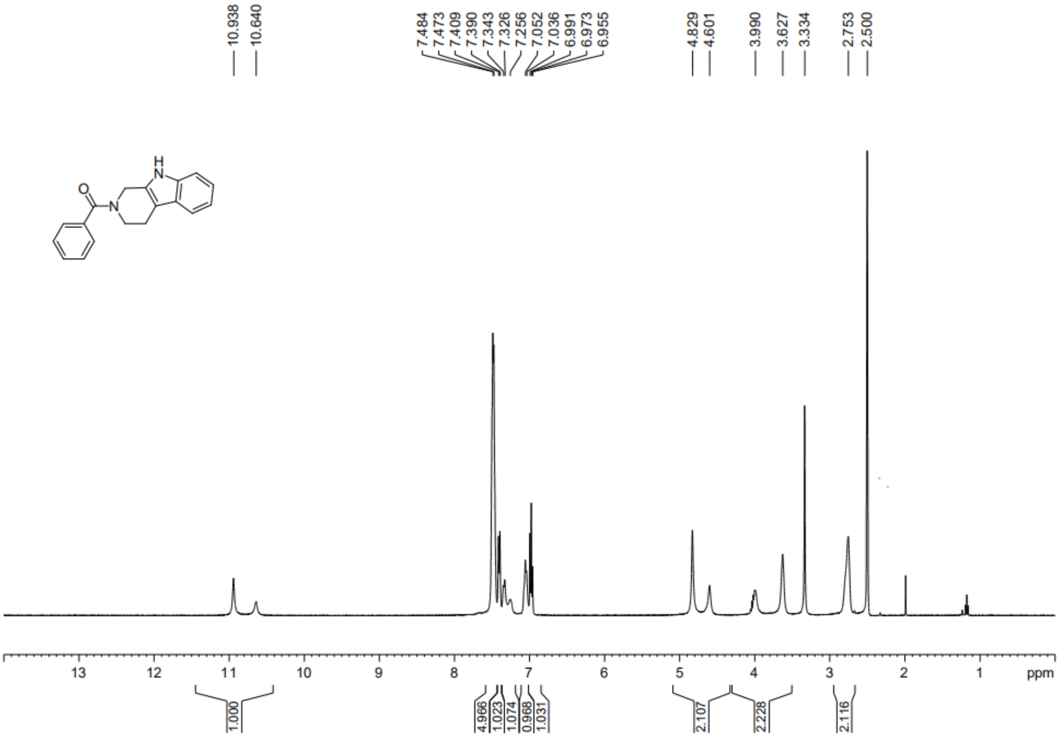

¹H NMR spectrum of **B2**

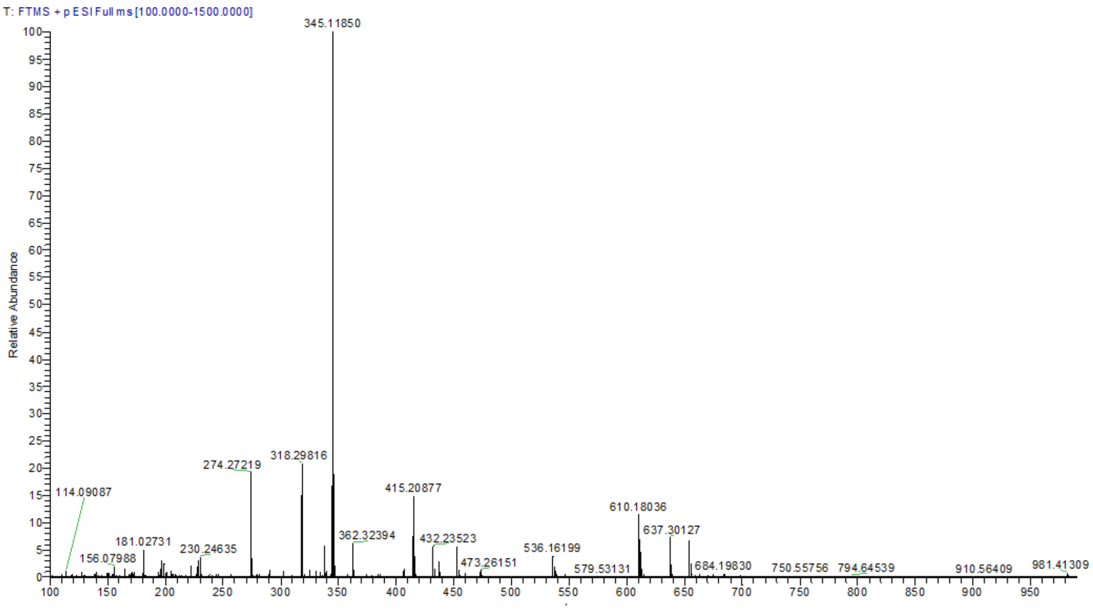

HRMS spectrum of **B3**

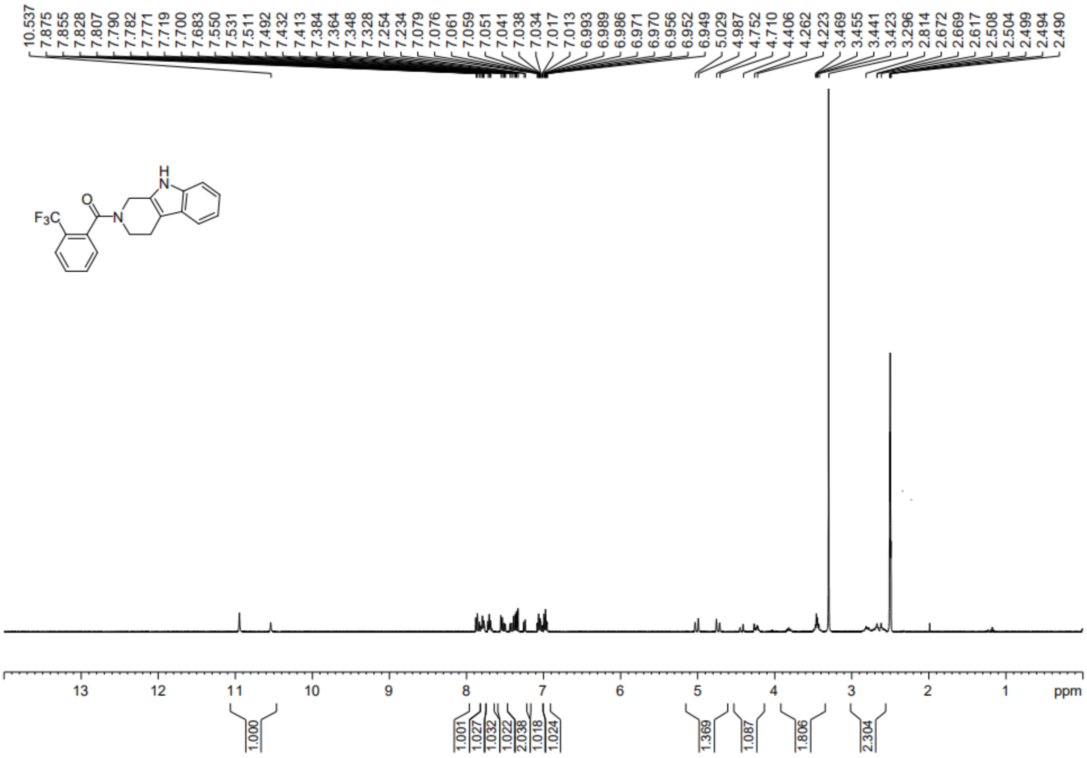

^{1}H NMR spectrum of **B3**

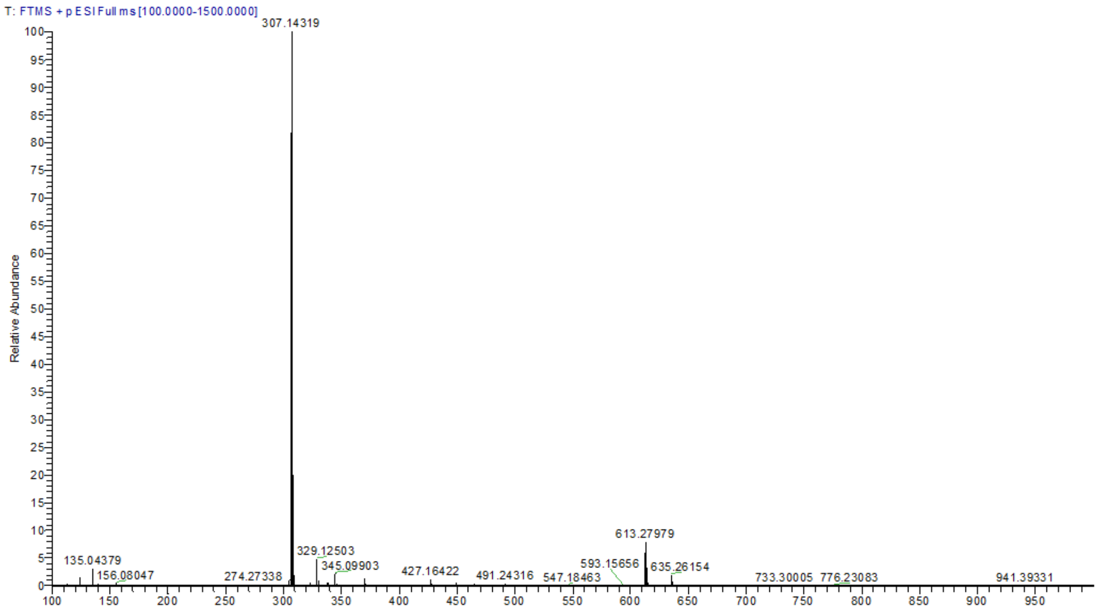

HRMS spectrum of **B4**

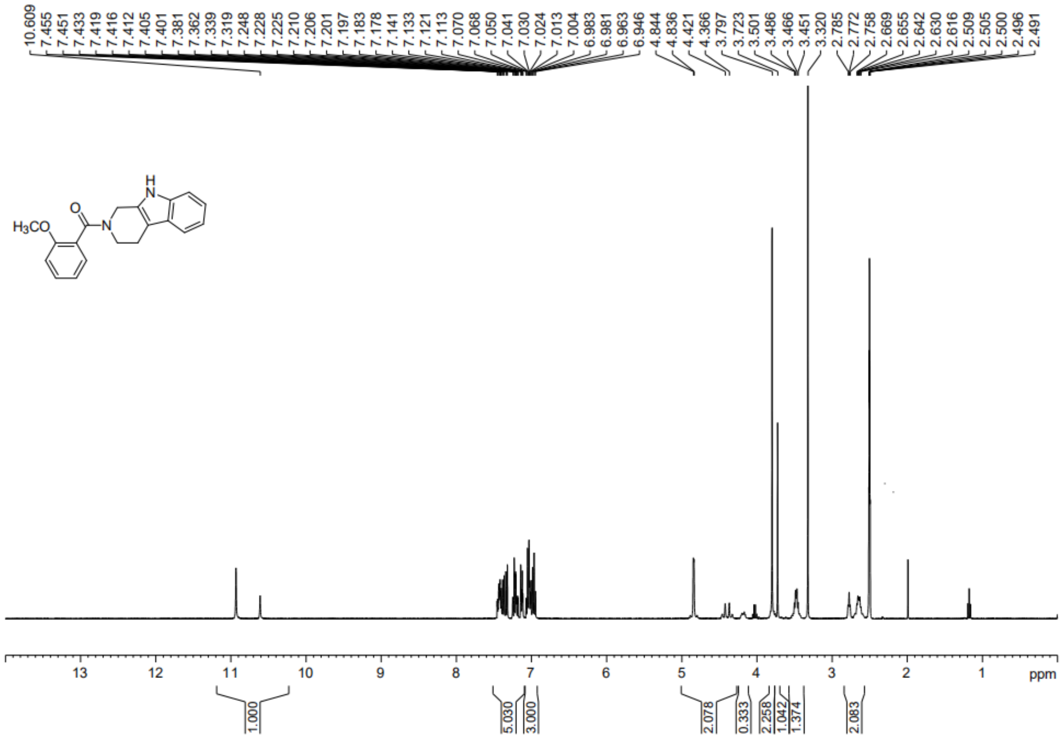

^{1}H NMR spectrum of **B4**

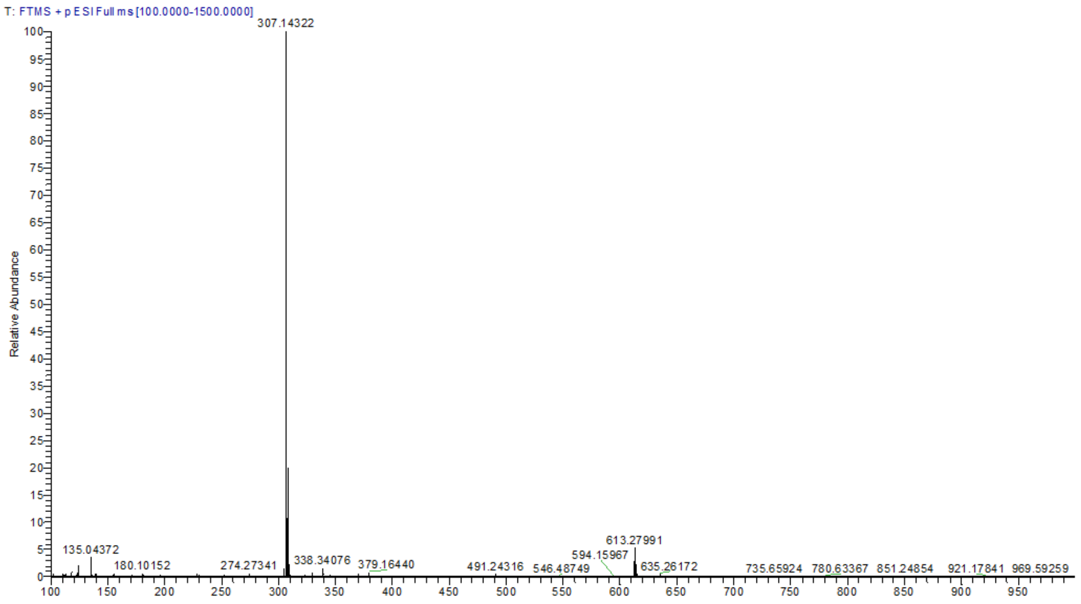

HRMS spectrum of **B5**

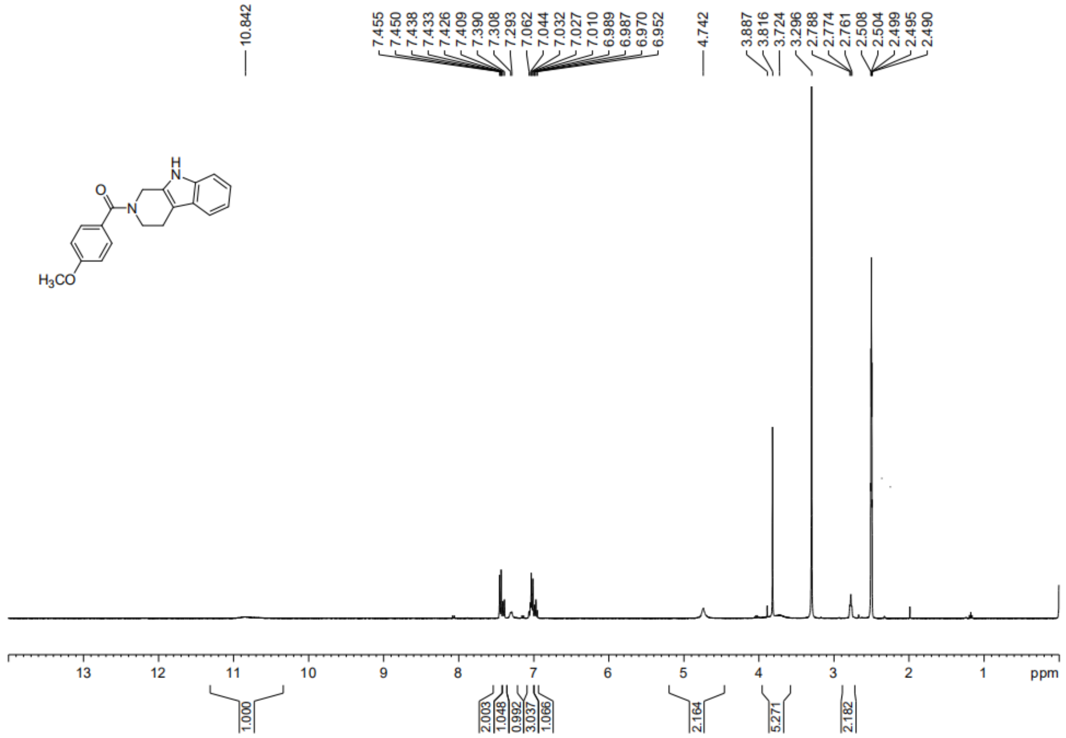

^{1}H NMR spectrum of **B5**

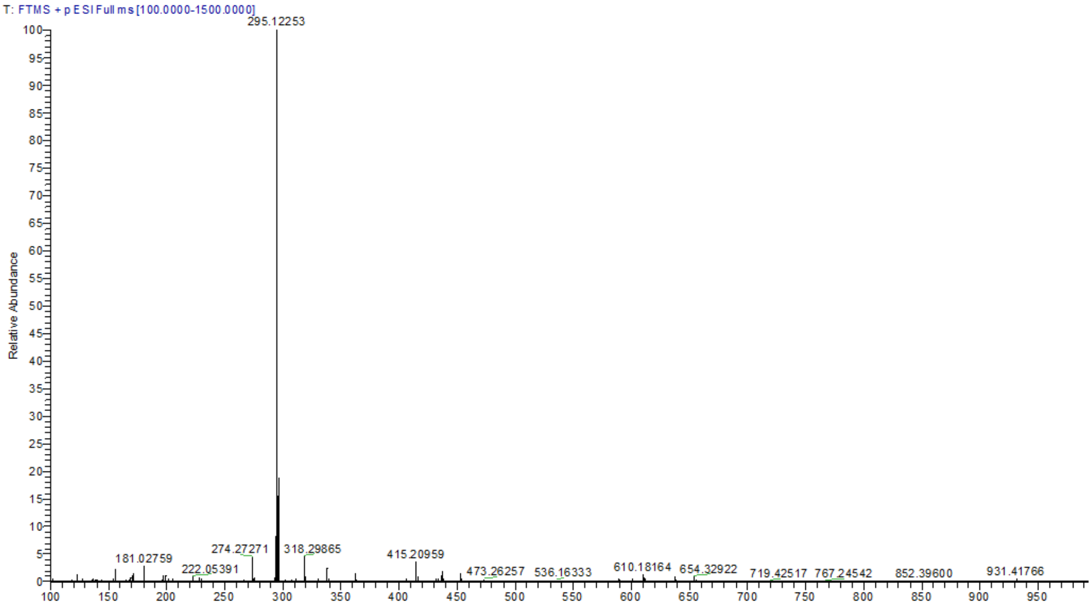

HRMS spectrum of **B6**

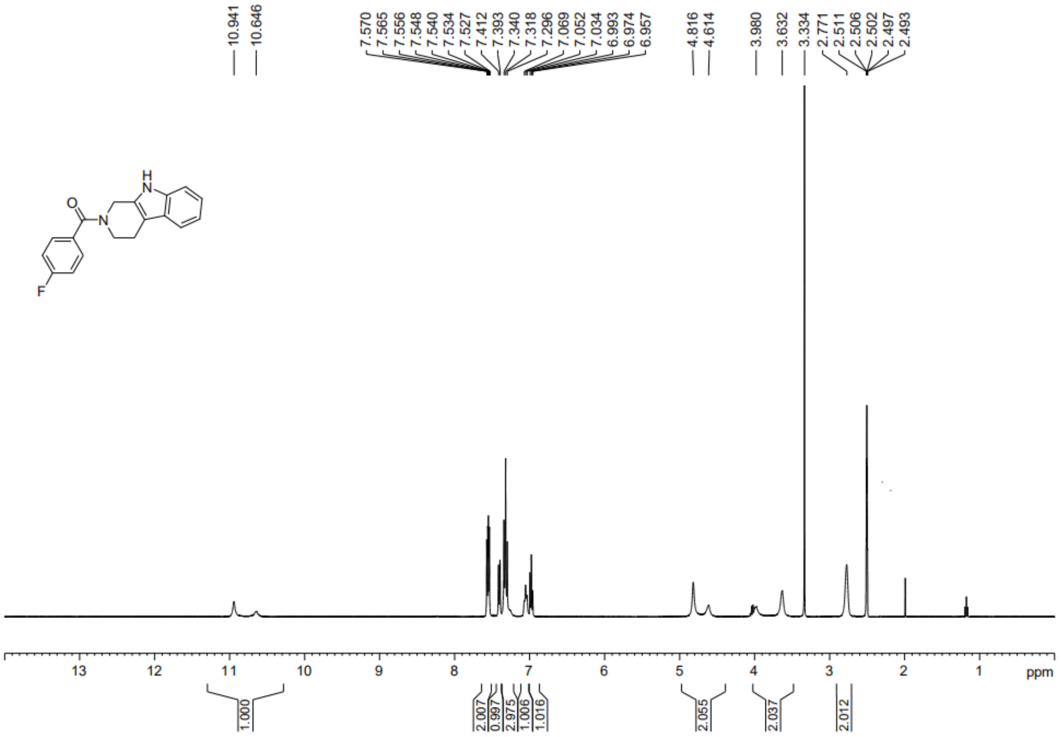

^{1}H NMR spectrum of **B6**

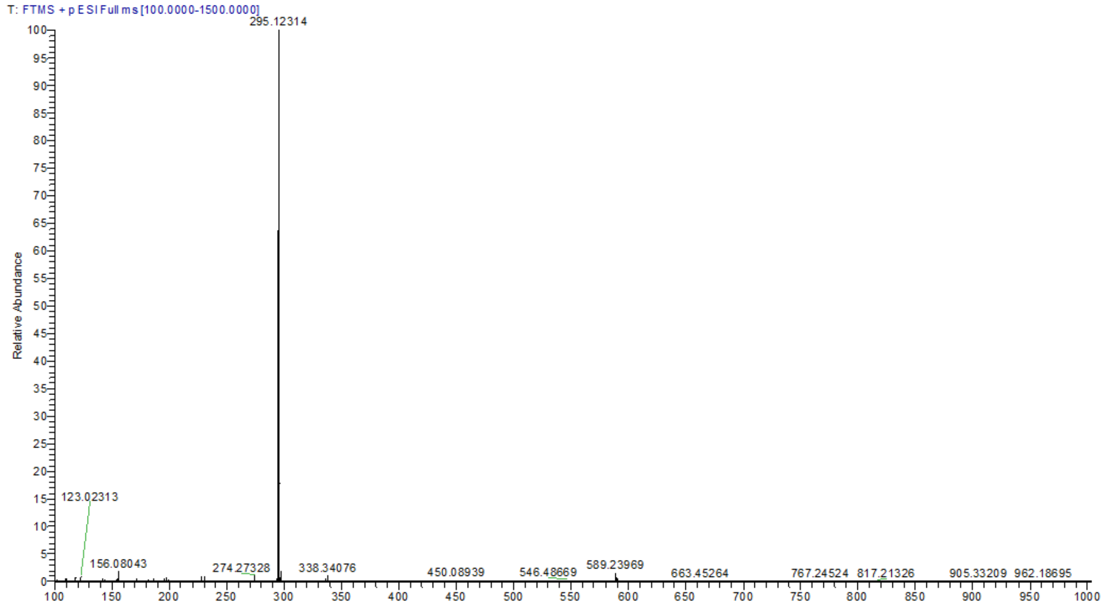

HRMS spectrum of **B7**

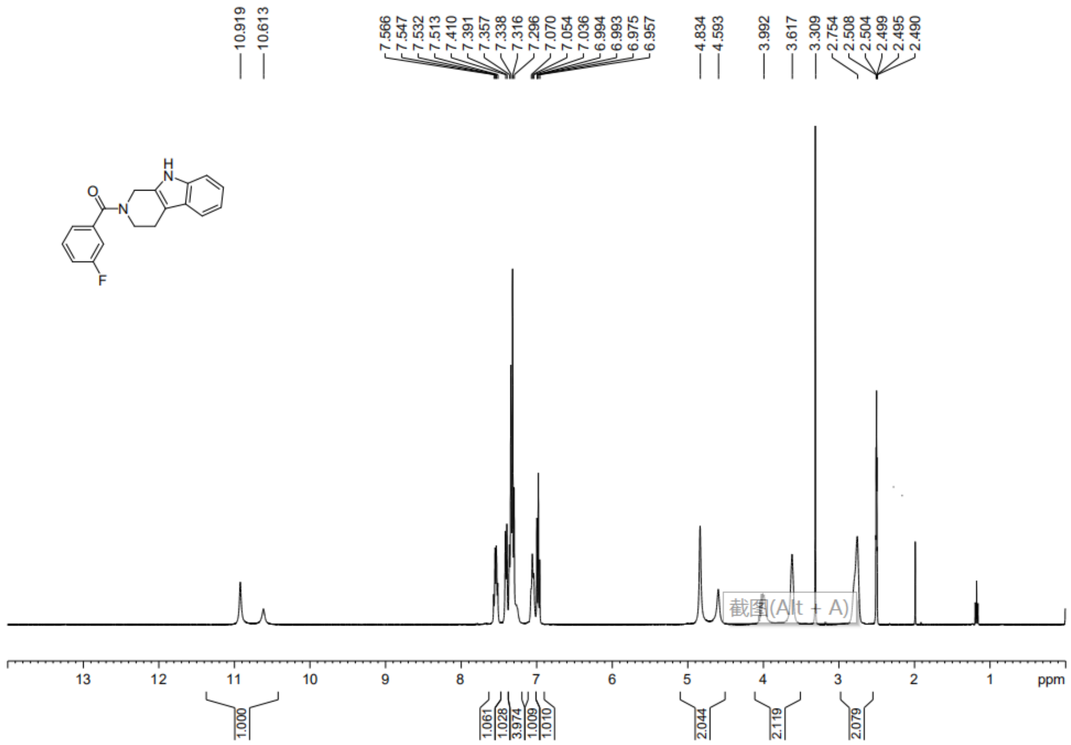

^{1}H NMR spectrum of **B7**

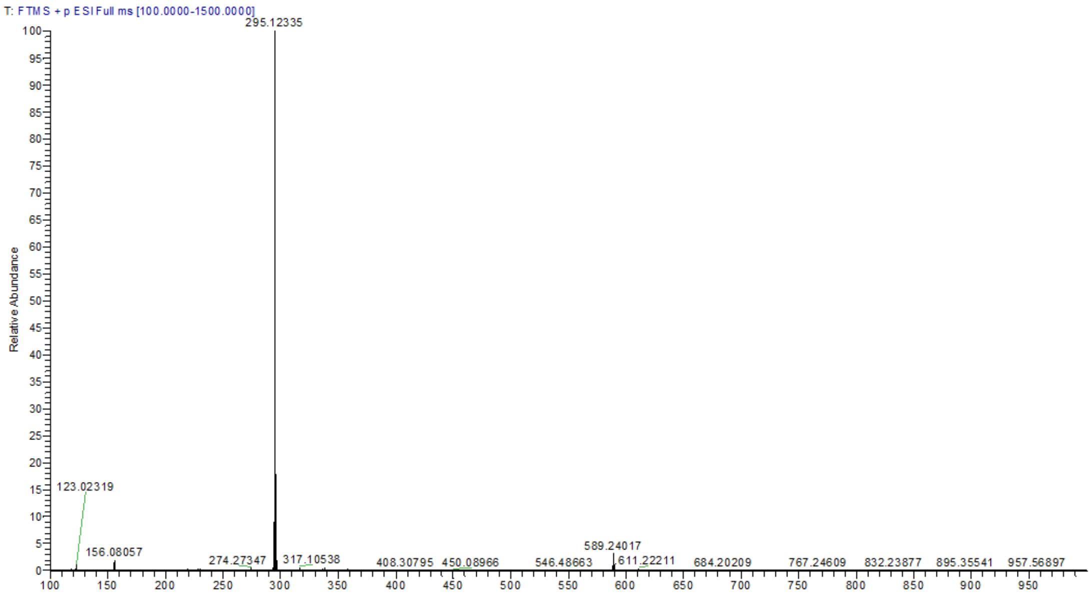

HRMS spectrum of **B8**

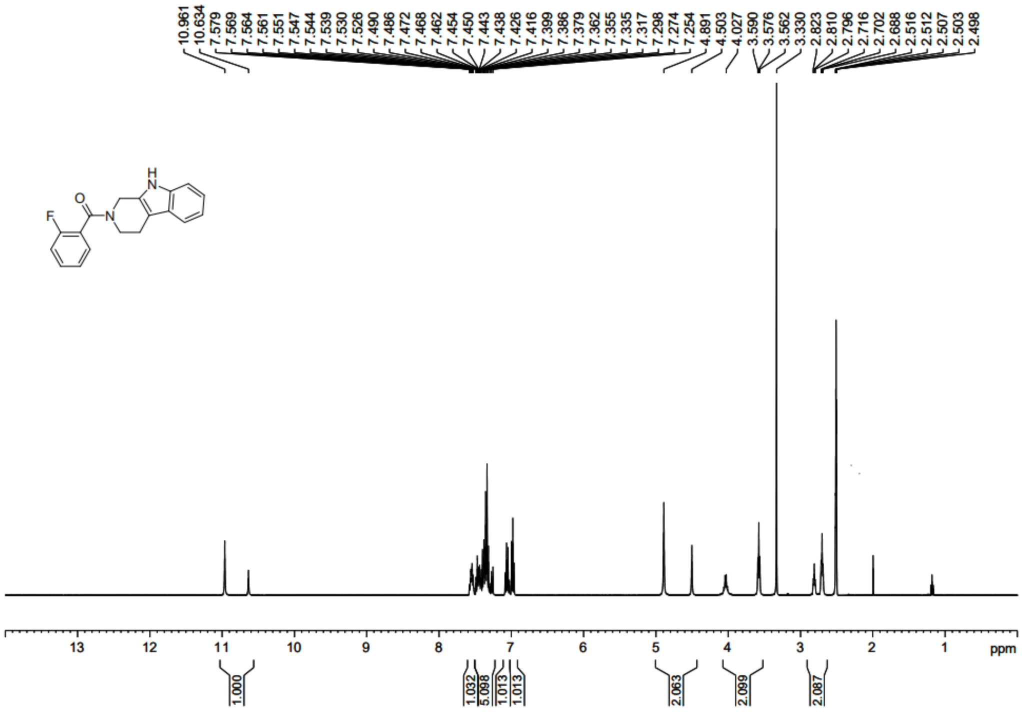

^{1}H NMR spectrum of **B8**

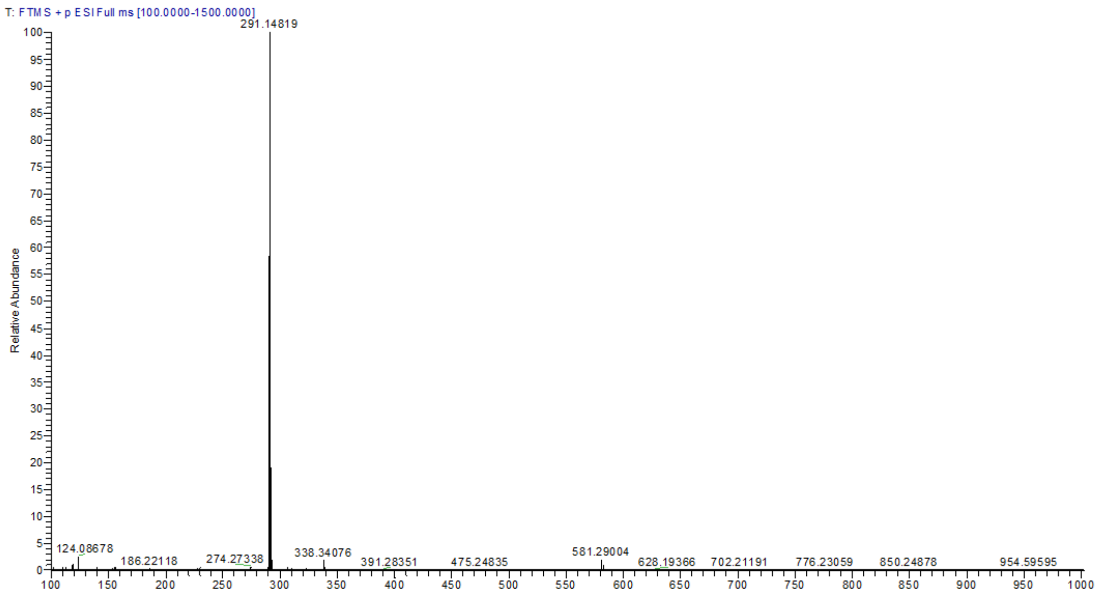

HRMS spectrum of **B9**

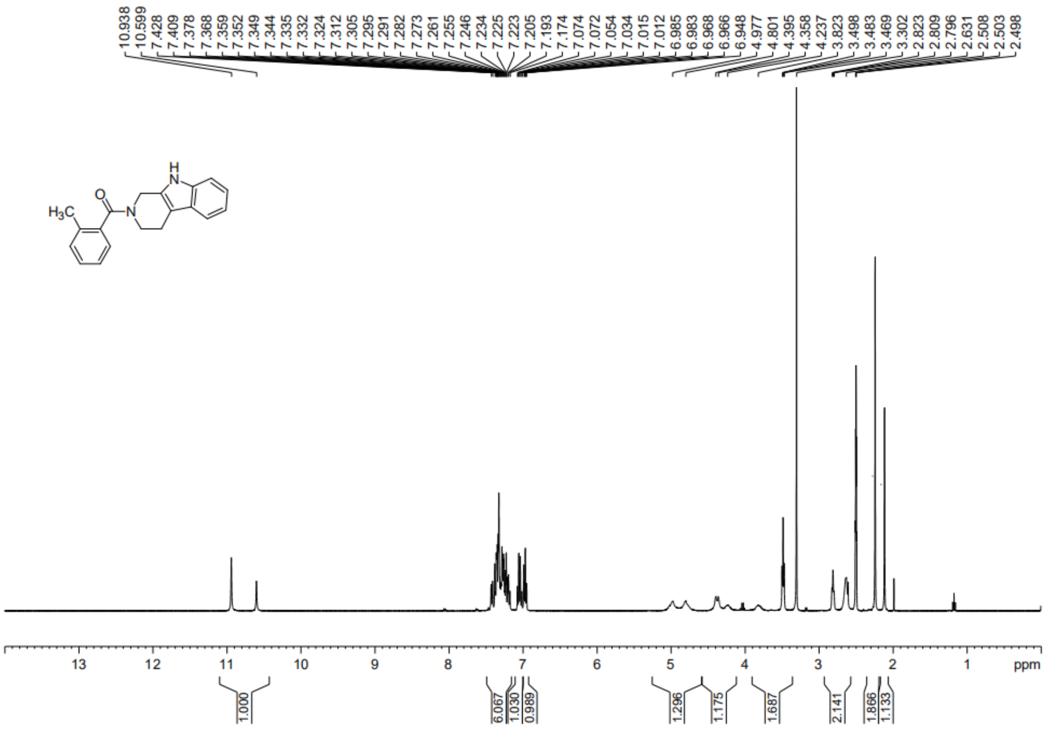

^{1}H NMR spectrum of **B9**

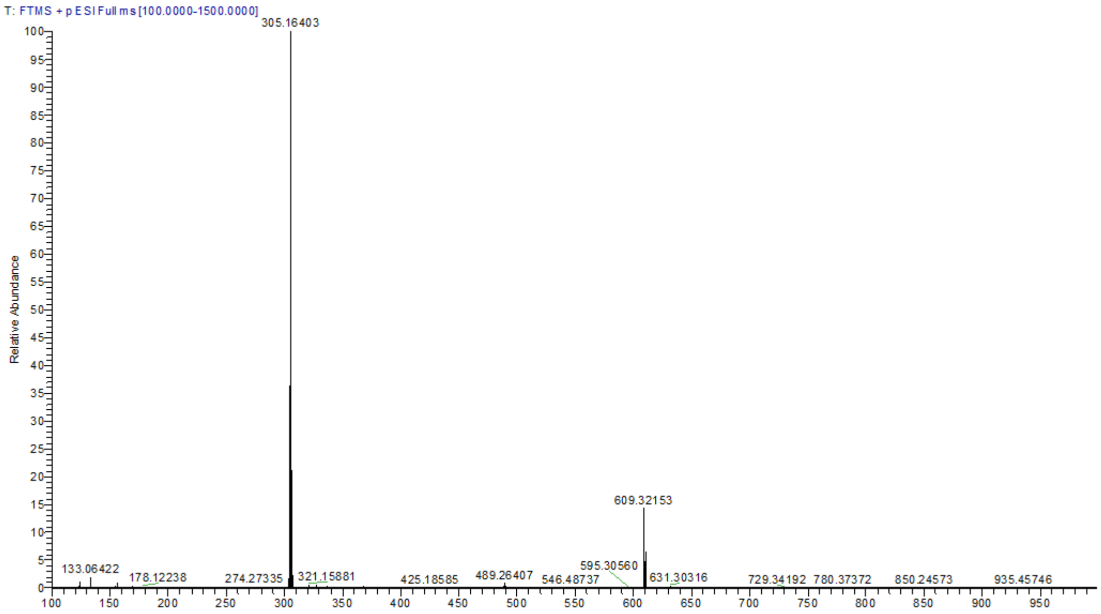

HRMS spectrum of **B10**

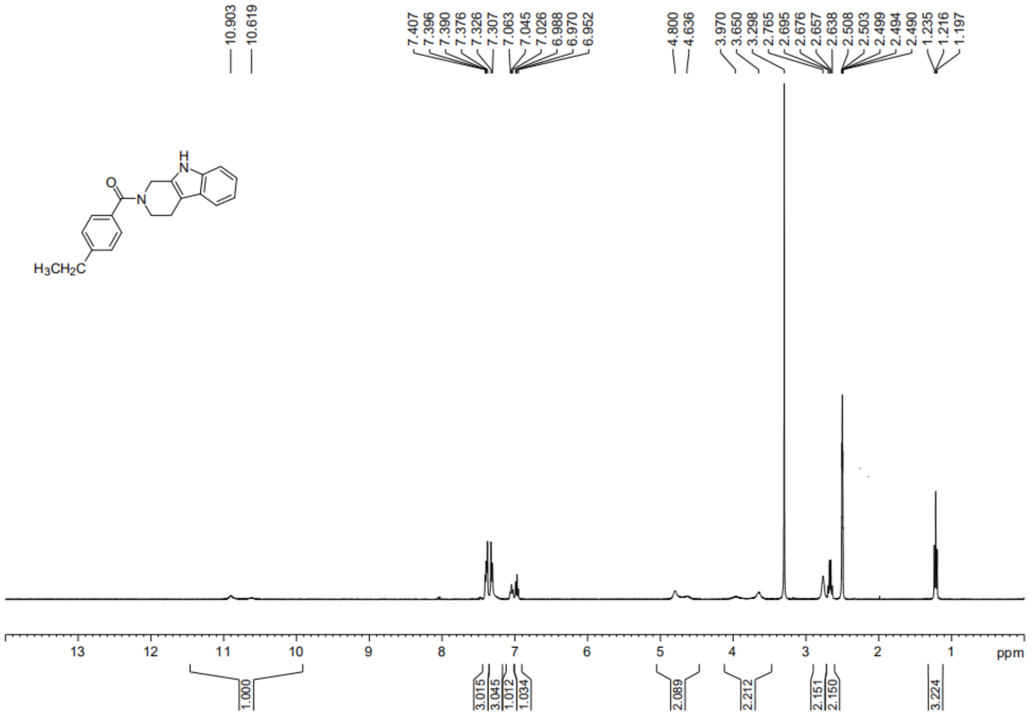

^{1}H NMR spectrum of **B10**

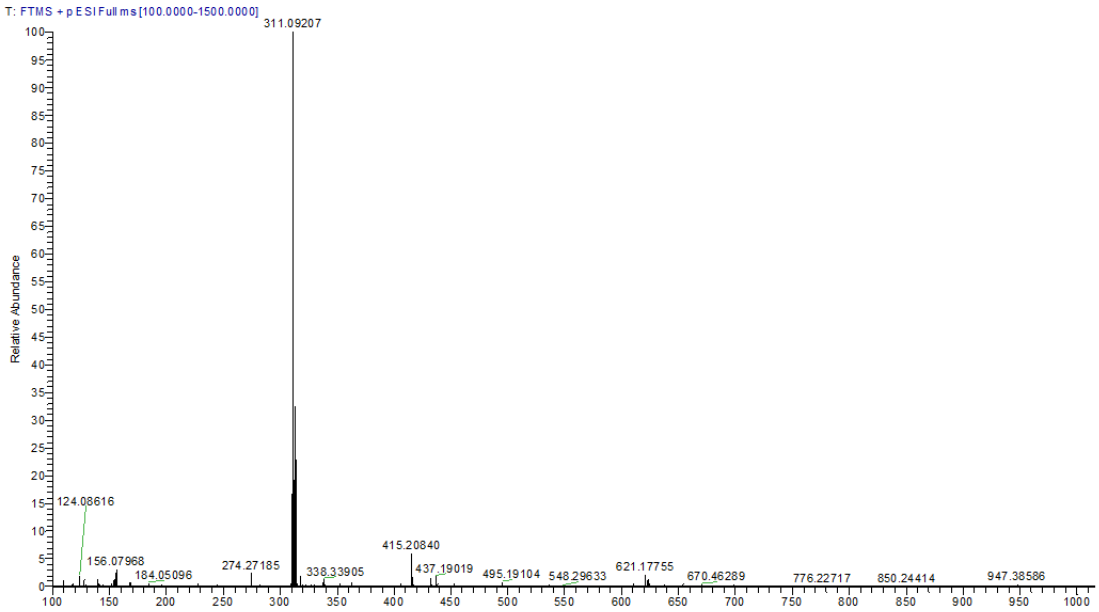

HRMS spectrum of **B11**

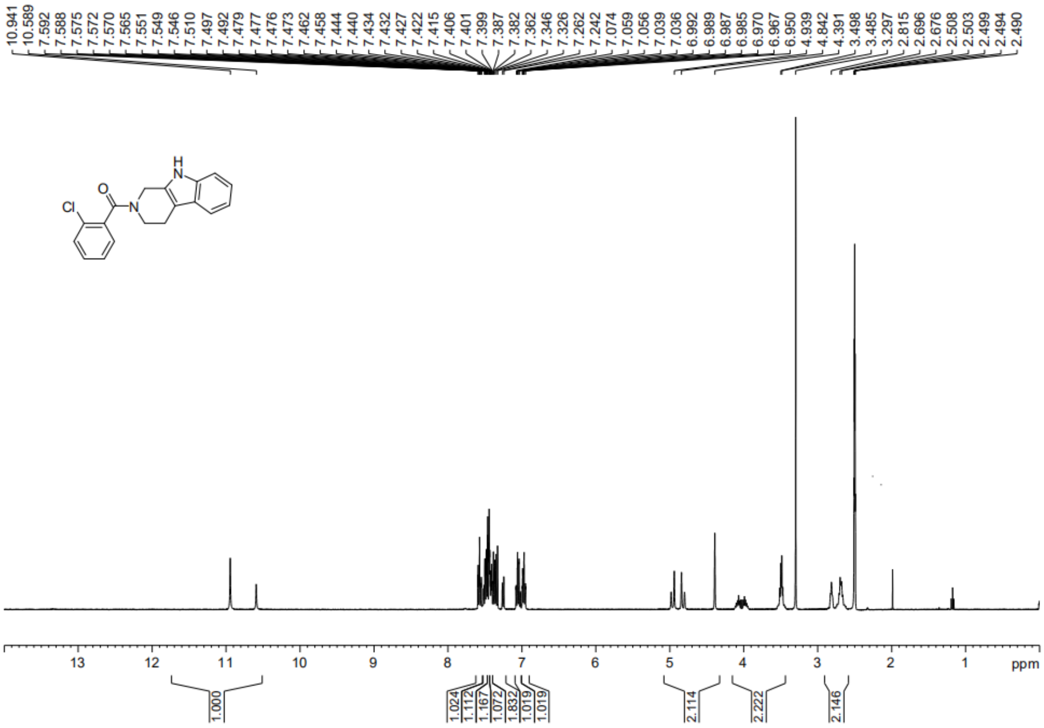

^{1}H NMR spectrum of **B11**

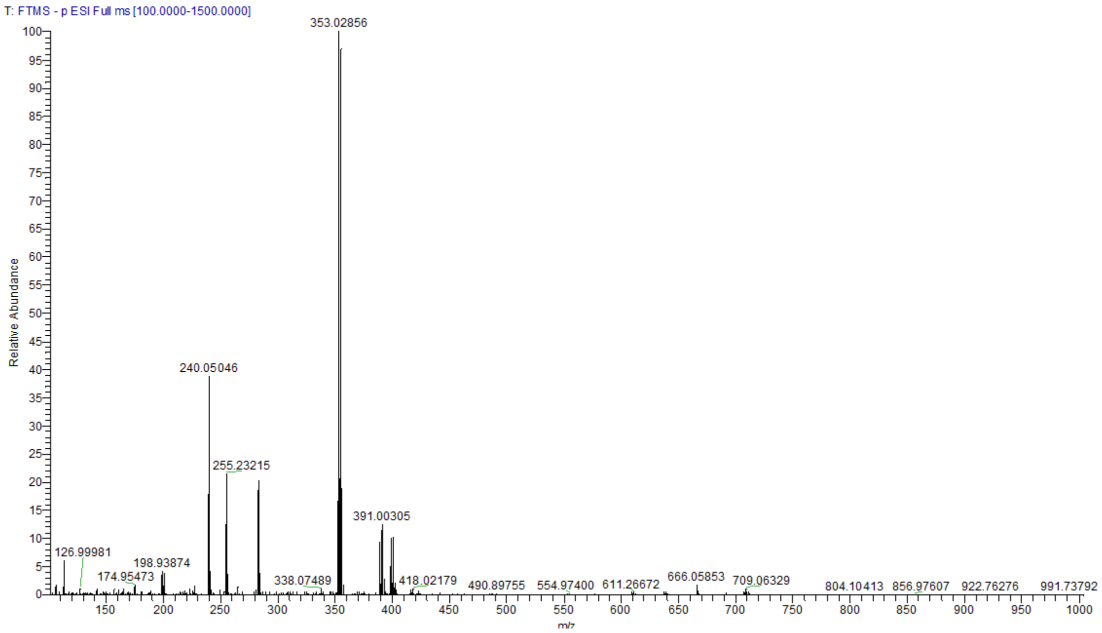

HRMS spectrum of **B12**

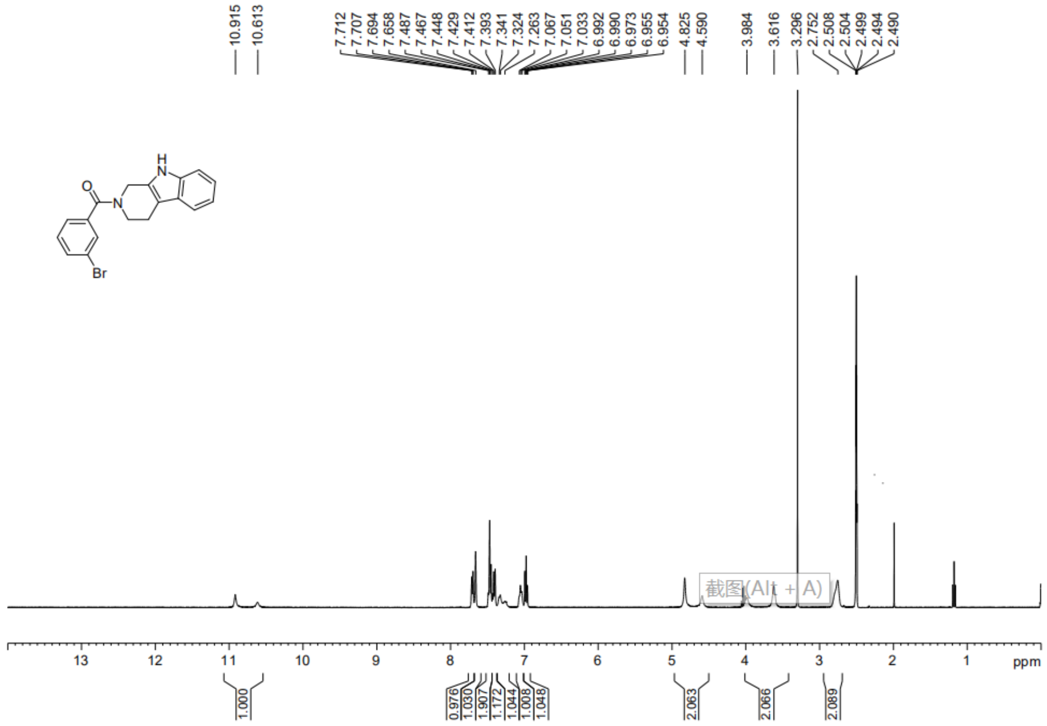

^{1}H NMR spectrum of **B12**

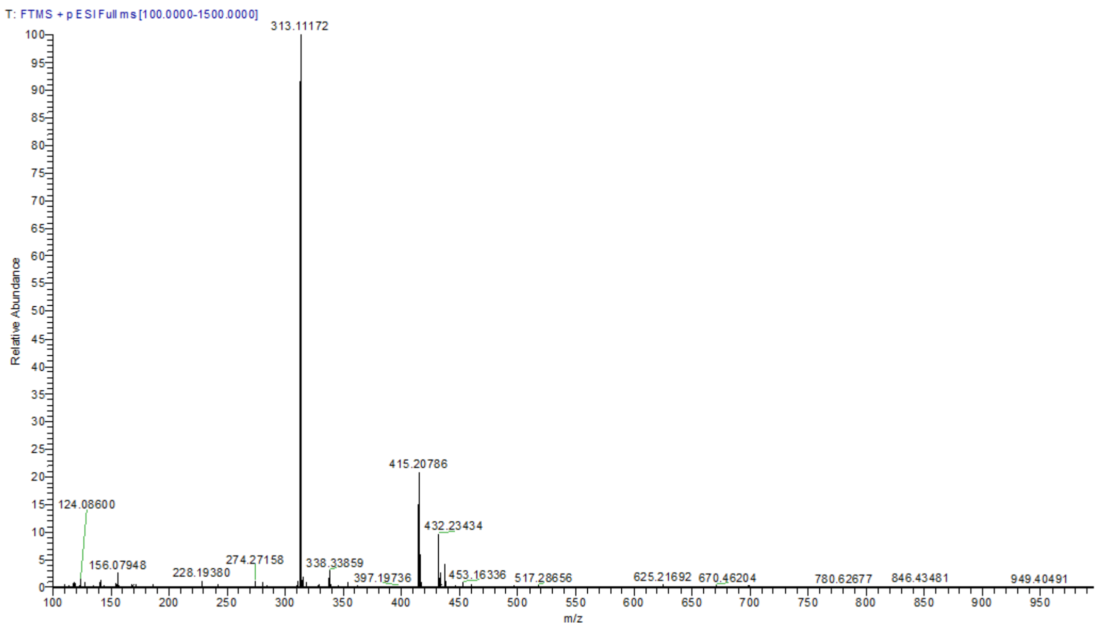

HRMS spectrum of **B13**

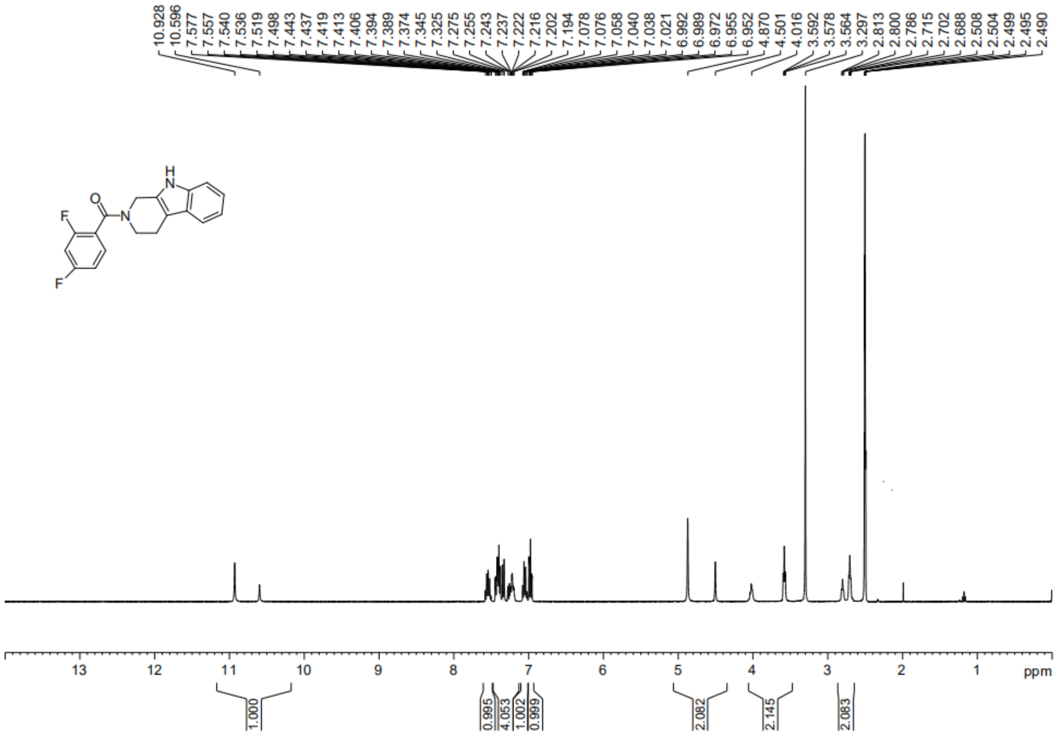

^{1}H NMR spectrum of **B13**

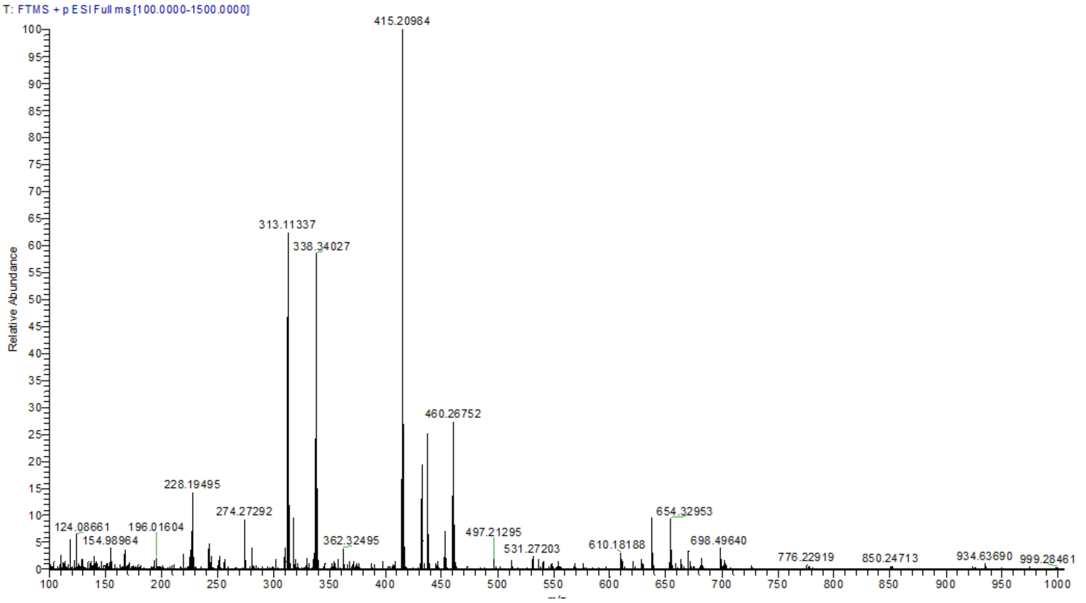

HRMS spectrum of **B14**

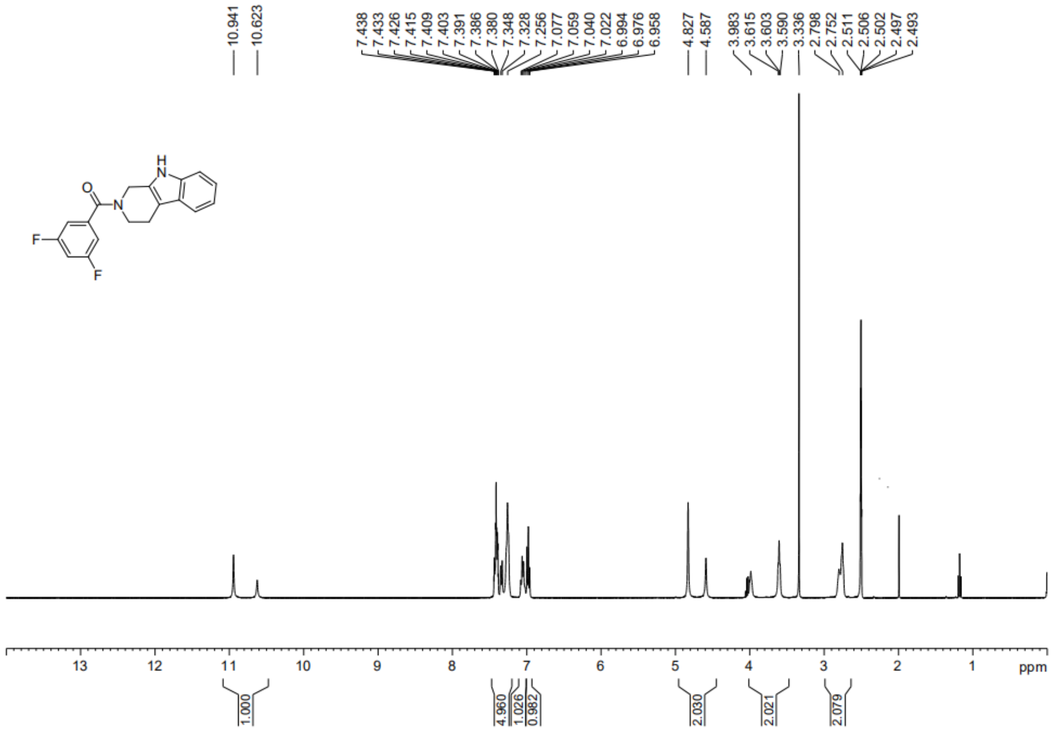

^{1}H NMR spectrum of **B14**

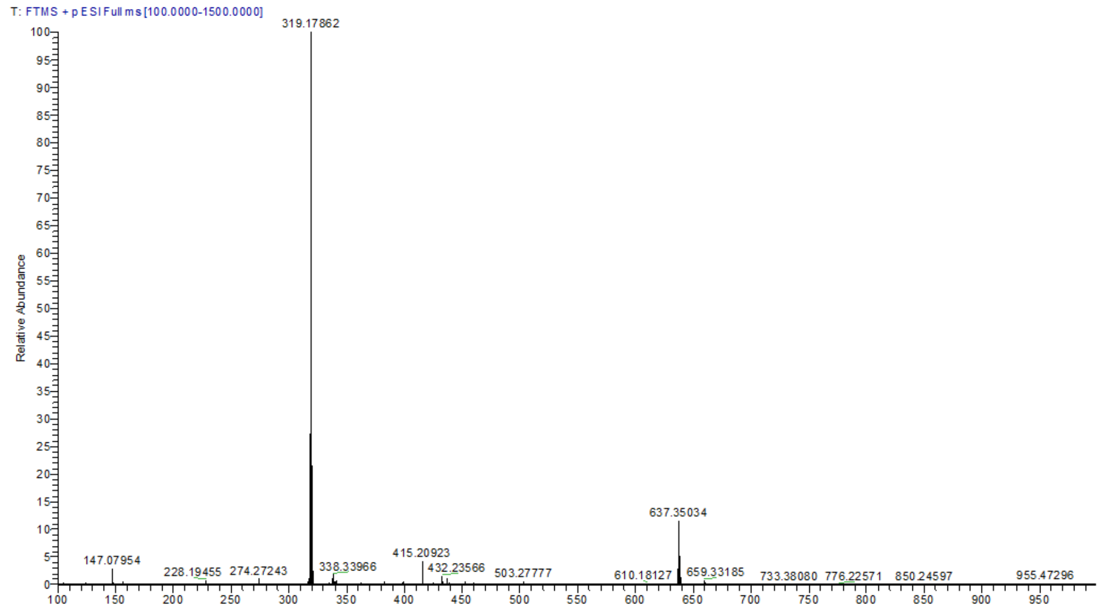

HRMS spectrum of **B15**

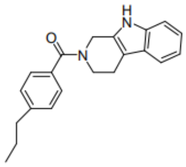

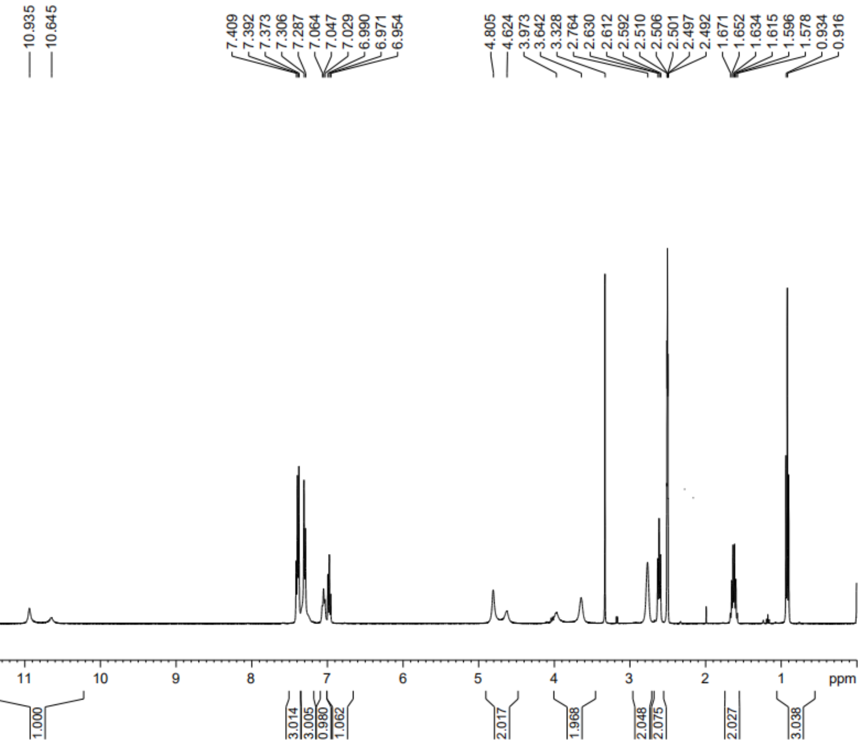

^{1}H NMR spectrum of **B15**

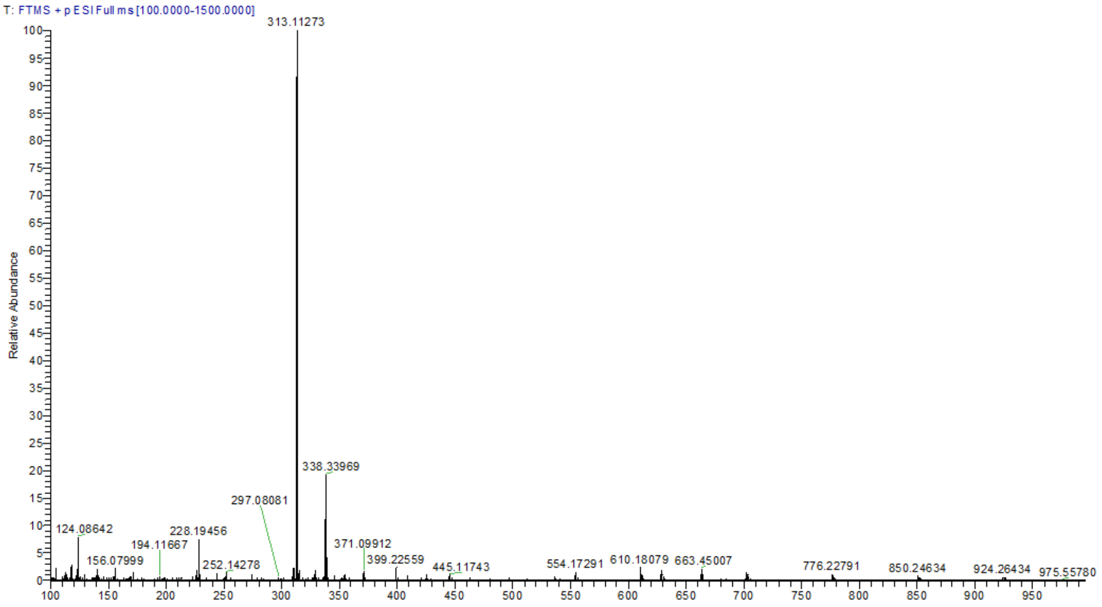

HRMS spectrum of **B16**

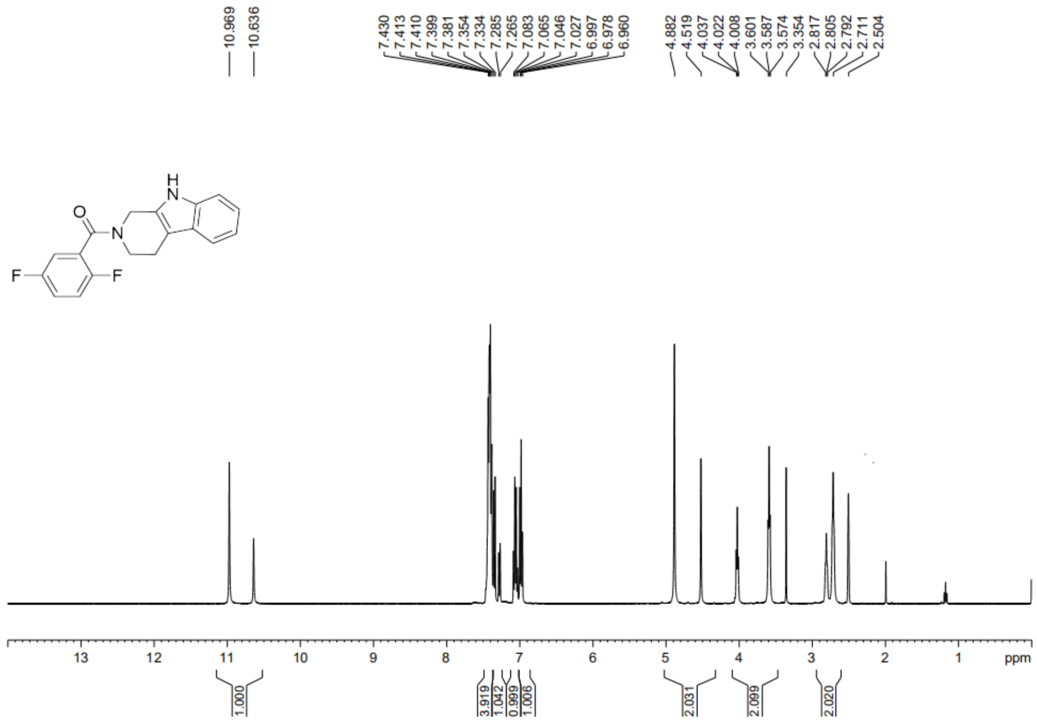

^{1}H NMR spectrum of **B16**

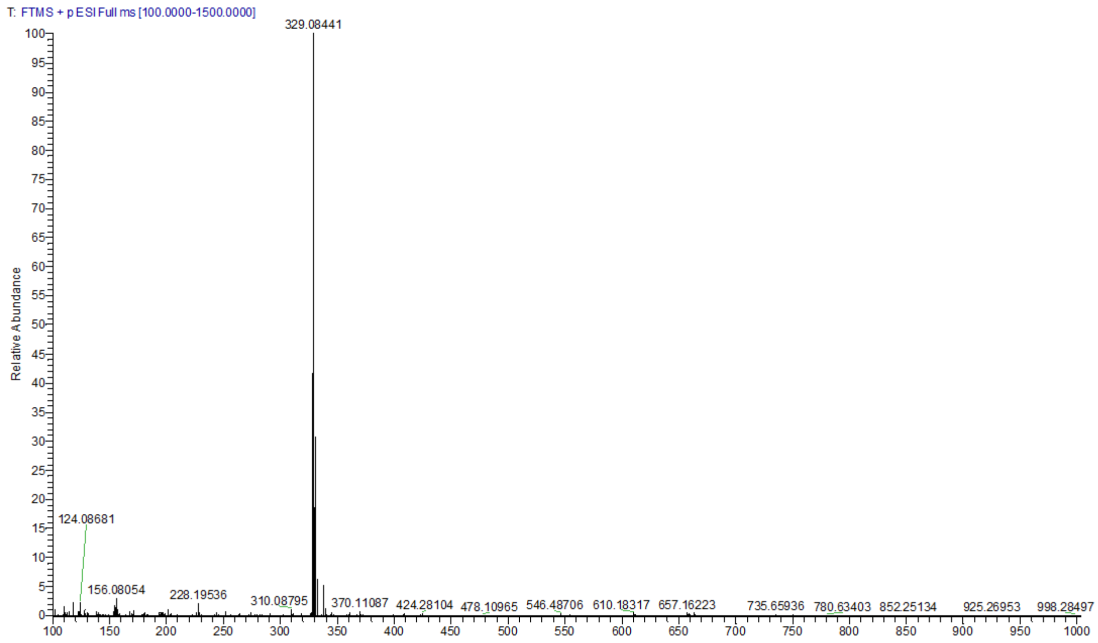

HRMS spectrum of **B17**

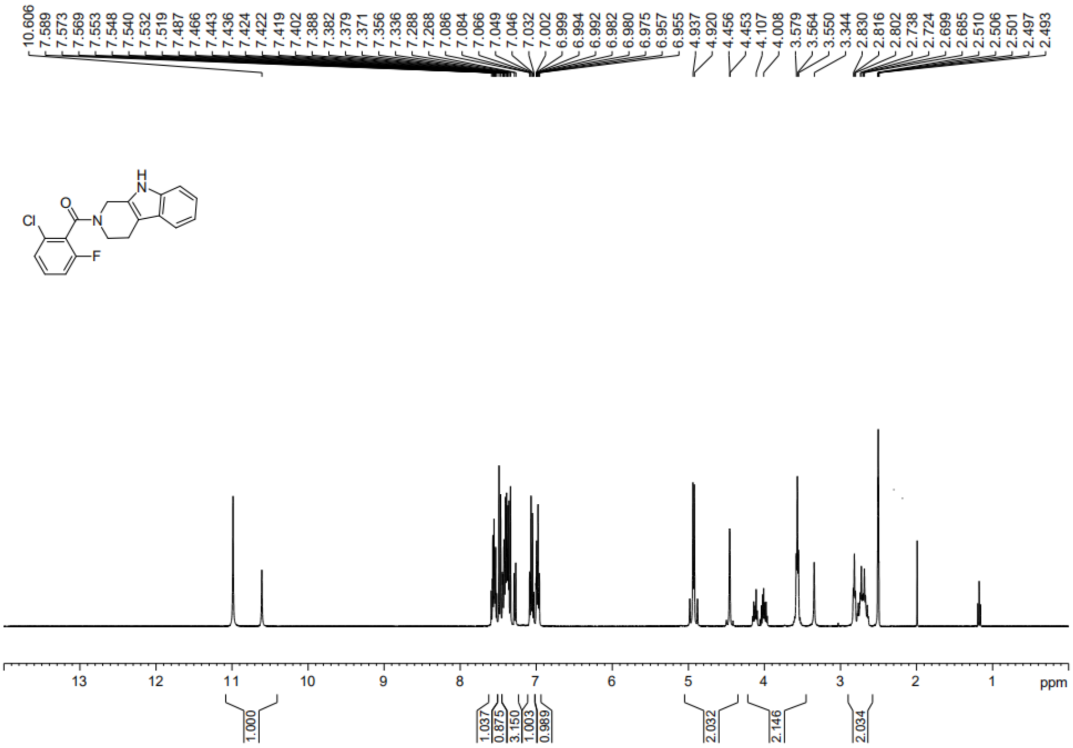

^{1}H NMR spectrum of **B17**

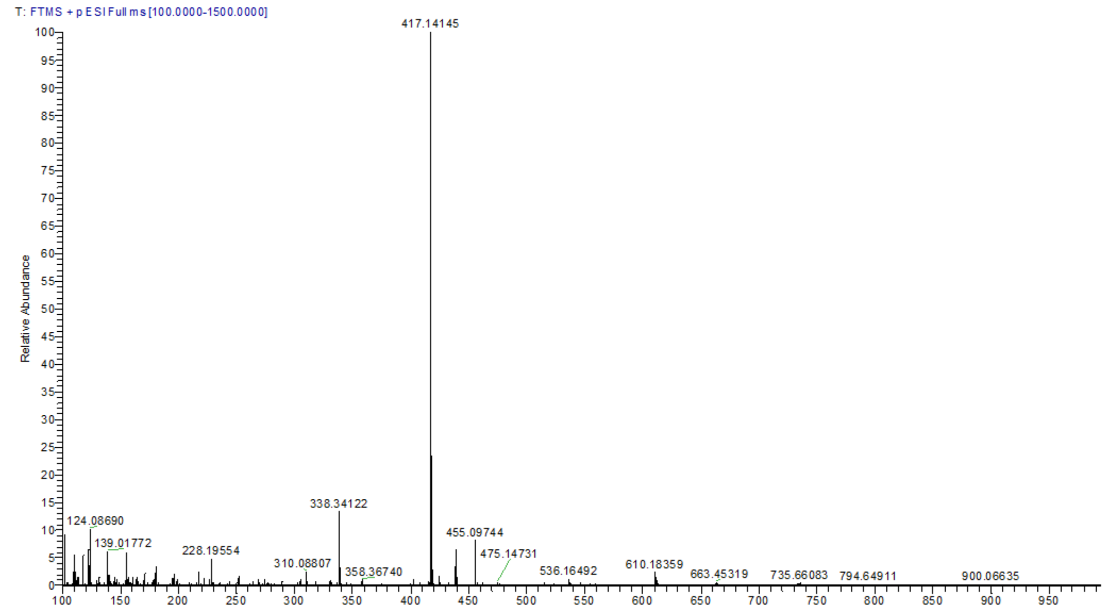

HRMS spectrum of **C1**

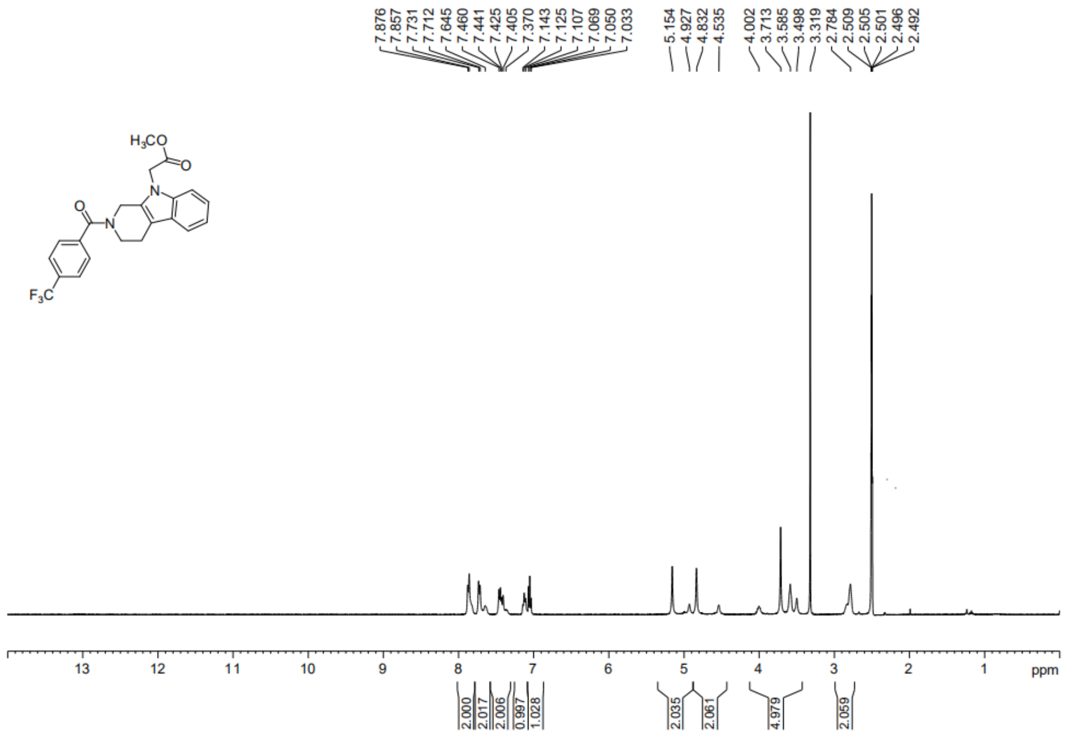

^{1}H NMR spectrum of **C1**

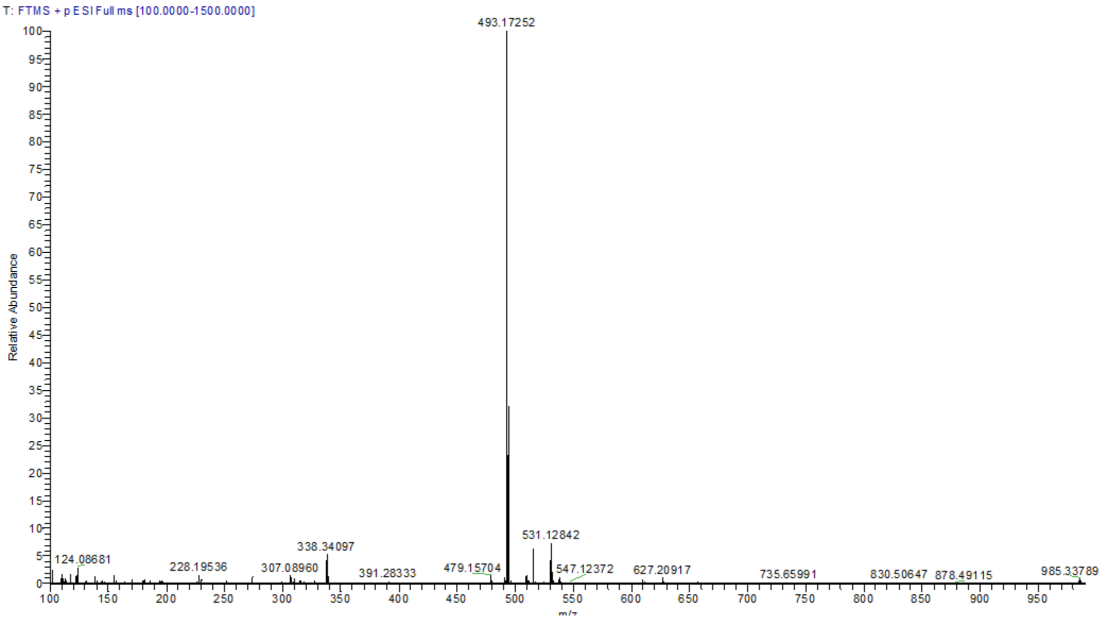

HRMS spectrum of **C2**

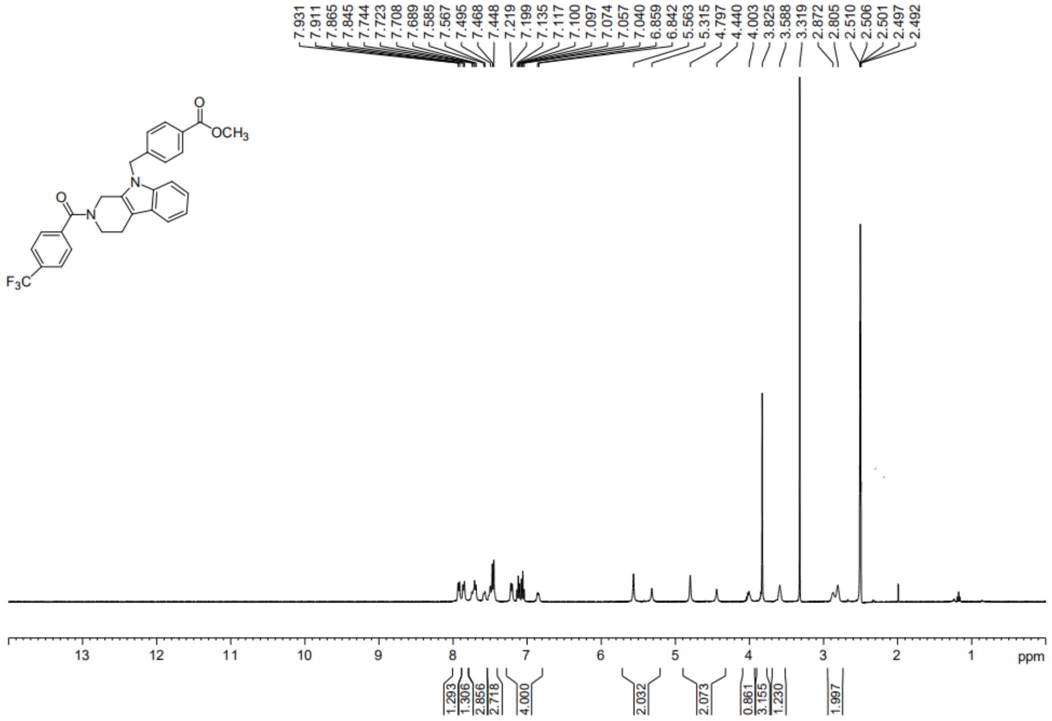

^{1}H NMR spectrum of **C2**

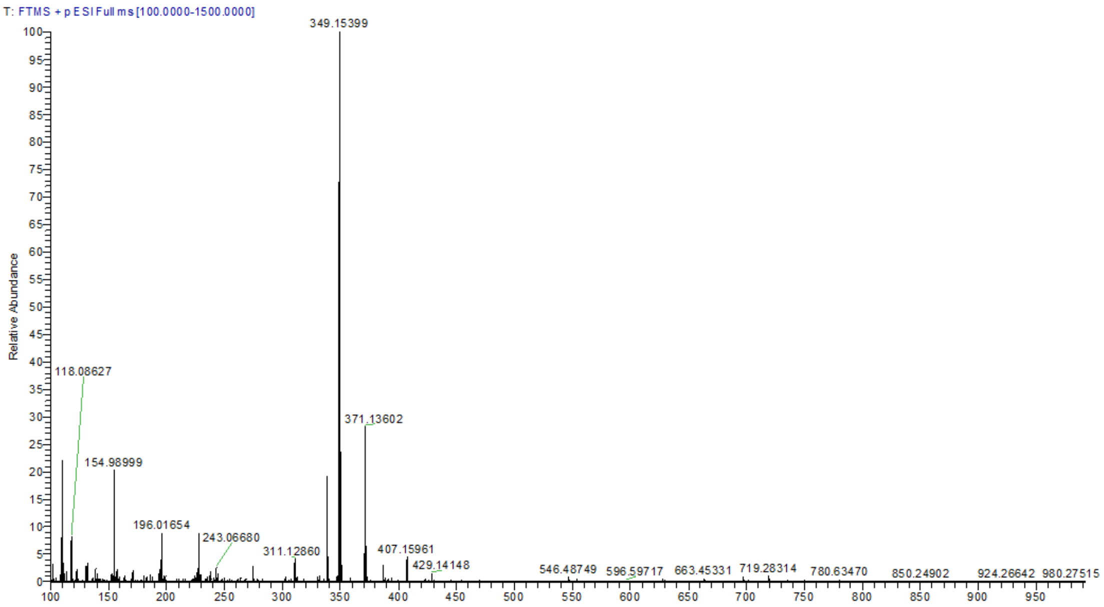

HRMS spectrum of **C3**

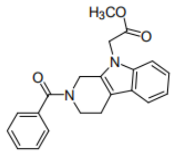

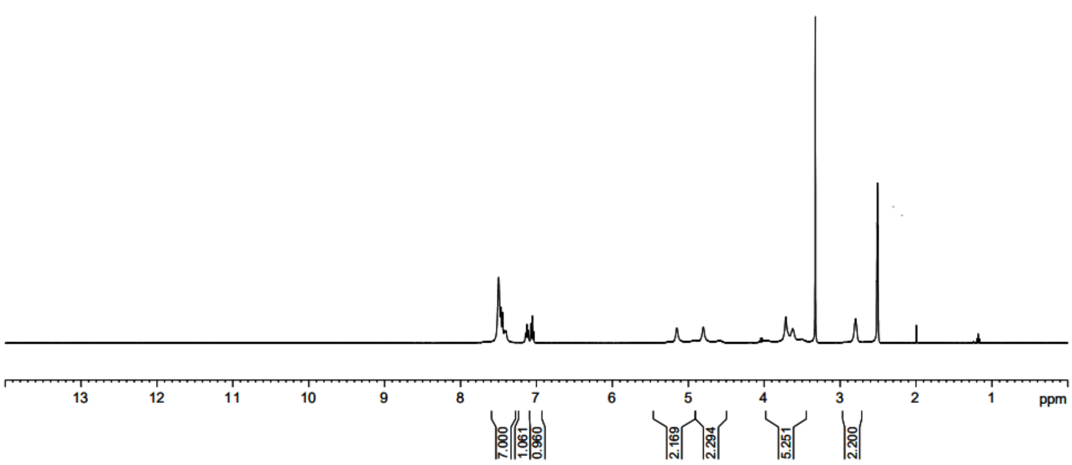

^{1}H NMR spectrum of **C3**

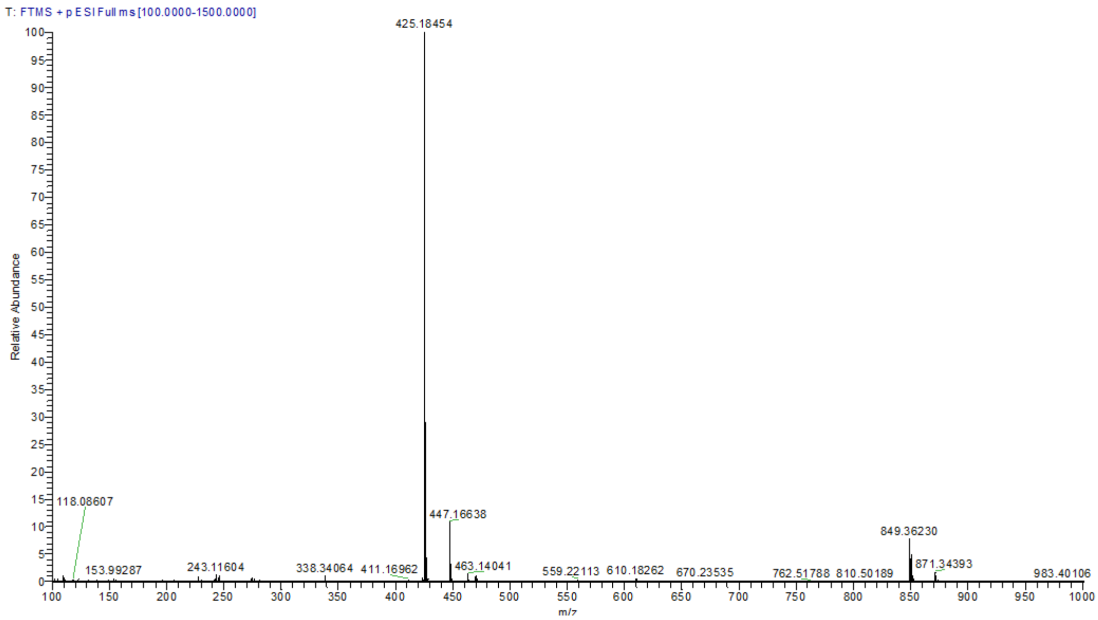

HRMS spectrum of **C4**

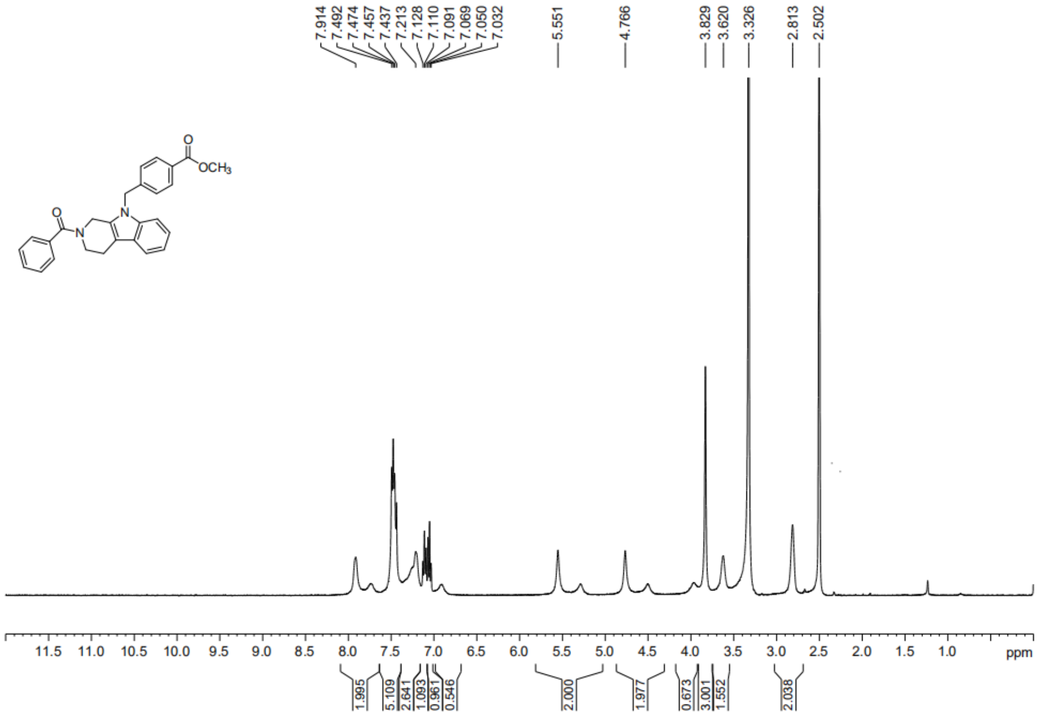

^{1}H NMR spectrum of **C4**

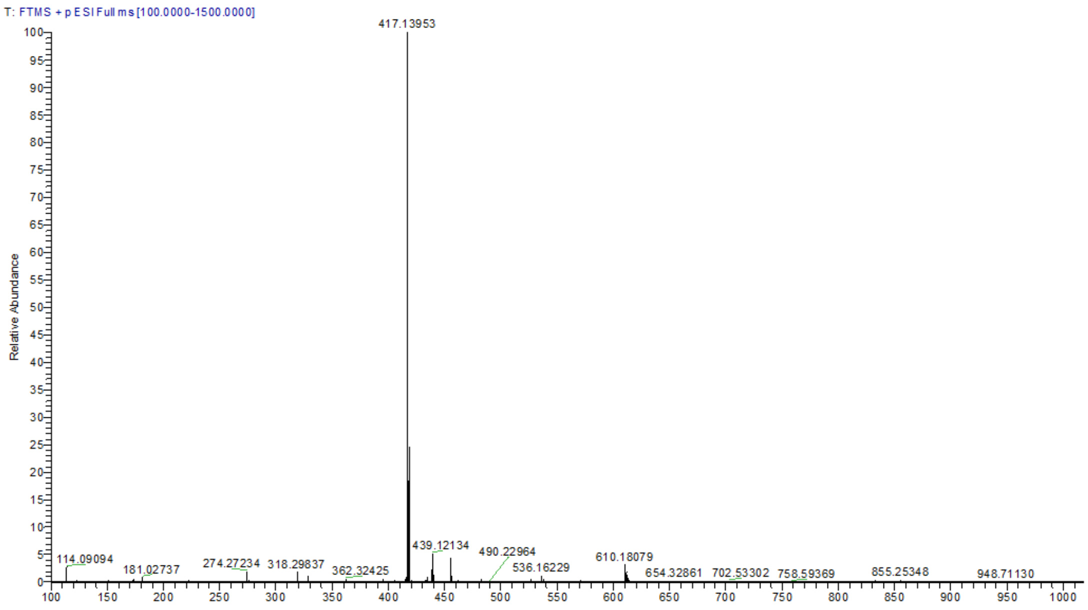

HRMS spectrum of **C5**

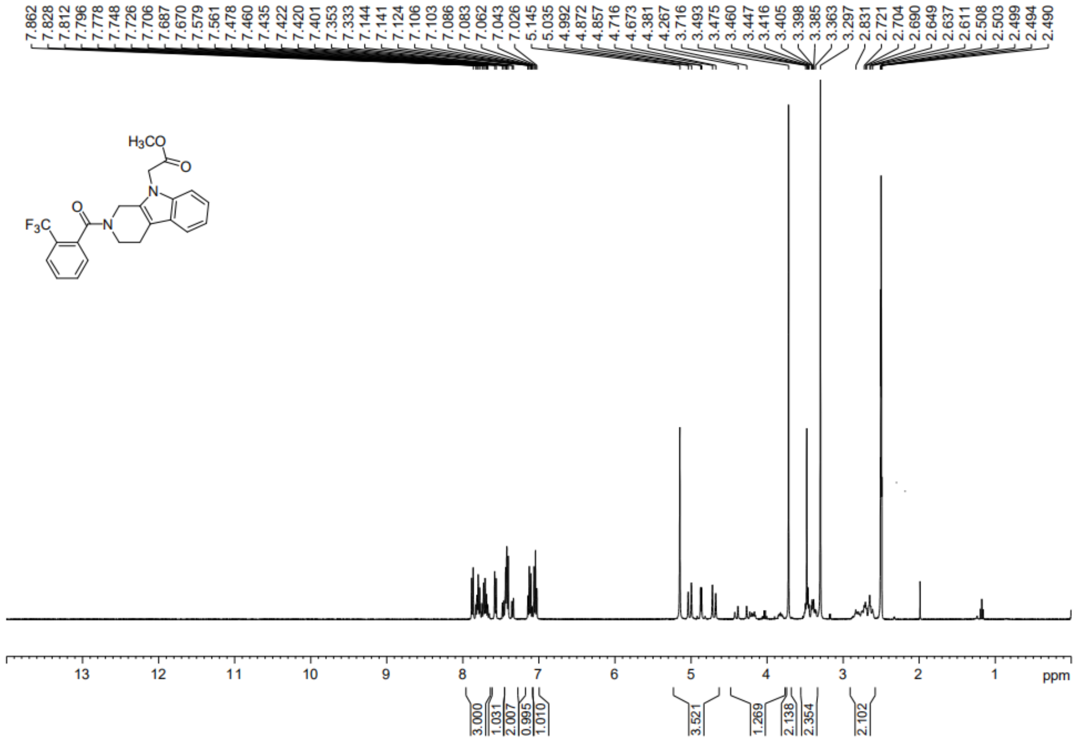

^{1}H NMR spectrum of **C5**

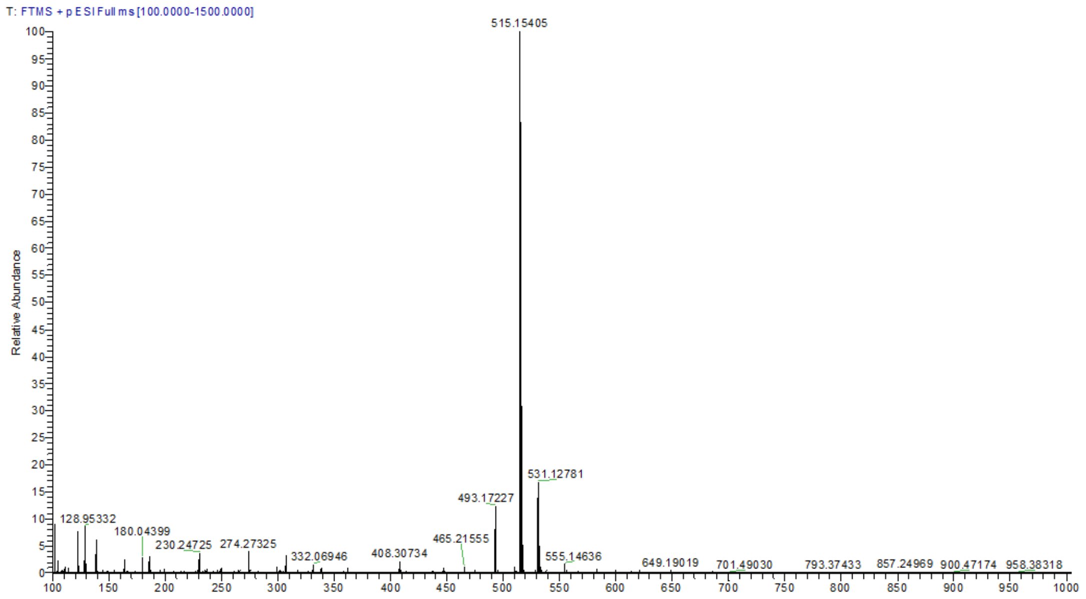

HRMS NMR spectrum of **C6**

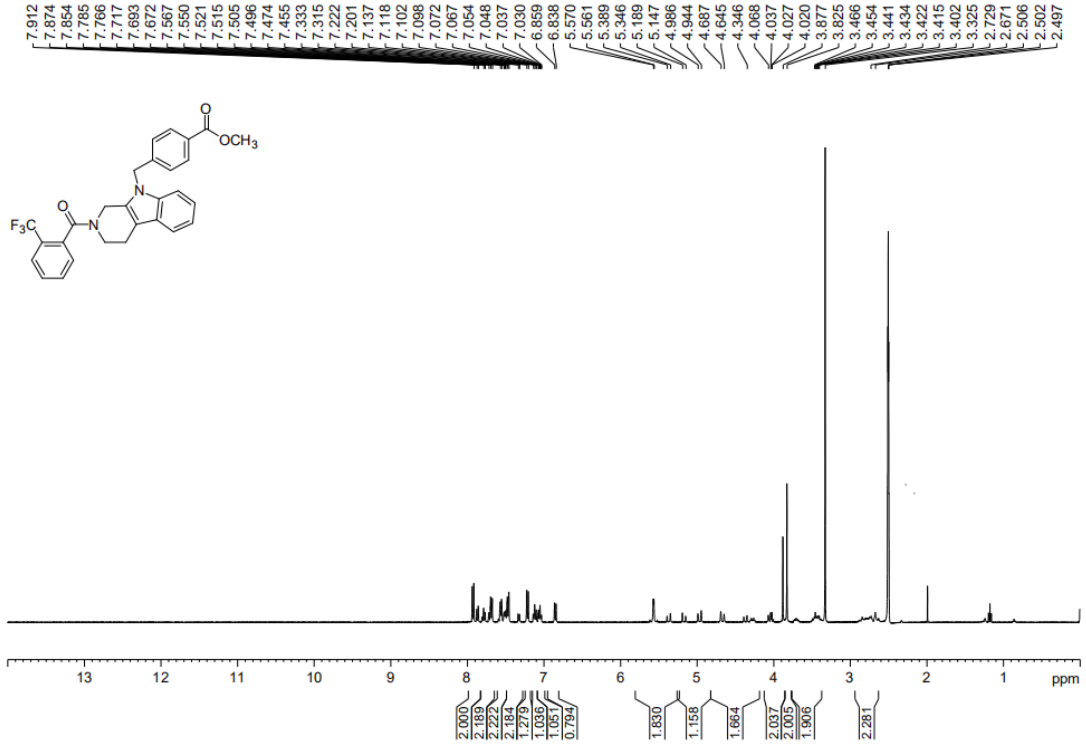

^{1}H NMR spectrum of **C6**

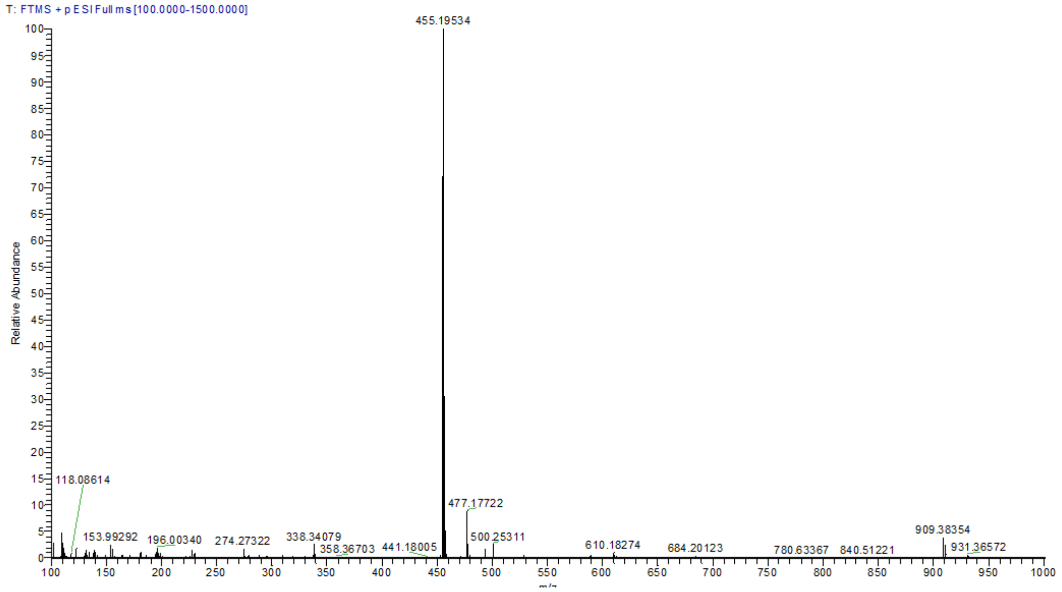

HRMS spectrum of **C7**

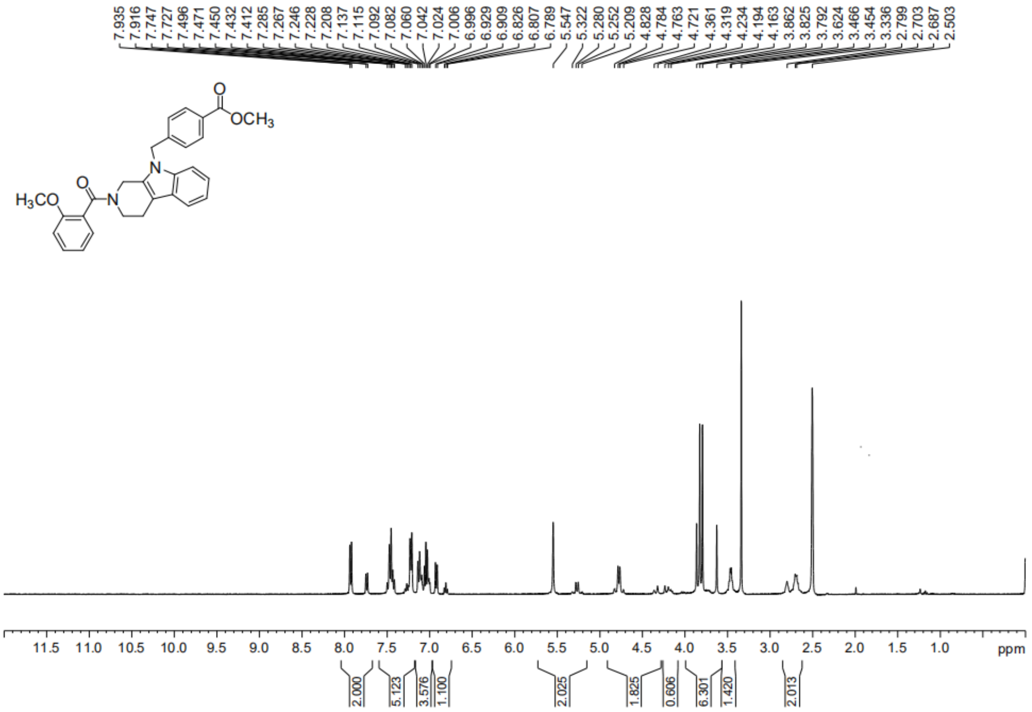

^{1}H NMR spectrum of **C7**

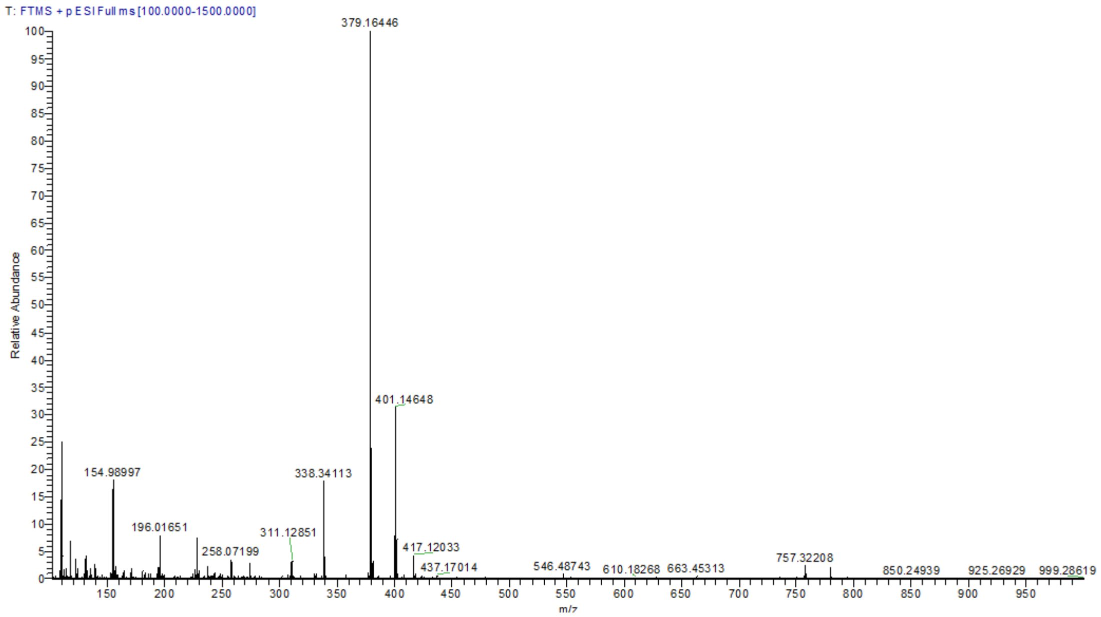

HRMS spectrum of **C8**

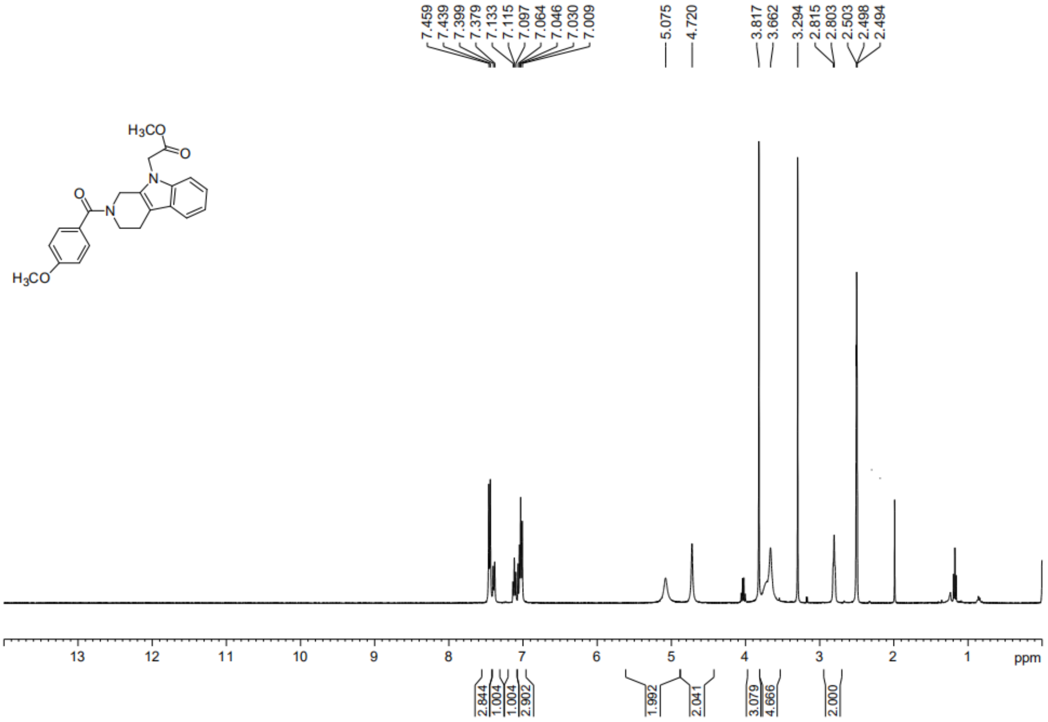

^{1}H NMR spectrum of **C8**

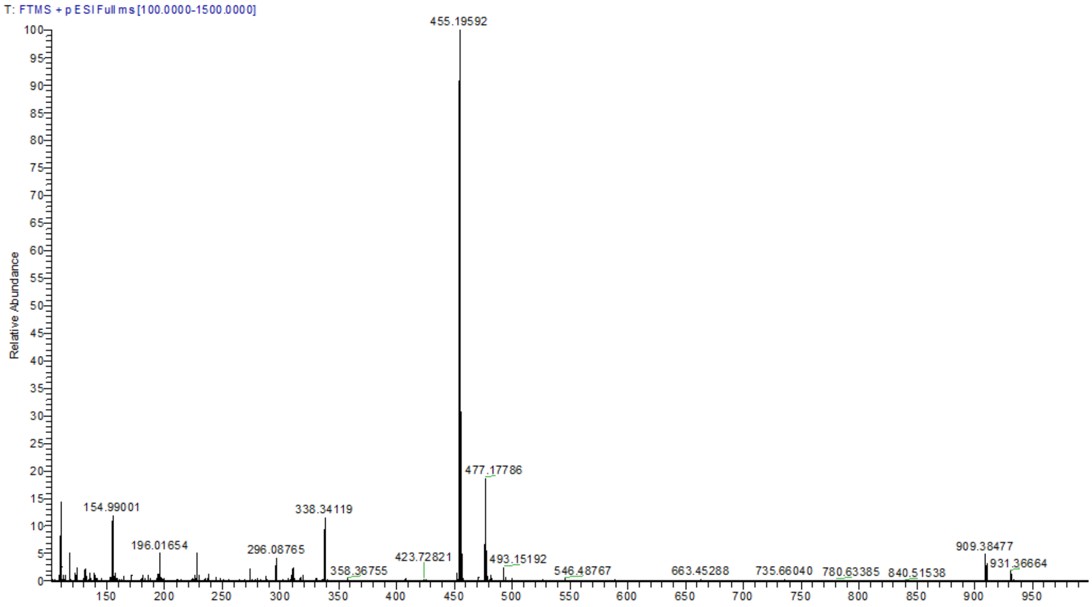

HRMS spectrum of **C9**

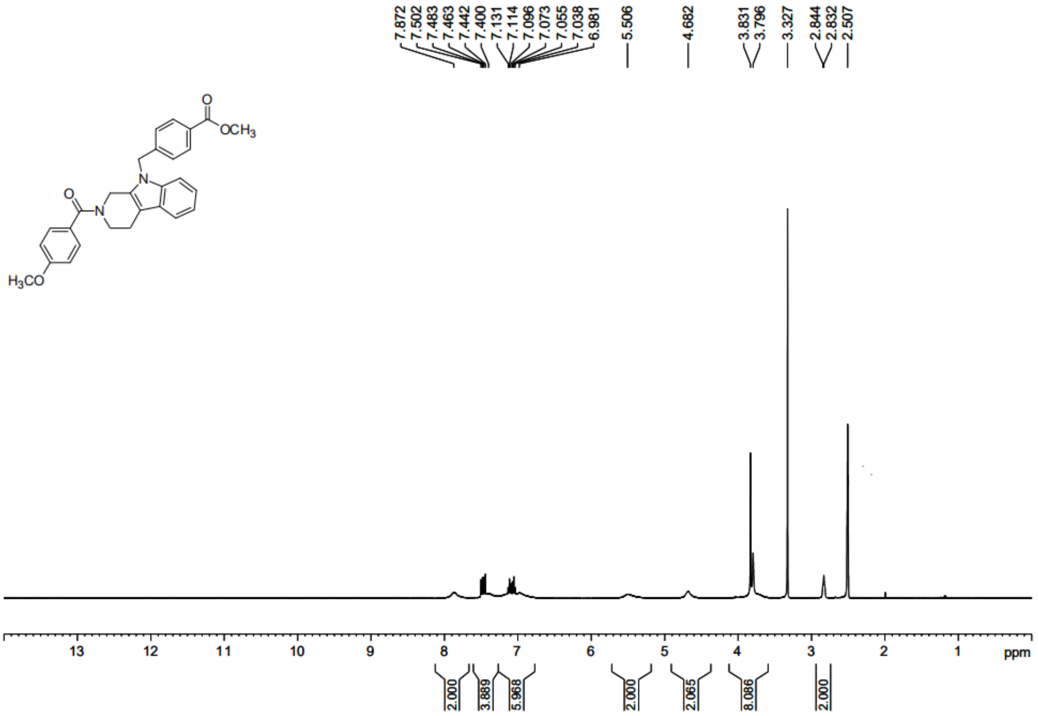

^{1}H NMR spectrum of **C9**

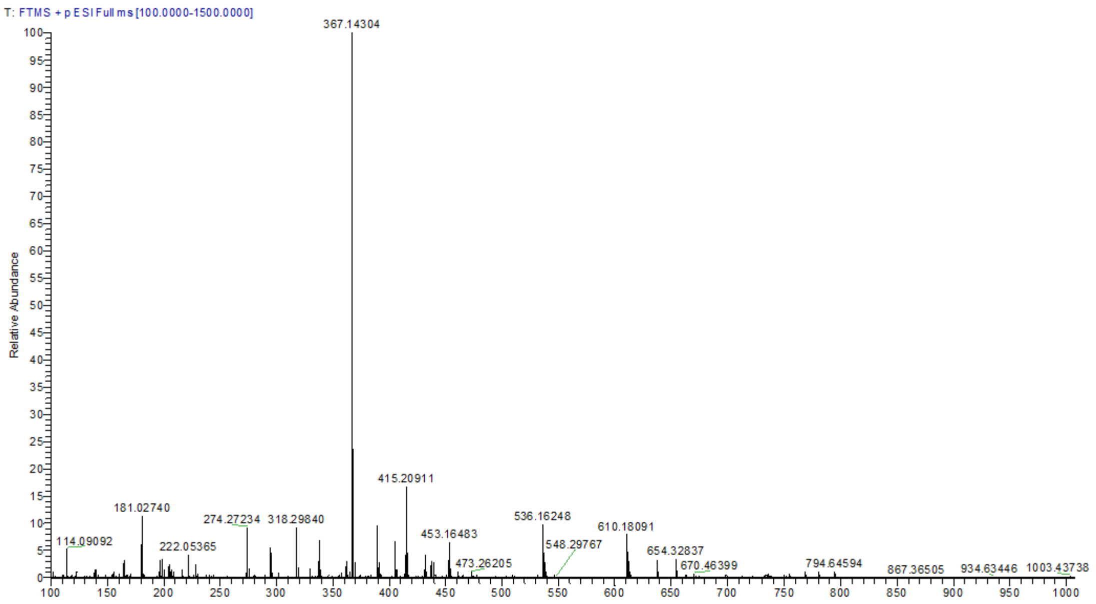

HRMS spectrum of **C10**

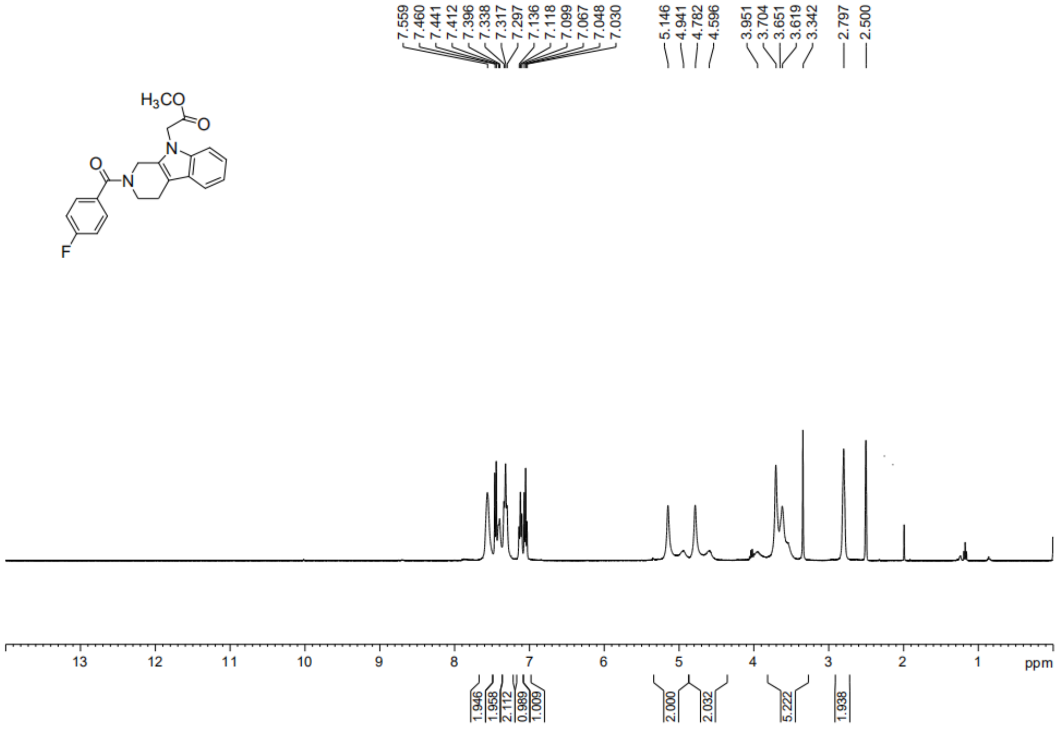

^{1}H NMR spectrum of **C10**

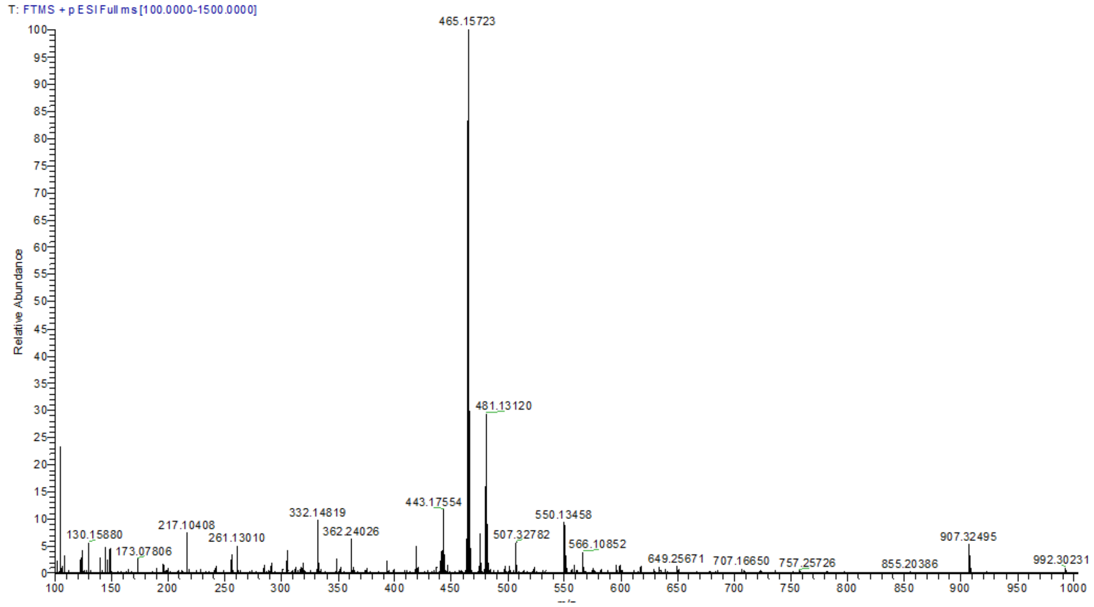

HRMS spectrum of **C11**

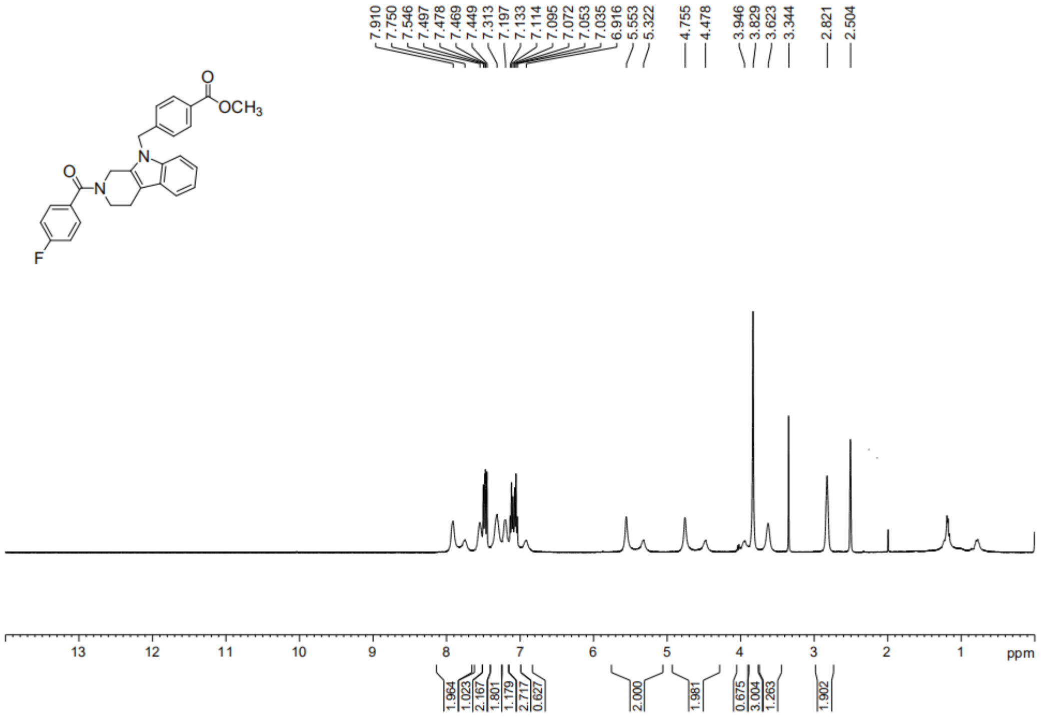

^{1}H NMR spectrum of **C11**

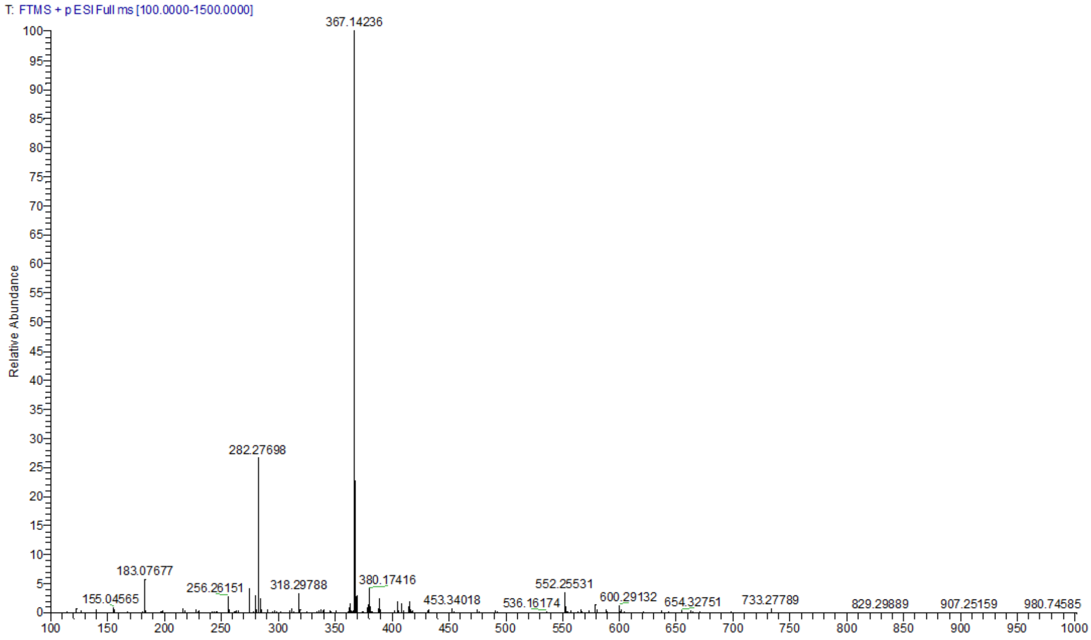

HRMS spectrum of **C12**

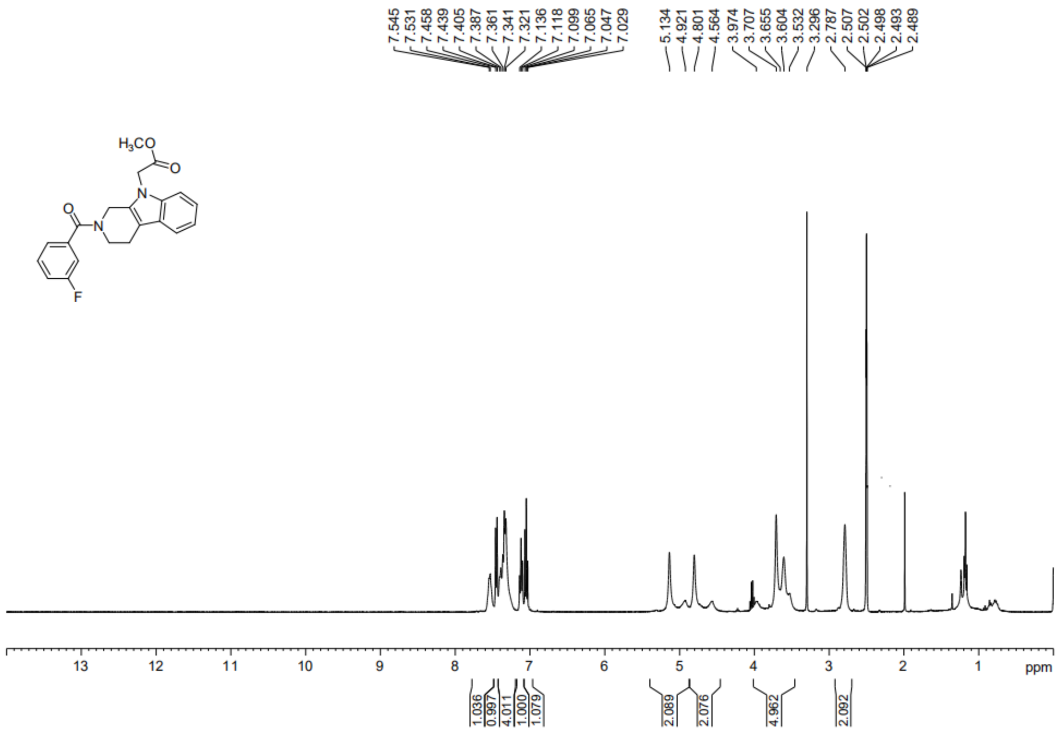

^{1}H NMR spectrum of **C12**

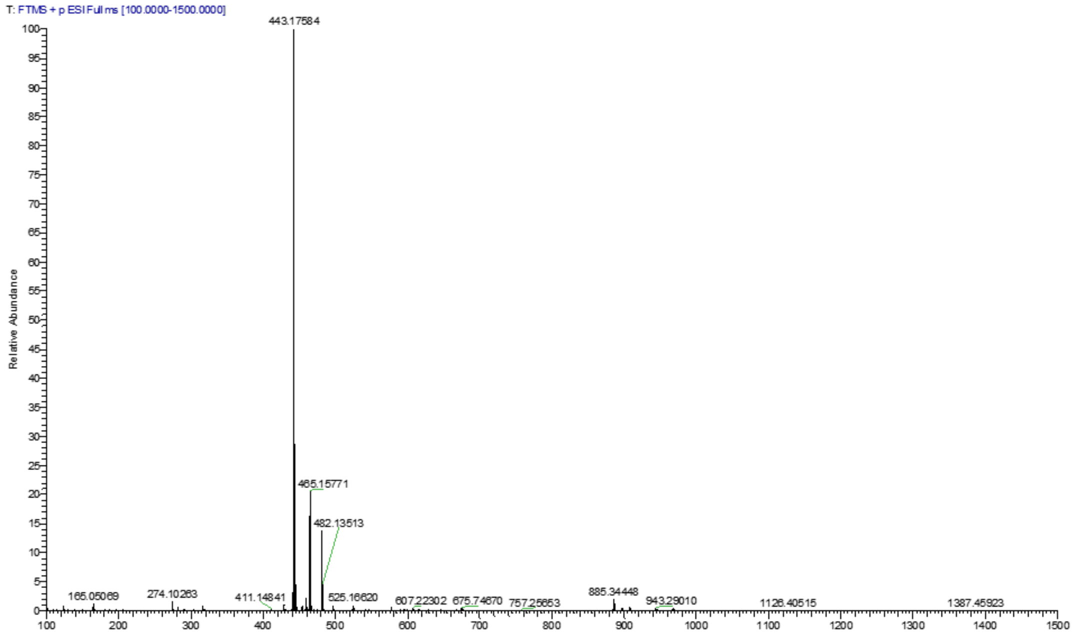

HRMS spectrum of **C13**

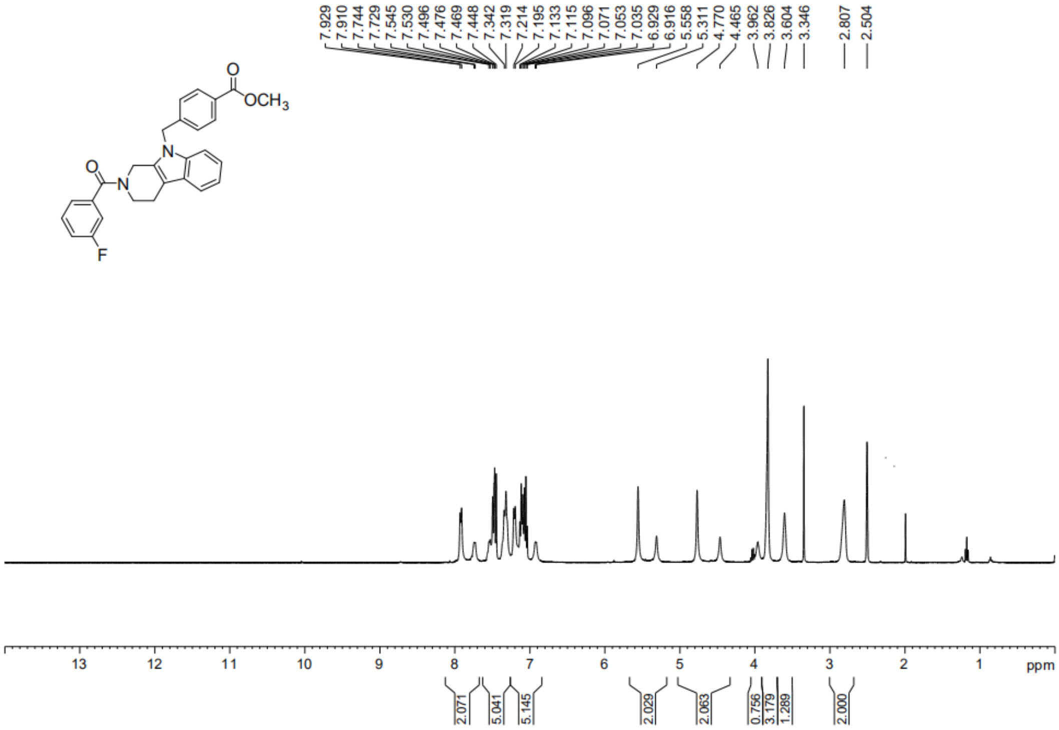

^{1}H NMR spectrum of **C13**

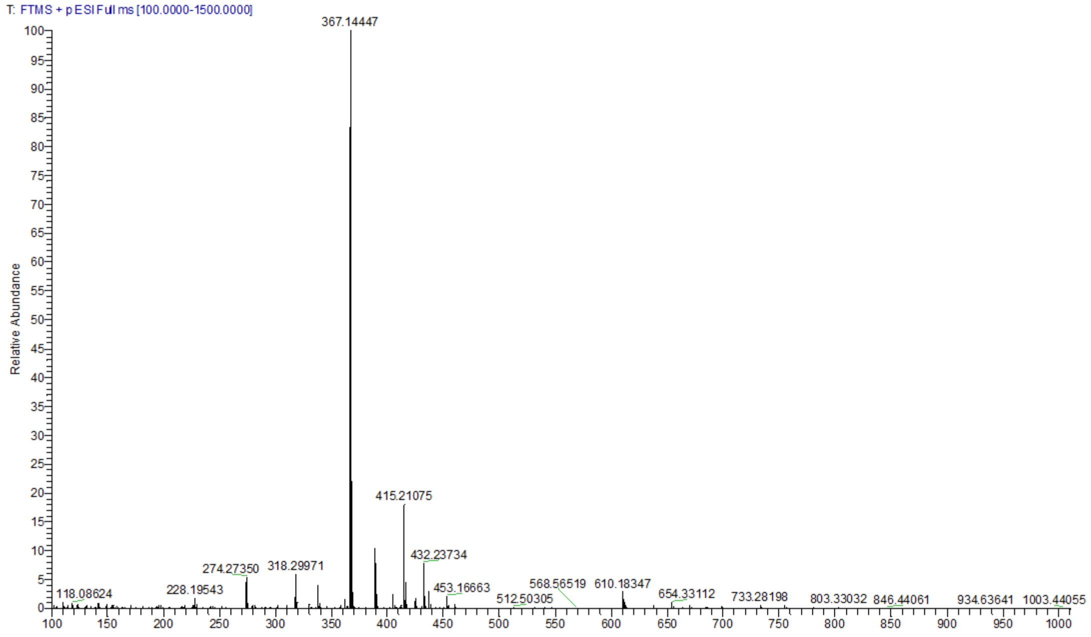

HRMS NMR spectrum of **C14**

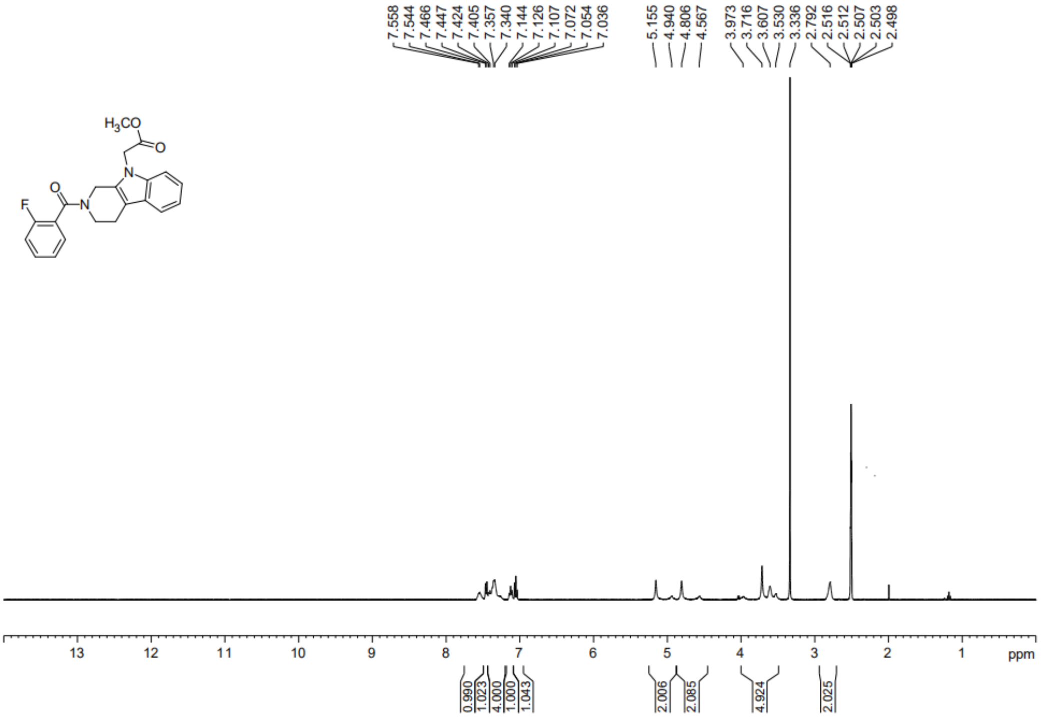

^{1}H NMR spectrum of **C14**

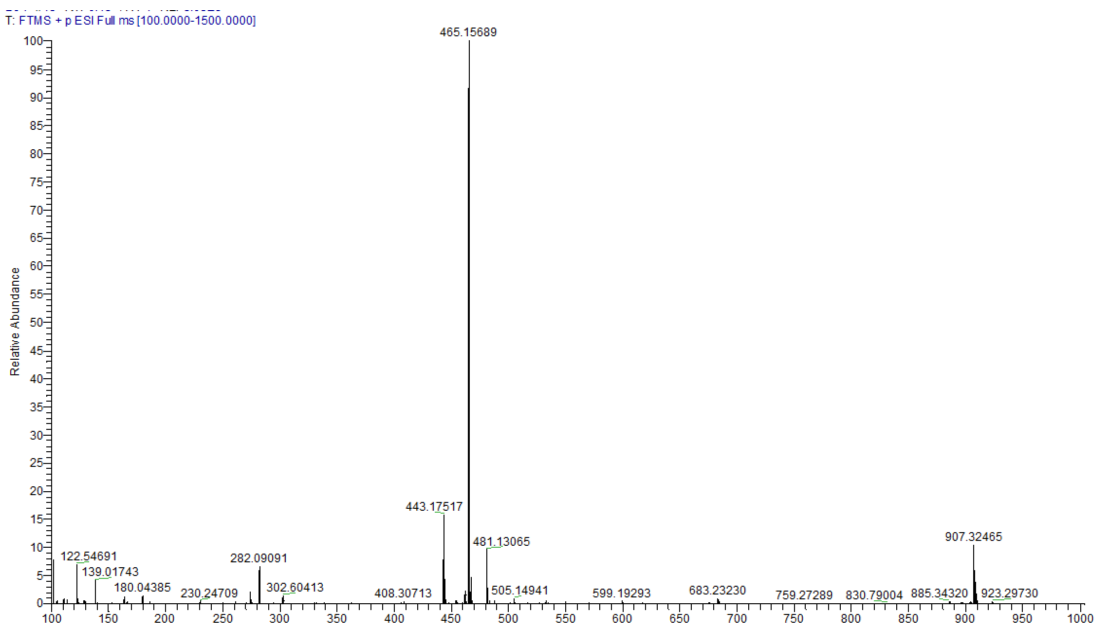

HRMS spectrum of **C15**

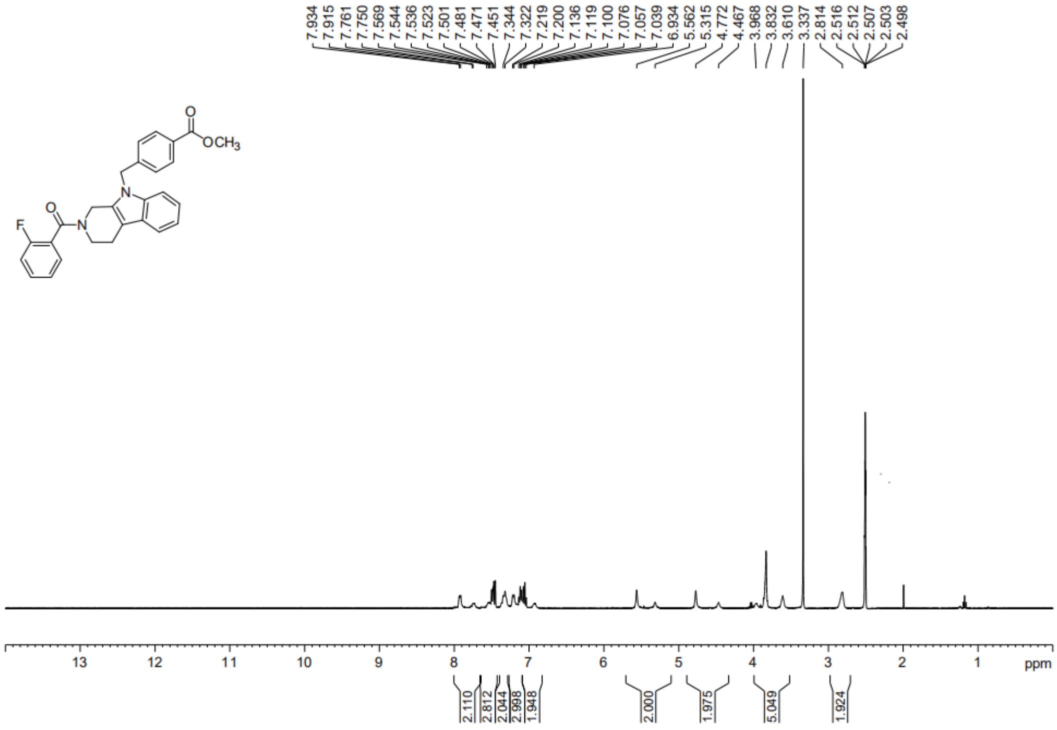

^{1}H NMR spectrum of **C15**

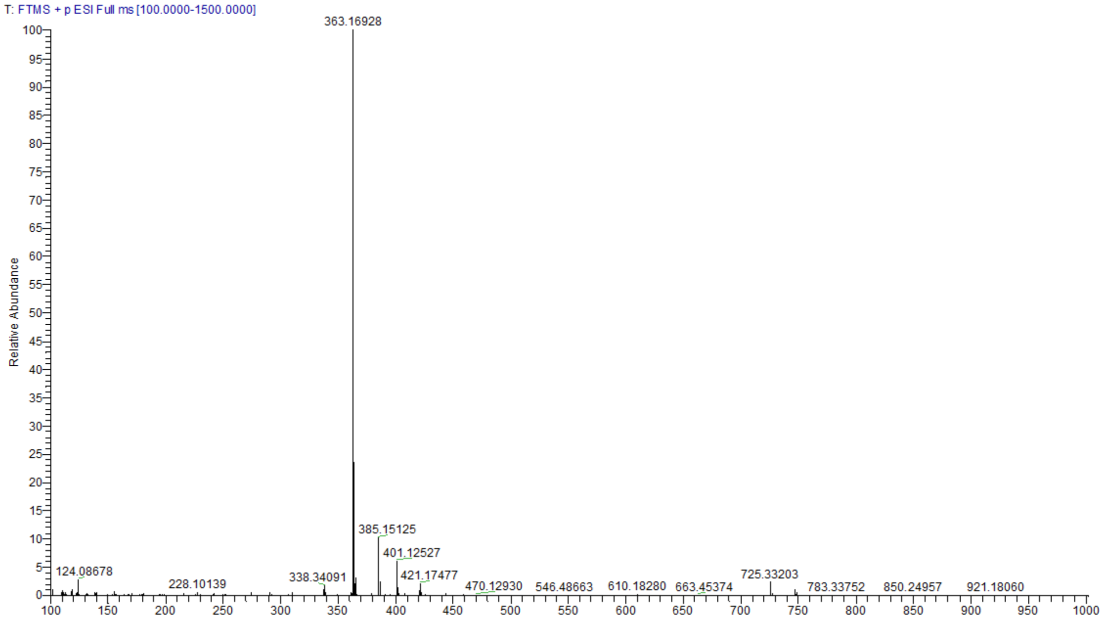

HRMS spectrum of **C16**

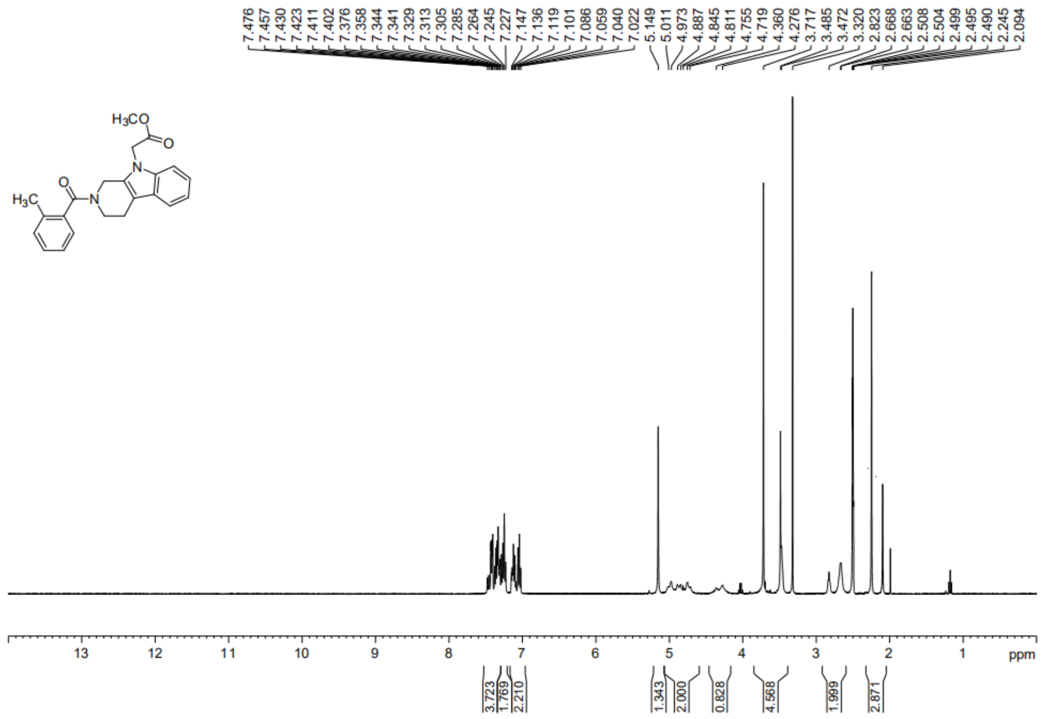

^{1}H NMR spectrum of **C16**

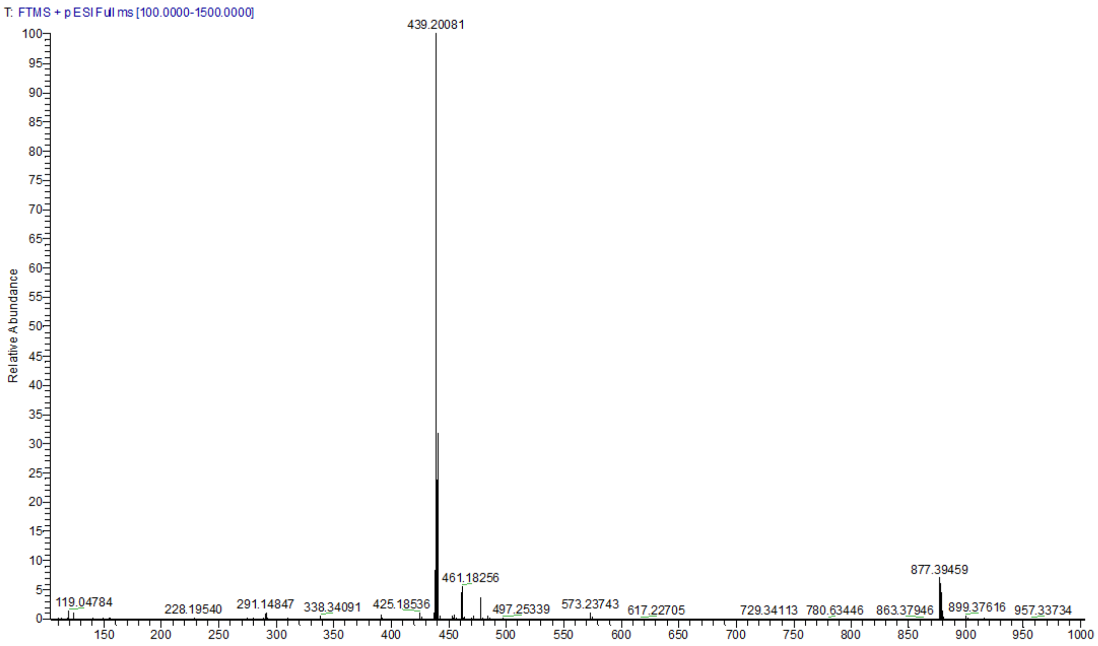

HRMS spectrum of **C17**

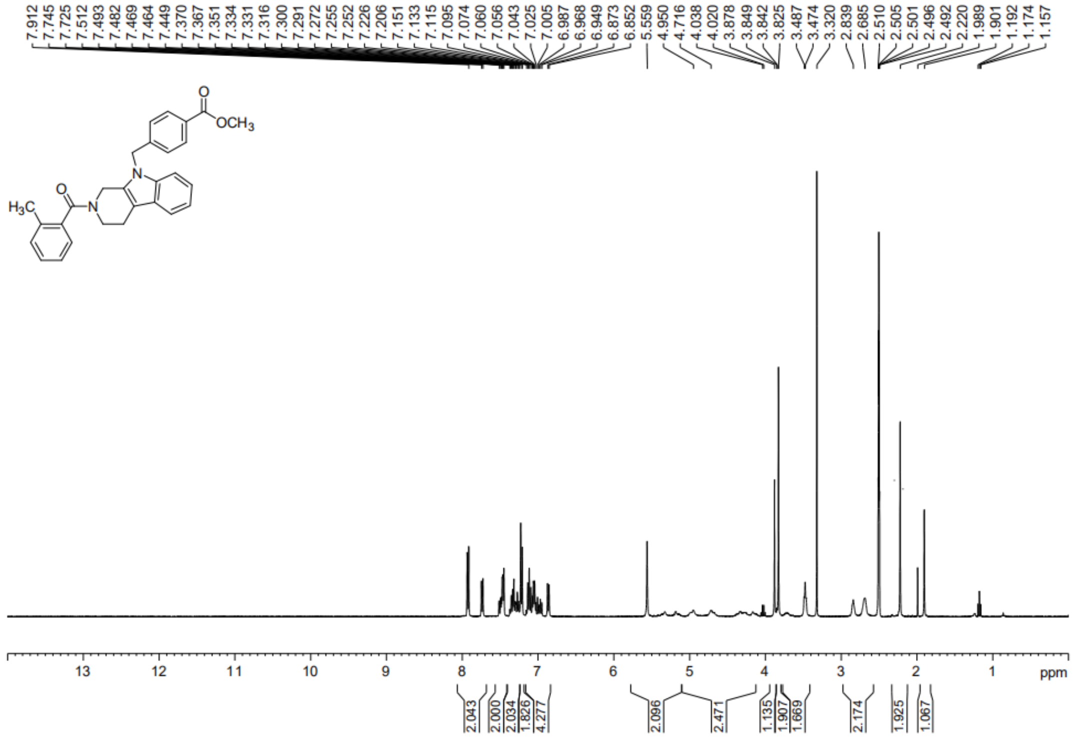

^{1}H NMR spectrum of **C17**

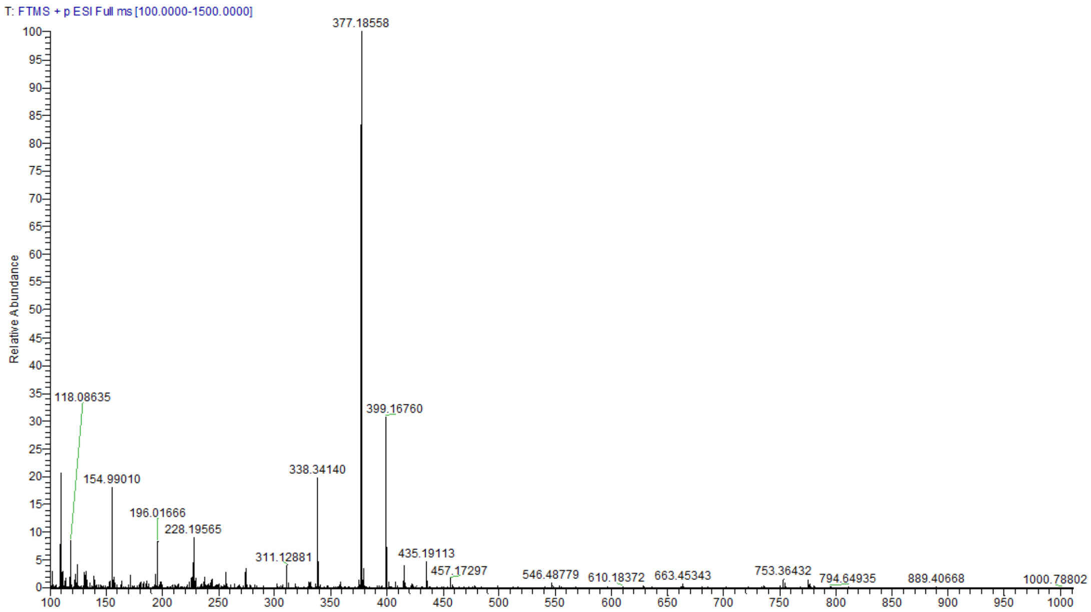

HRMS spectrum of **C18**

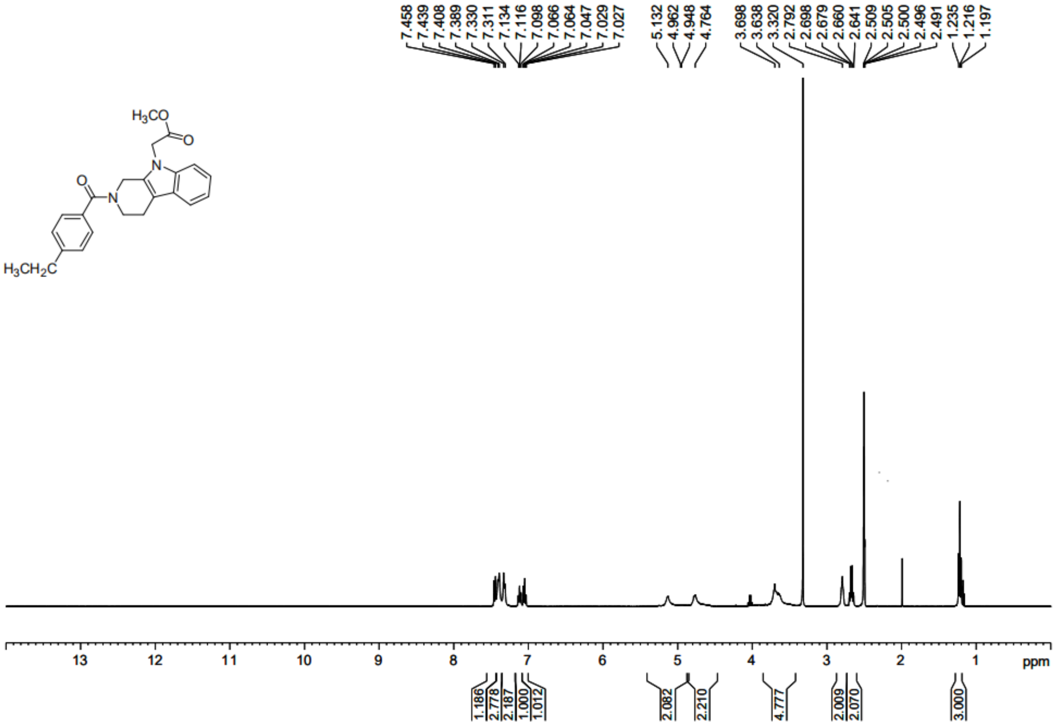

^{1}H NMR spectrum of **C18**

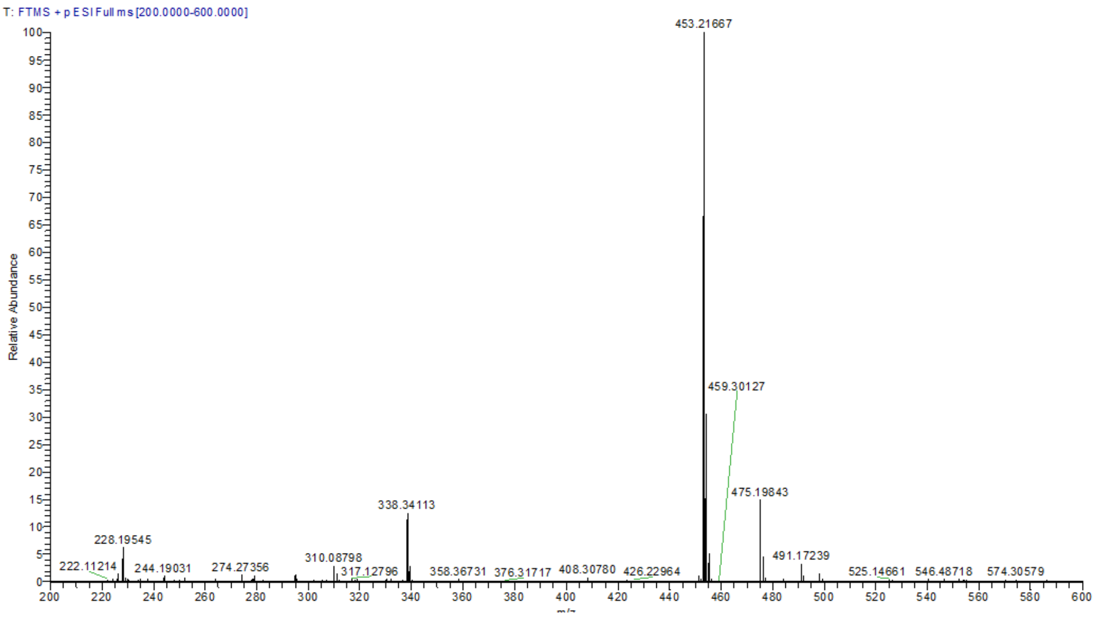

HRMS spectrum of **C19**

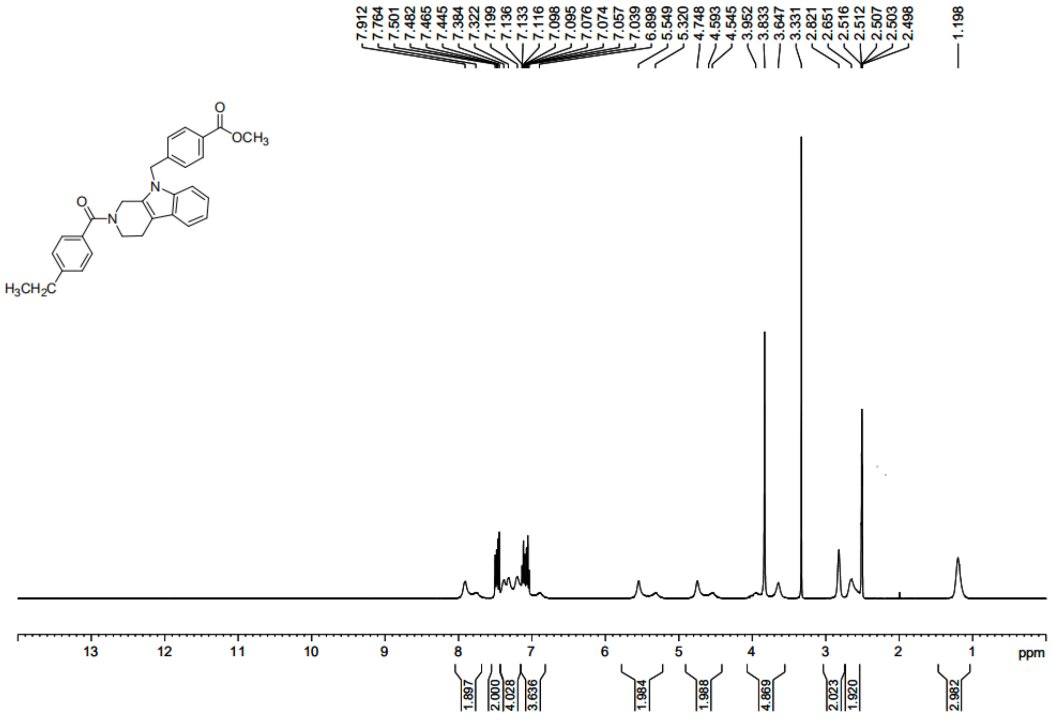

^{1}H NMR spectrum of **C19**

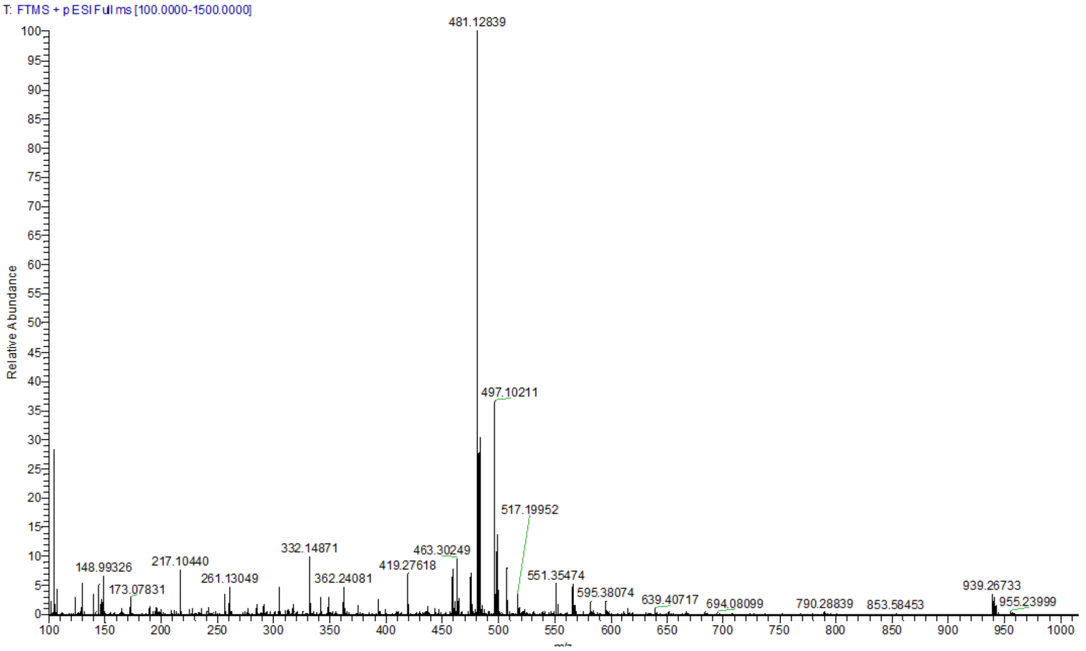

HRMS spectrum of **C20**

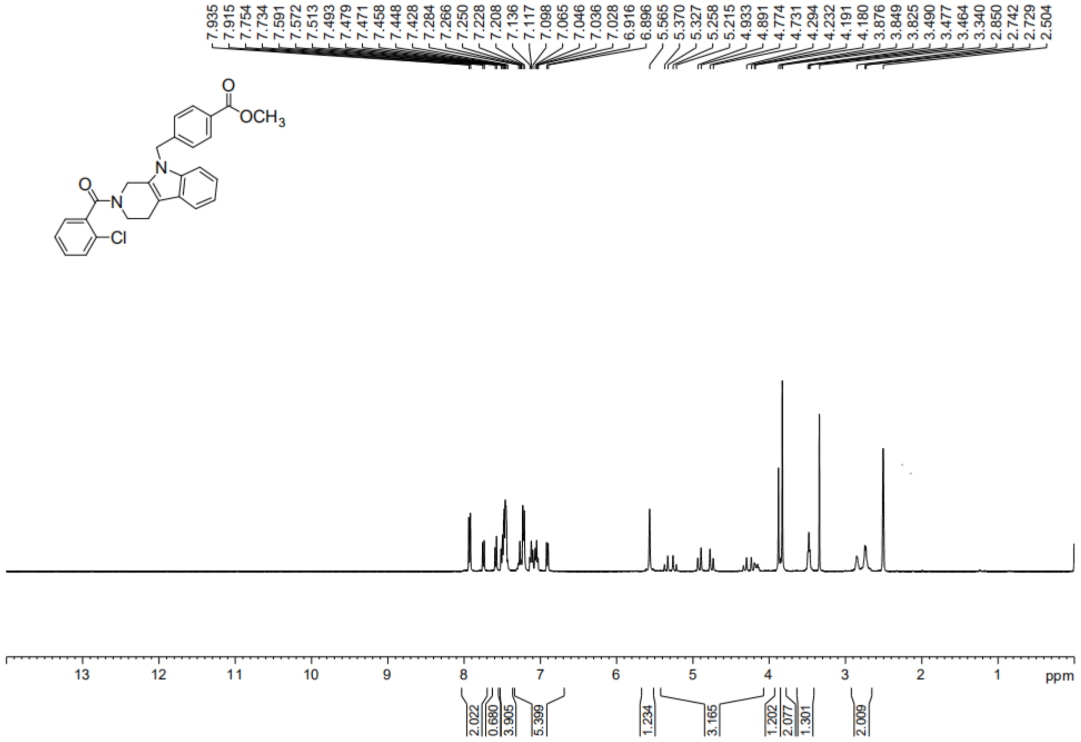

^{1}H NMR spectrum of **C20**

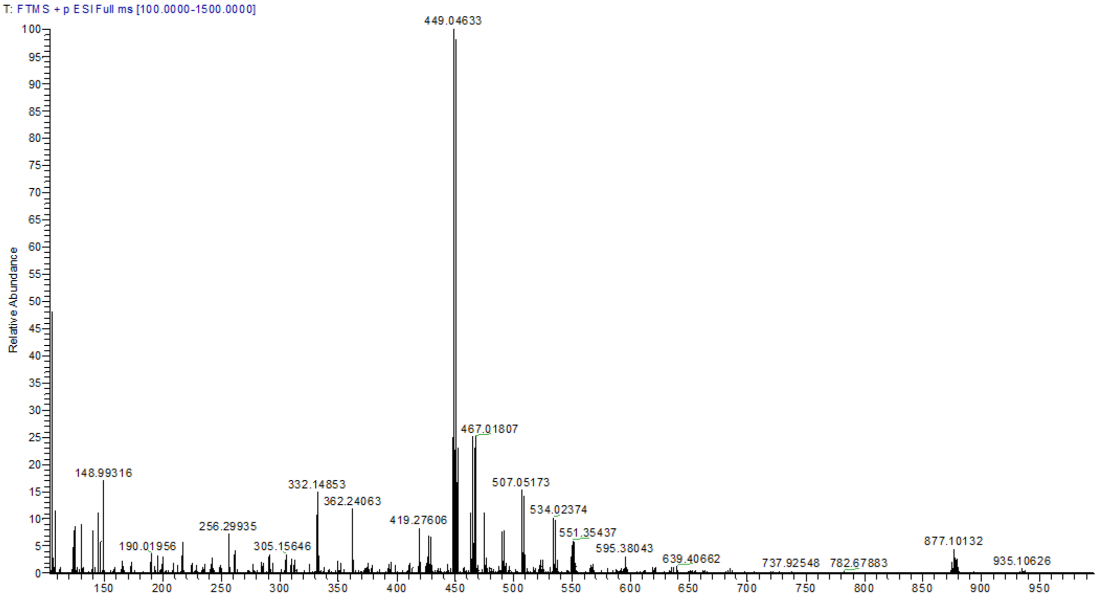

HRMS spectrum of **C21**

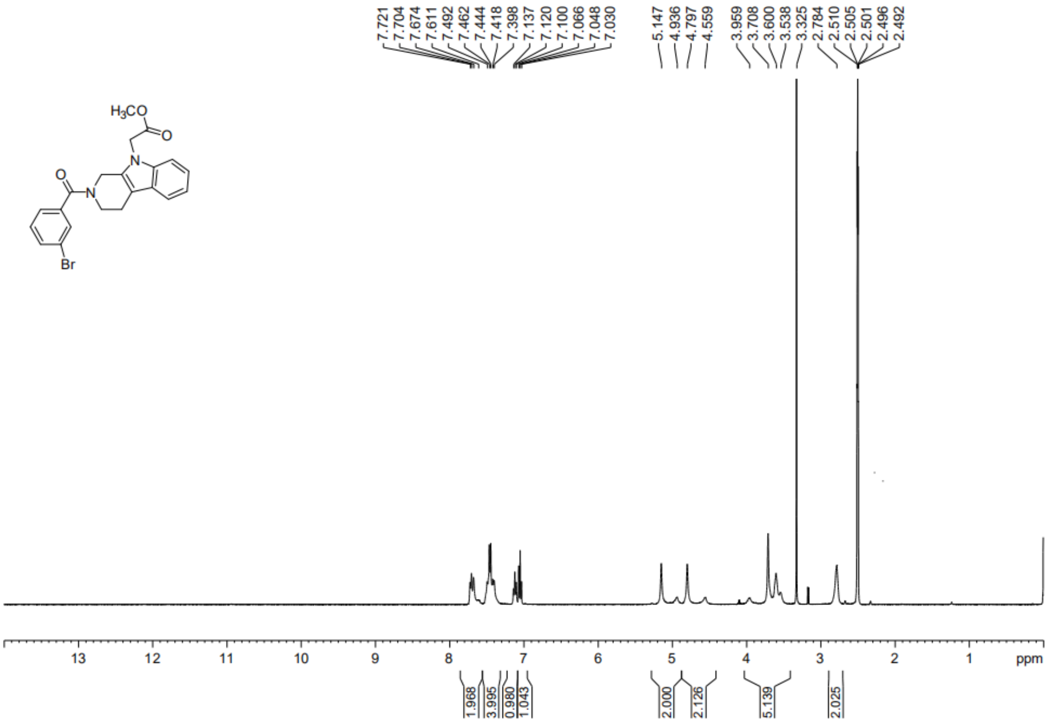

^{1}H NMR spectrum of **C21**

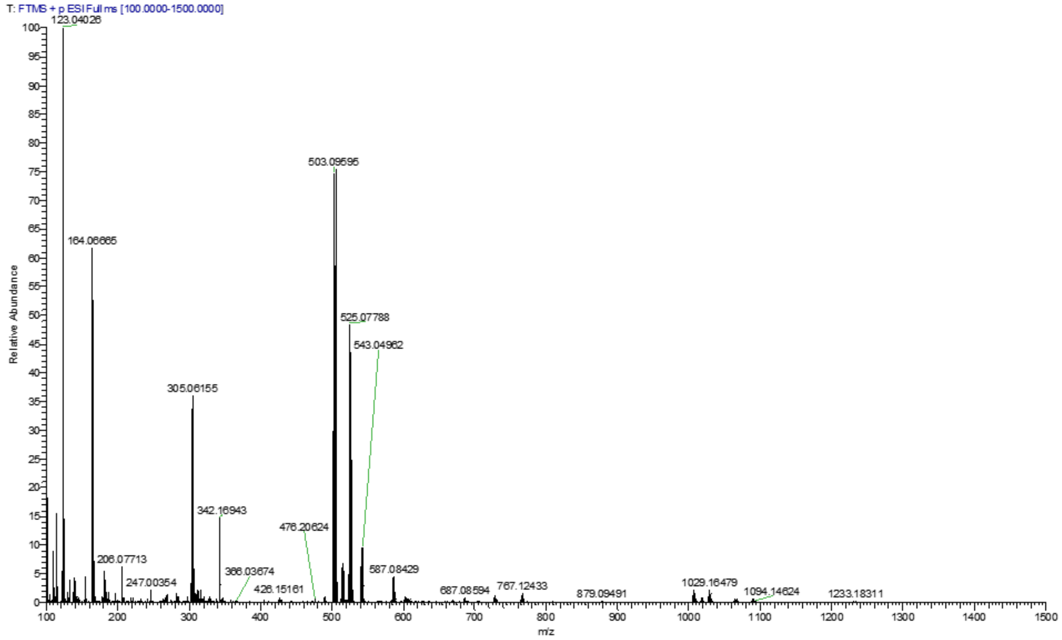

HRMS spectrum of **C22**

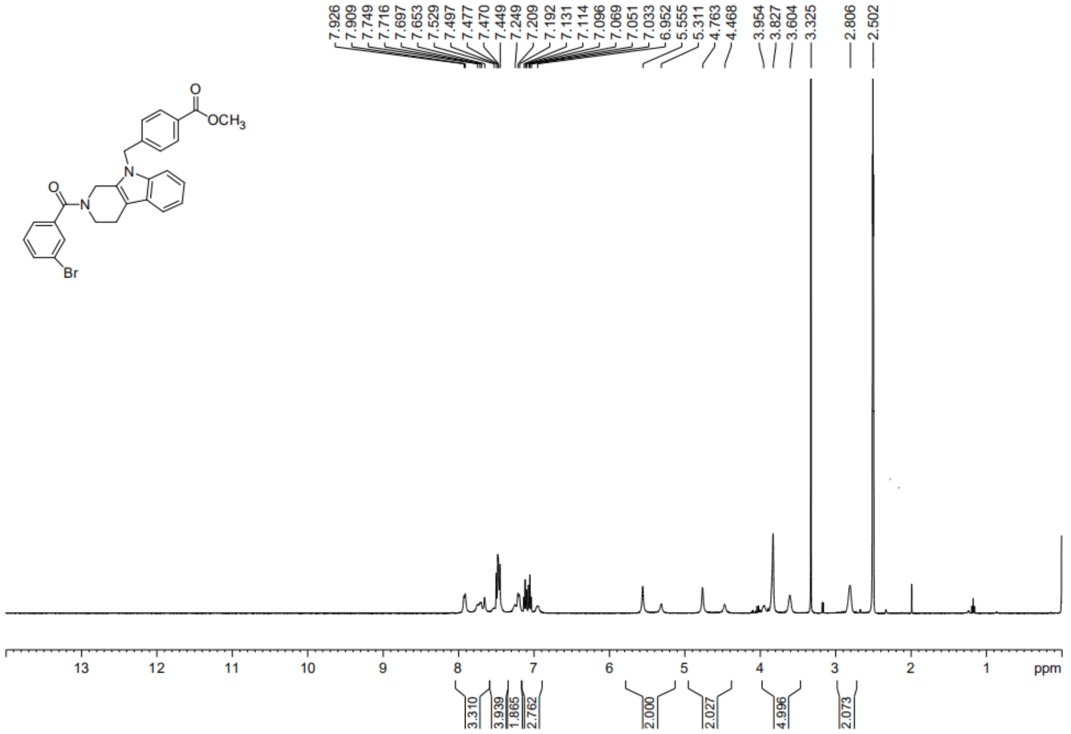

^{1}H NMR spectrum of **C22**

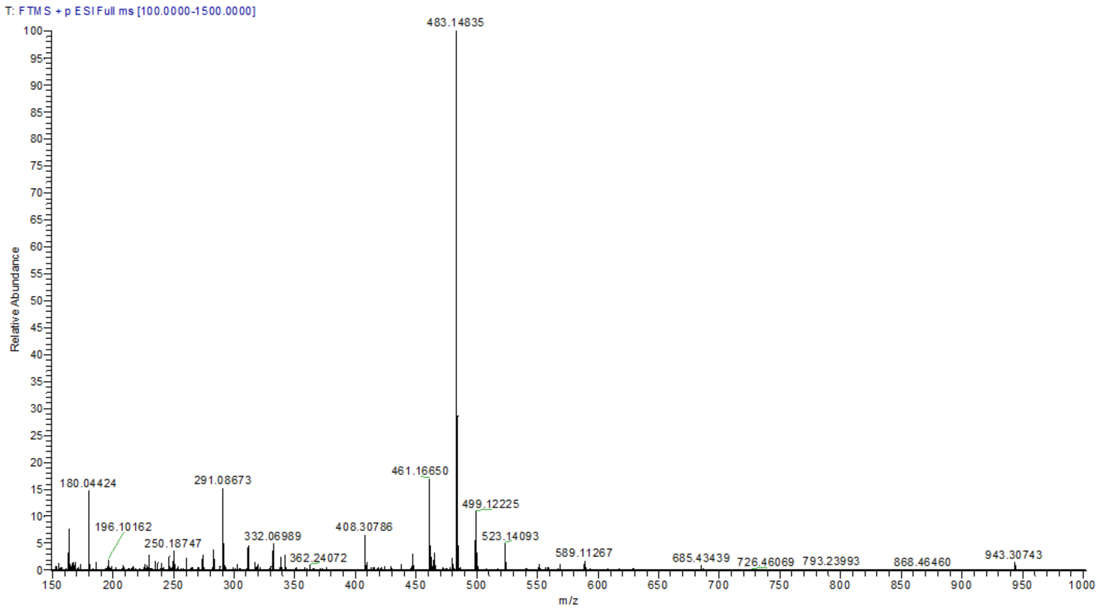

HRMS spectrum of **C23**

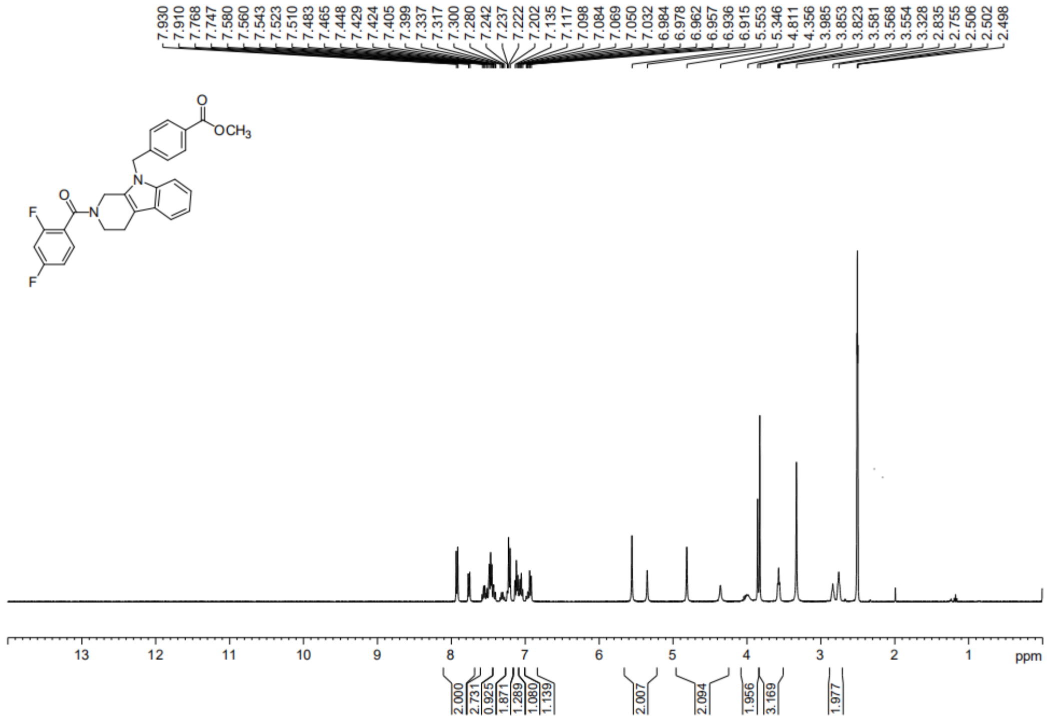

^{1}H NMR spectrum of **C23**

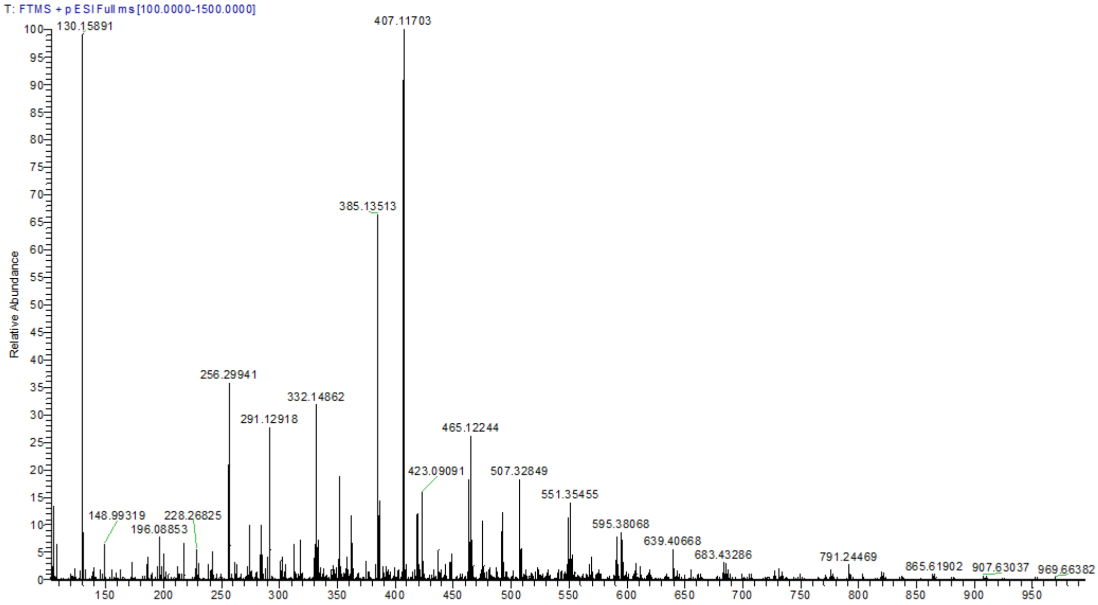

HRMS spectrum of **C24**

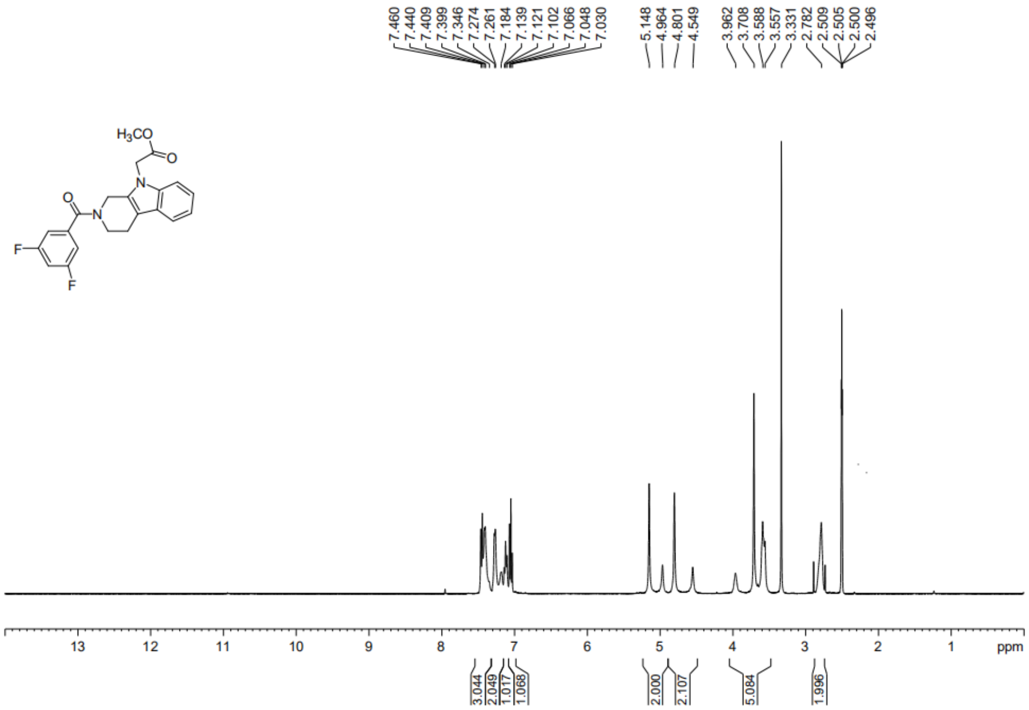

^{1}H NMR spectrum of **C24**

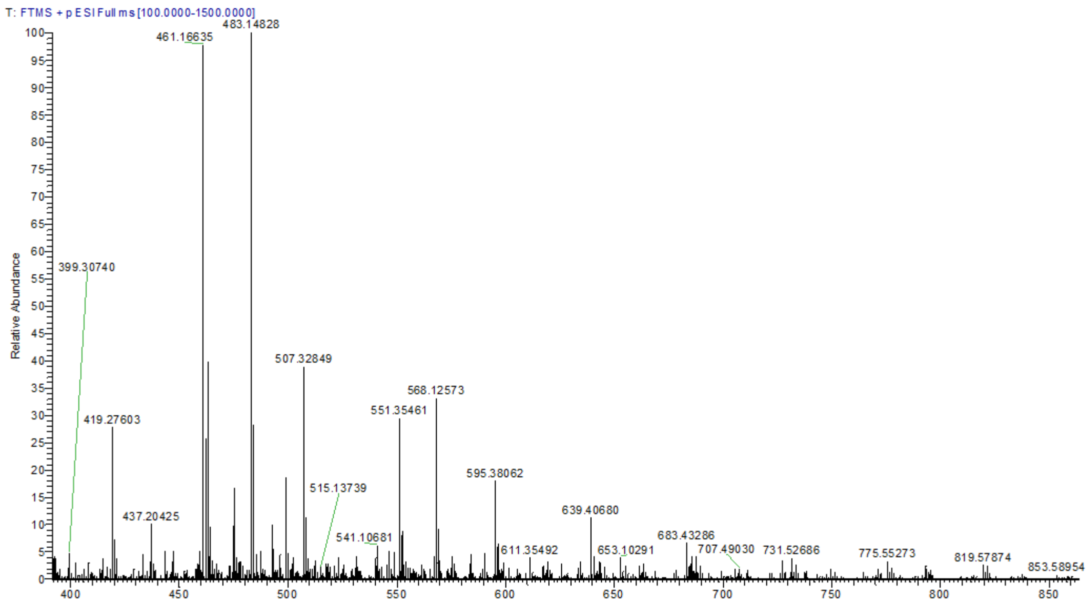

HRMS spectrum of **C25**

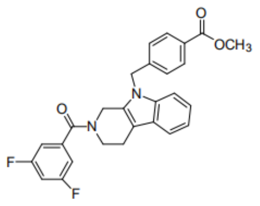

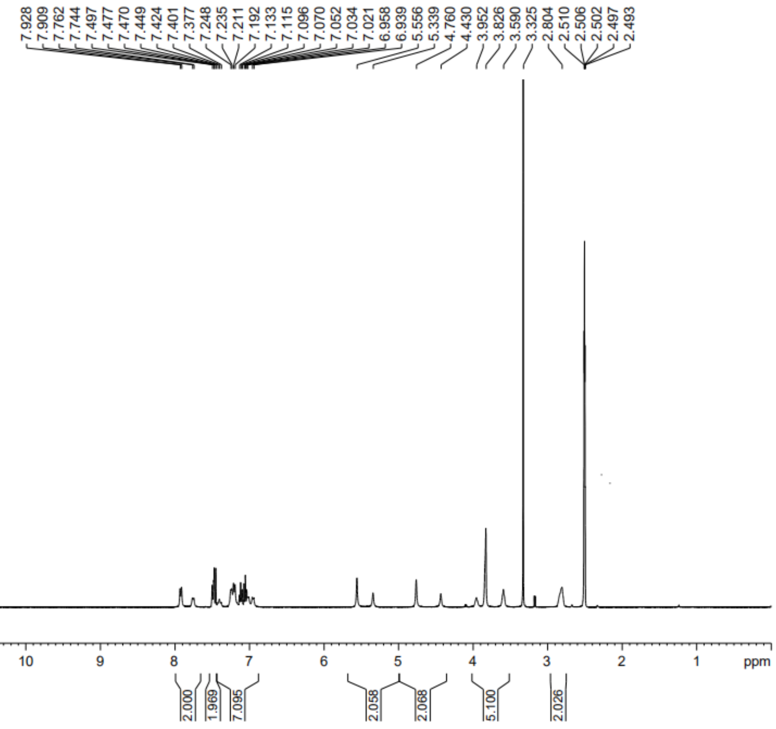

^{1}H NMR spectrum of **C25**

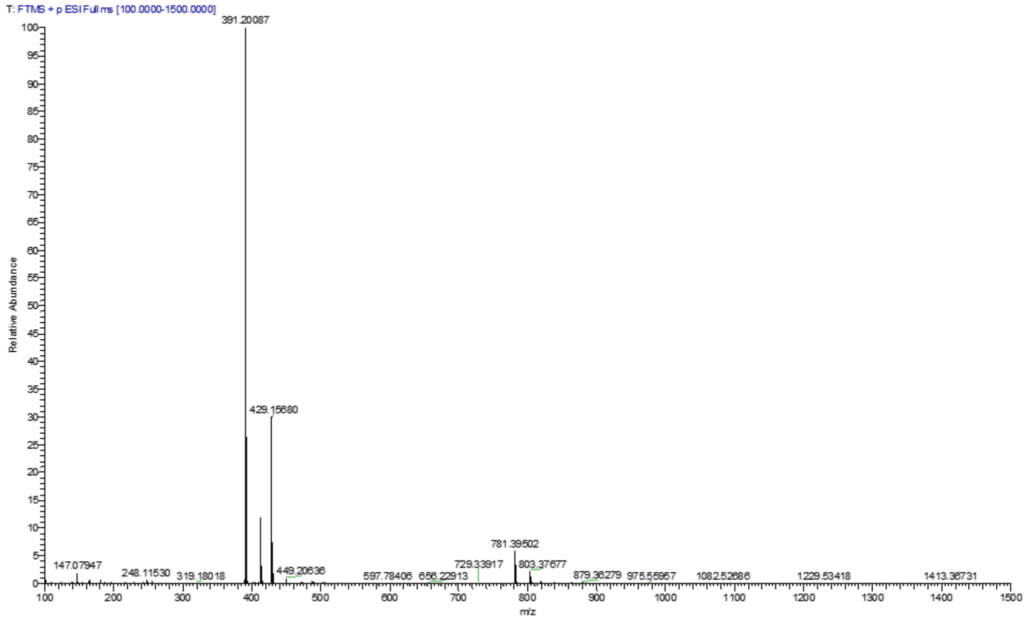

HRMS spectrum of **C26**

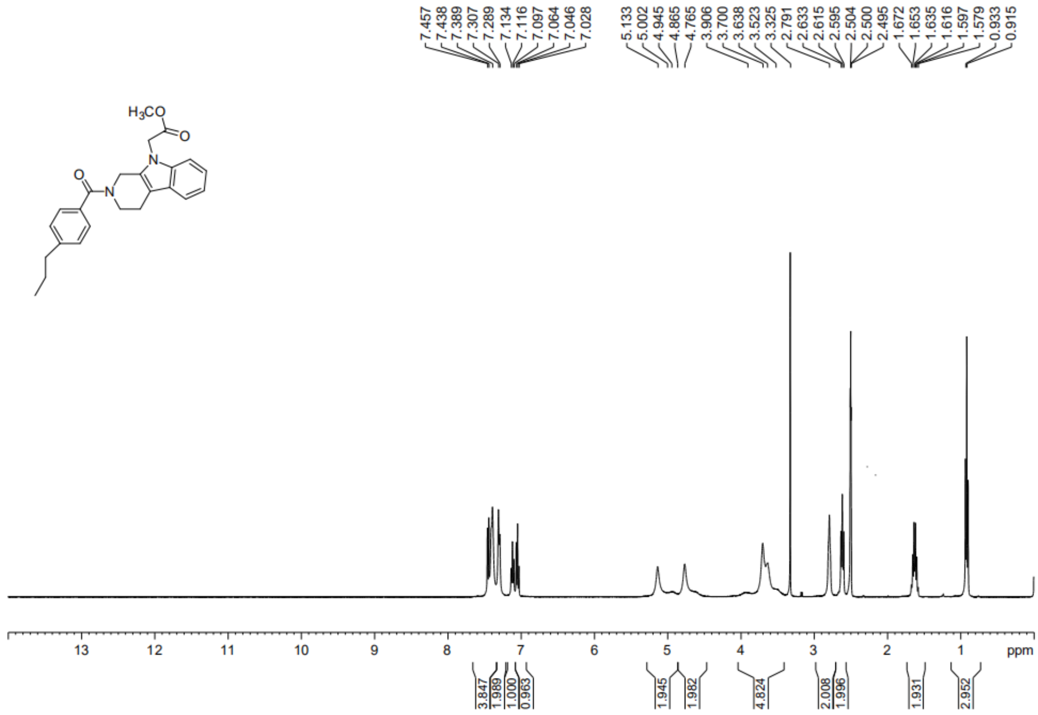

^{1}H NMR spectrum of **C26**

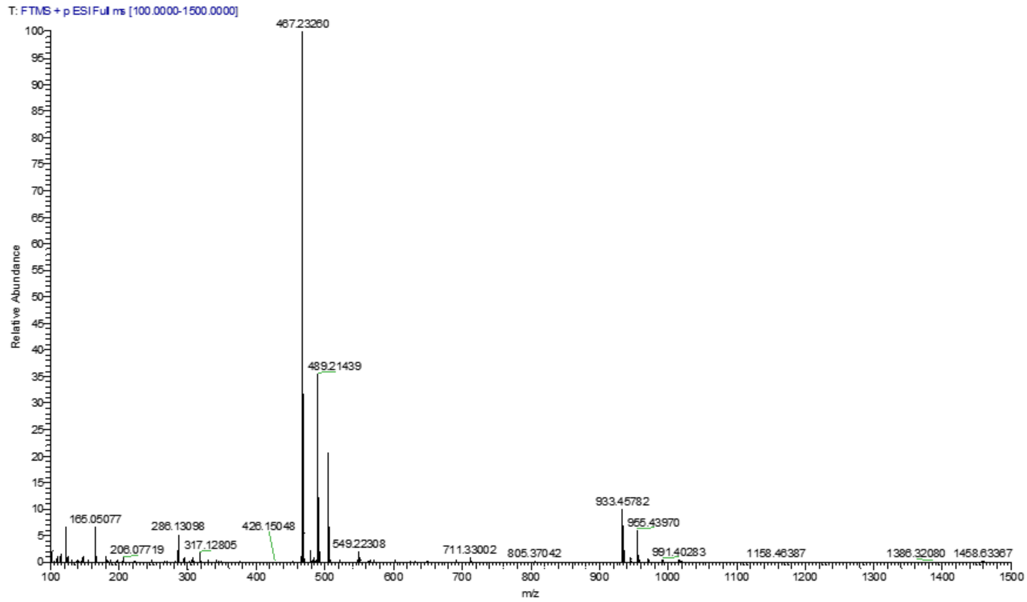

HRMS spectrum of **C27**

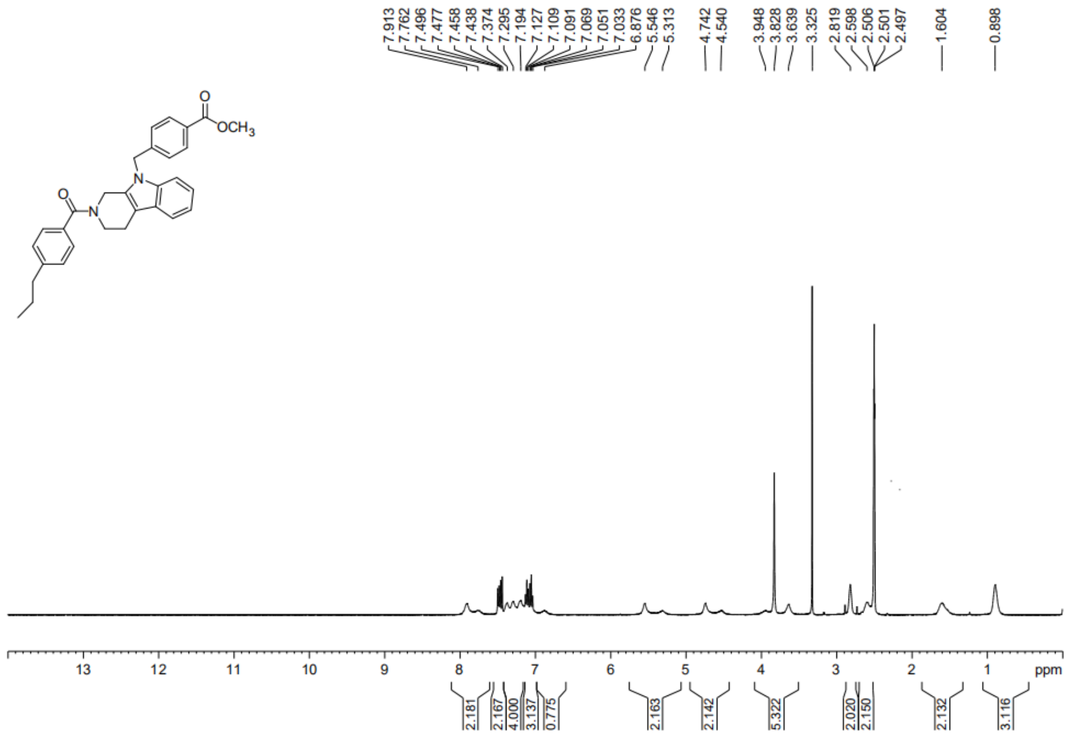

^{1}H NMR spectrum of **C27**

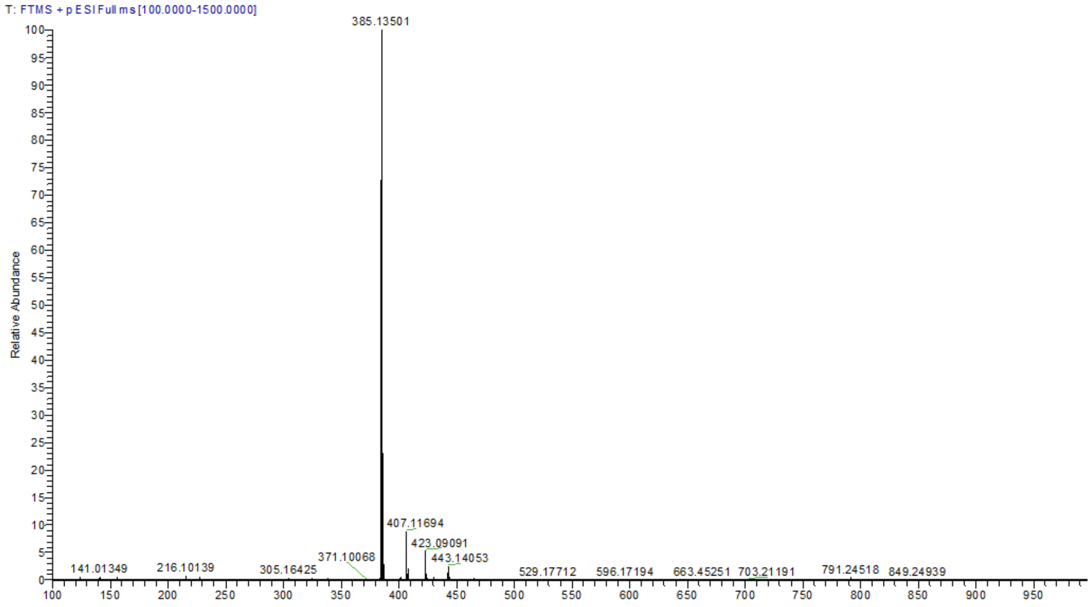

HRMS spectrum of **C28**

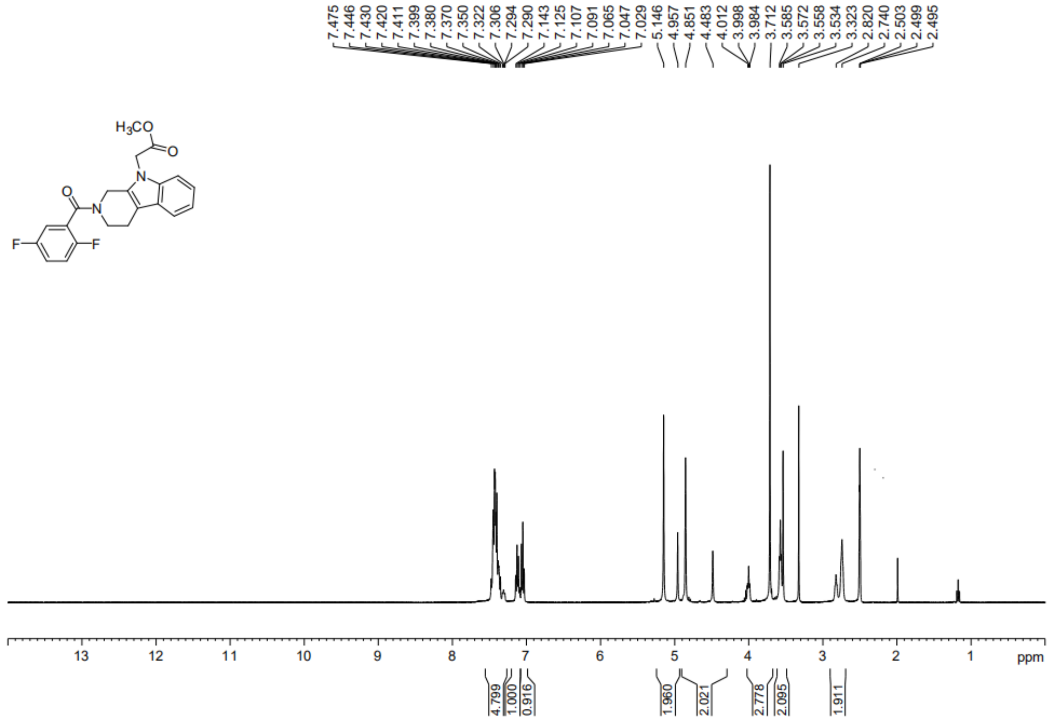

^{1}H NMR spectrum of **C28**

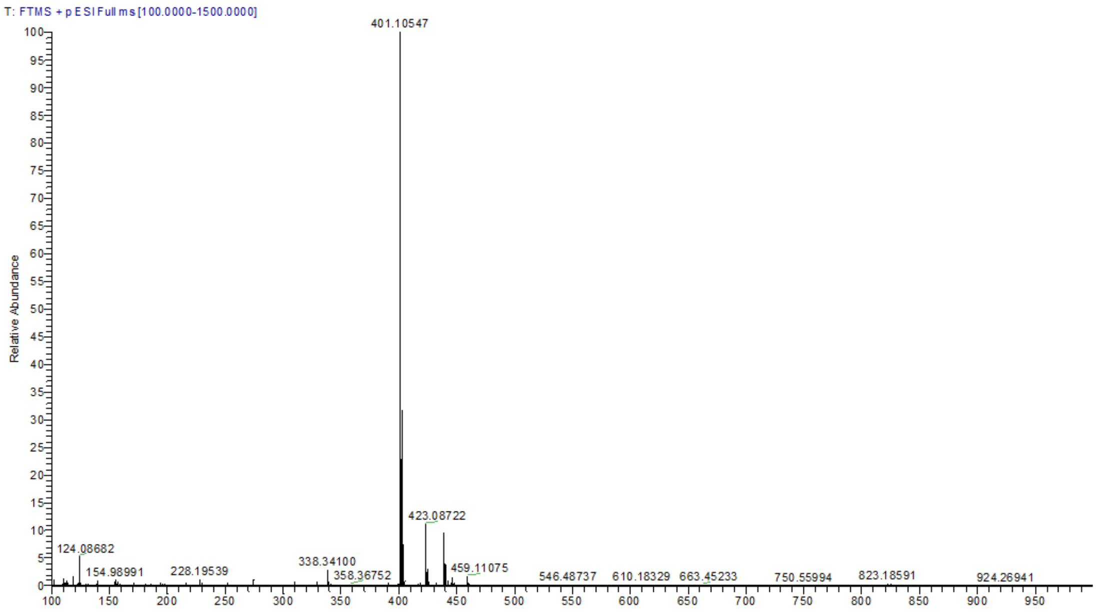

HRMS spectrum of **C29**

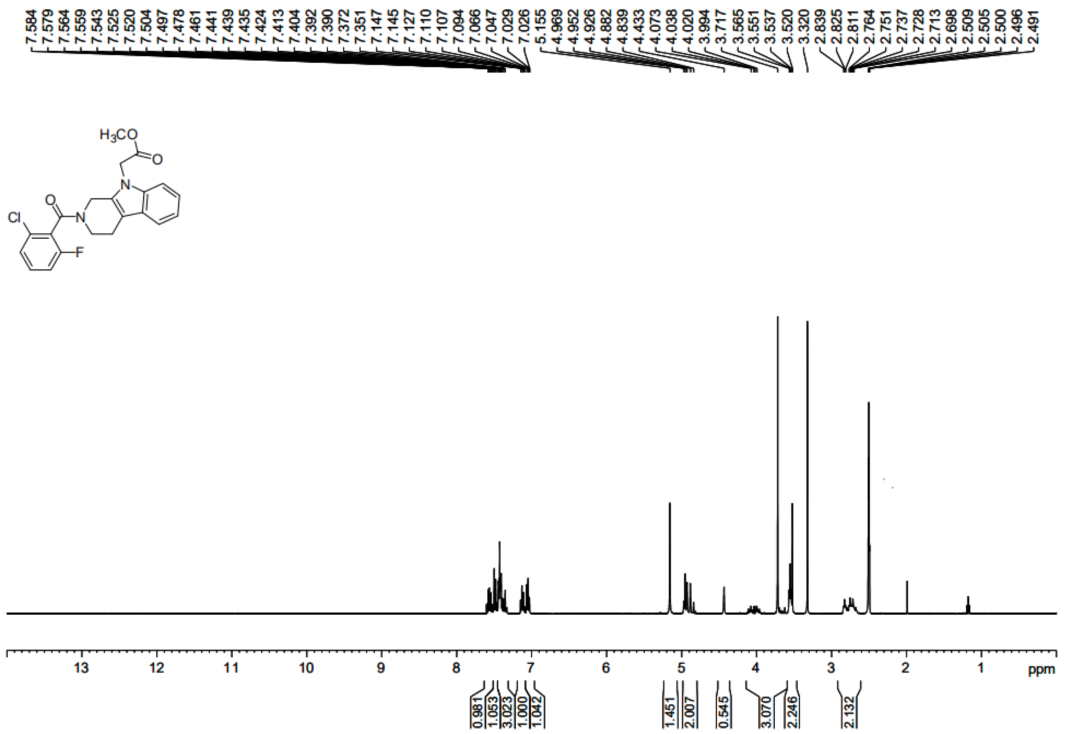

^{1}H NMR spectrum of **C29**

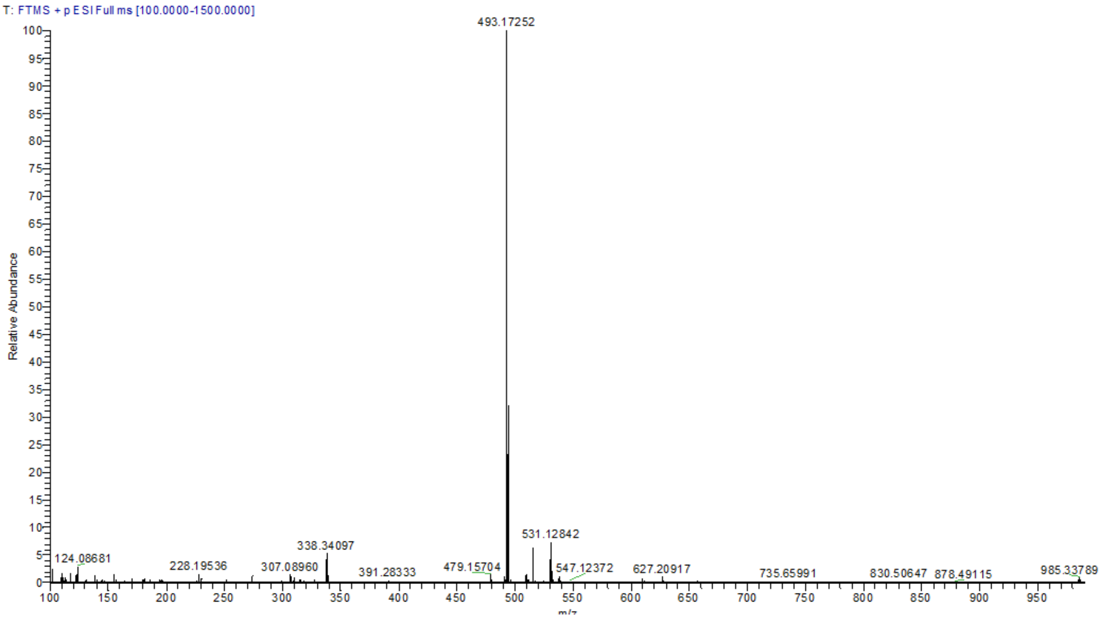

HRMS spectrum of **D1**

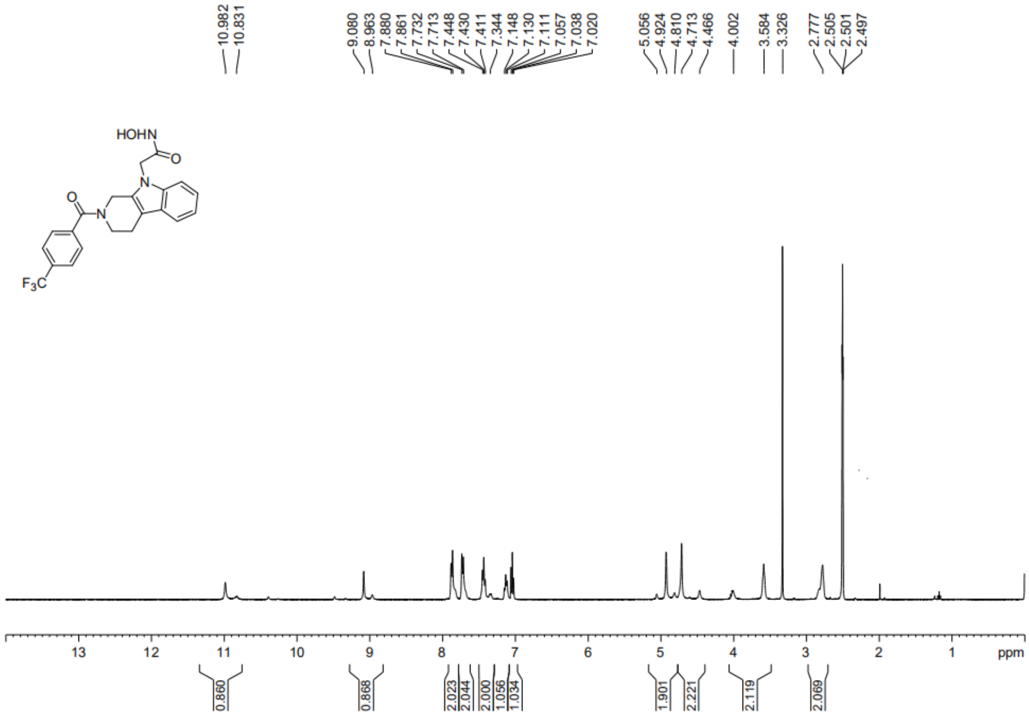

^{1}H NMR spectrum of **D1**

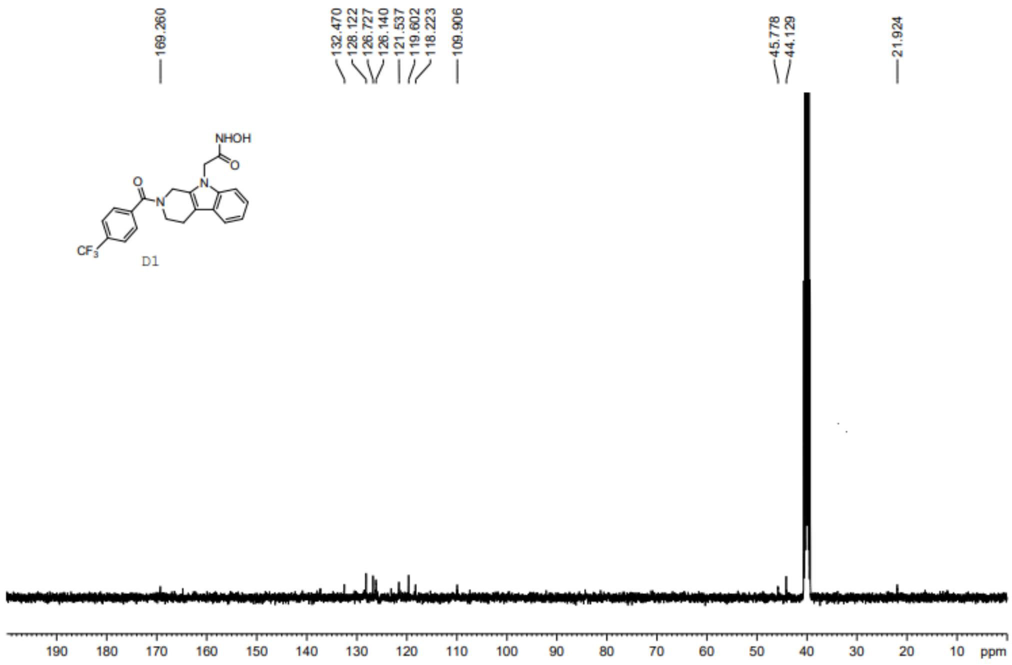

^{13}C NMR spectrum of **D1**

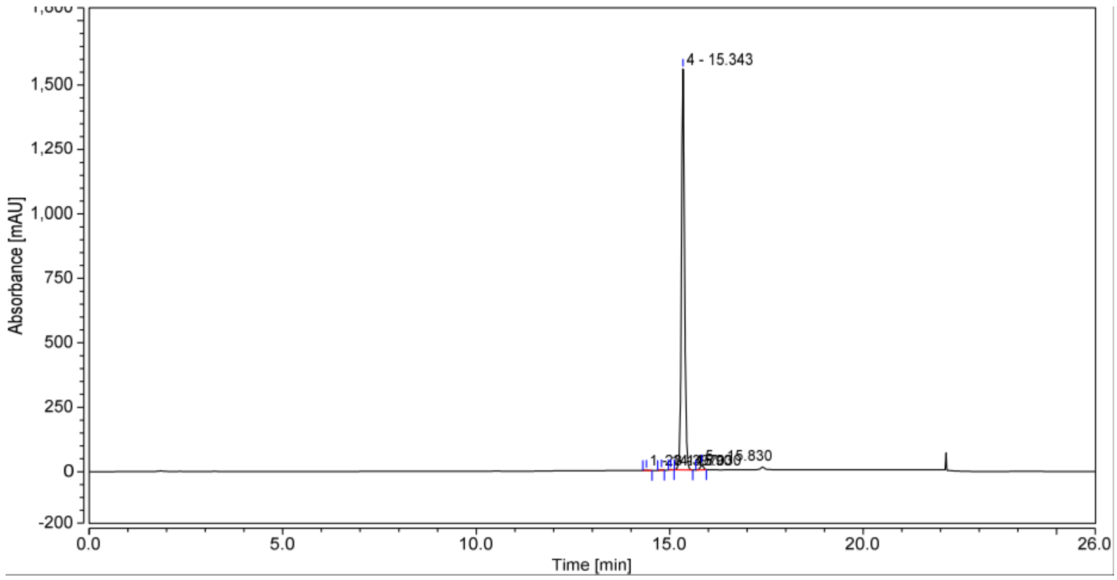

HPLC spectrum of **D1**

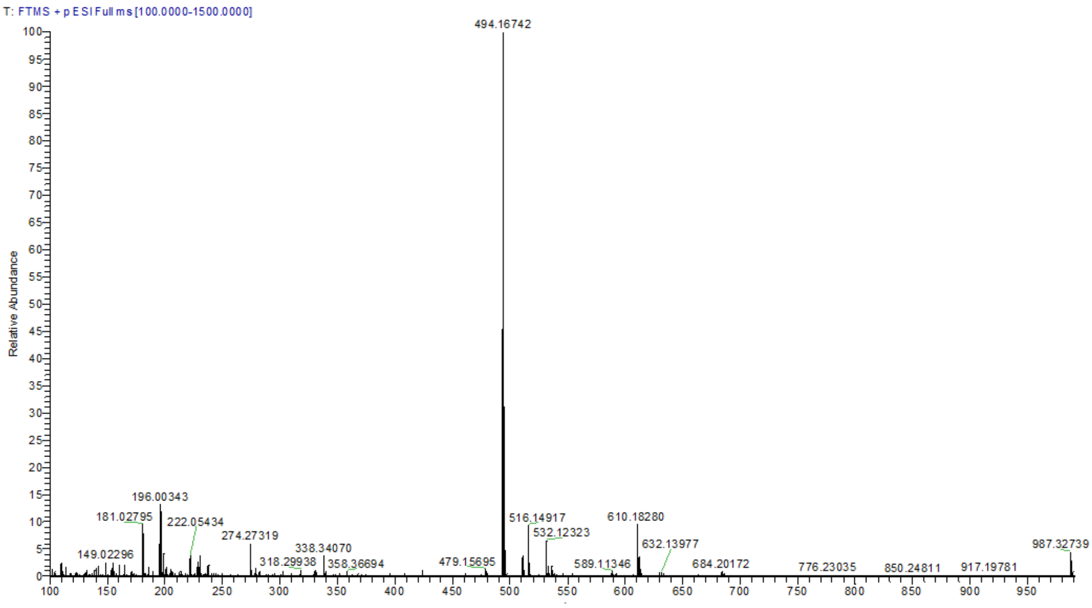

HRMS spectrum of **D2**

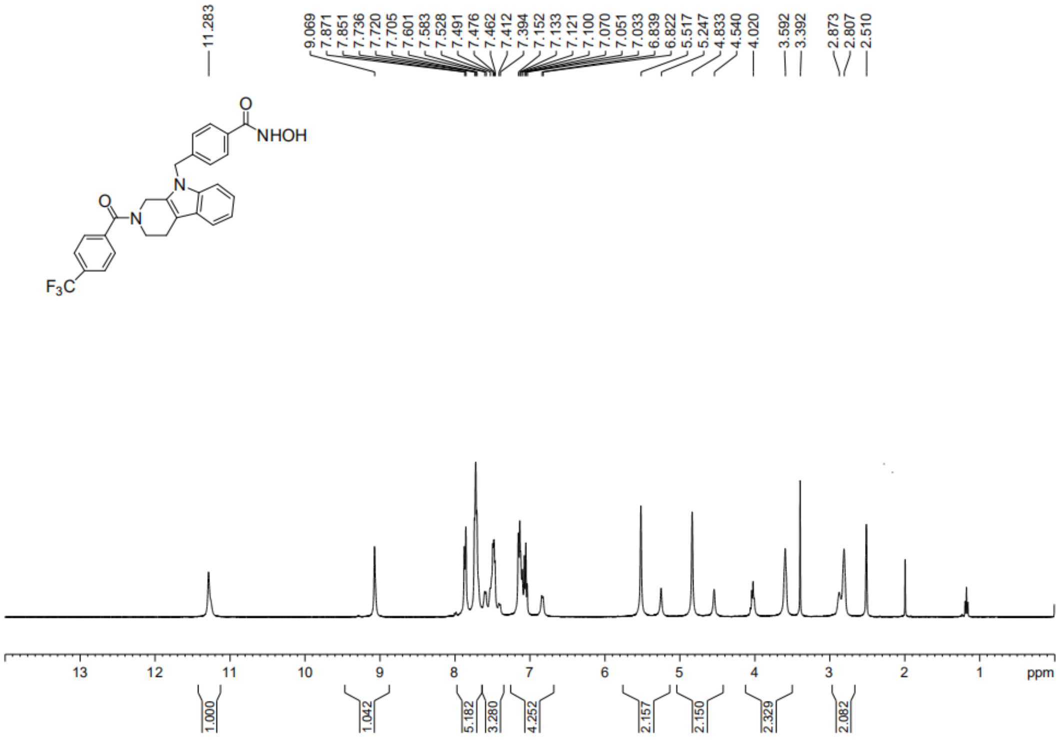

^{1}H NMR spectrum of **D2**

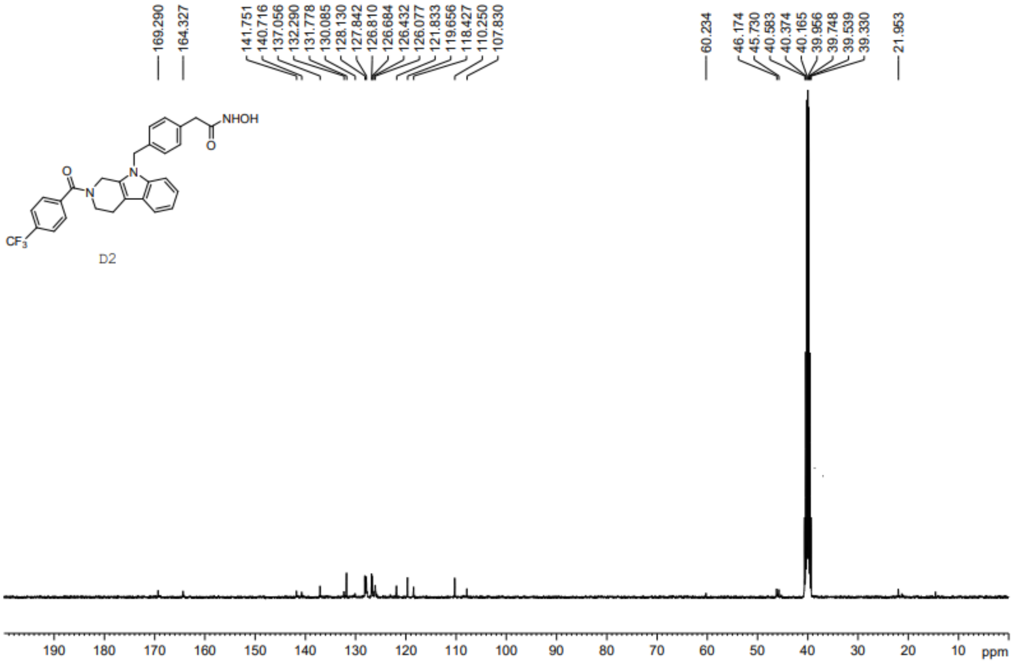

^{13}C NMR spectrum of **D2**

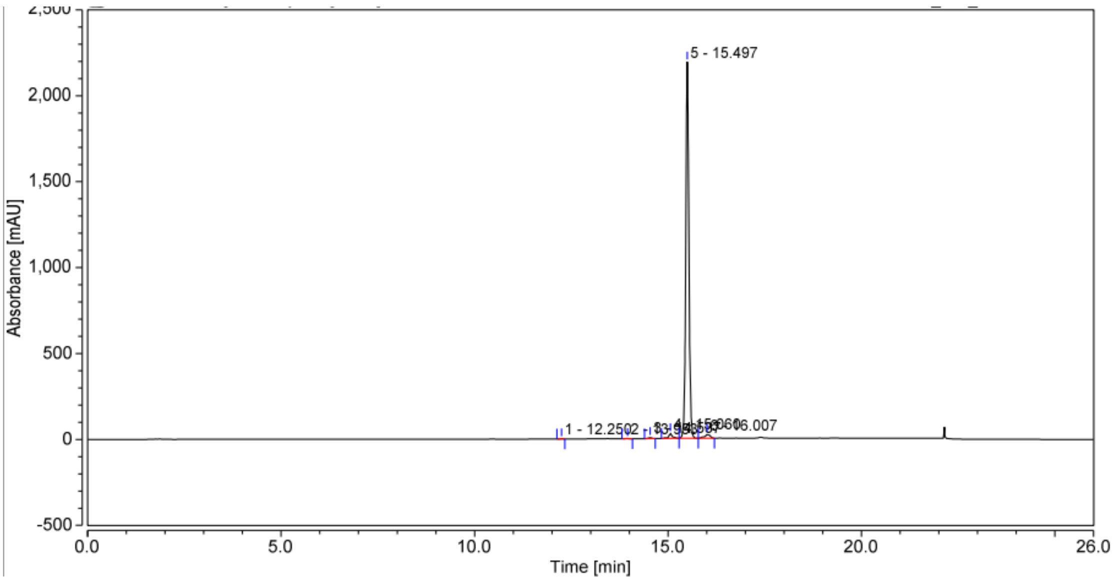

HPLC spectrum of **D2**

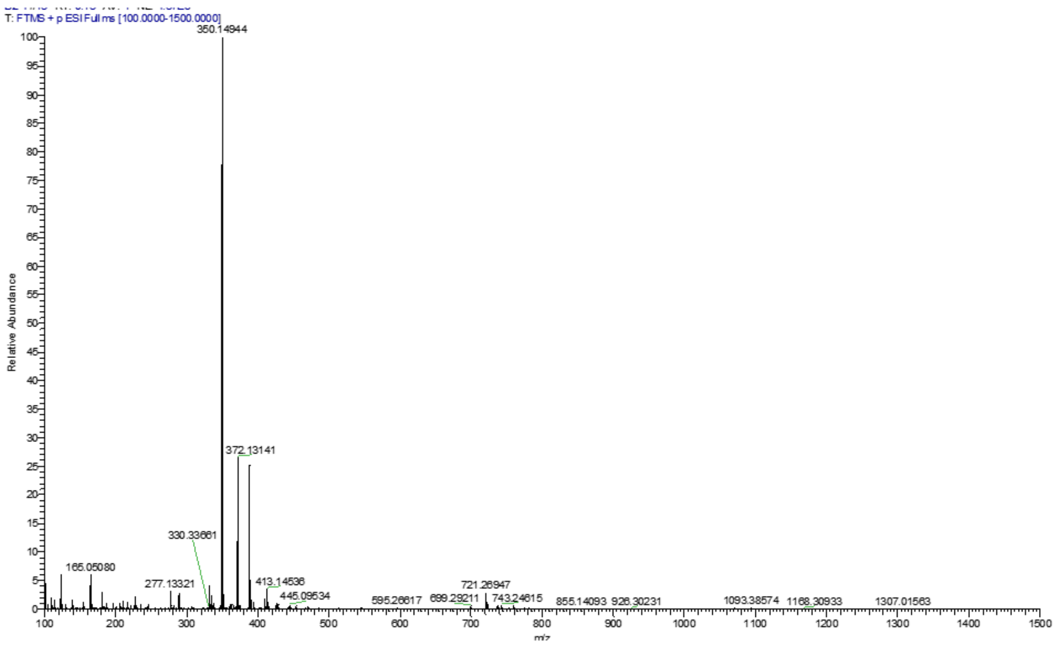

HRMS spectrum of **D3**

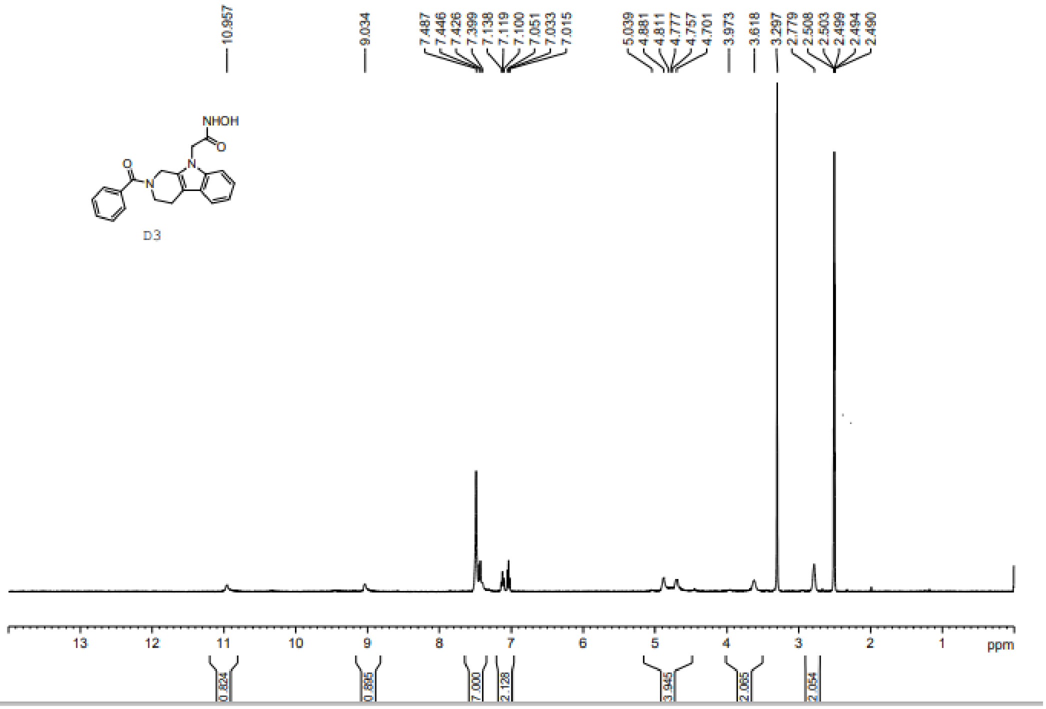

^{1}H NMR spectrum of **D3**

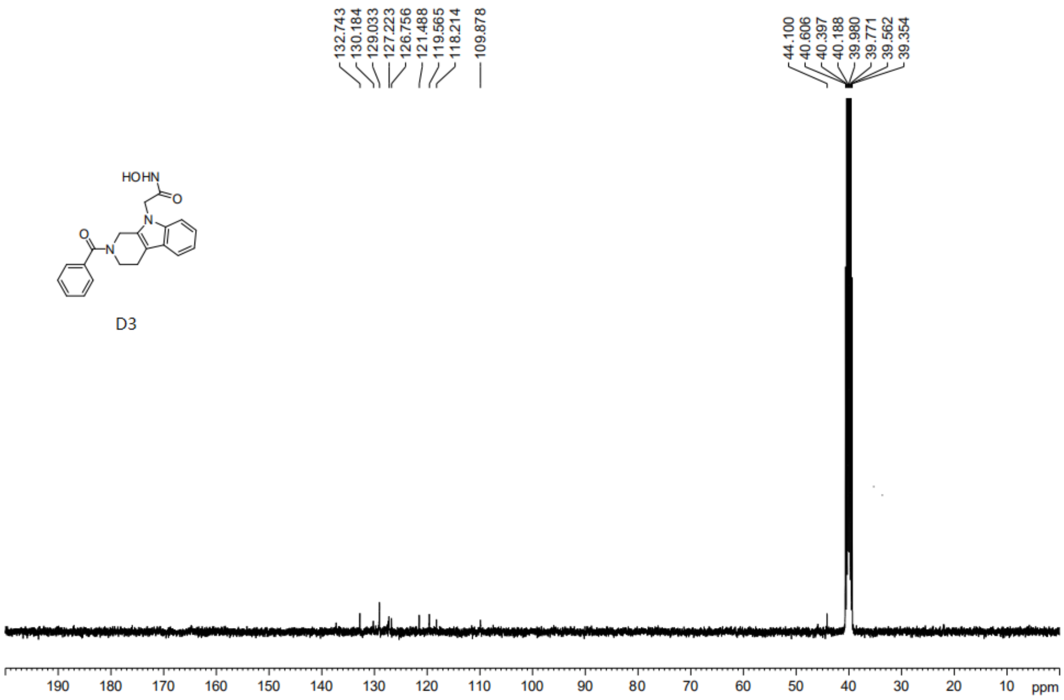

^{13}C NMR spectrum of **D3**

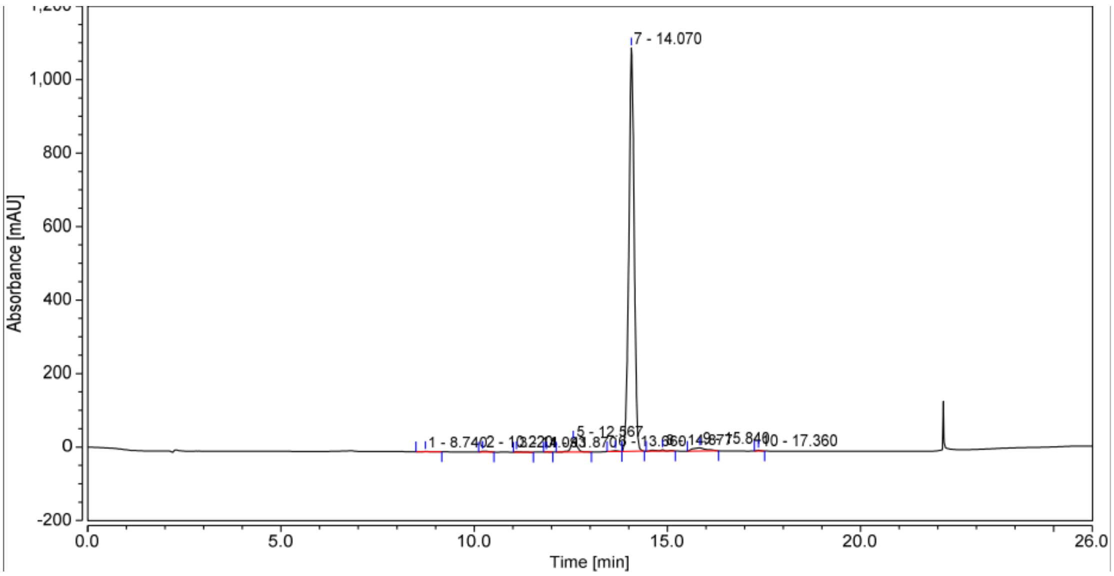

HPLC spectrum of **D3**

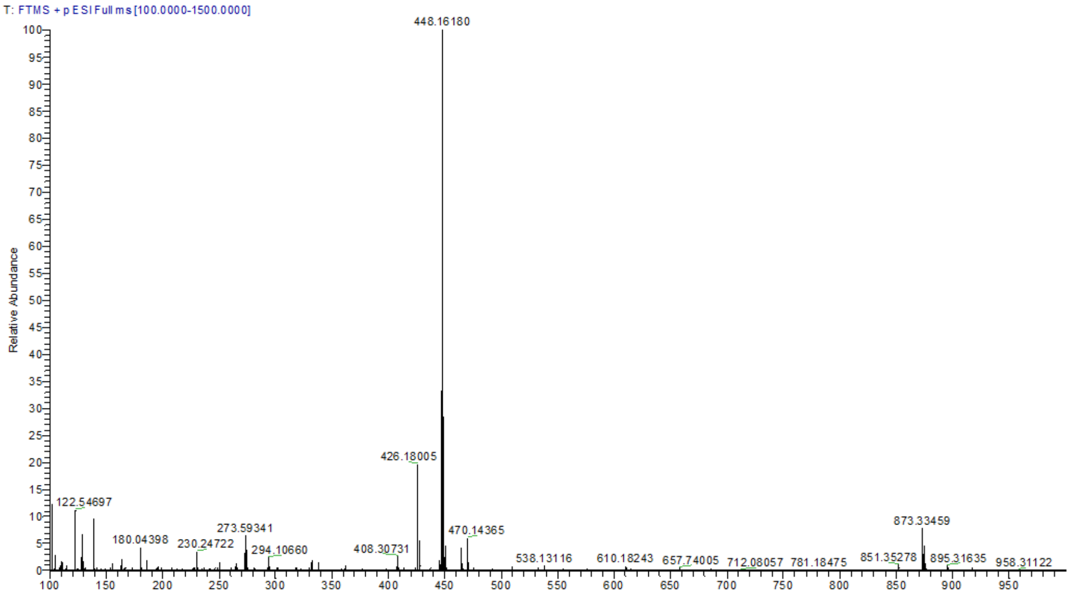

HRMS spectrum of **D4**

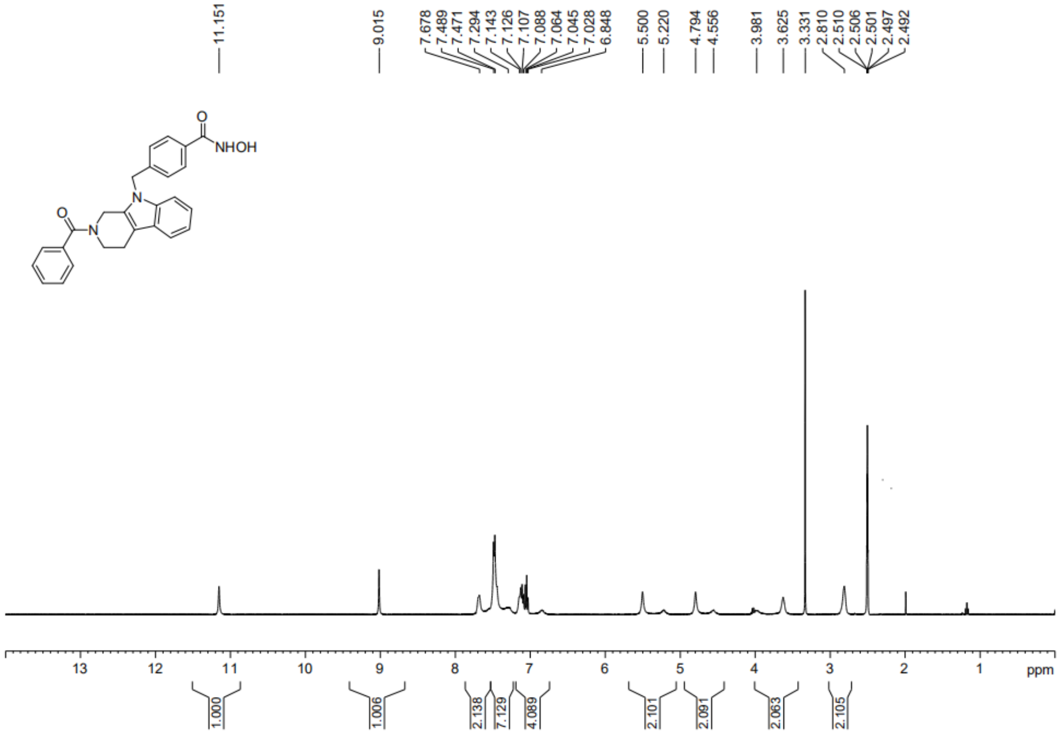

^{1}H NMR spectrum of **D4**

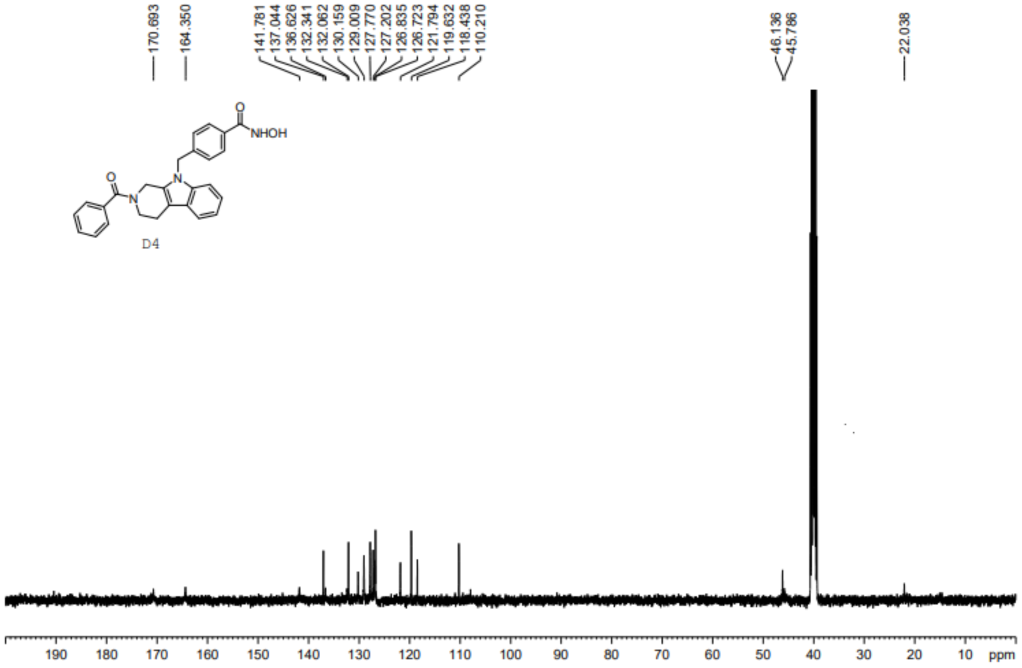

^{13}C NMR spectrum of **D4**

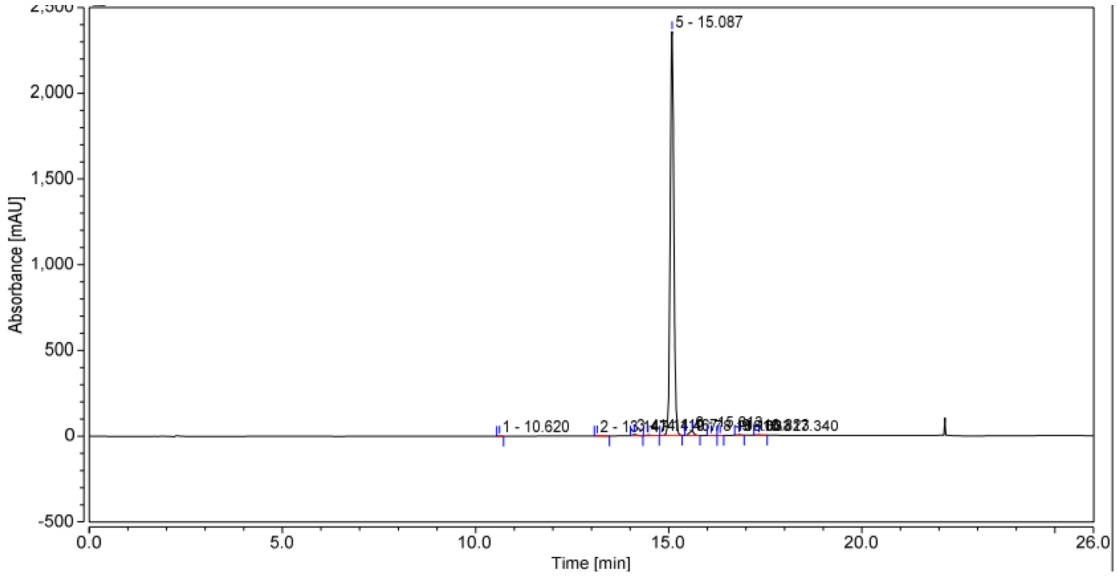

HPLC spectrum of **D4**

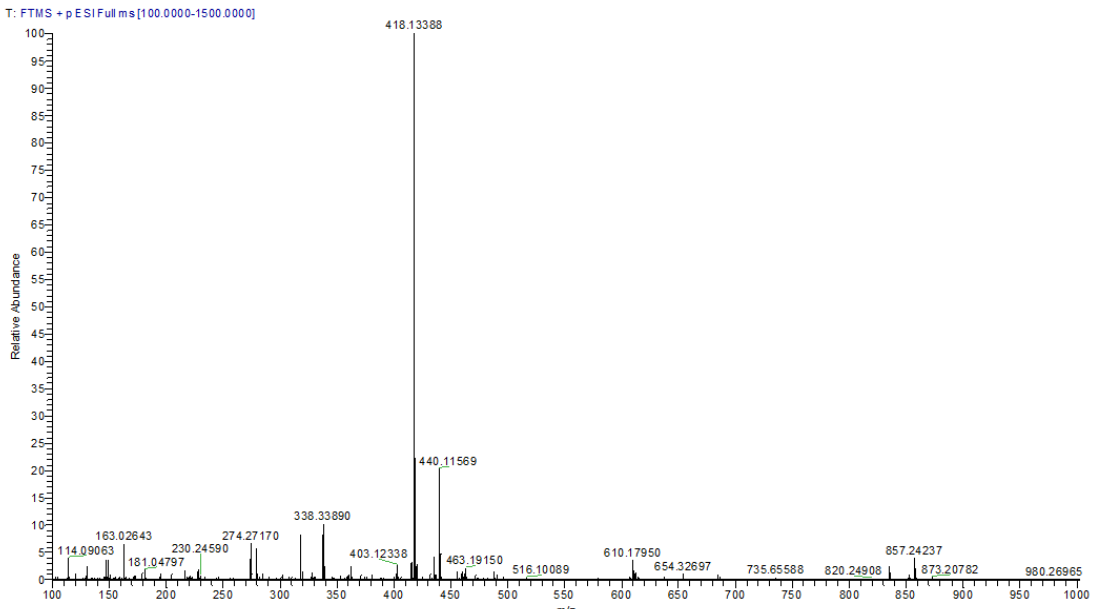

HRMS spectrum of **D5**

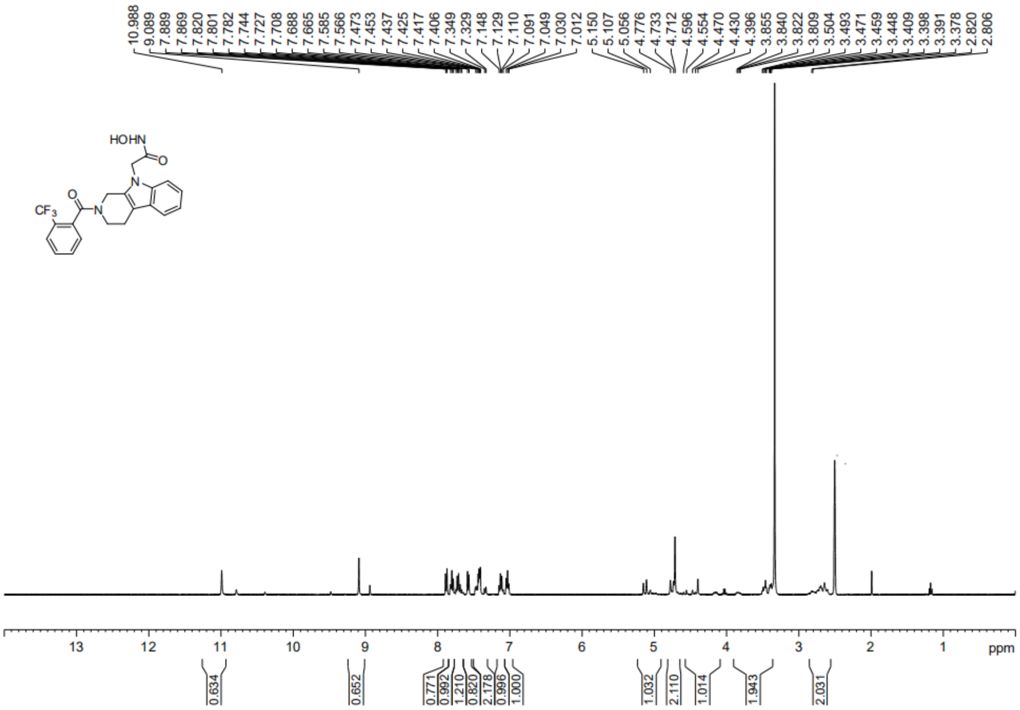

^{1}H NMR spectrum of **D5**

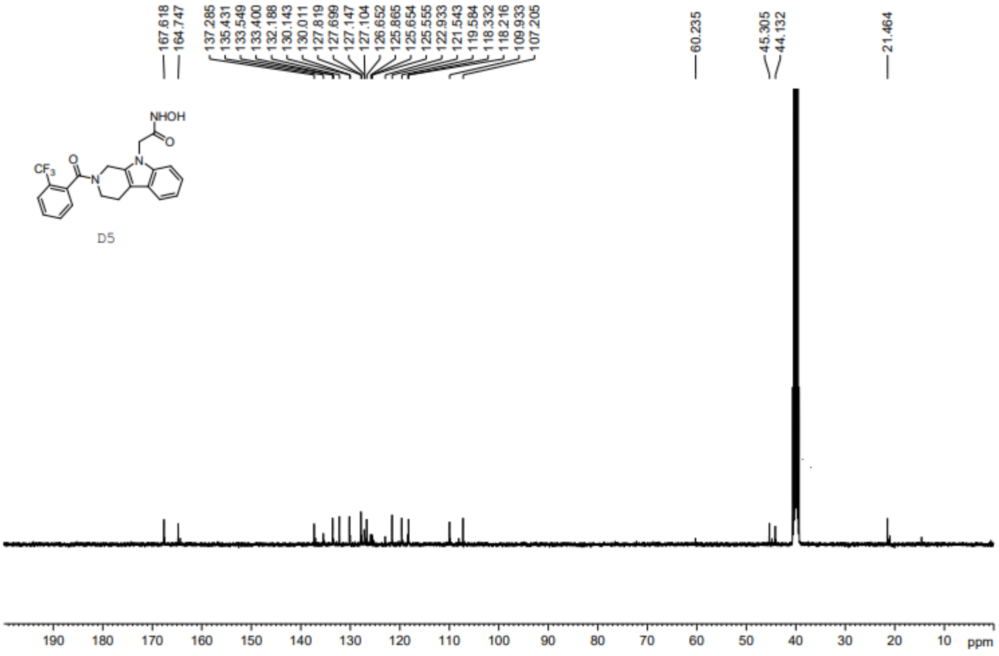

^{13}C NMR spectrum of **D5**

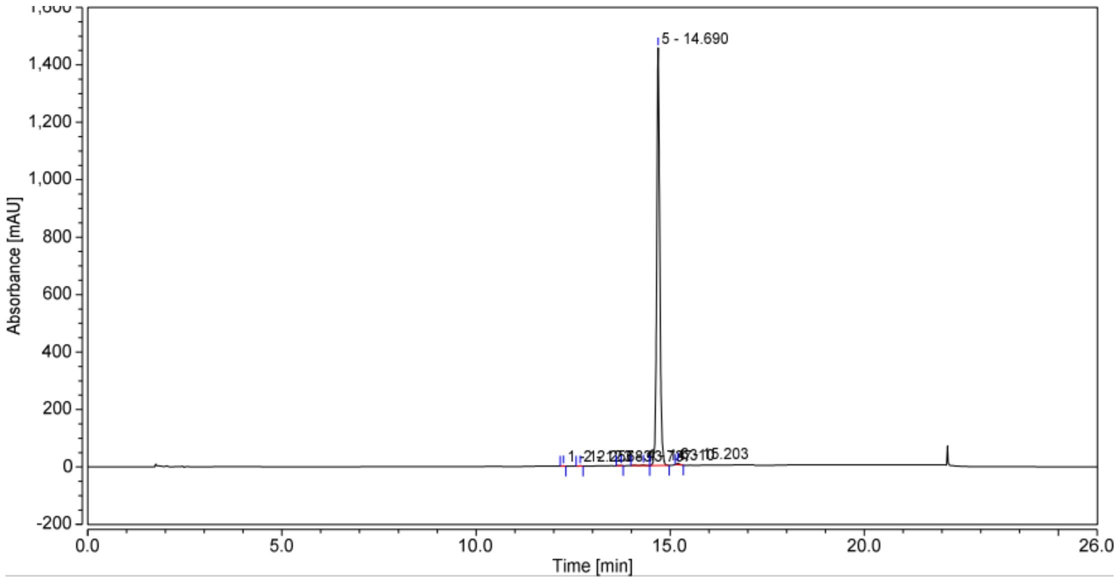

HPLC spectrum of **D5**

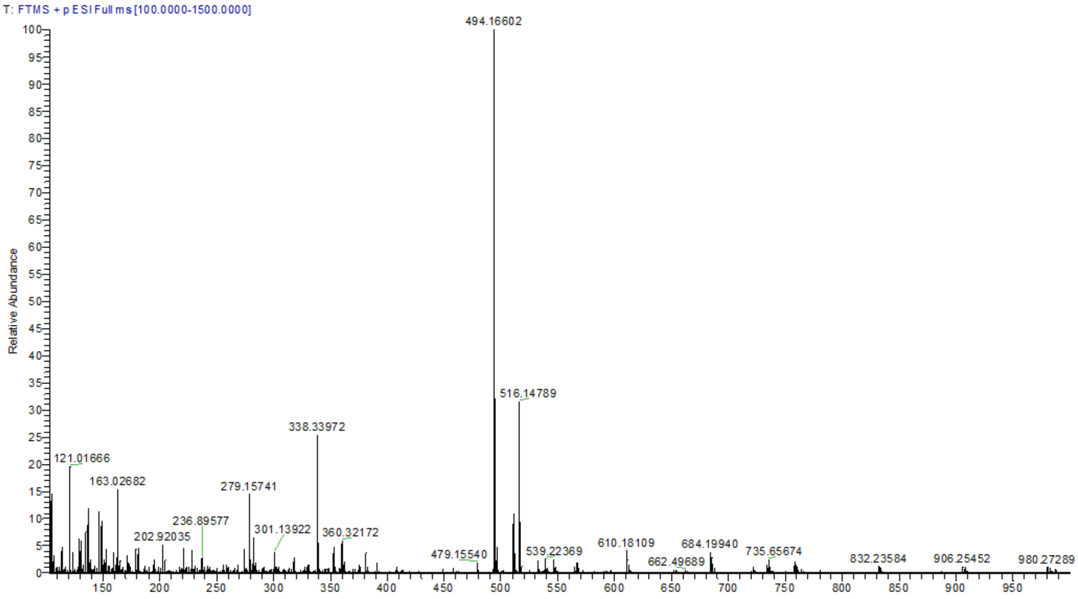

HRMS spectrum of **D6**

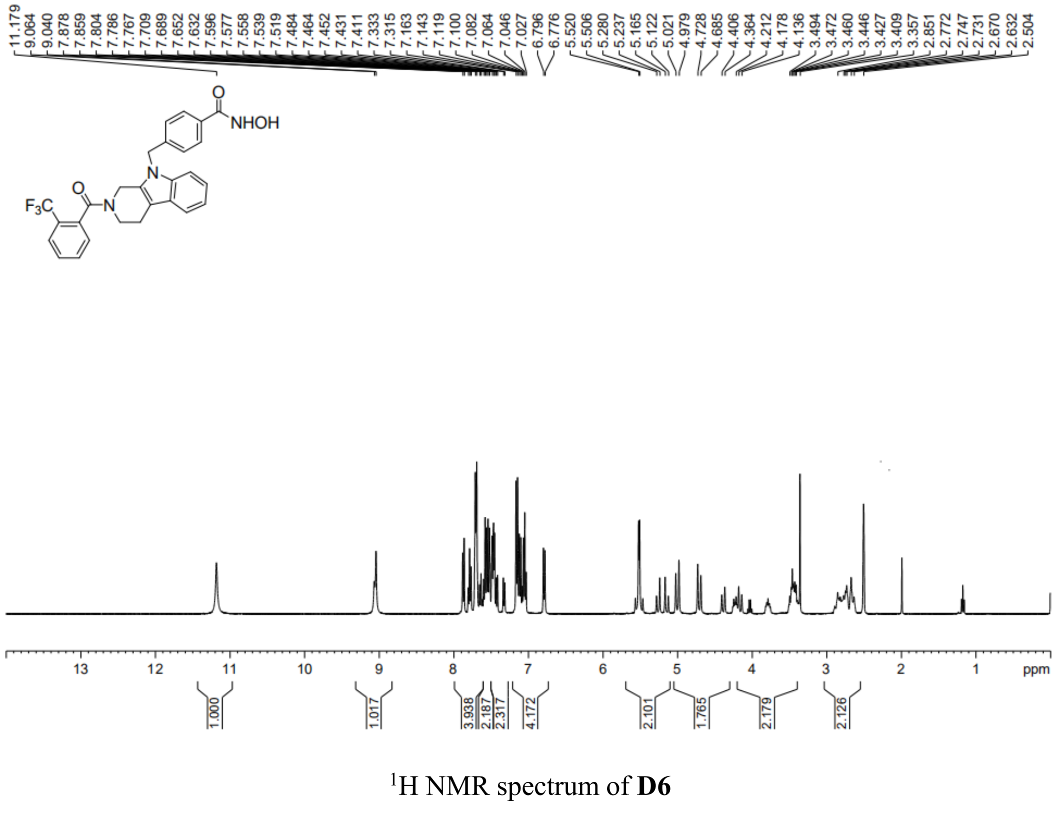

^{1}H NMR spectrum of **D6**

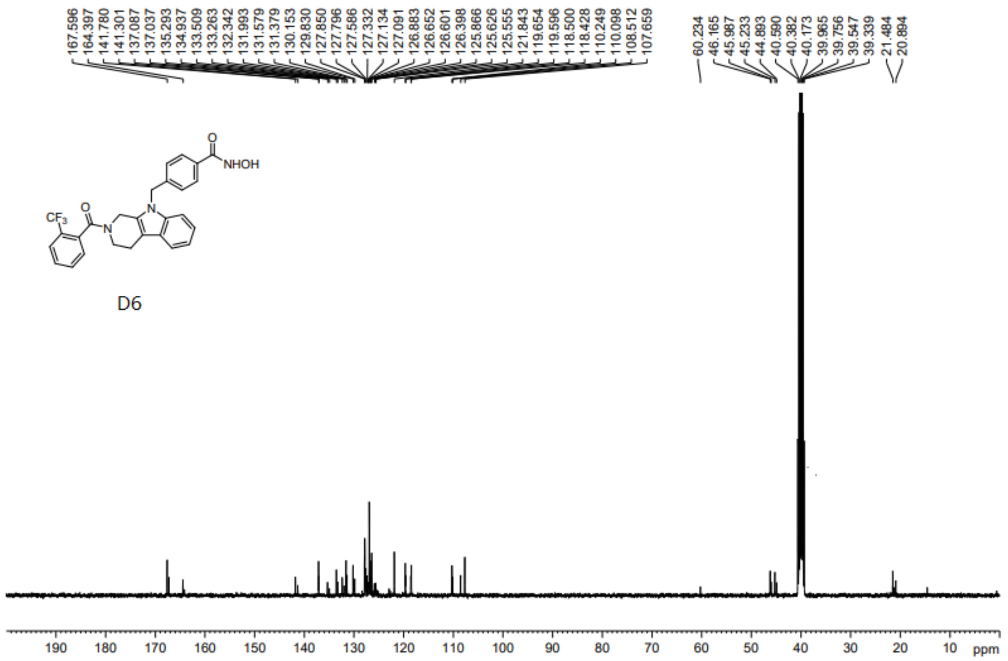

^{13}C NMR spectrum of **D6**

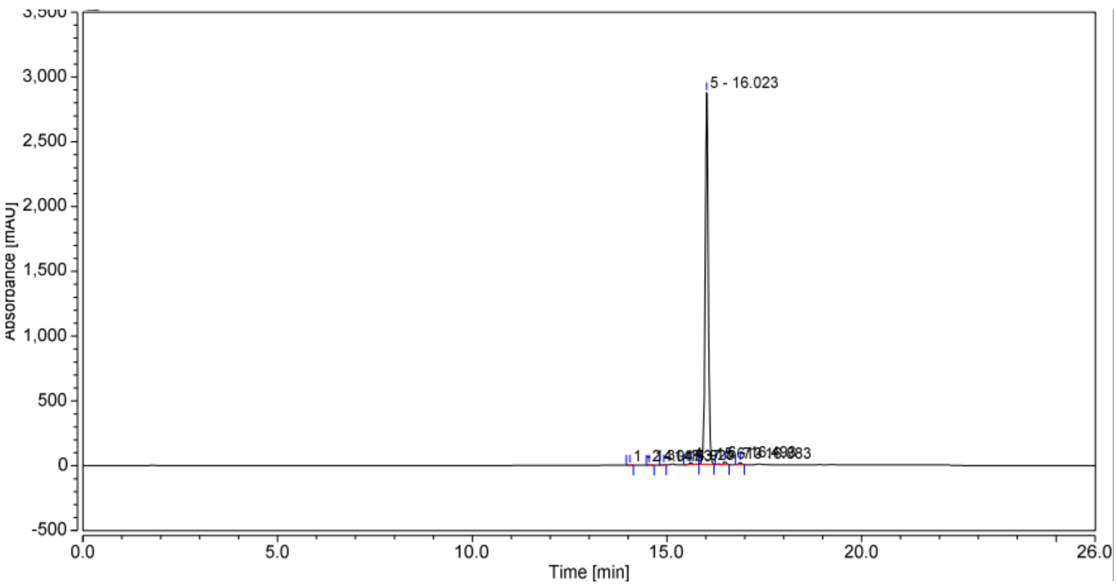

HPLC spectrum of **D6**

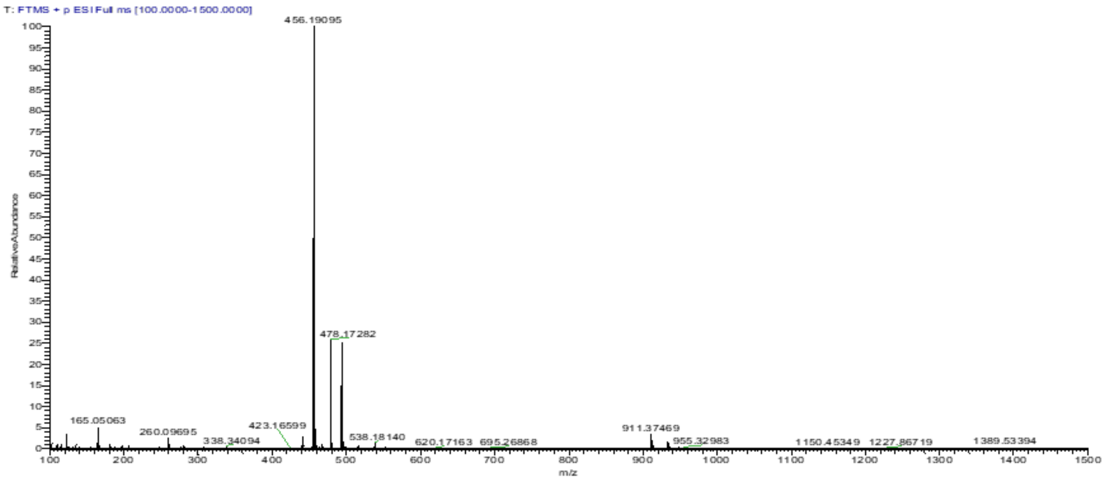

HRMS spectrum of **D7**

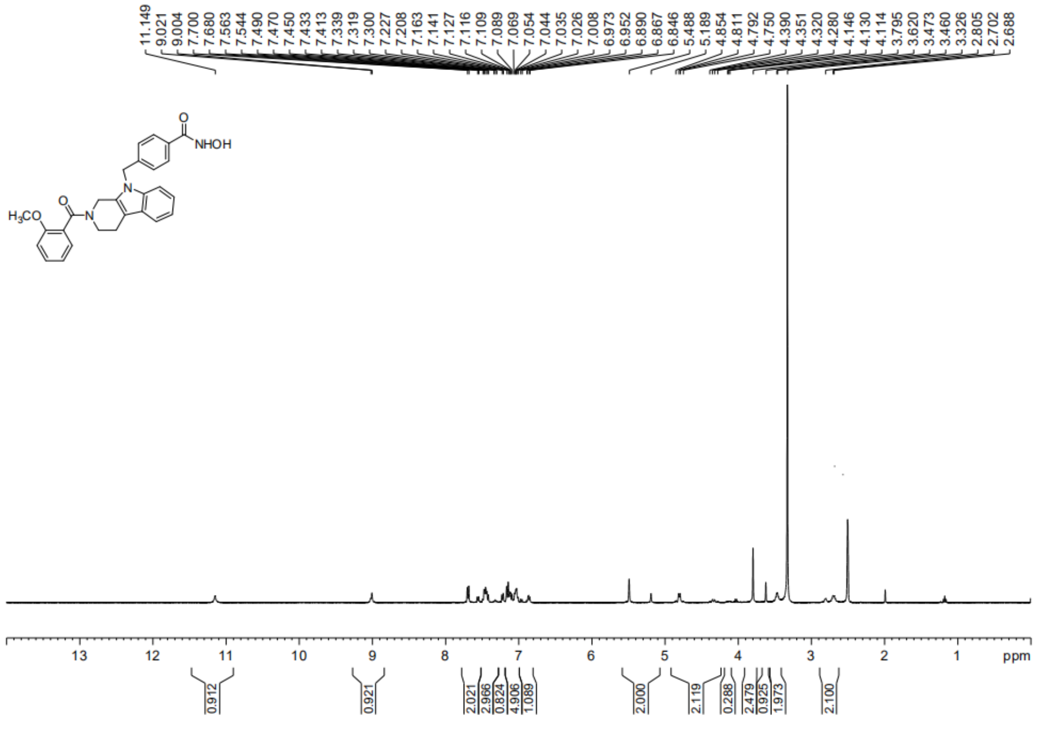

^{1}H NMR spectrum of **D7**

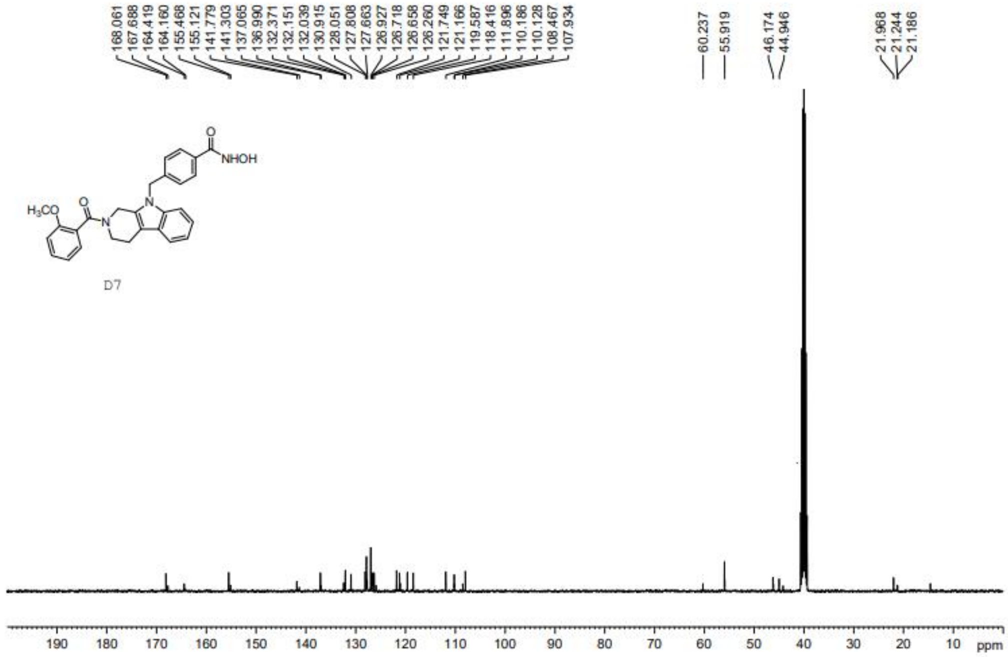

^{13}C NMR spectrum of **D7**

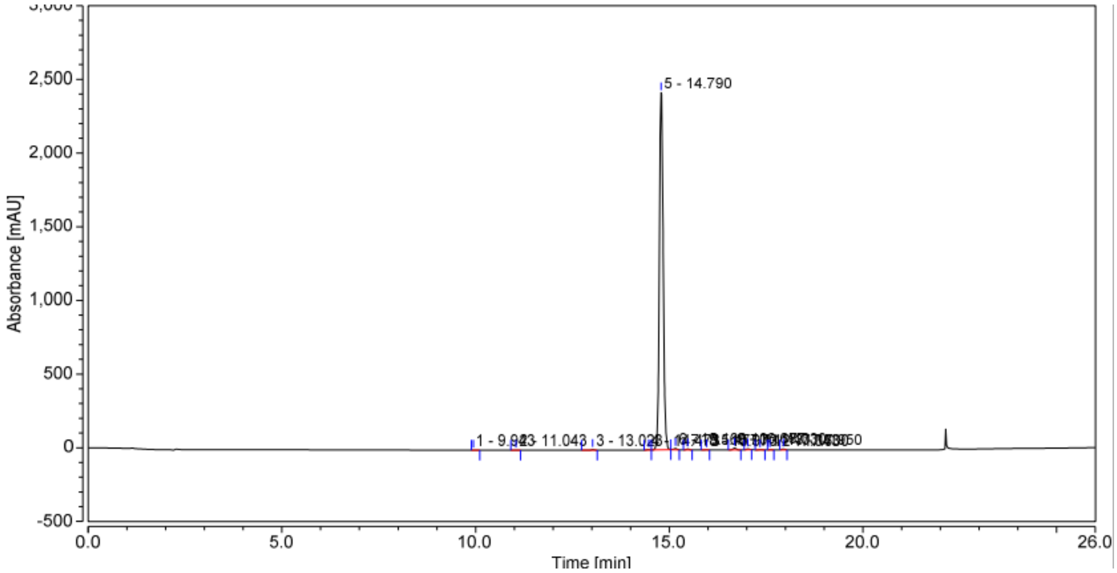

HPLC spectrum of **D7**

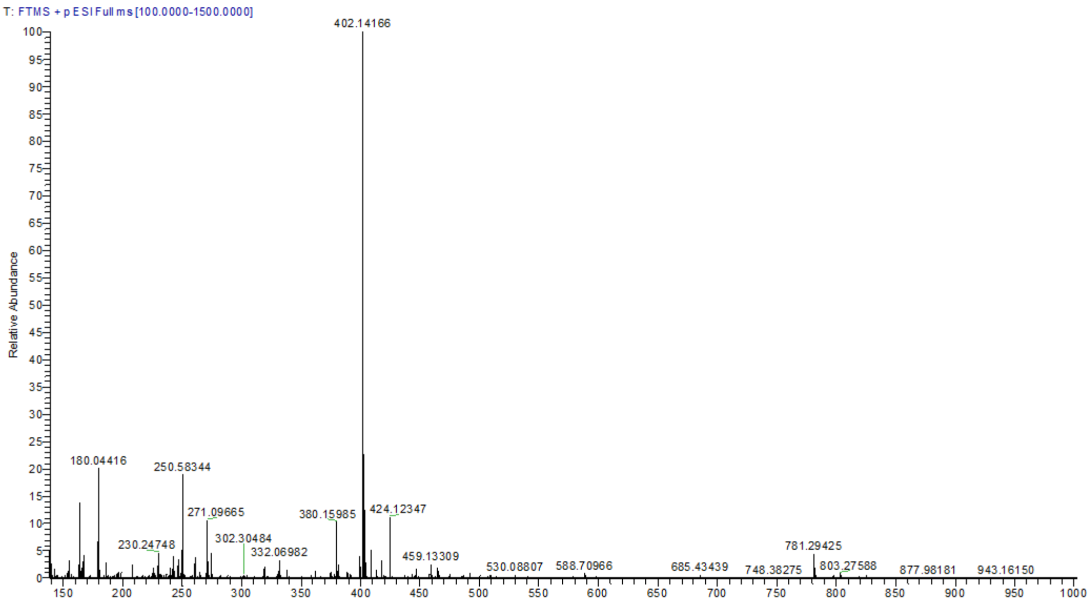

HRMS spectrum of **D8**

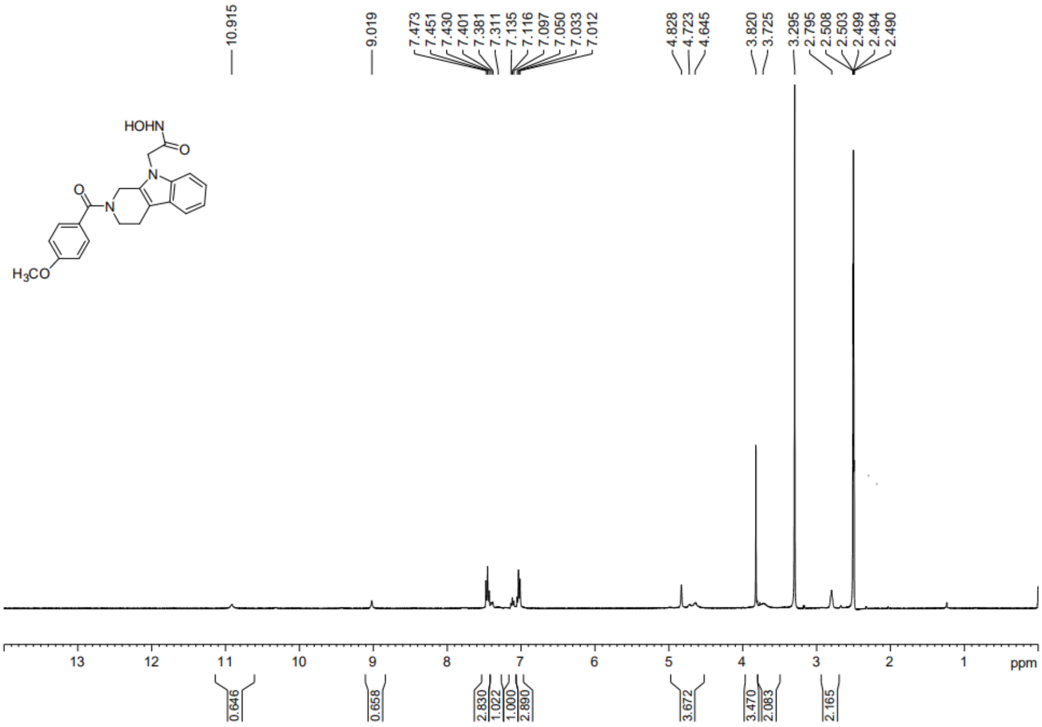

^{1}H NMR spectrum of **D8**

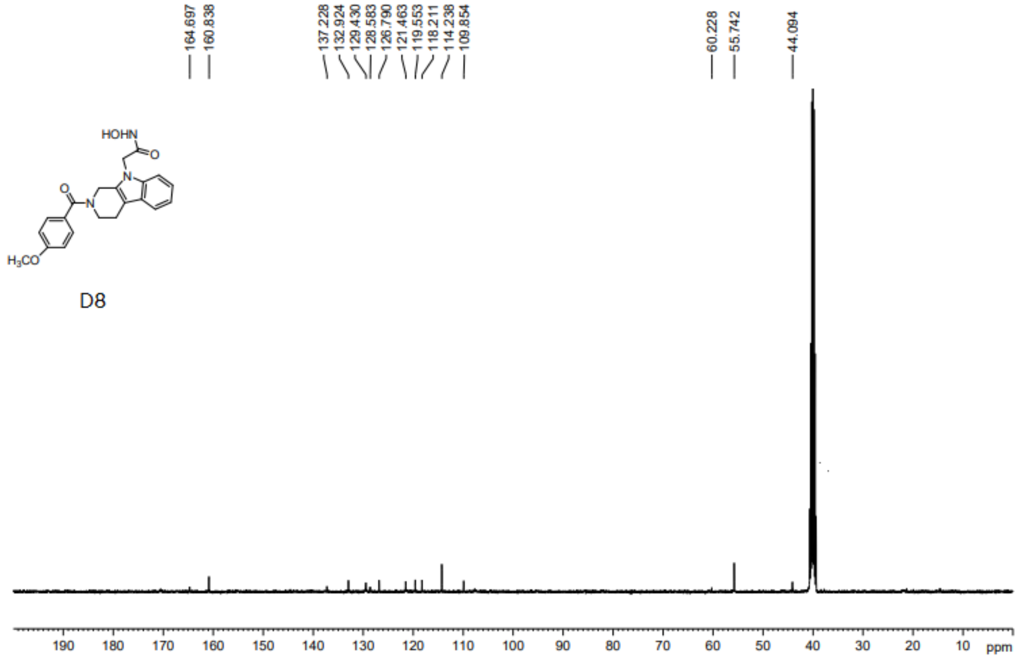

^{13}C NMR spectrum of **D8**

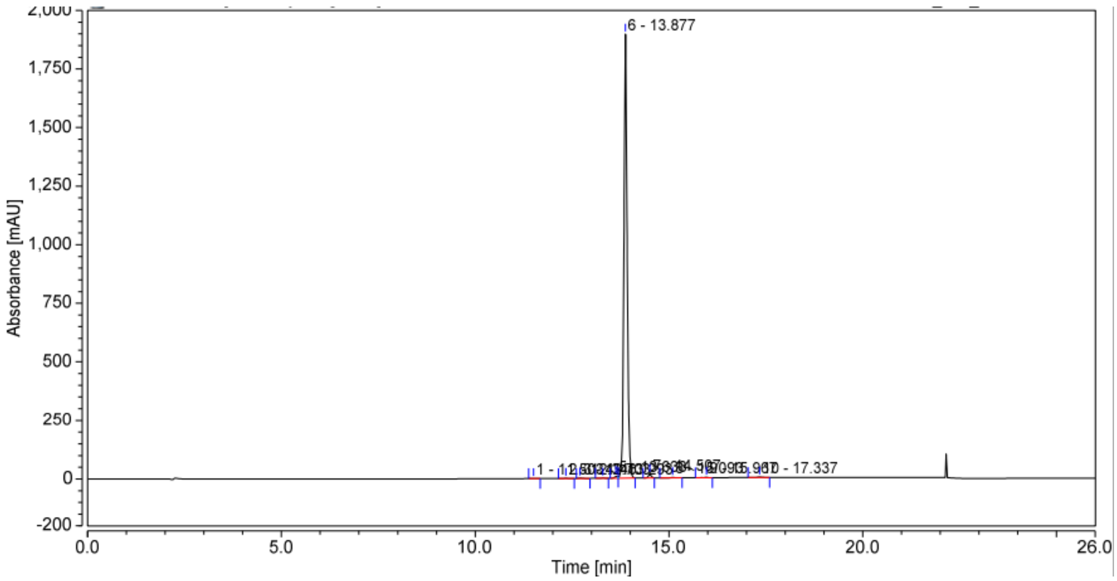

HPLC spectrum of **D8**

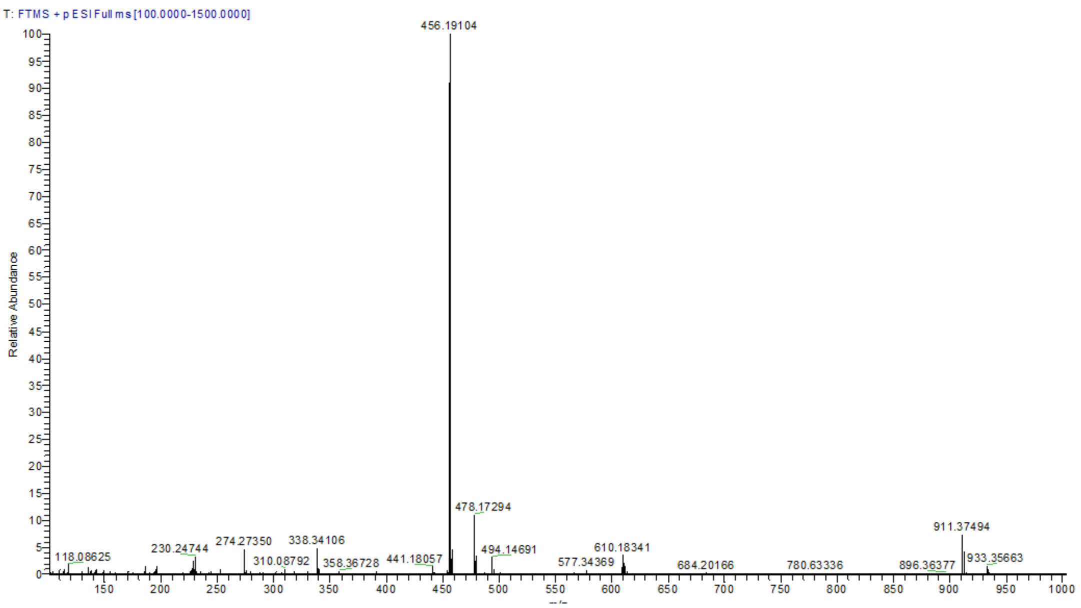

HRMS spectrum of **D9**

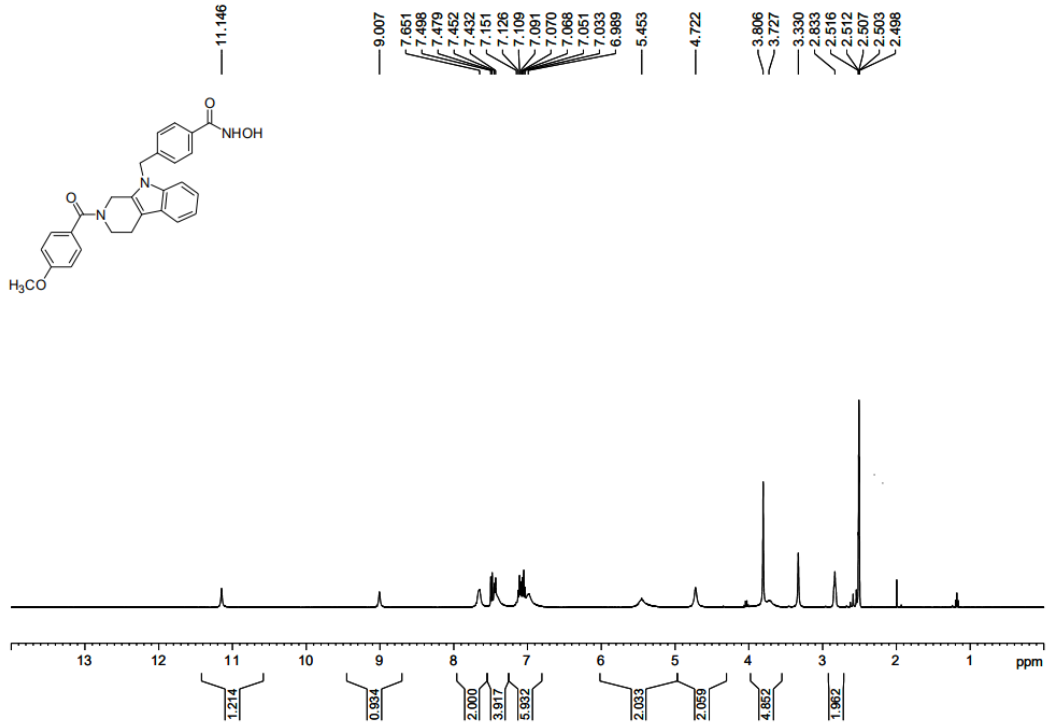

^{1}H NMR spectrum of **D9**

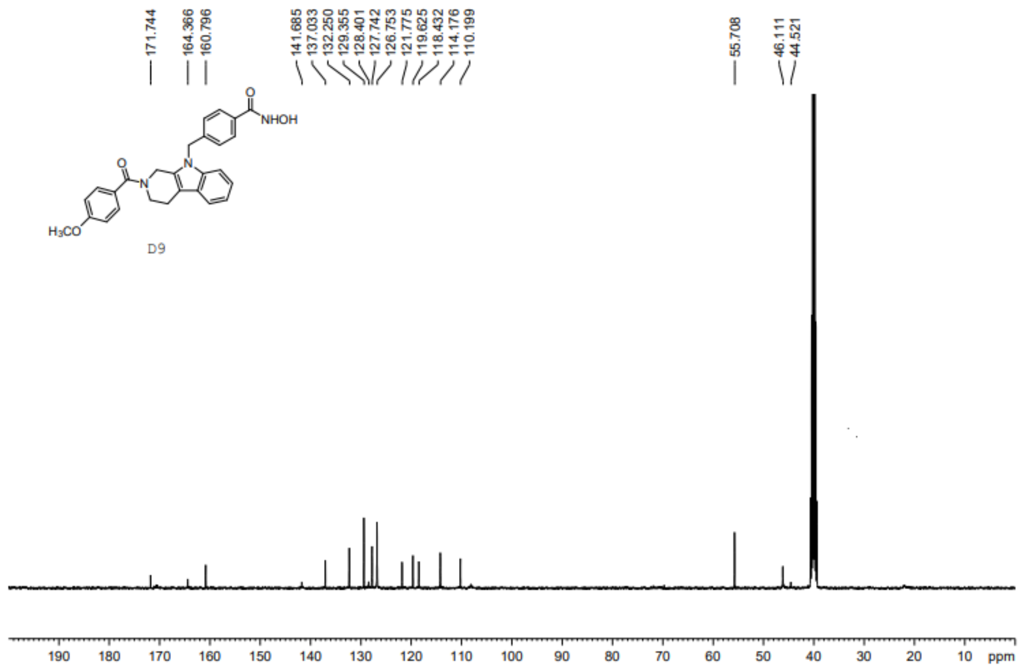

^{13}C NMR spectrum of **D9**

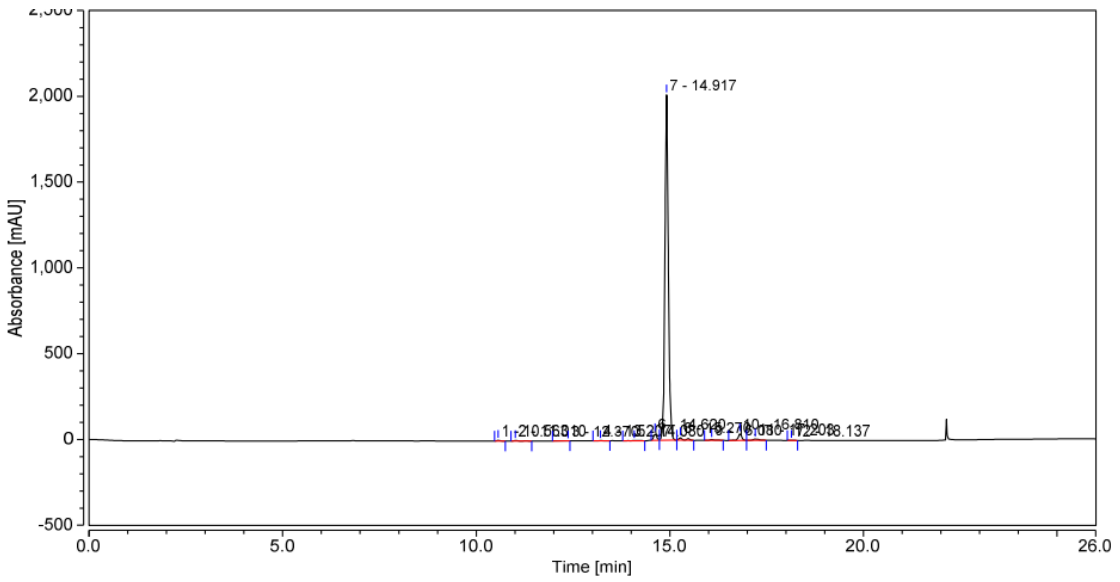

HPLC spectrum of **D9**

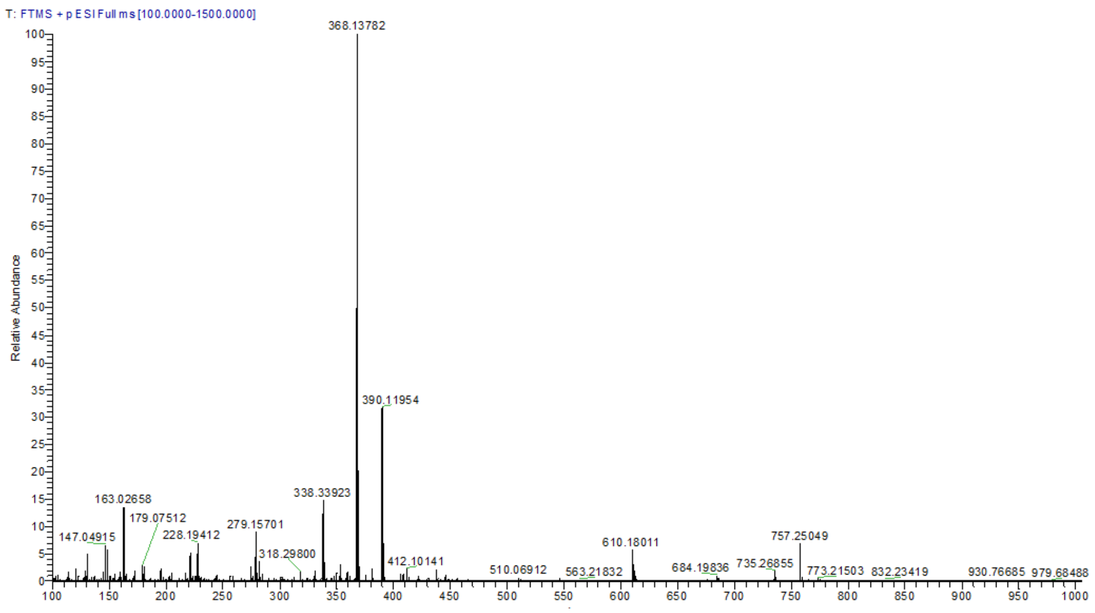

HRMS spectrum of **D10**

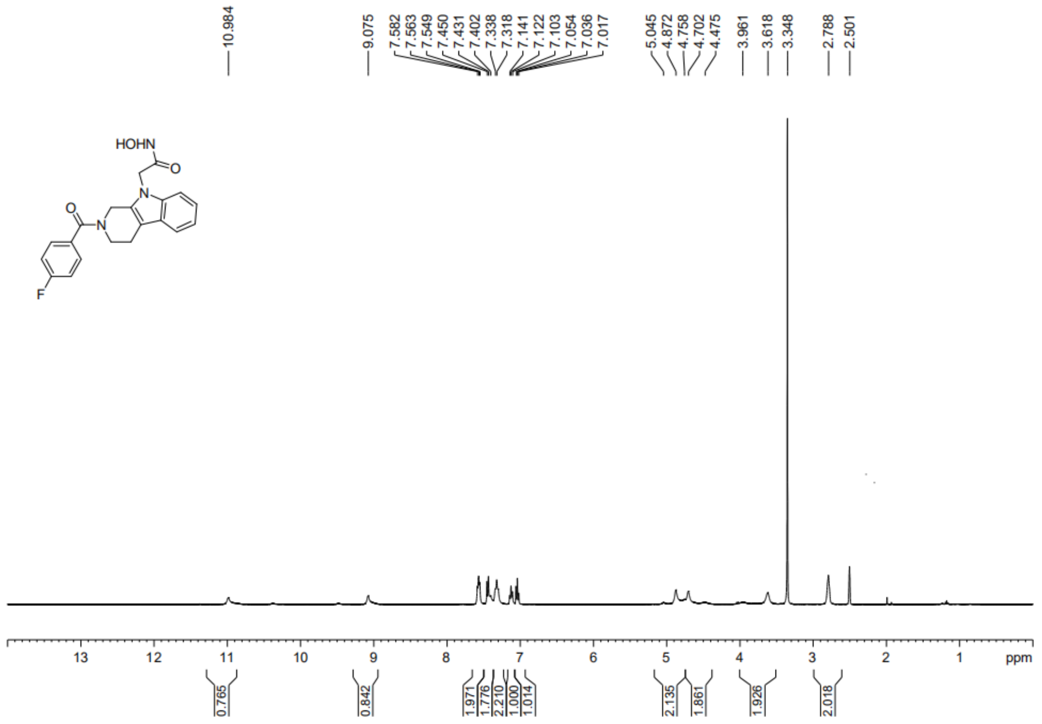

^{1}H NMR spectrum of **D10**

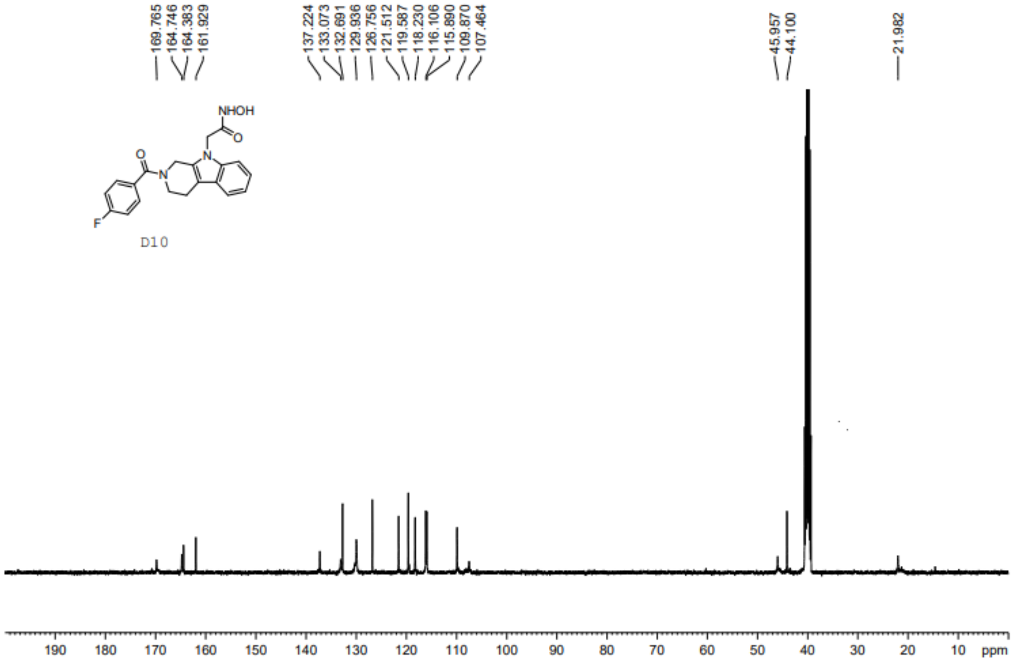

^{13}C NMR spectrum of **D10**

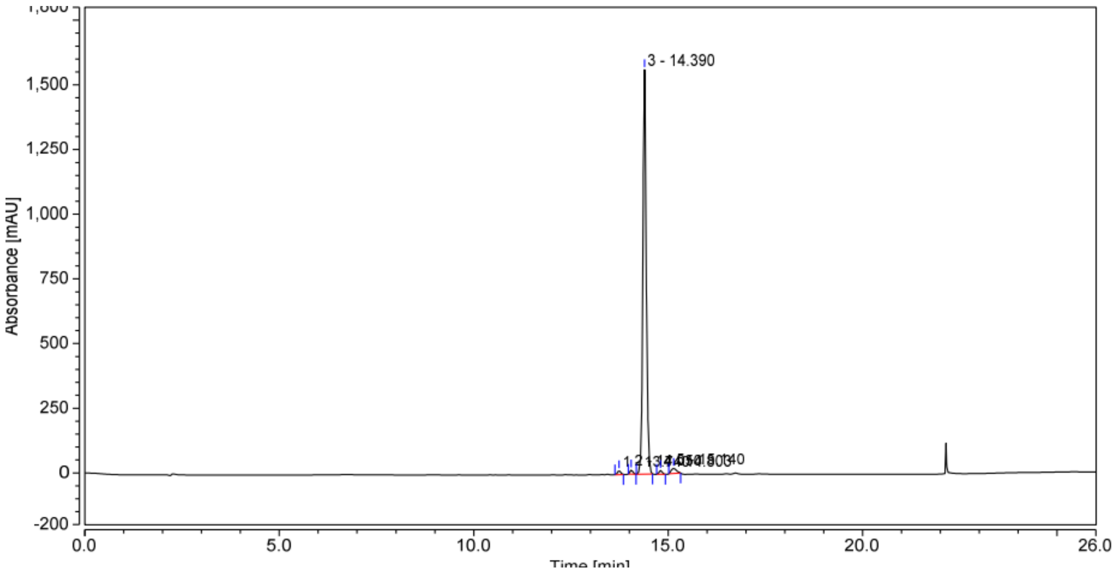

HPLC spectrum of **D10**

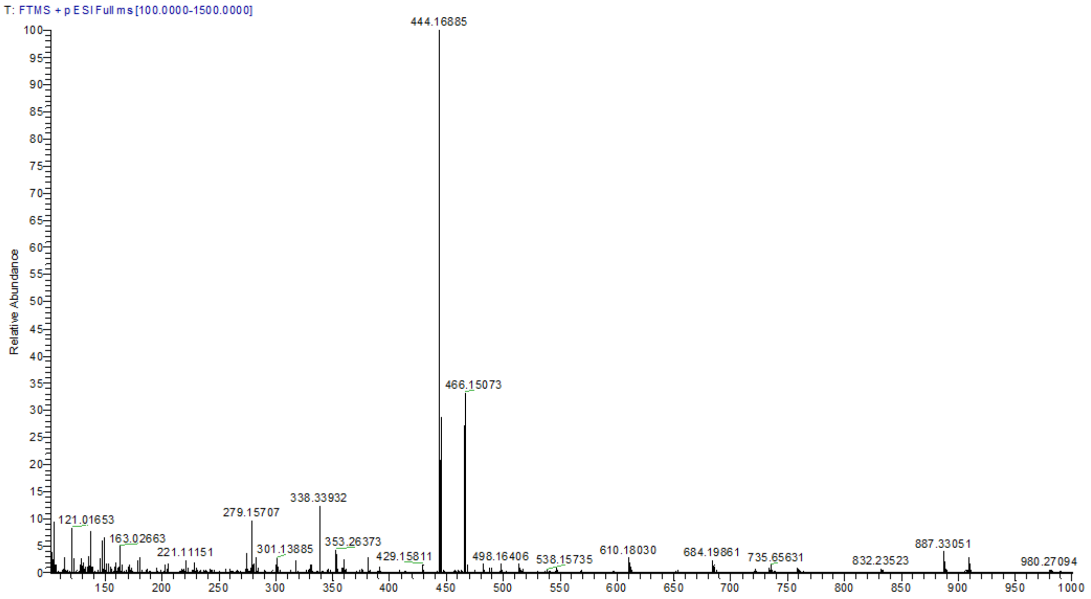

HRMS spectrum of **D11**

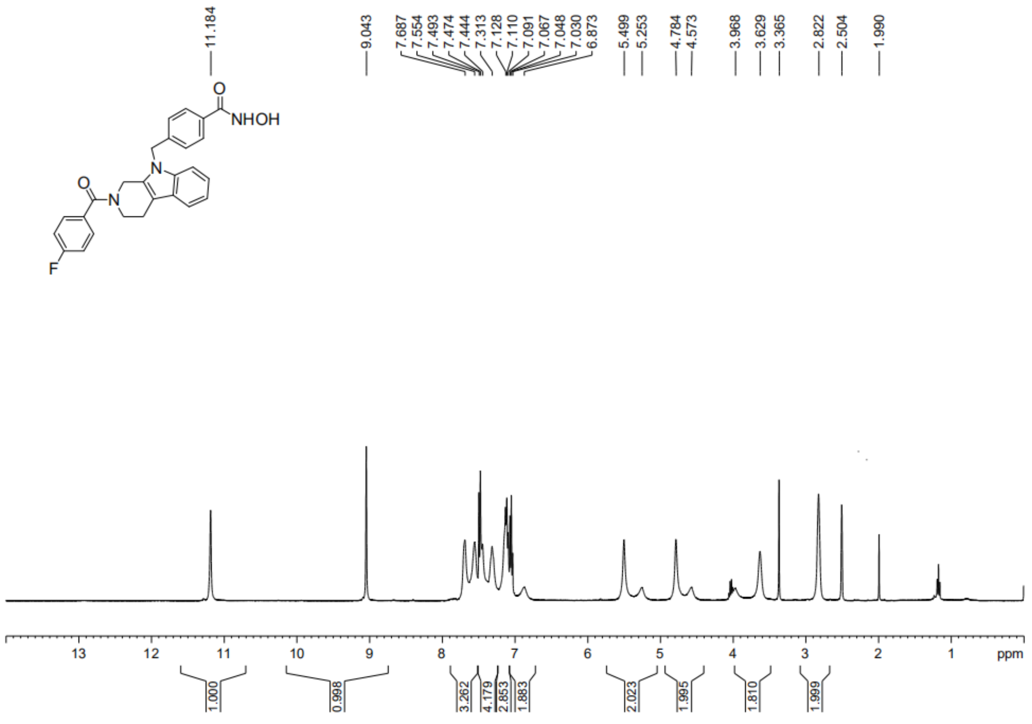

^{1}H NMR spectrum of **D11**

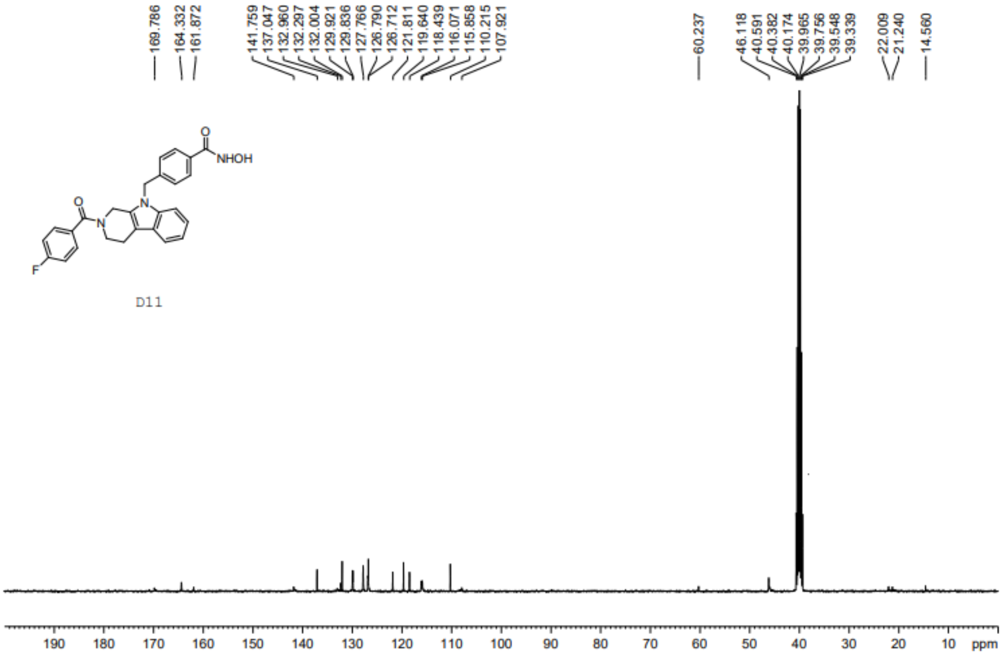

^{13}C NMR spectrum of **D11**

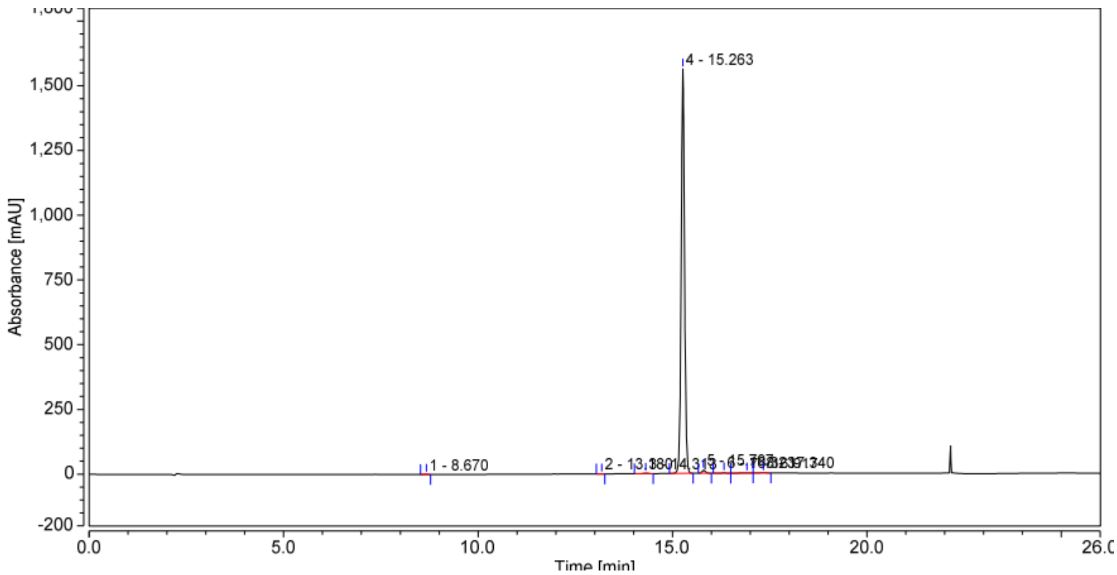

HPLC spectrum of **D11**

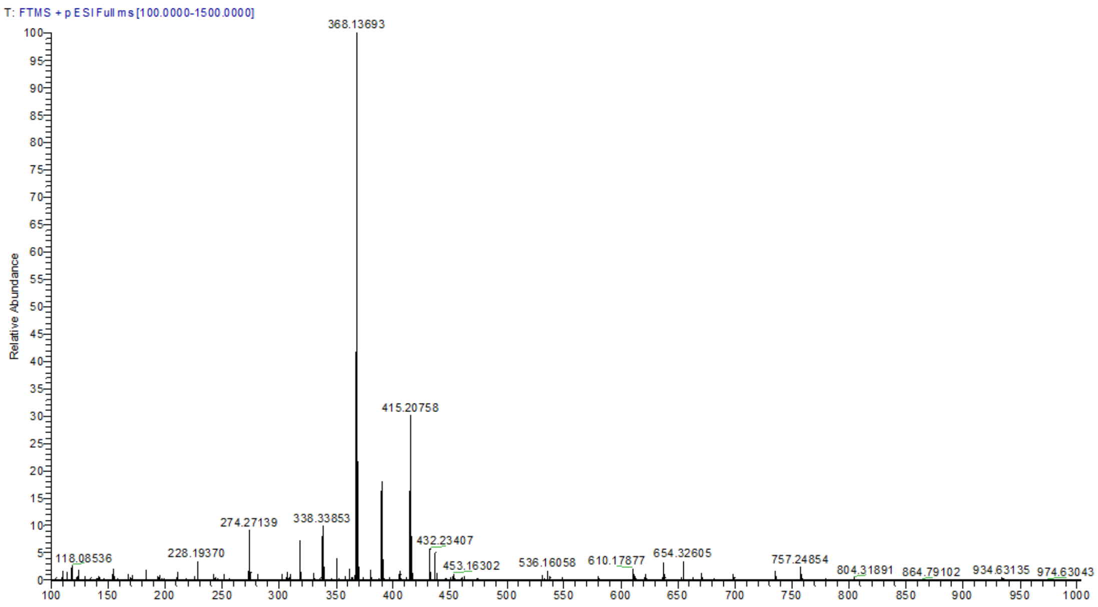

HRMS spectrum of **D12**

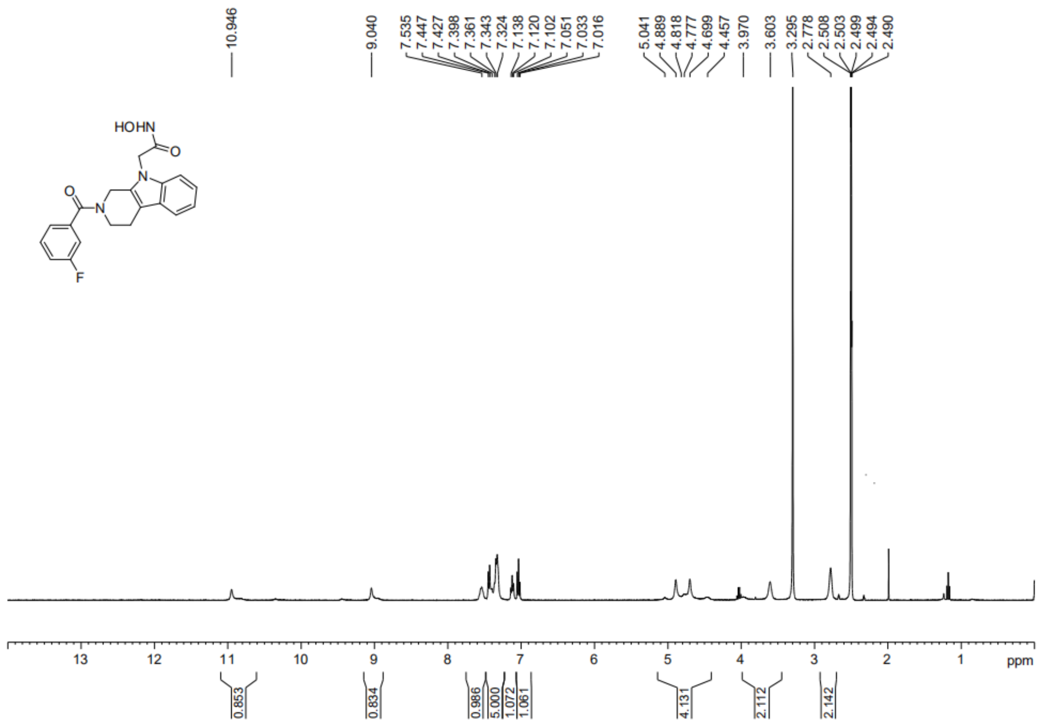

^{1}H NMR spectrum of **D12**

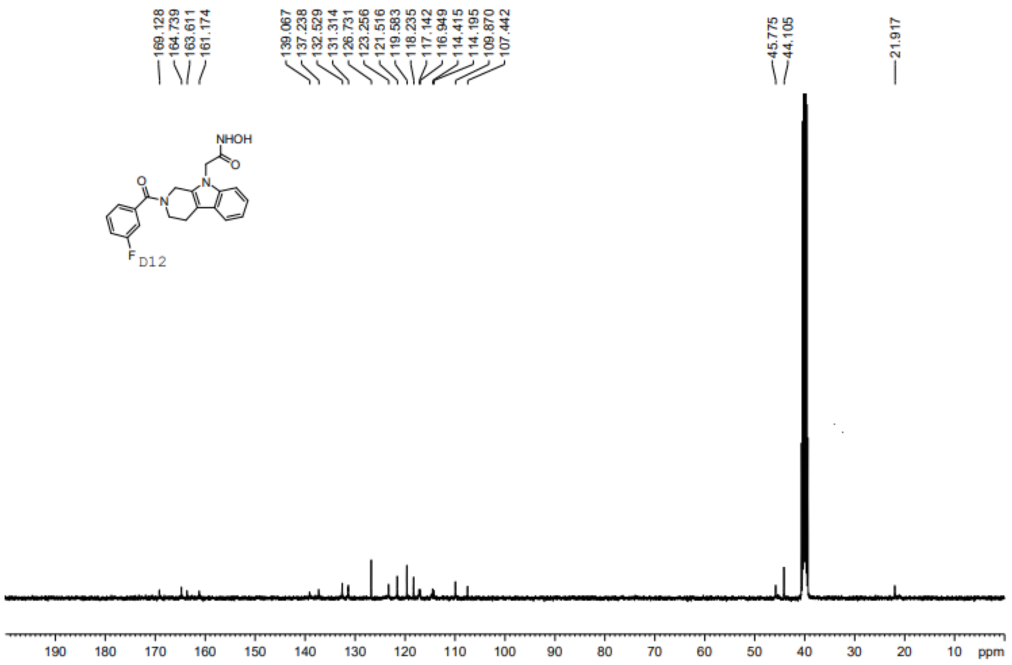

^{13}C NMR spectrum of **D12**

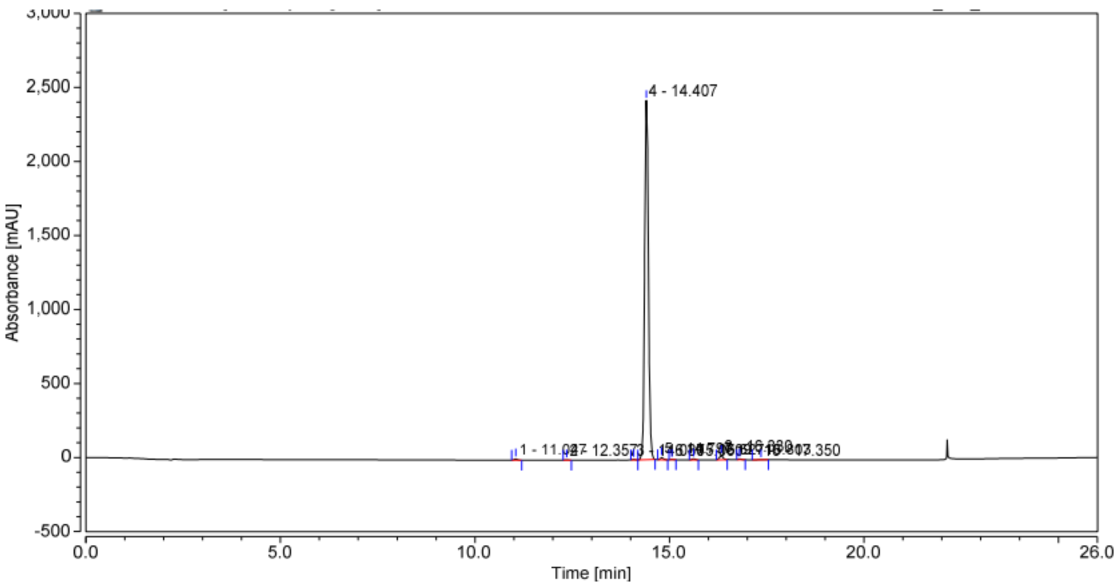

HPLC spectrum of **D12**

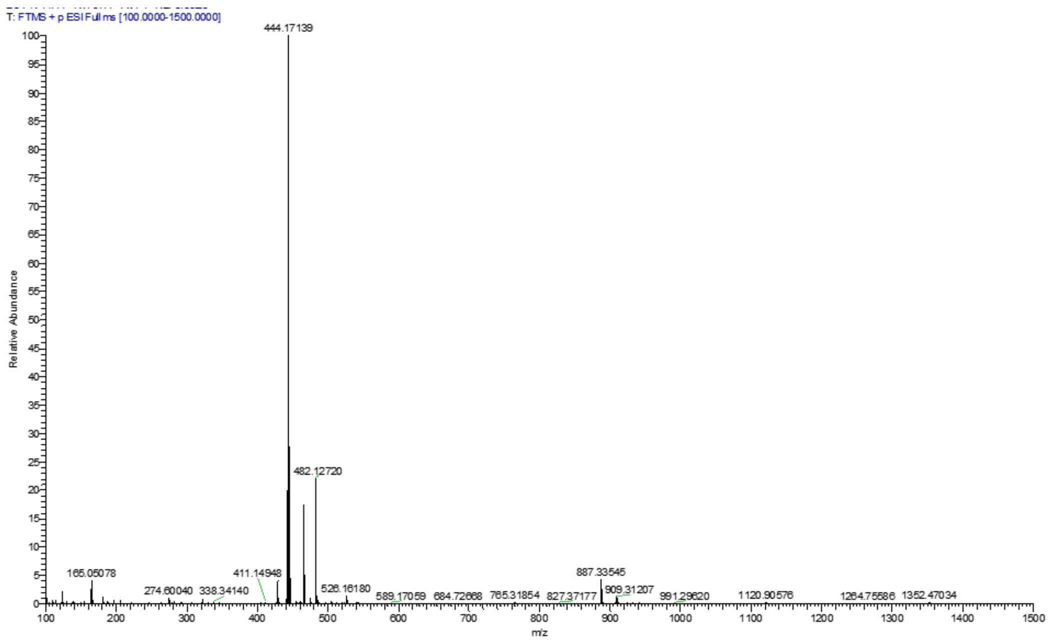

HRMS spectrum of **D13**

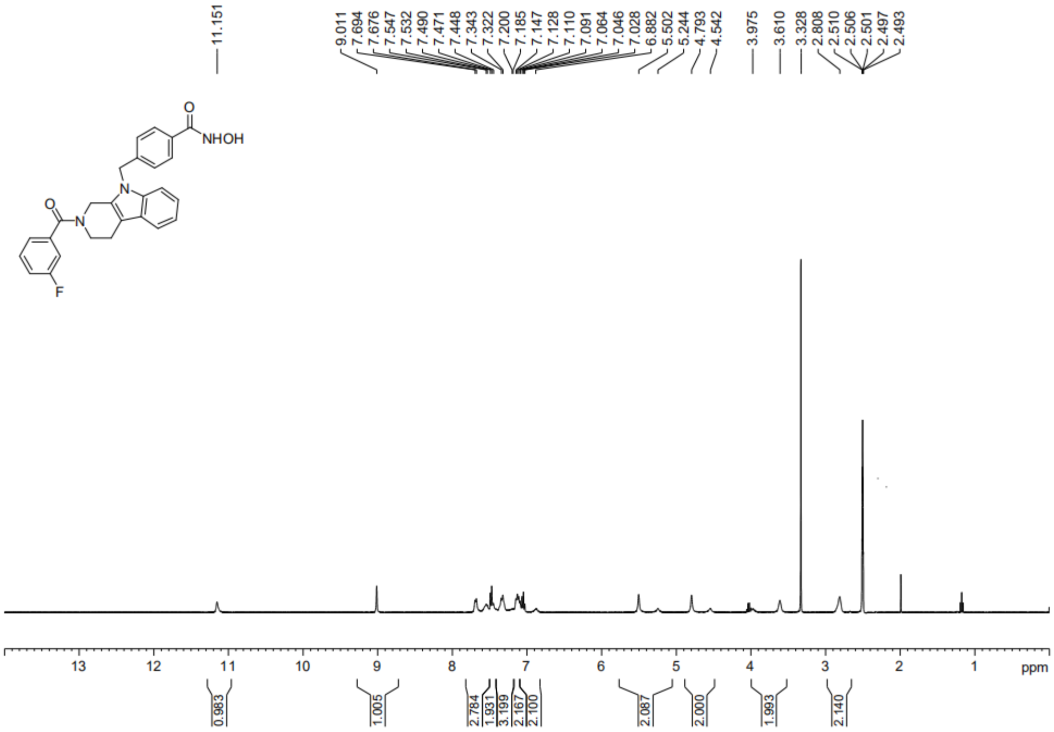

^{1}H NMR spectrum of **D13**

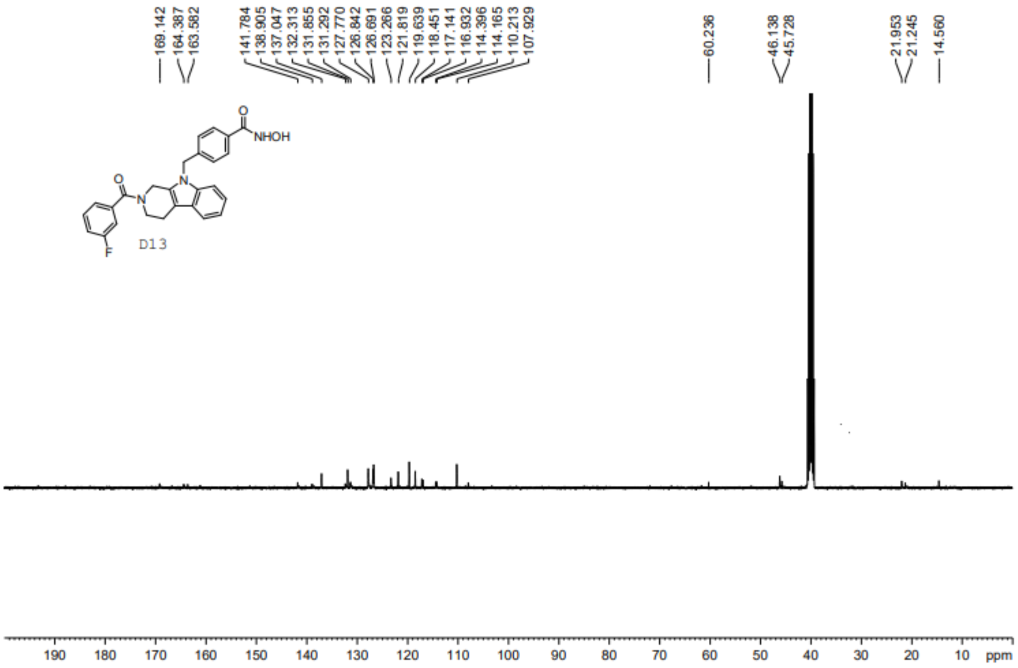

^{13}C NMR spectrum of **D13**

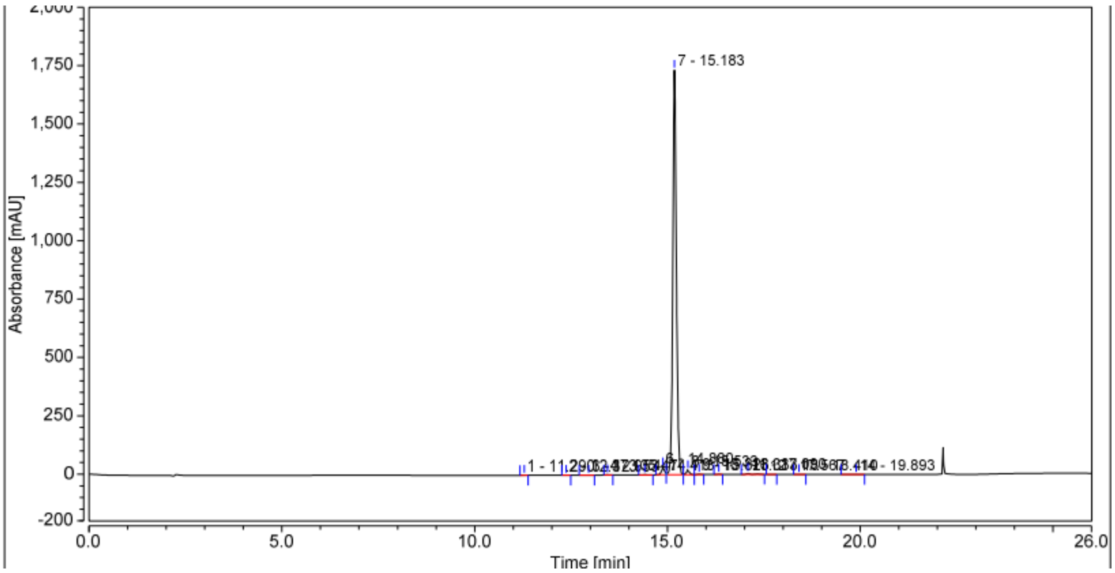

HPLC spectrum of **D13**

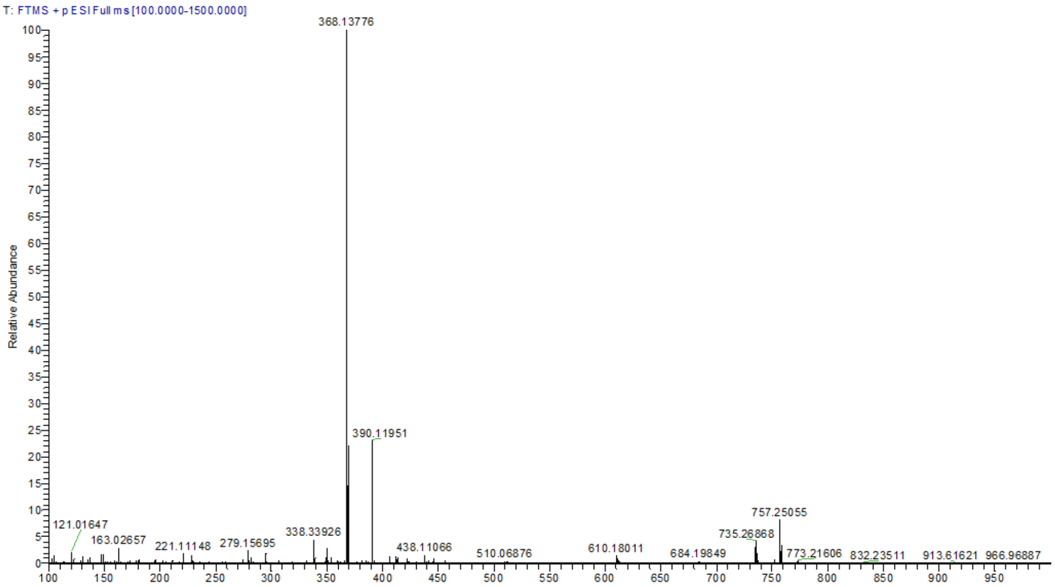

HRMS spectrum of **D14**

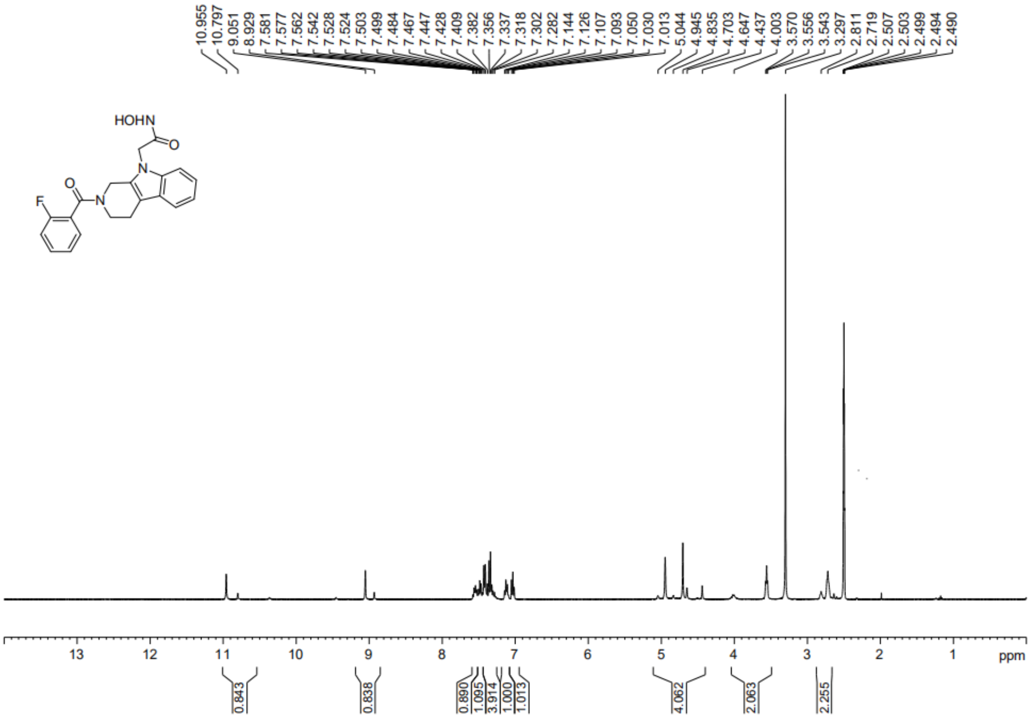

^{1}H NMR spectrum of **D14**

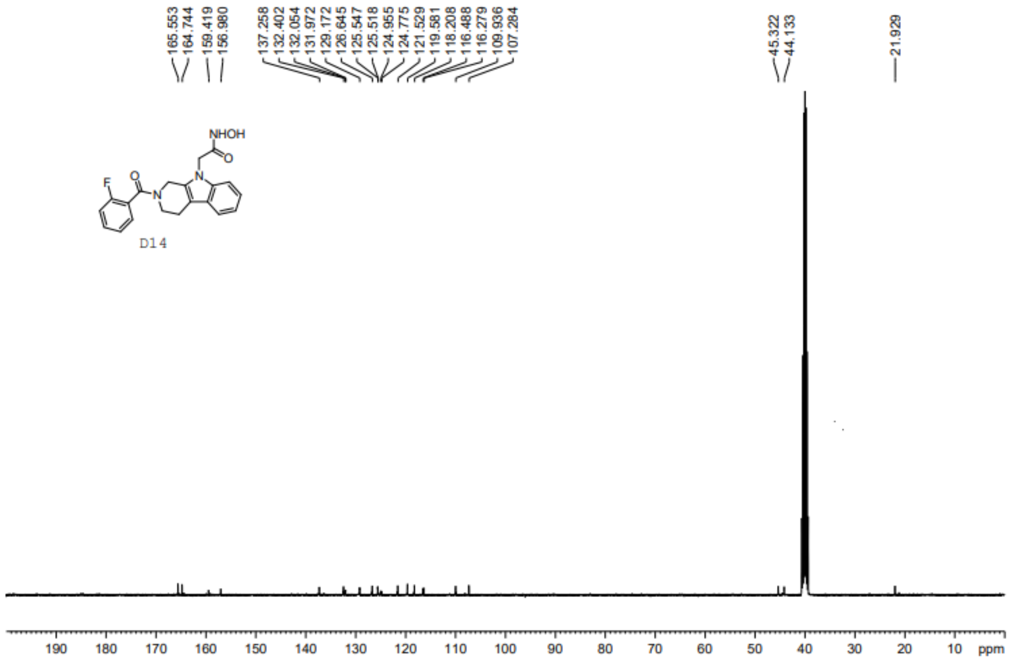

^{13}C NMR spectrum of **D14**

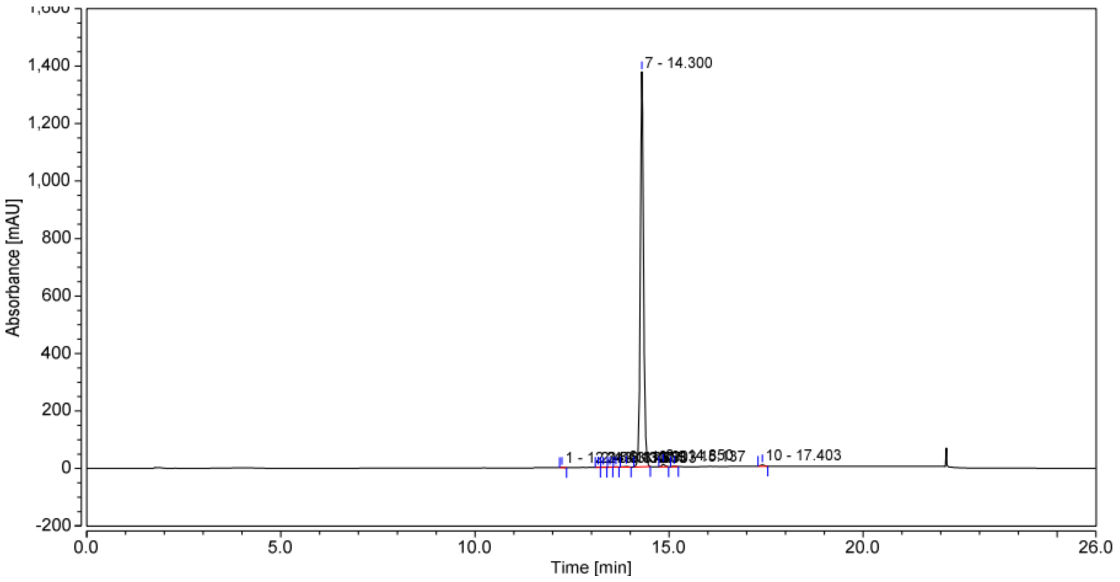

HPLC spectrum of **D14**

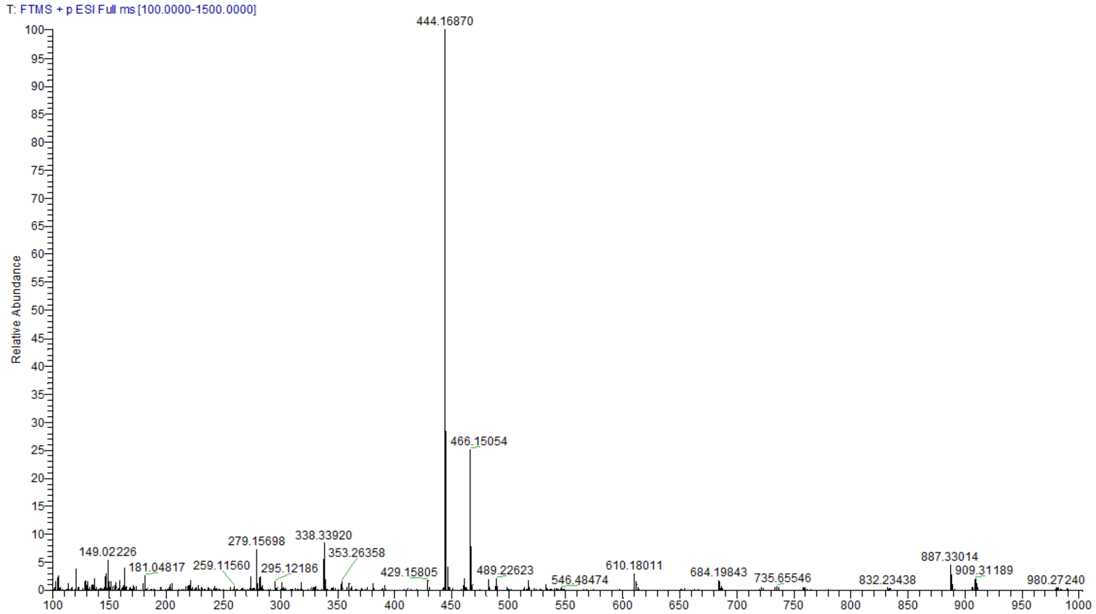

HRMS spectrum of **D15**

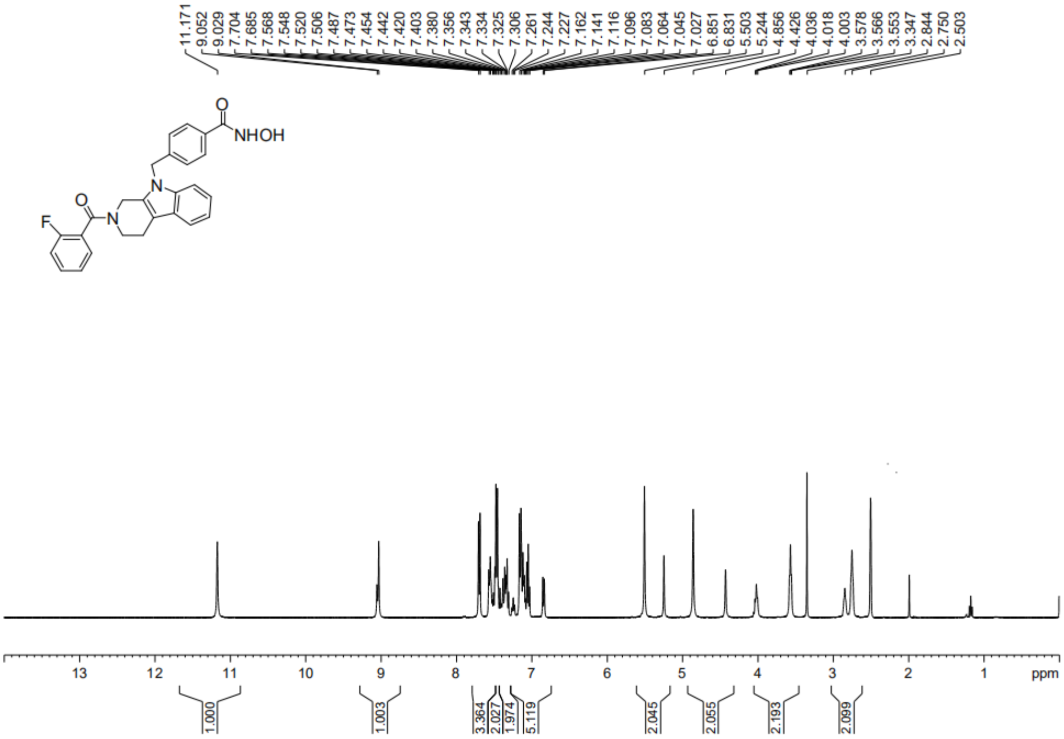

^{1}H NMR spectrum of **D15**

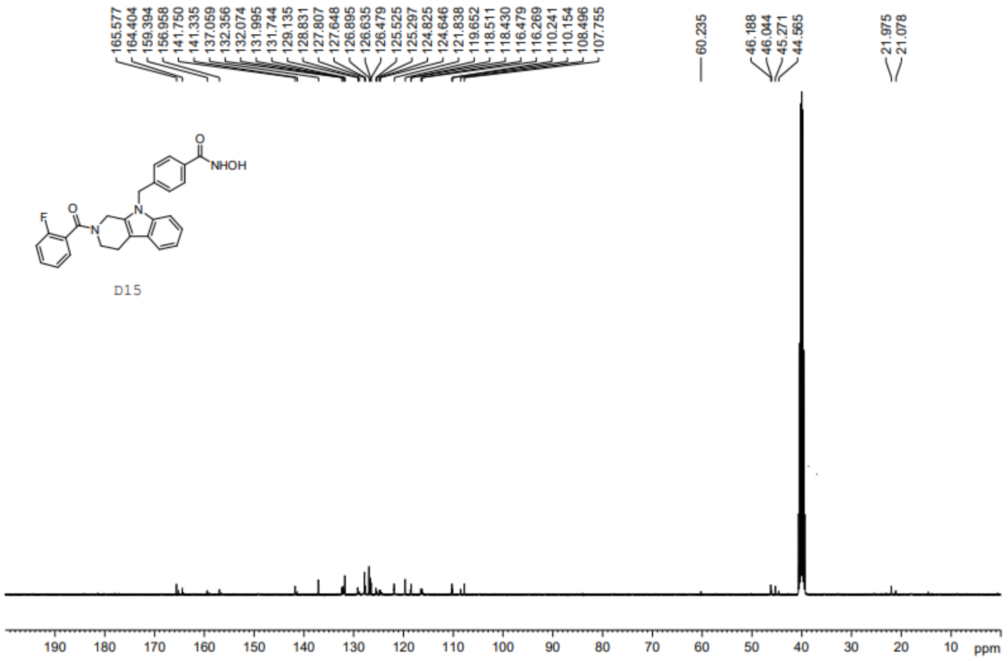

^{13}C NMR spectrum of **D15**

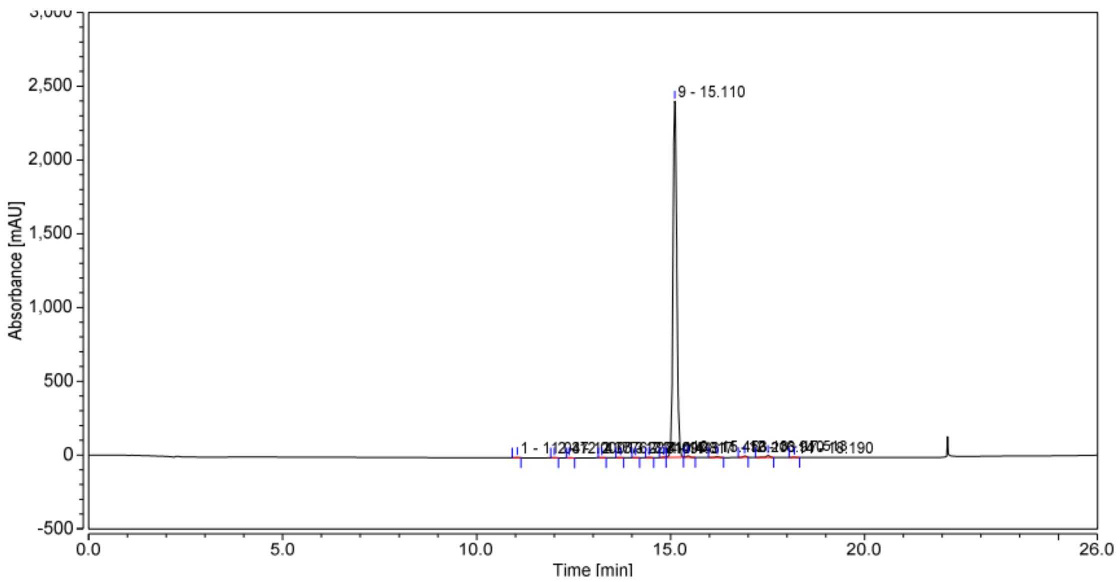

HPLC spectrum of **D15**

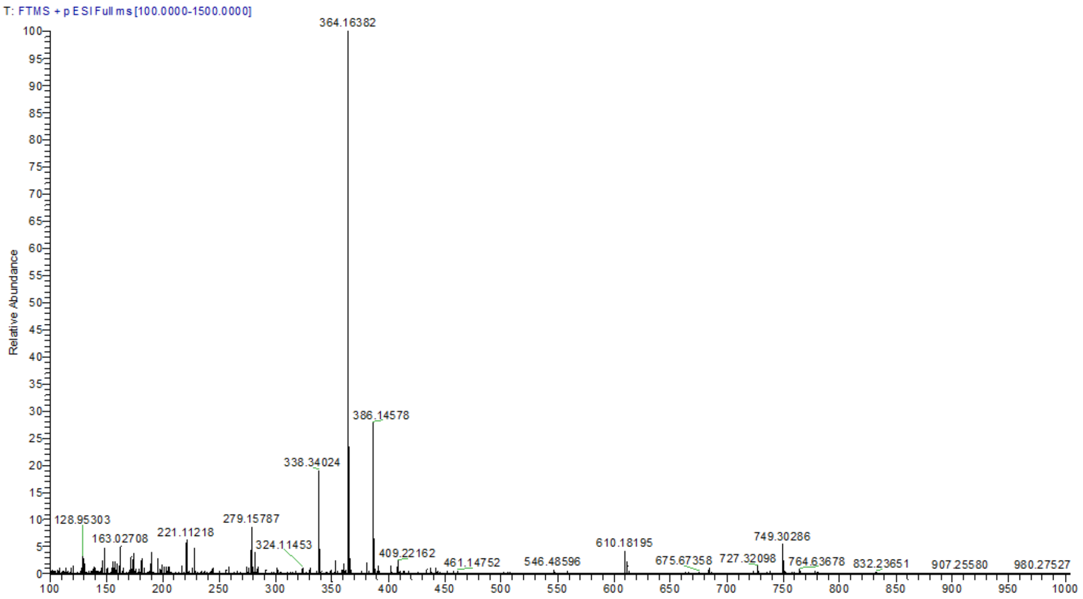

HRMS spectrum of **D16**

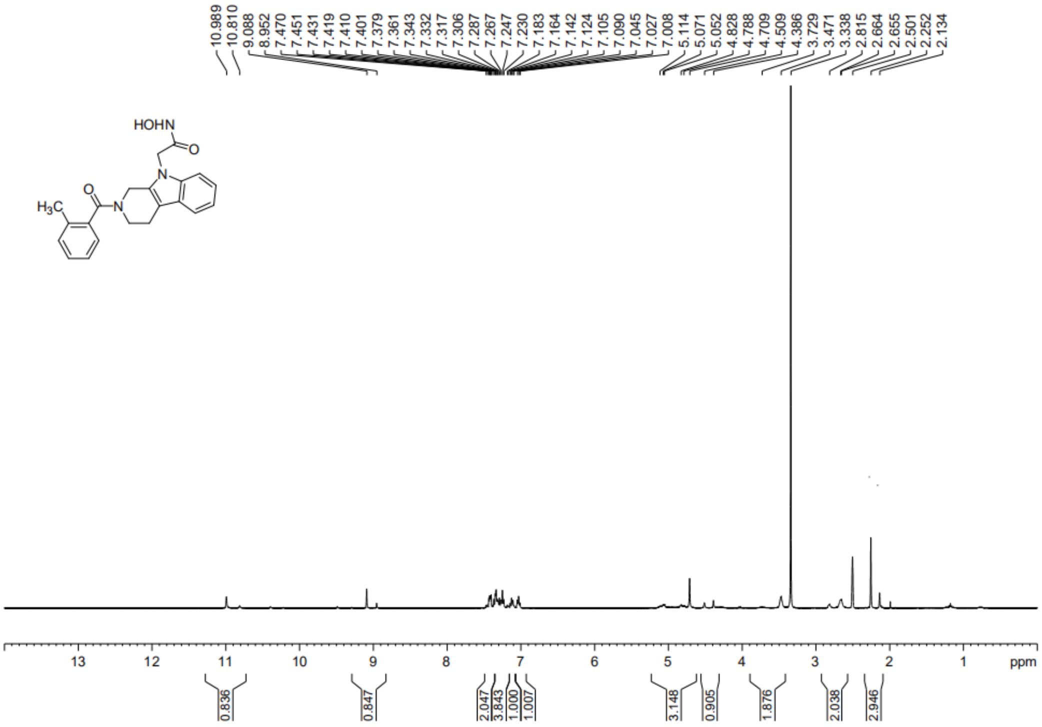

^{1}H NMR spectrum of **D16**

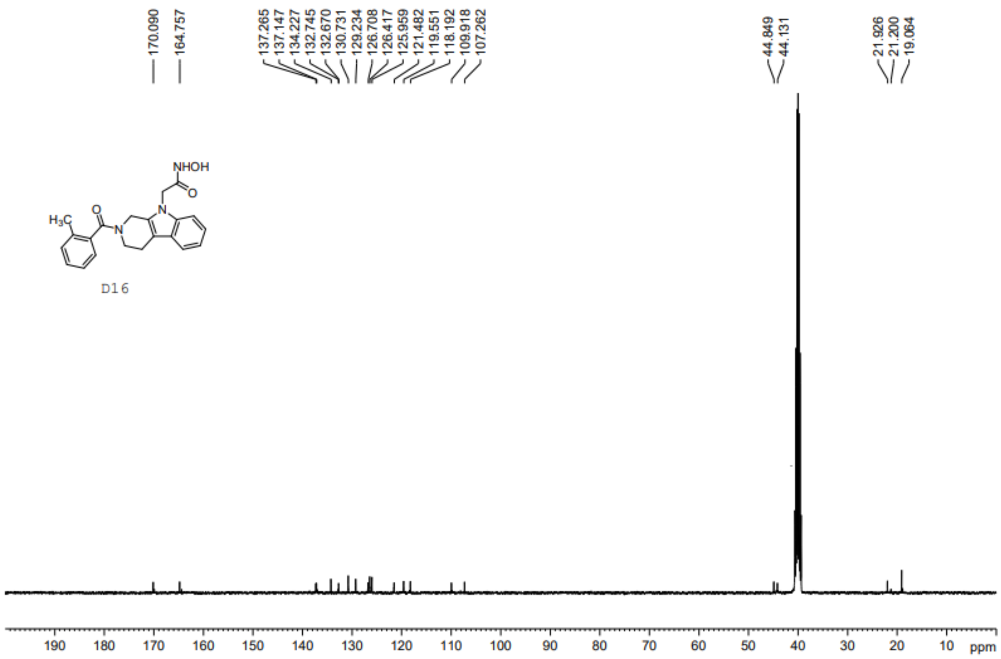

^{13}C NMR spectrum of **D16**

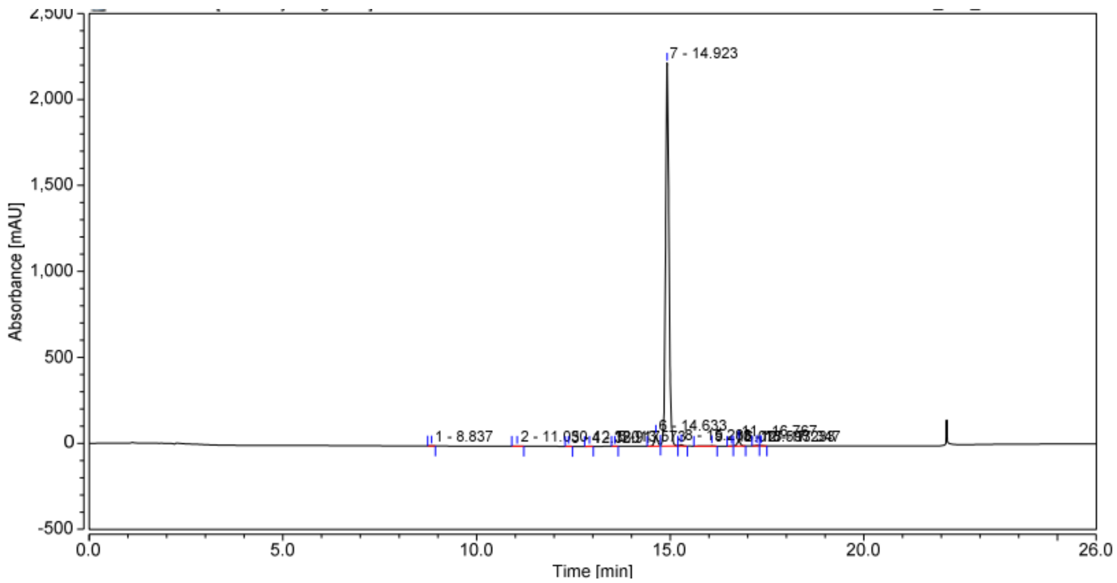

HPLC spectrum of **D16**

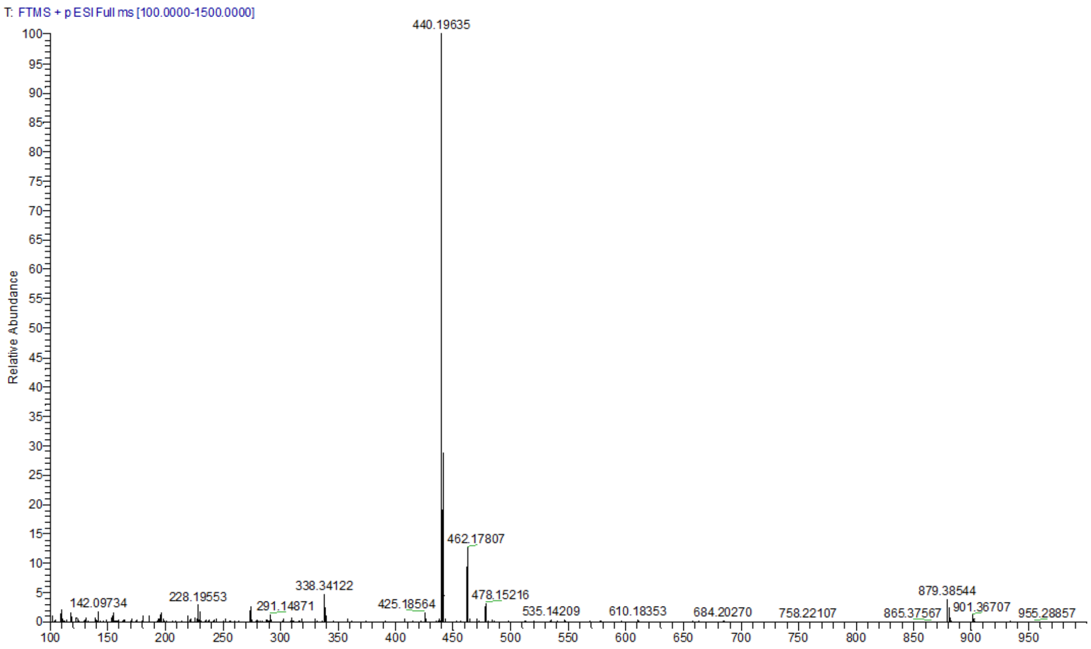

HRMS spectrum of **D17**

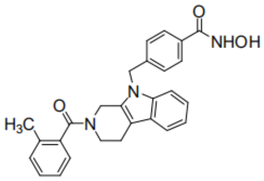

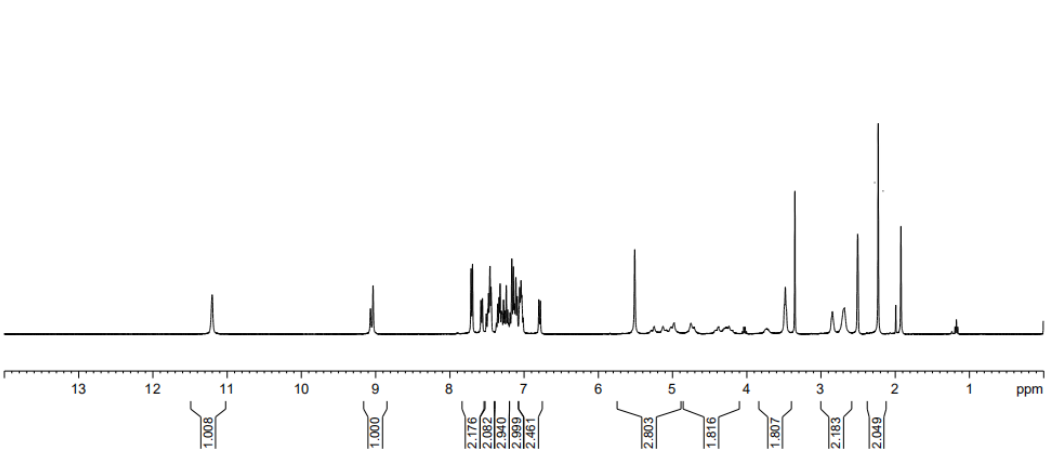

^{1}H NMR spectrum of **D17**

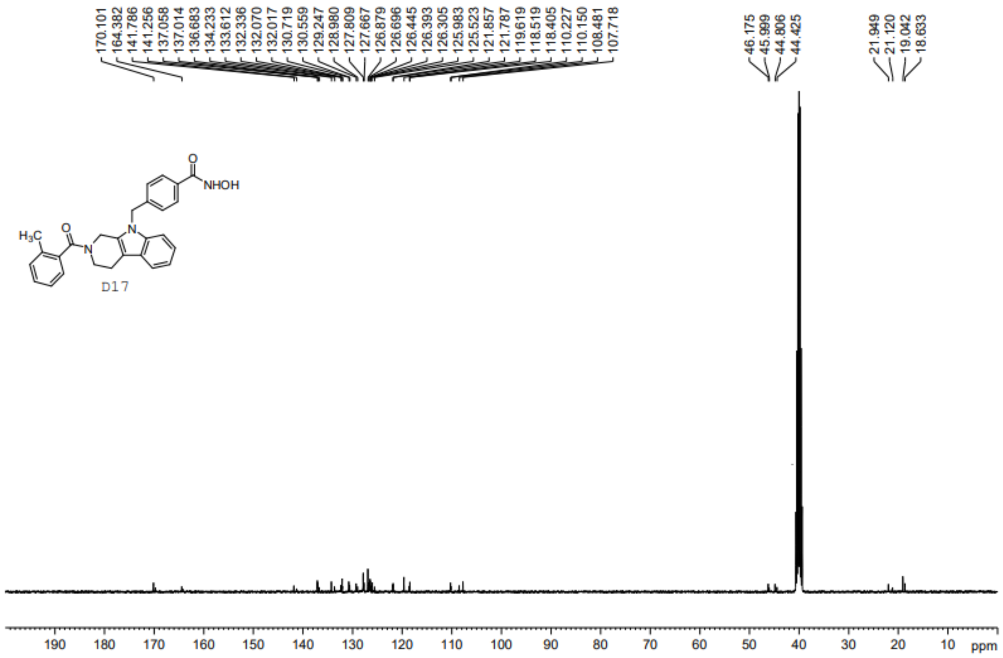

^{13}C NMR spectrum of **D17**

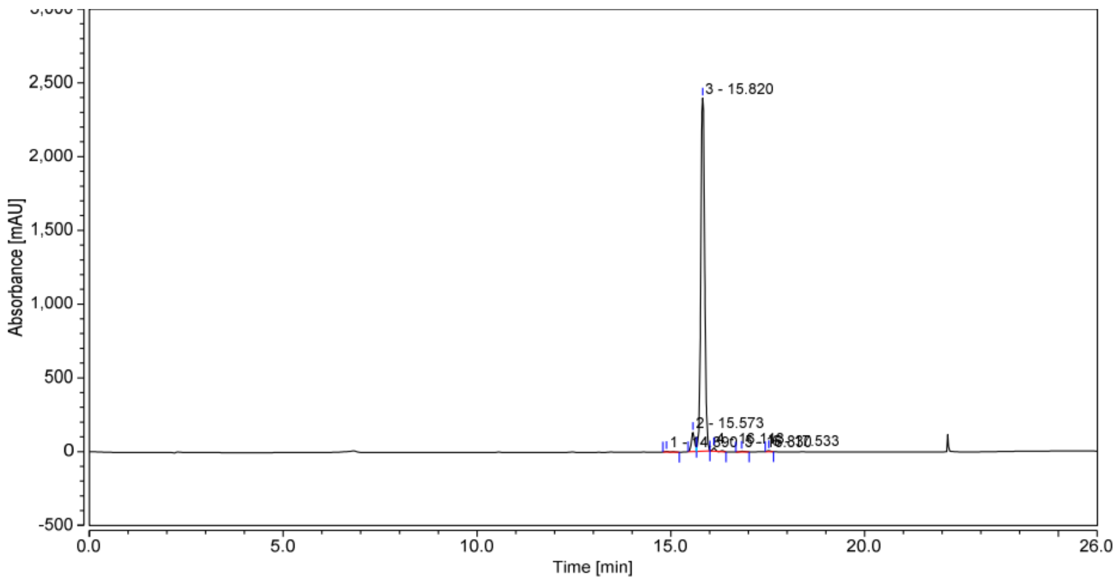

HPLC spectrum of **D17**

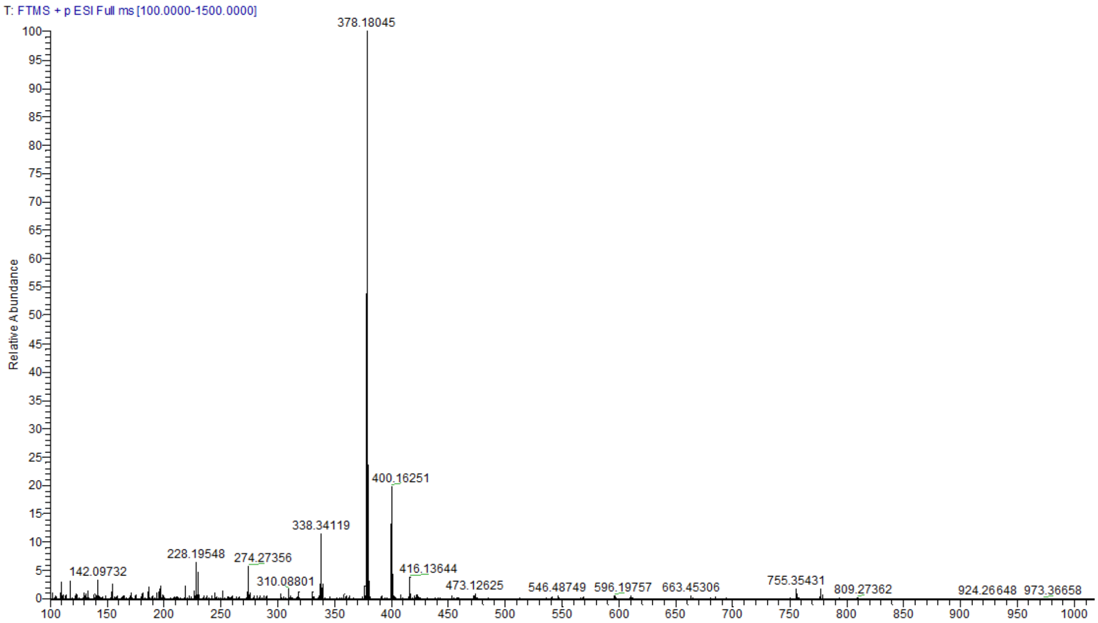

HRMS spectrum of **D18**

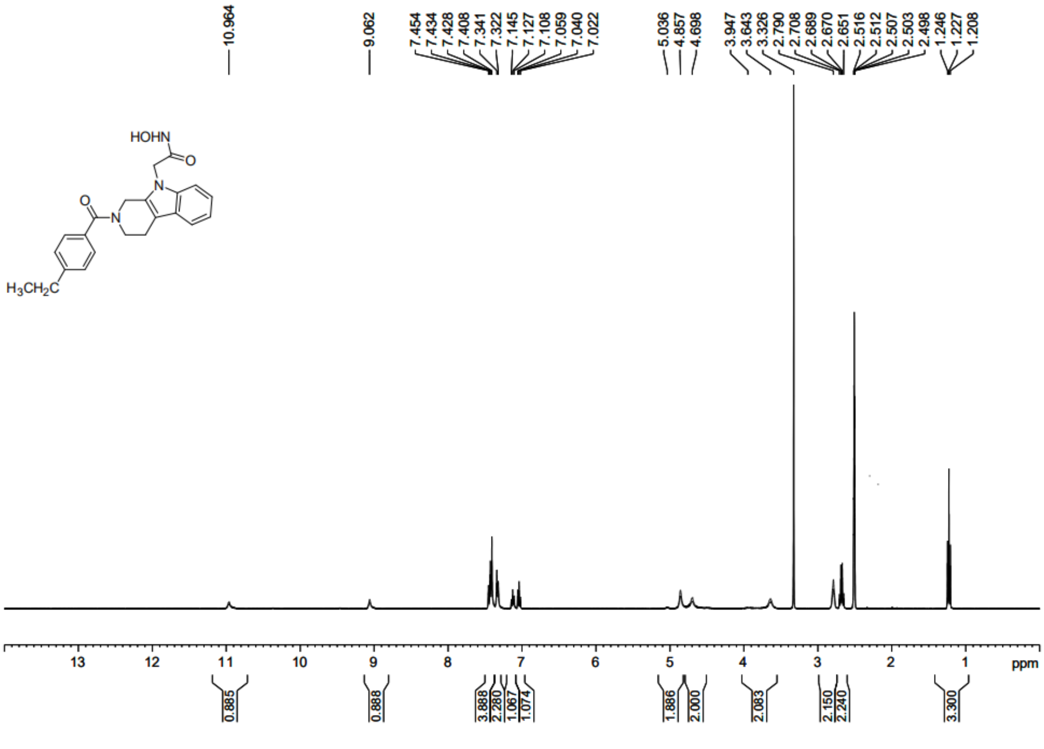

^{1}H NMR spectrum of **D18**

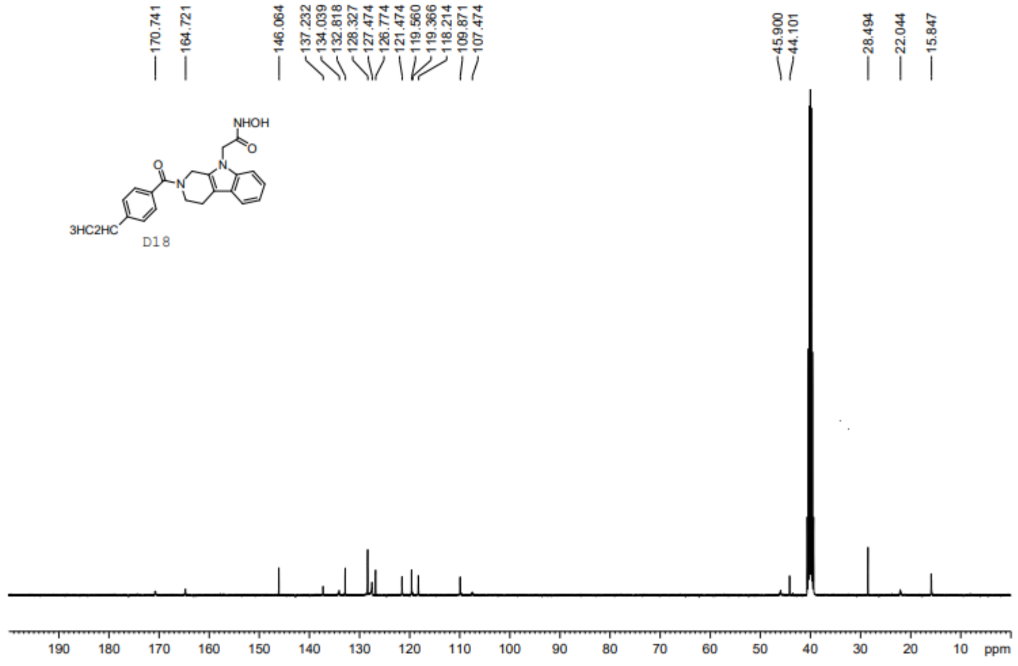

^{13}C NMR spectrum of **D18**

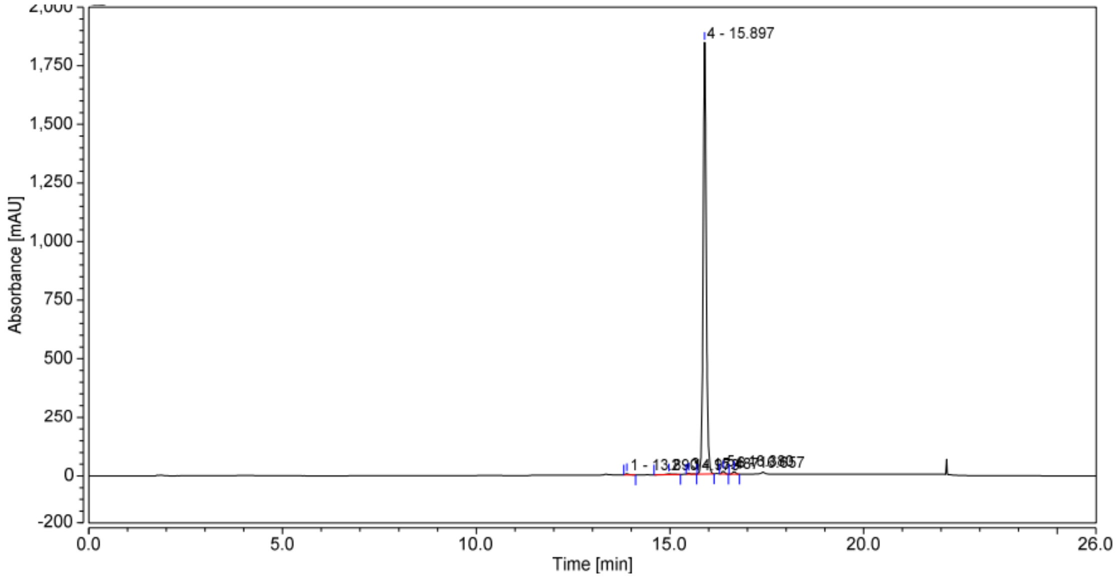

HPLC spectrum of **D18**

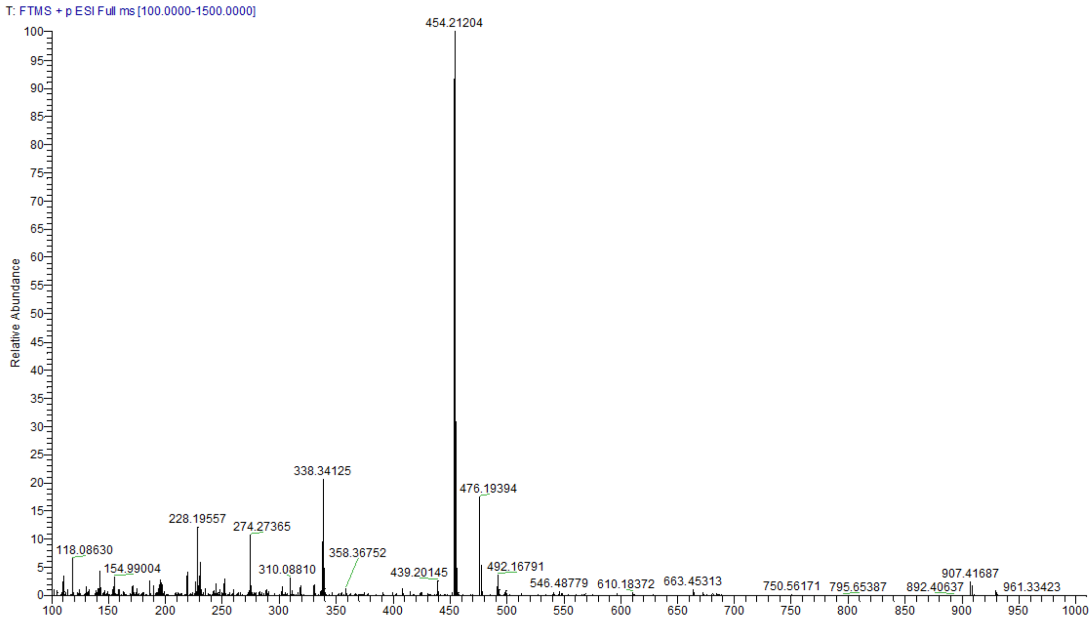

HRMS spectrum of **D19**

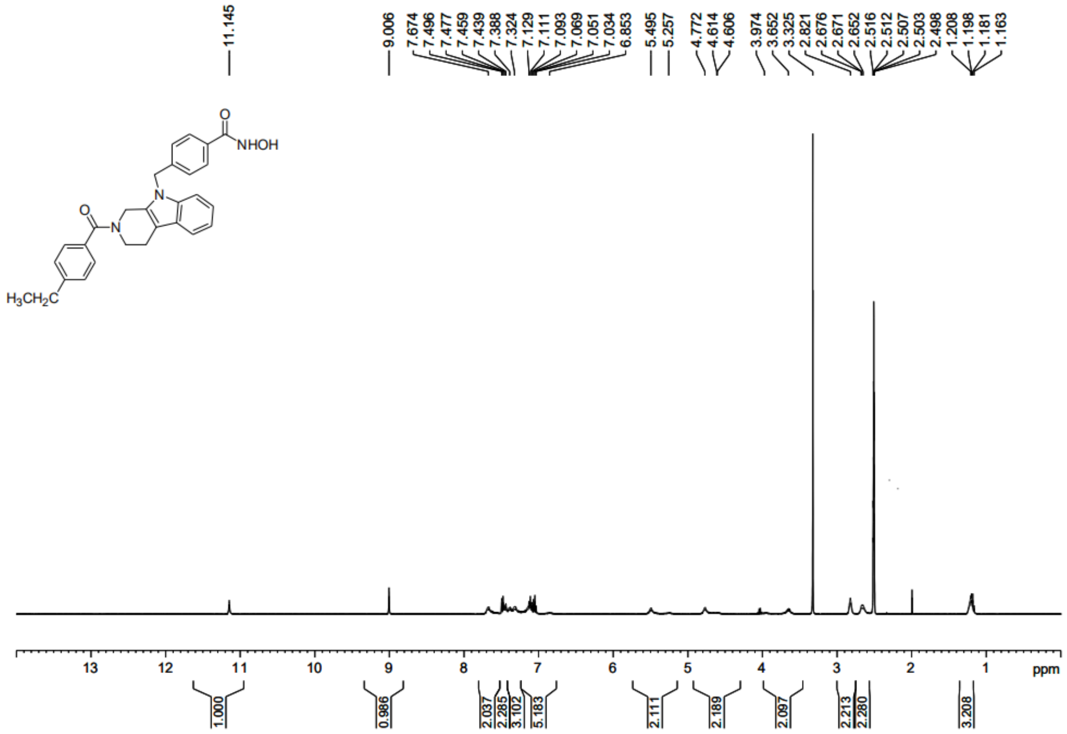

^{1}H NMR spectrum of **D19**

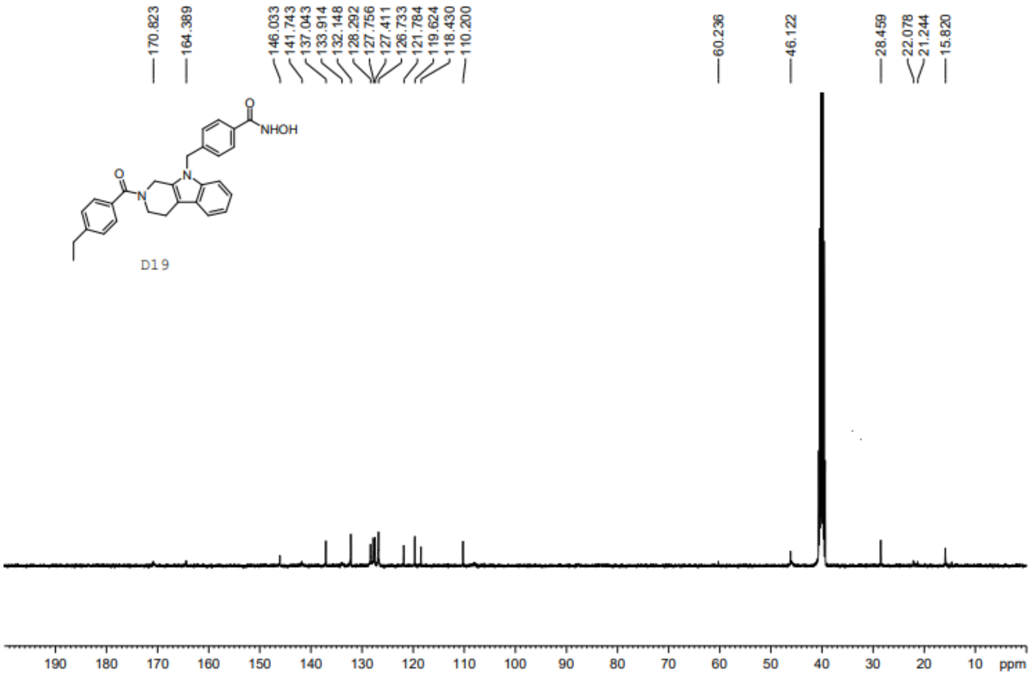

^{13}C NMR spectrum of **D19**

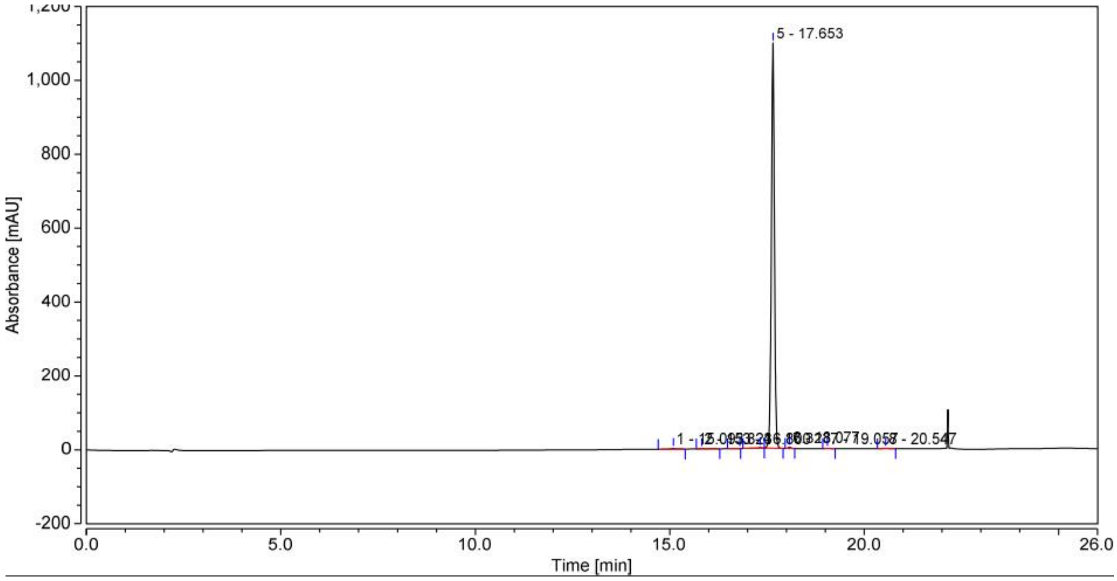

HPLC spectrum of **D19**

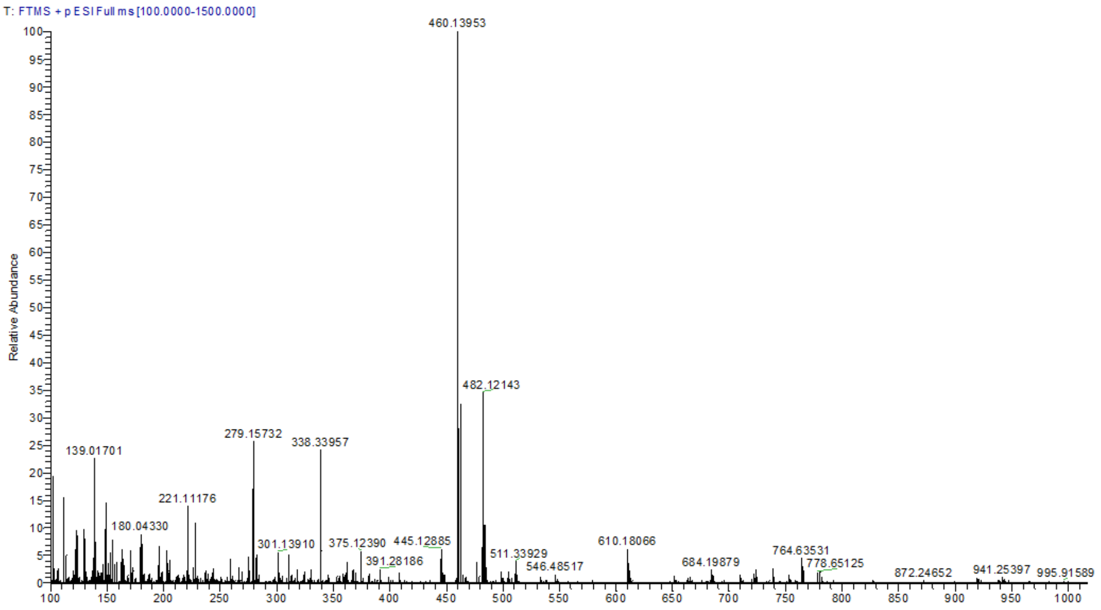

HRMS spectrum of **D20**

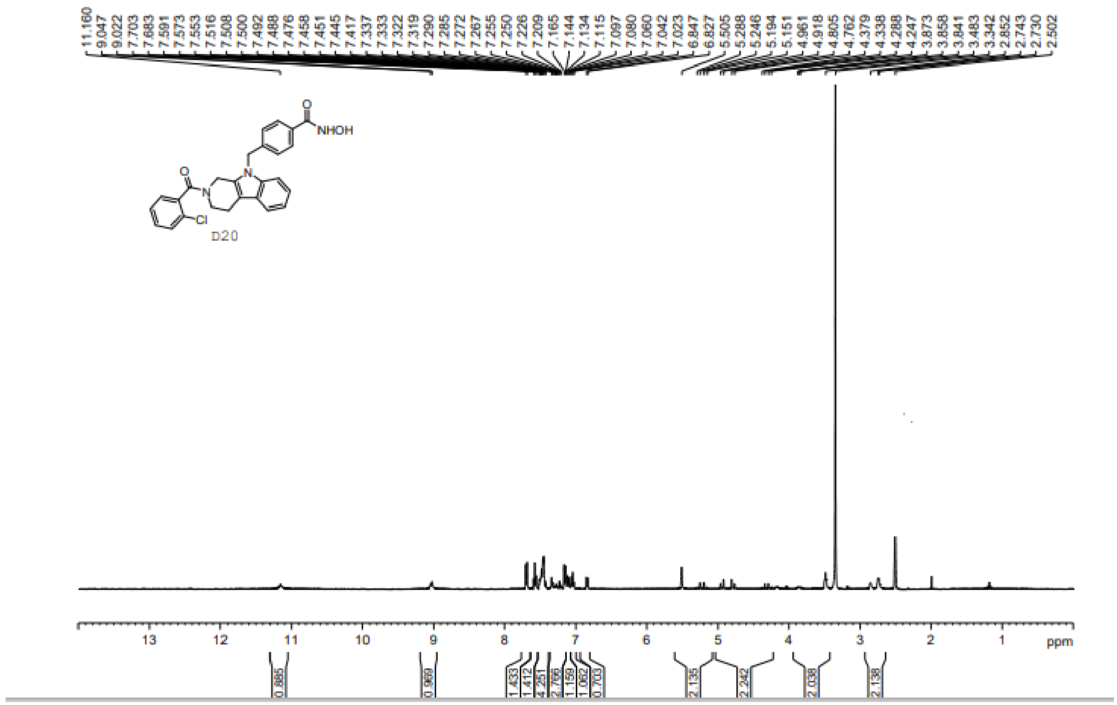

^{1}H NMR spectrum of **D20**

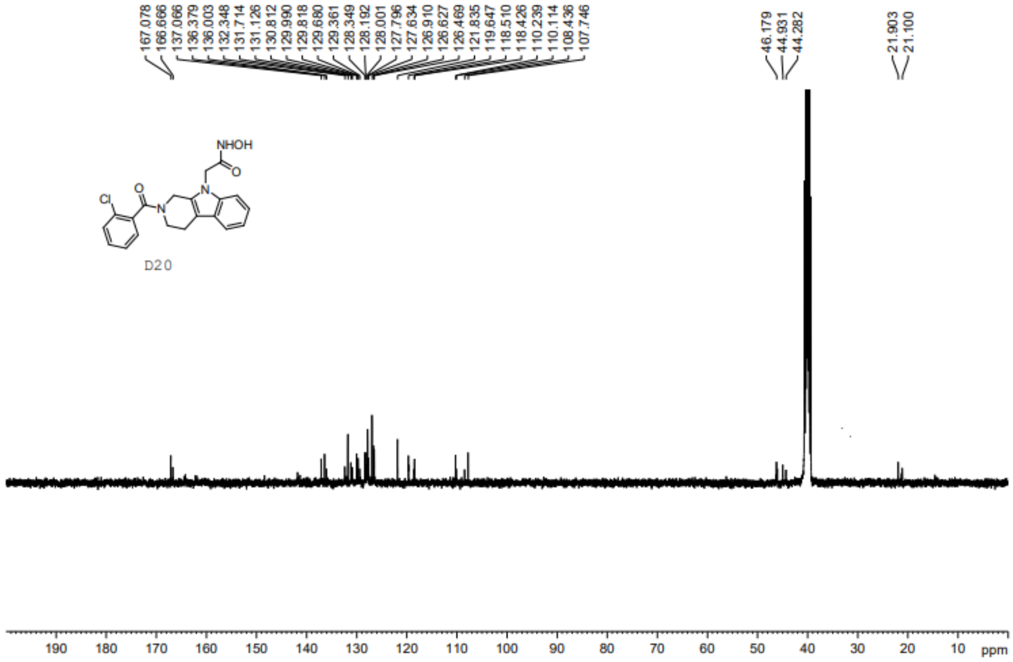

^{13}C NMR spectrum of **D20**

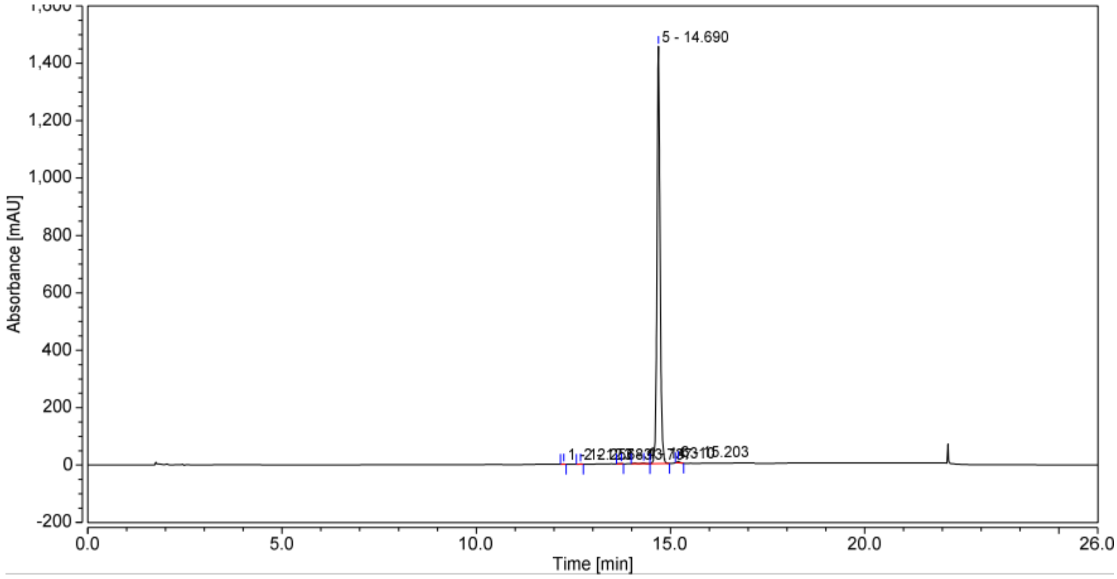

HPLC spectrum of **D20**

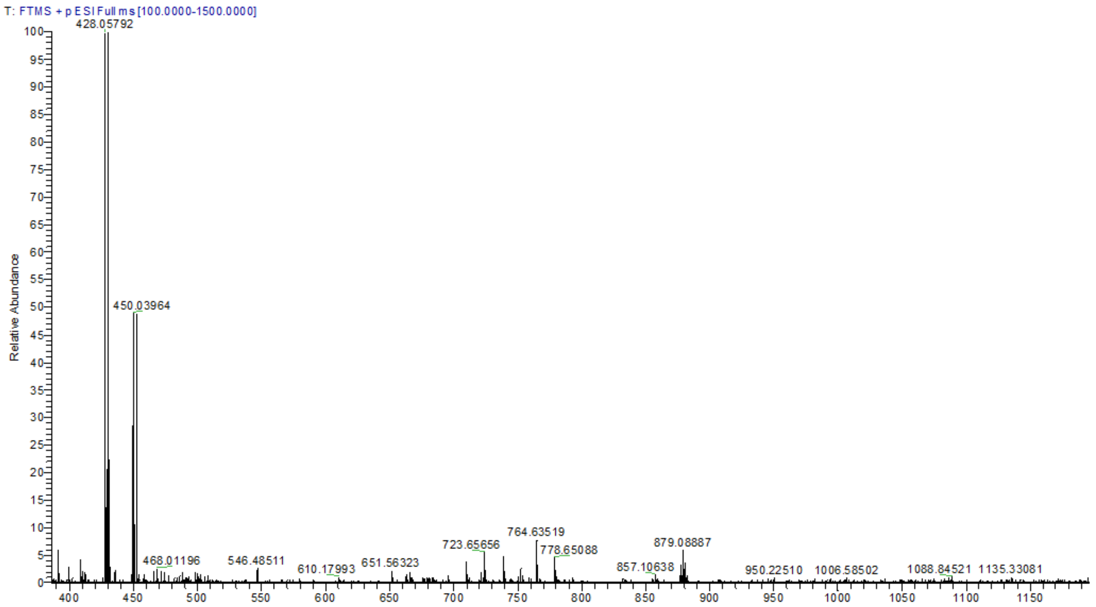

HRMS spectrum of **D21**

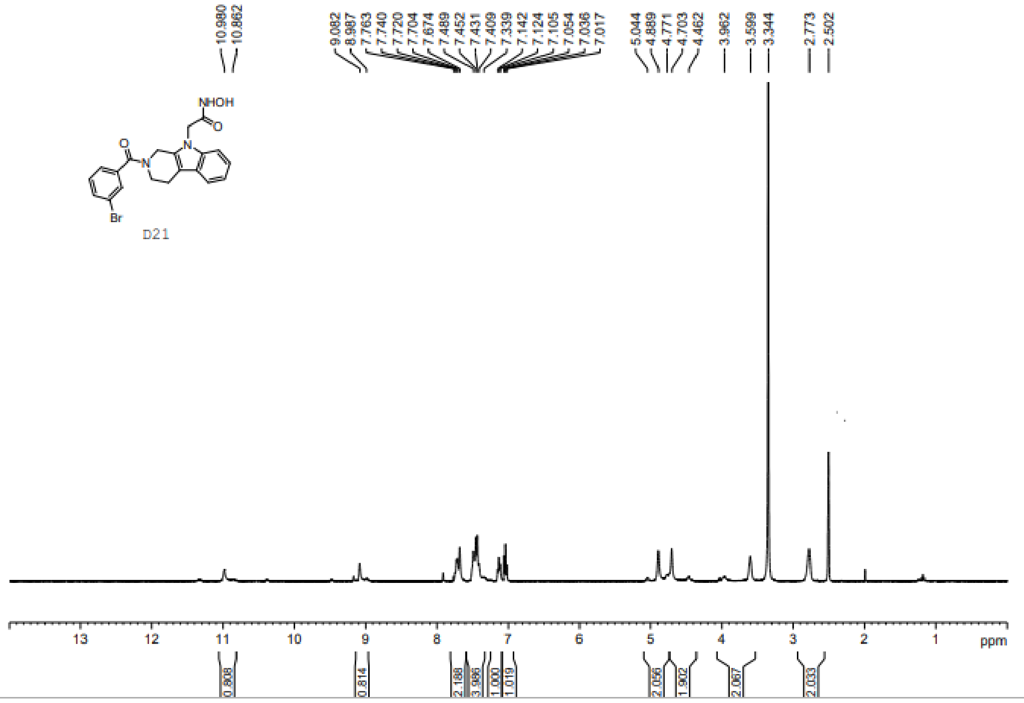

^{1}H NMR spectrum of **D21**

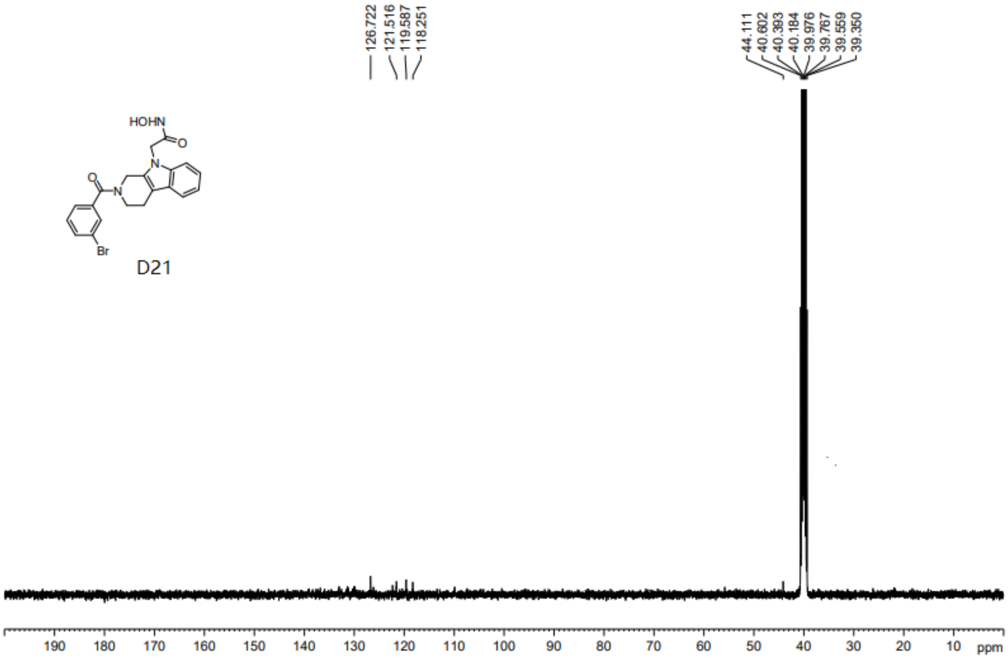

^{13}C NMR spectrum of **D21**

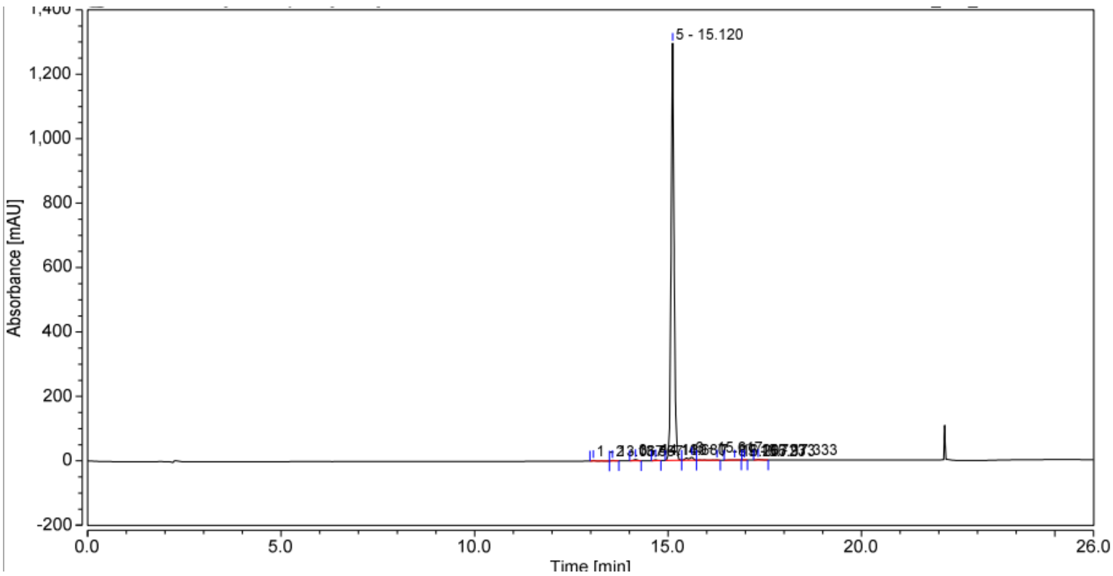

HPLC spectrum of **D21**

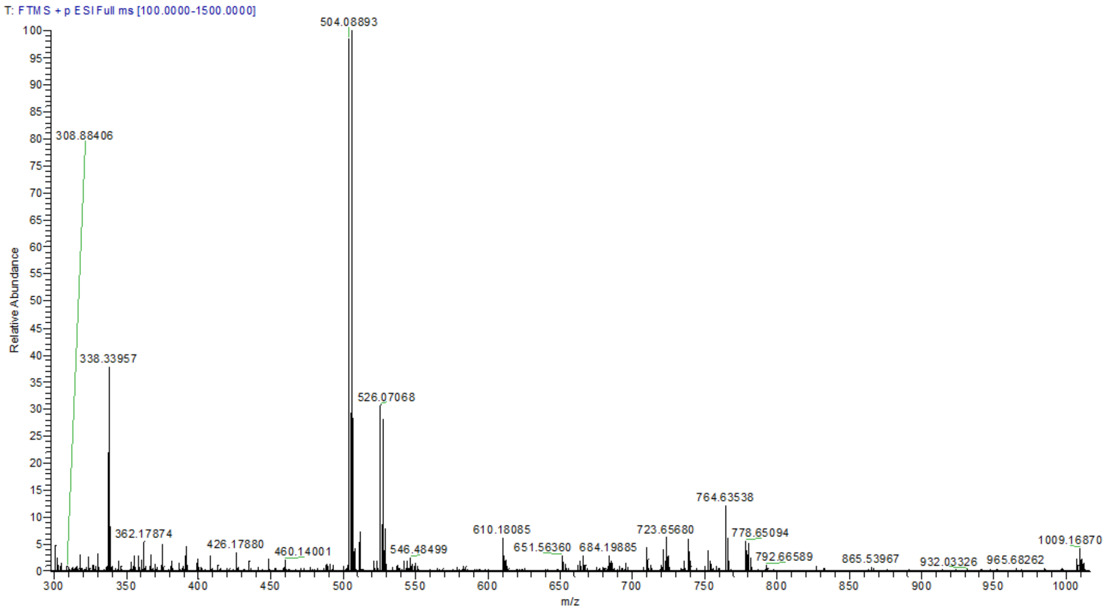

HRMS spectrum of **D22**

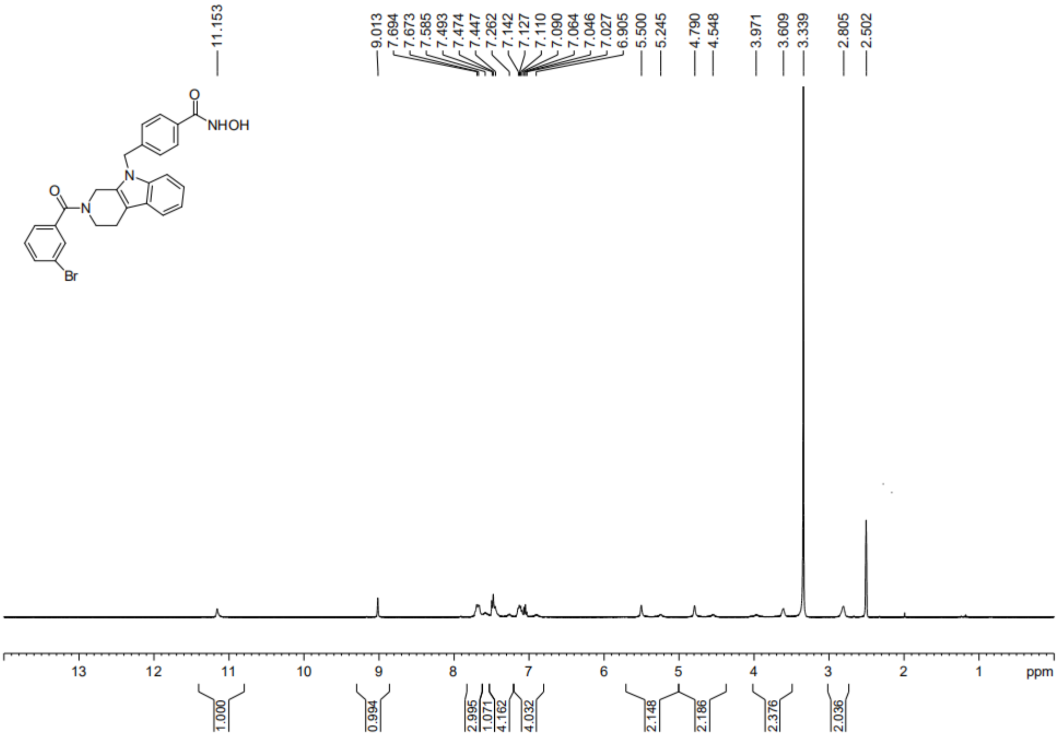

^{1}H NMR spectrum of **D22**

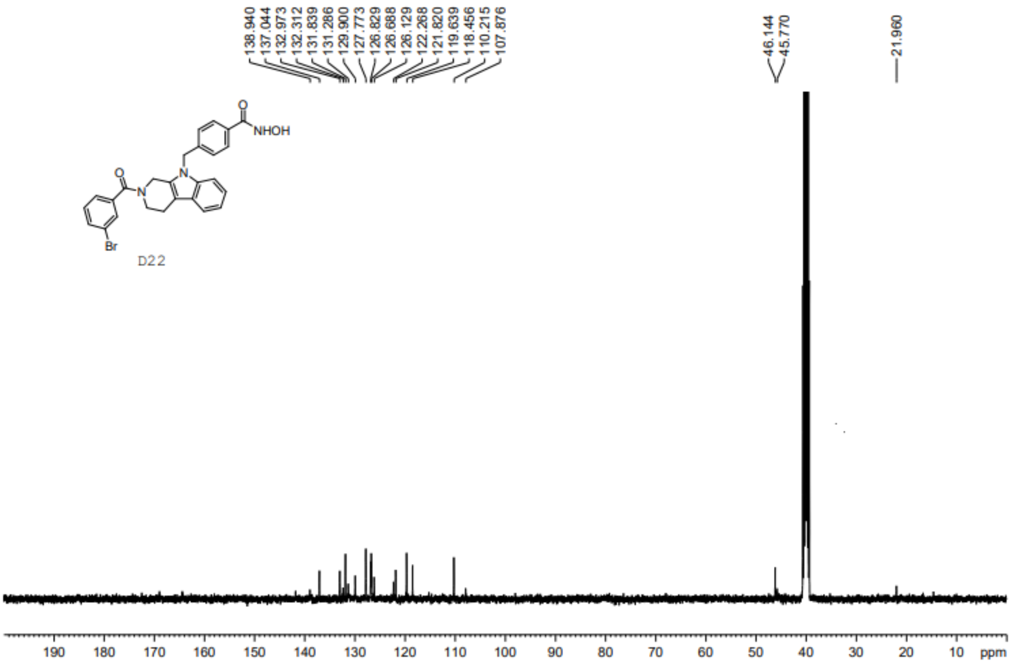

^{13}C NMR spectrum of **D22**

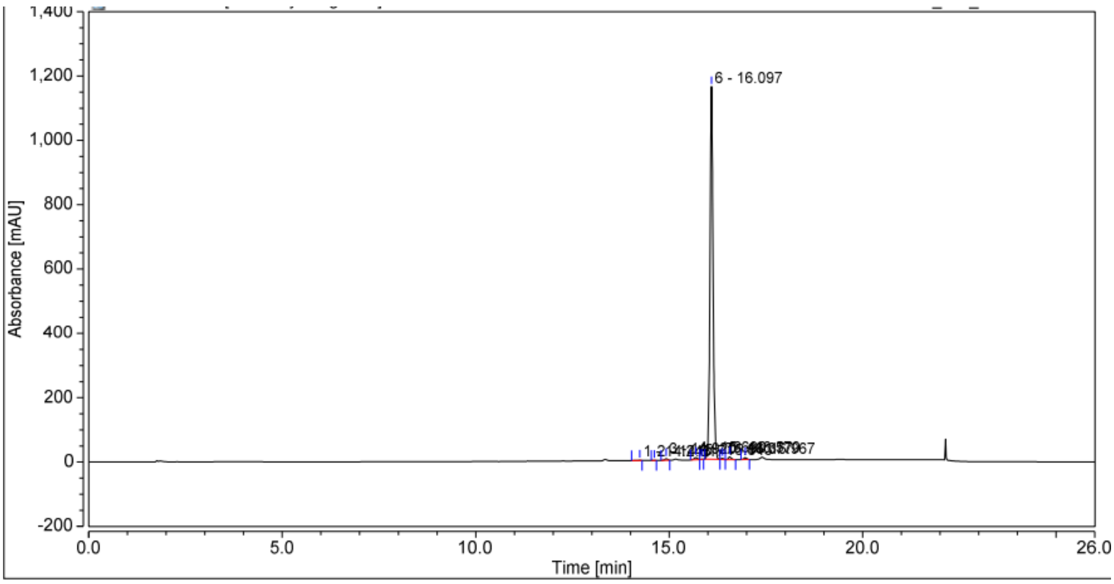

HPLC spectrum of **D22**

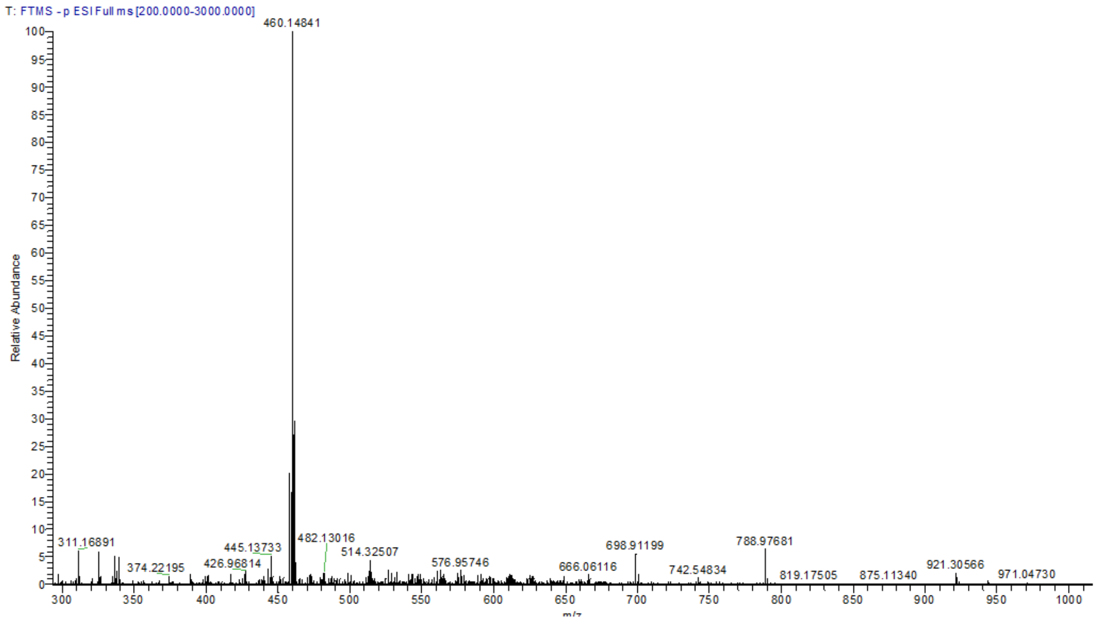

HRMS spectrum of **D23**

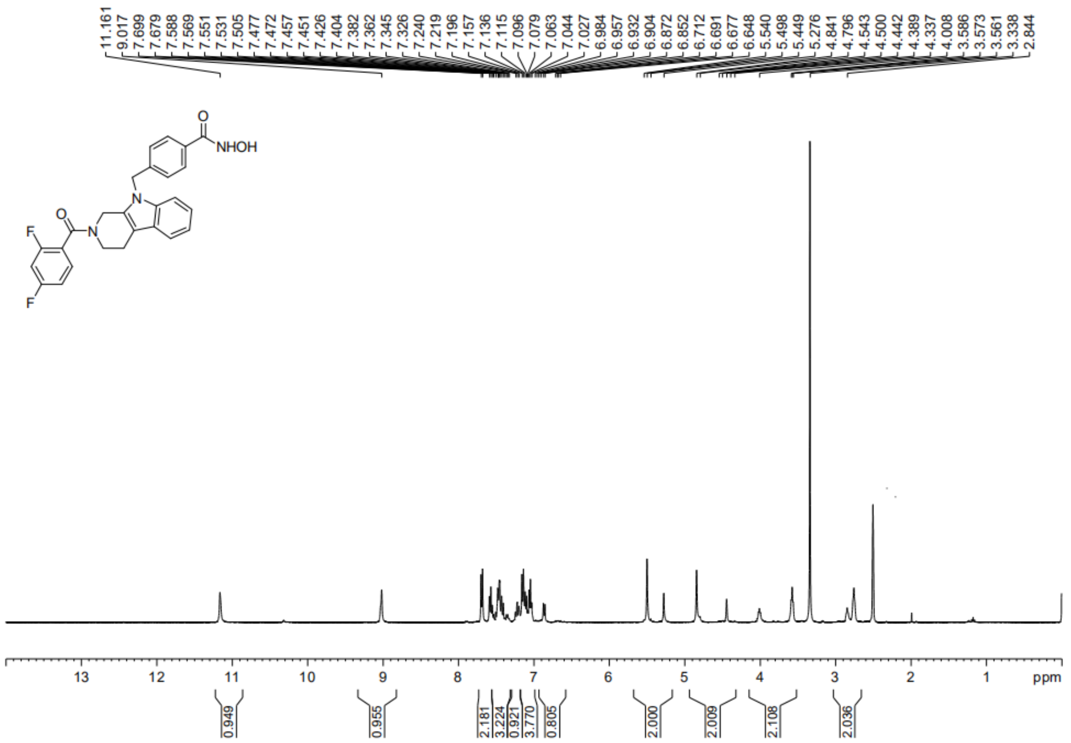

^{1}H NMR spectrum of **D23**

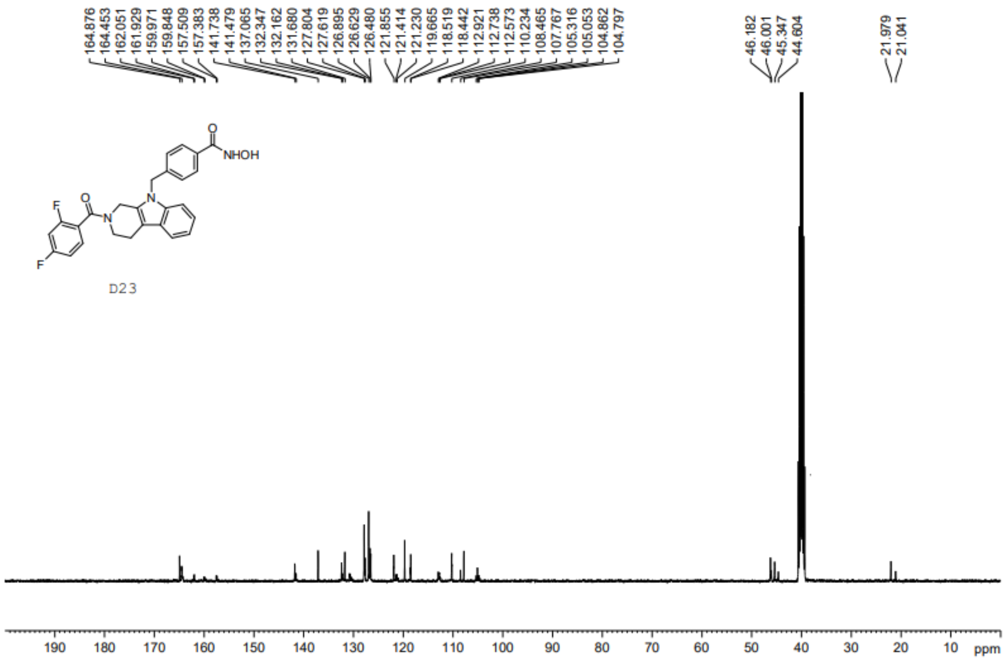

^{13}C NMR spectrum of **D23**

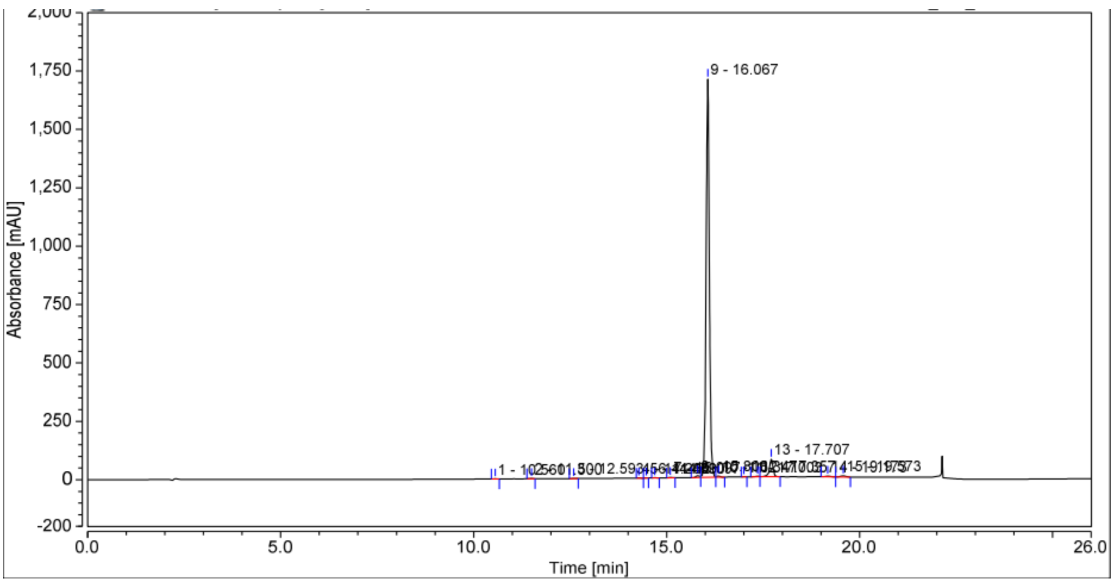

HPLC spectrum of **D23**

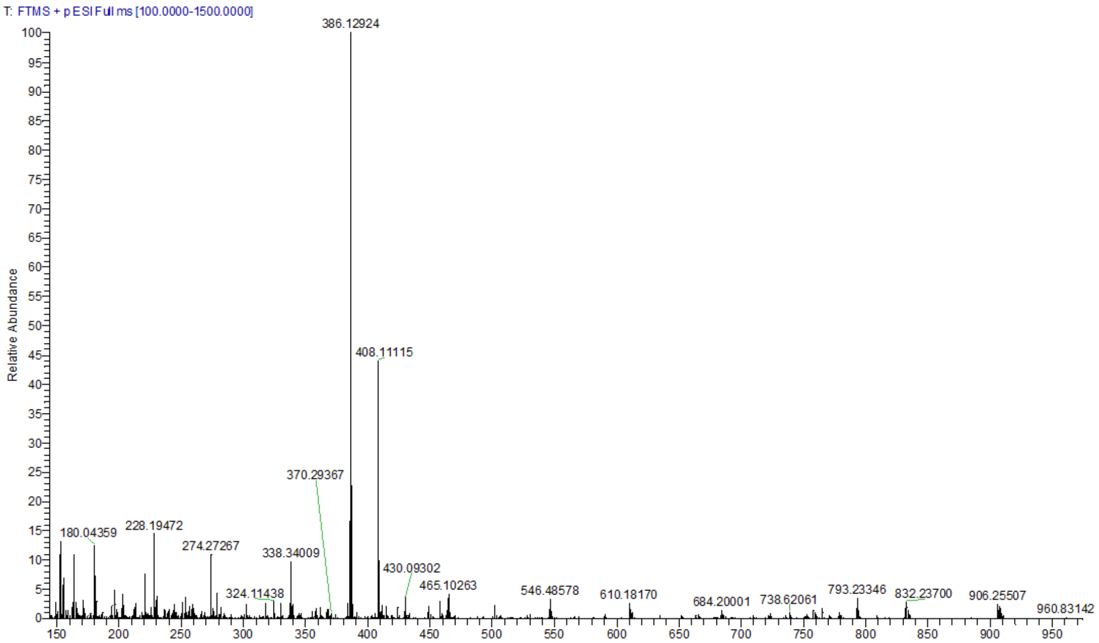

HRMS spectrum of **D24**

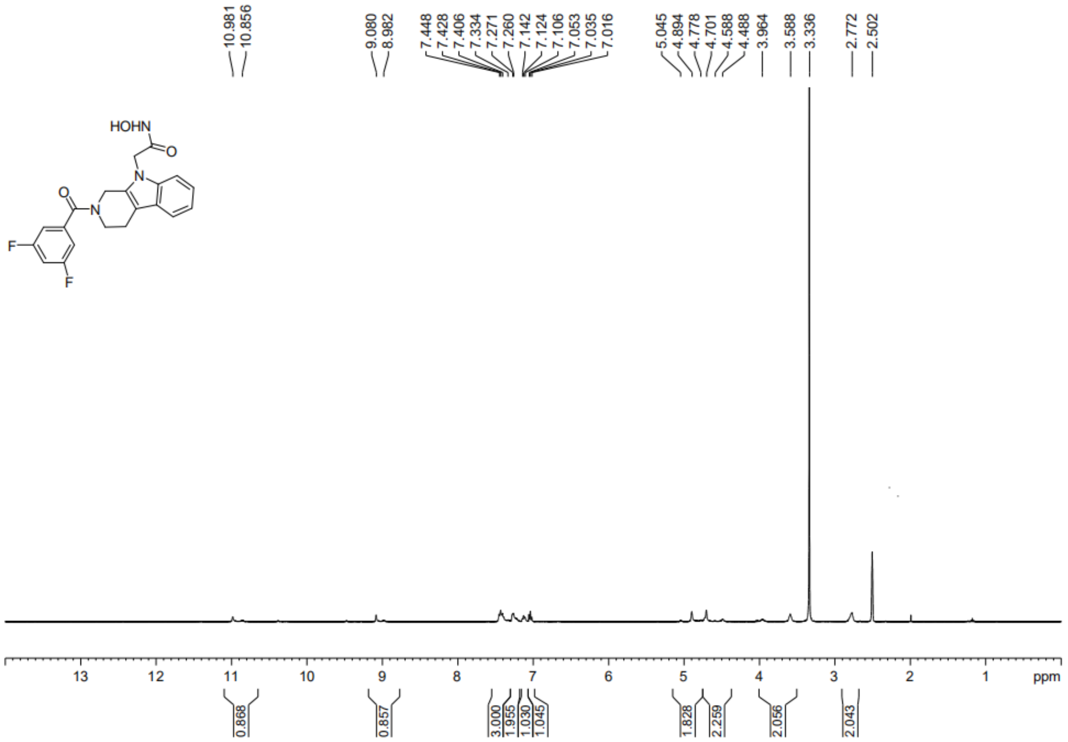

^{1}H NMR spectrum of **D24**

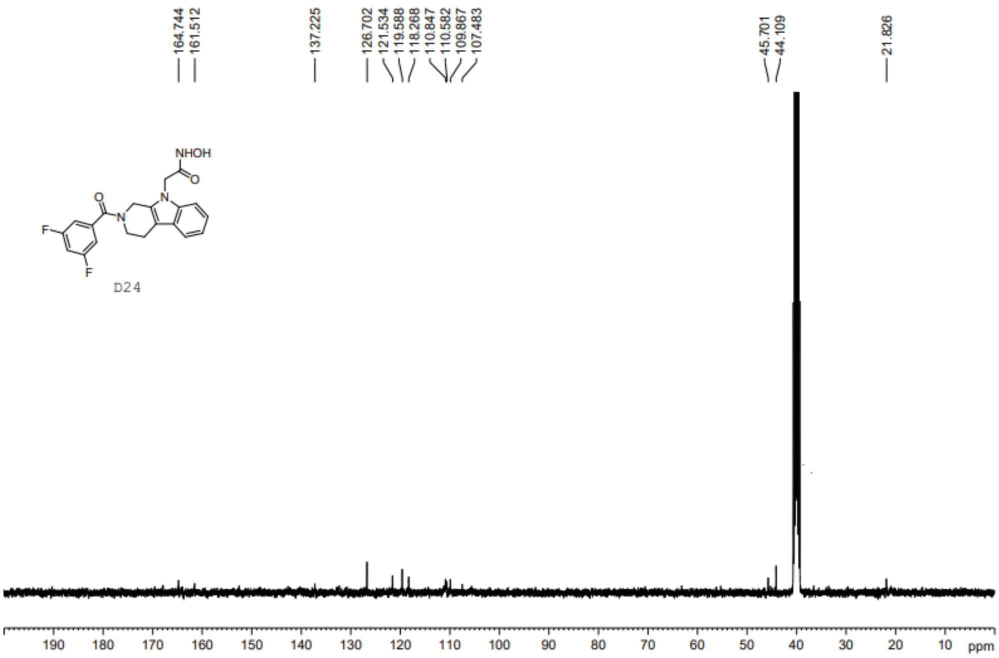

^{13}C NMR spectrum of **D24**

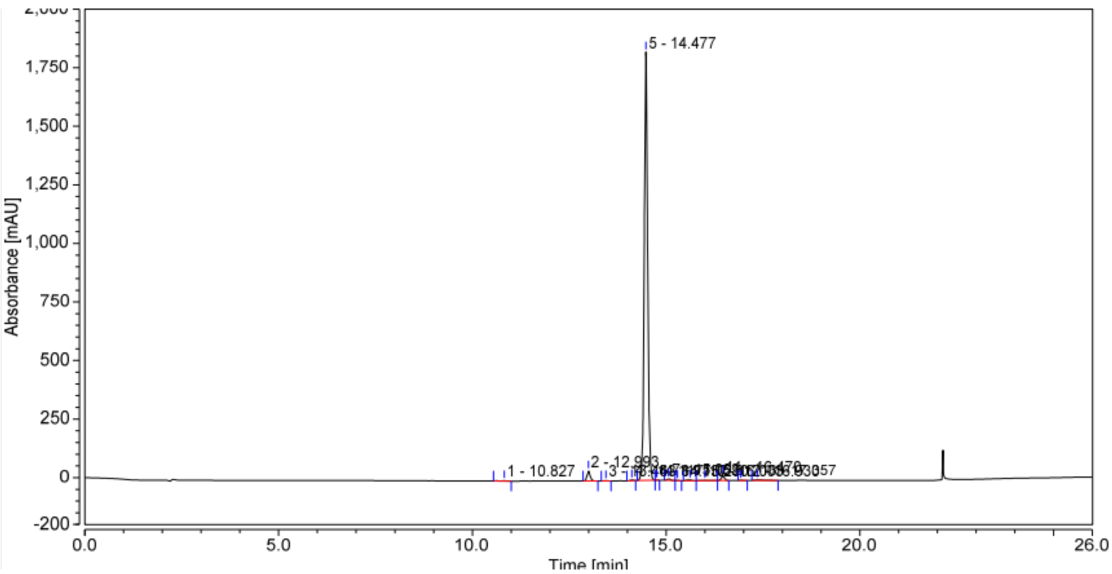

HPLC spectrum of **D24**

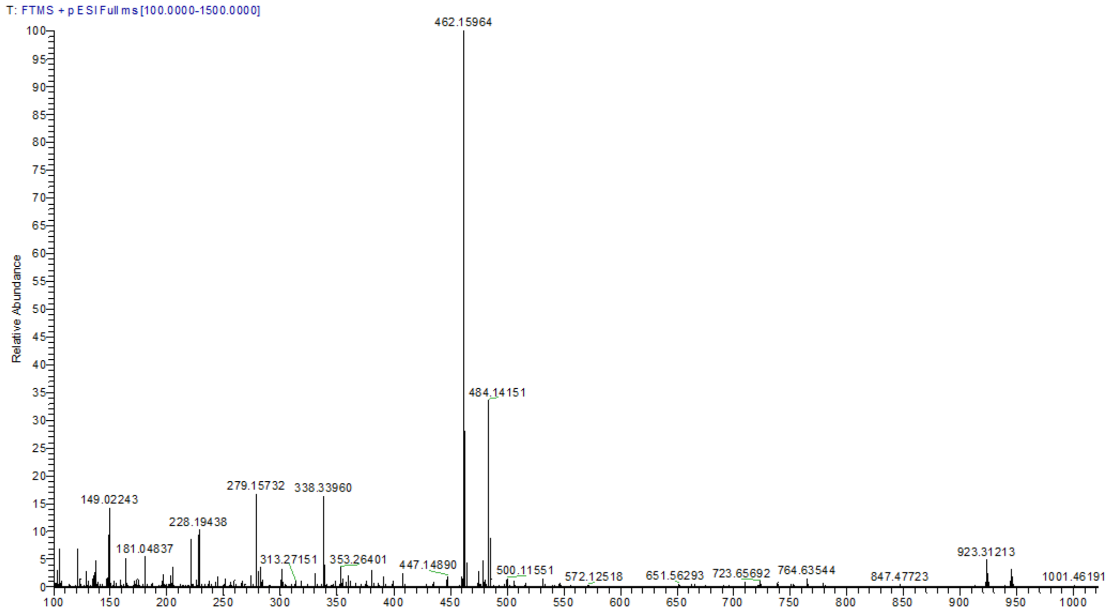

HRMS spectrum of **D25**

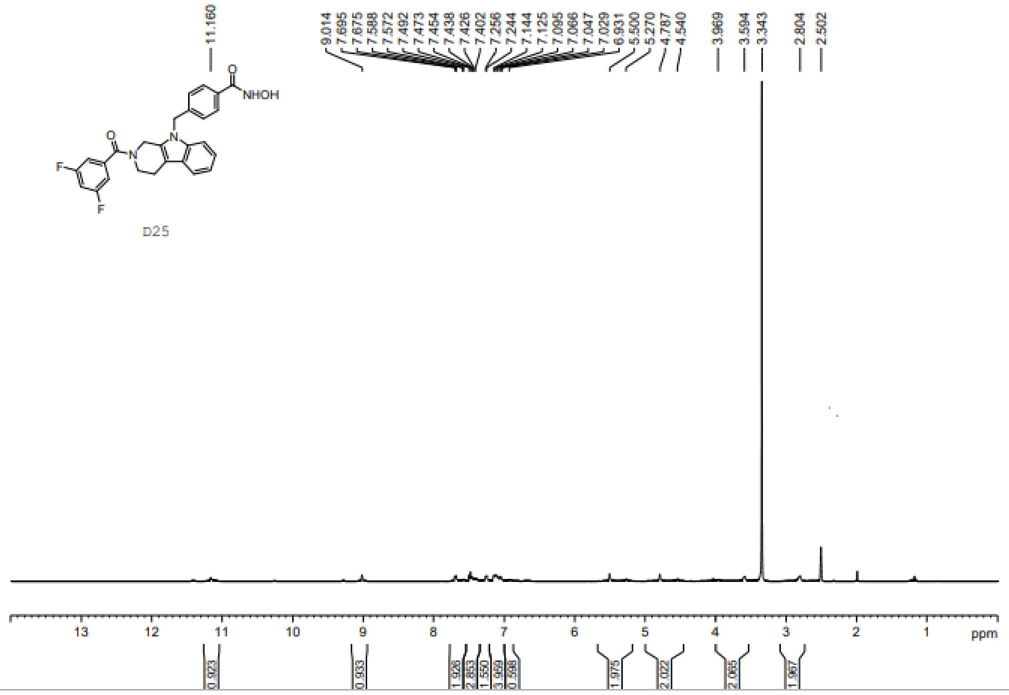

^{1}H NMR spectrum of **D25**

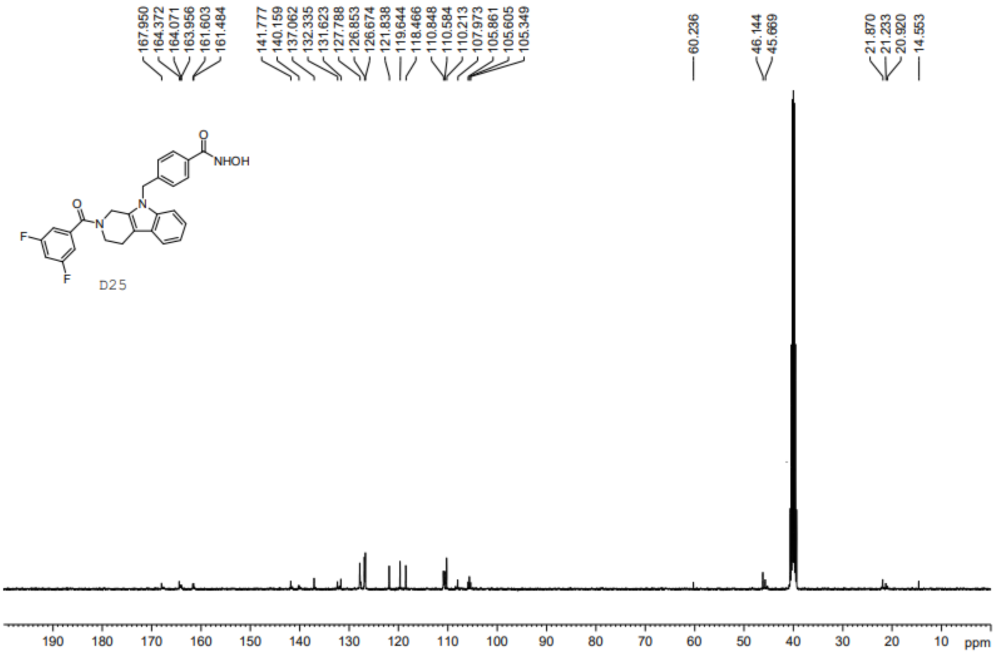

^{13}C NMR spectrum of **D25**

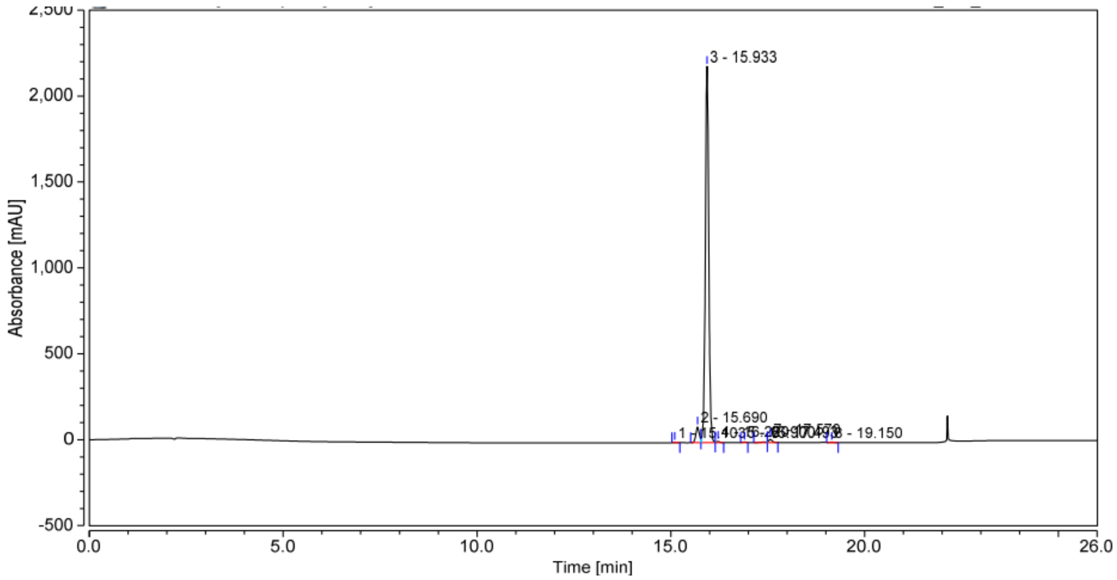

HPLC spectrum of **D25**

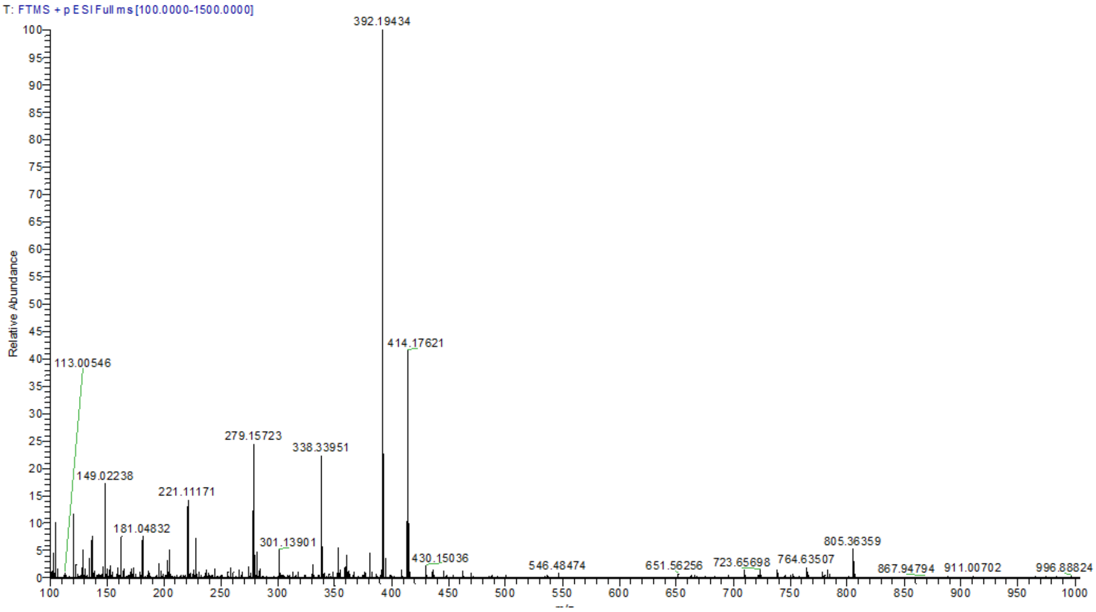

HRMS spectrum of **D26**

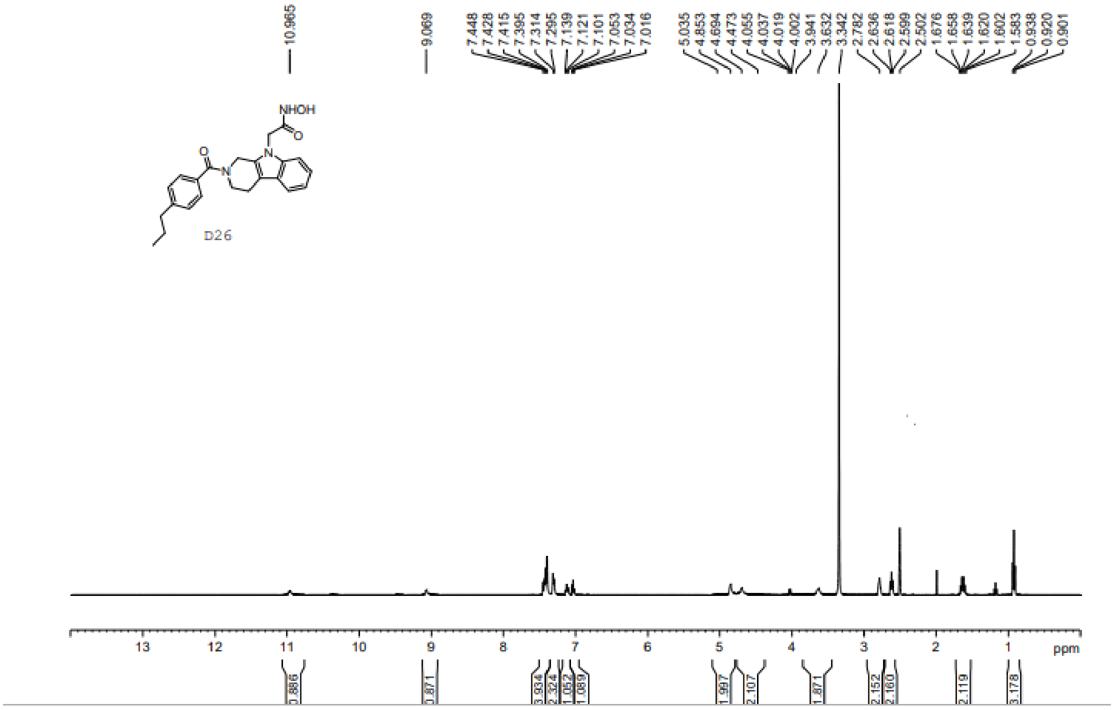

^{1}H NMR spectrum of **D26**

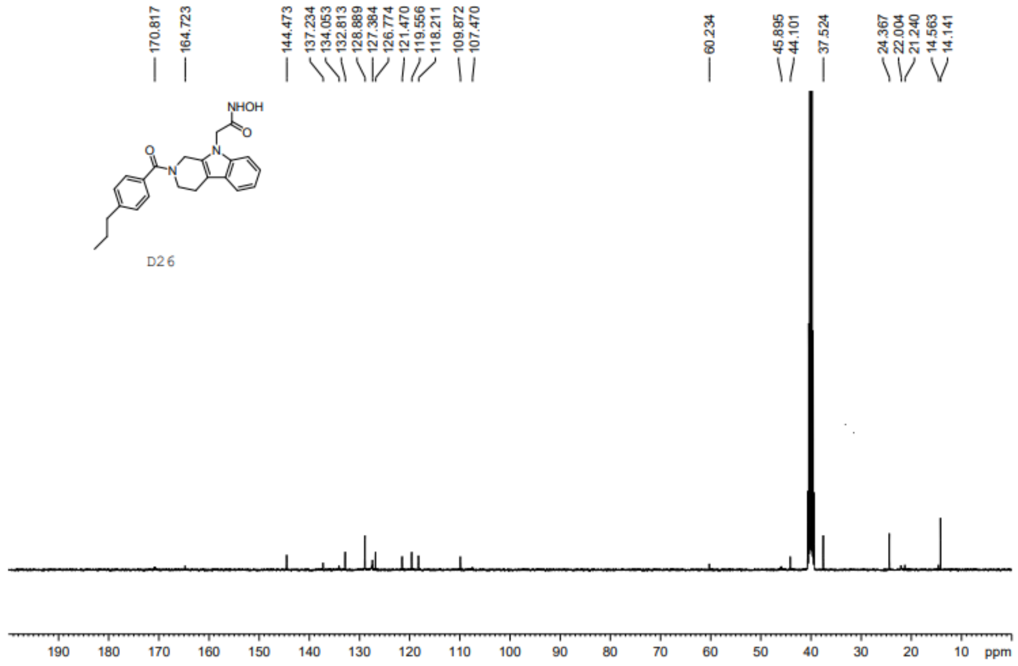

^{13}C NMR spectrum of **D26**

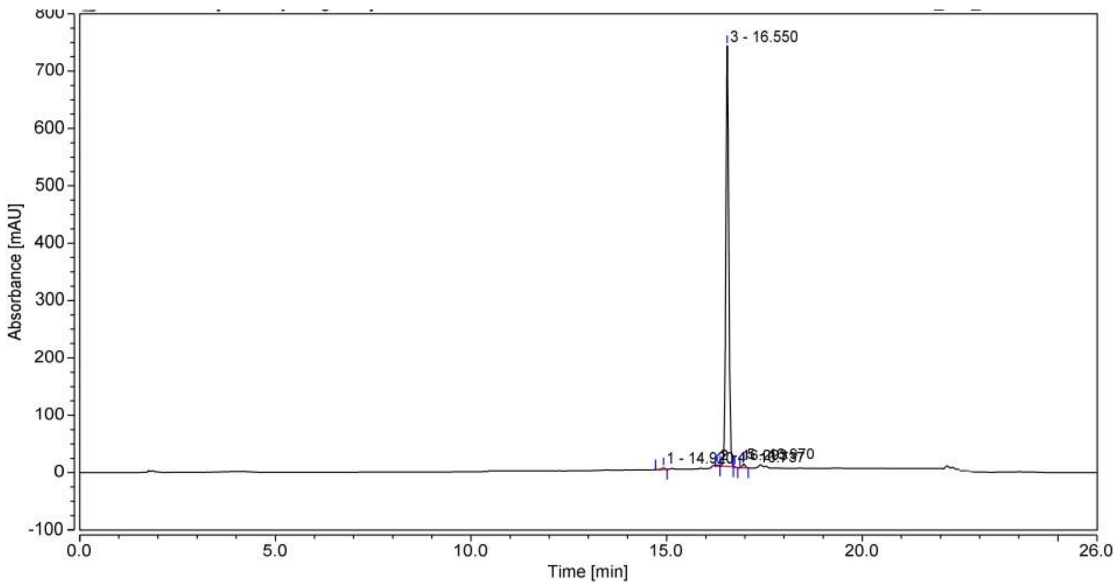

HPLC spectrum of **D26**

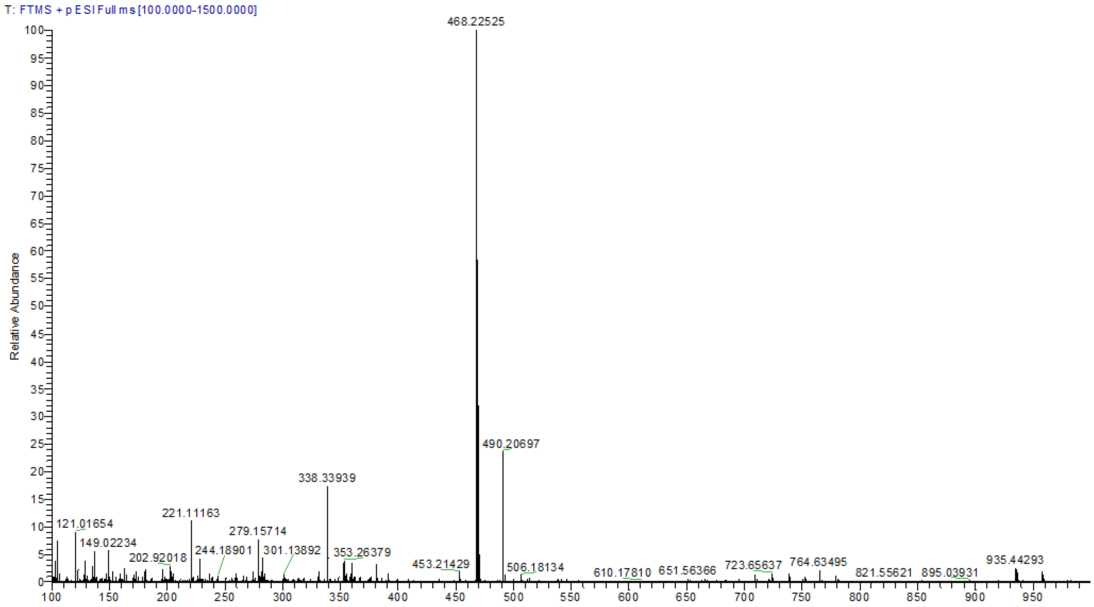

HRMS spectrum of **D27**

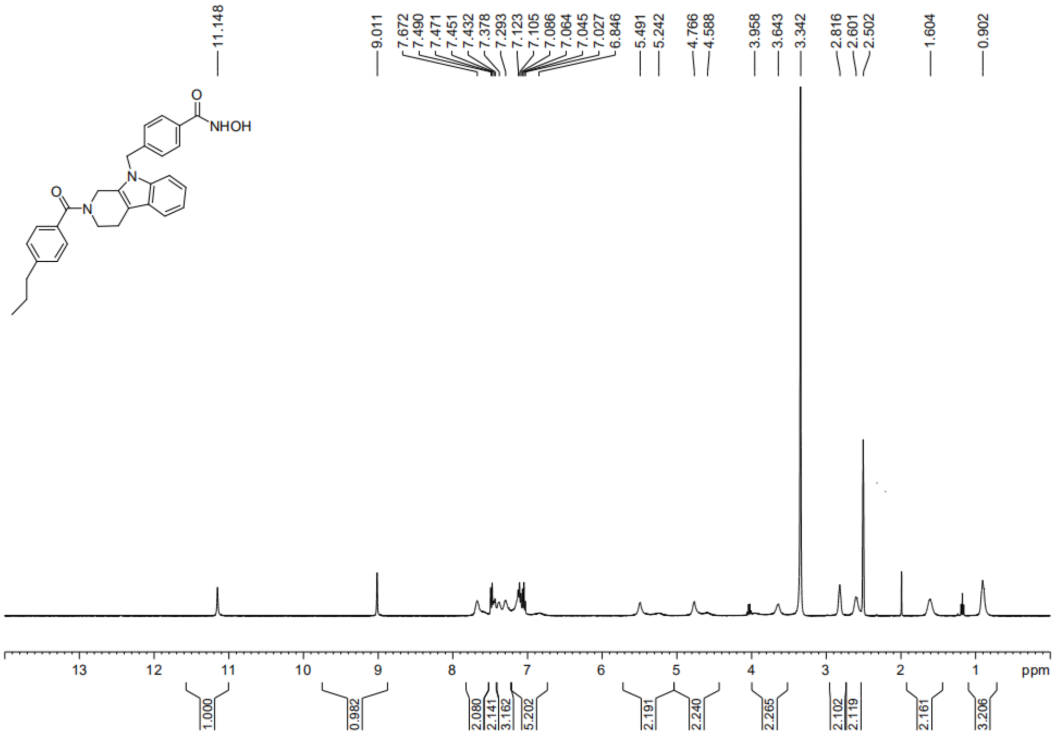

^{1}H NMR spectrum of **D27**

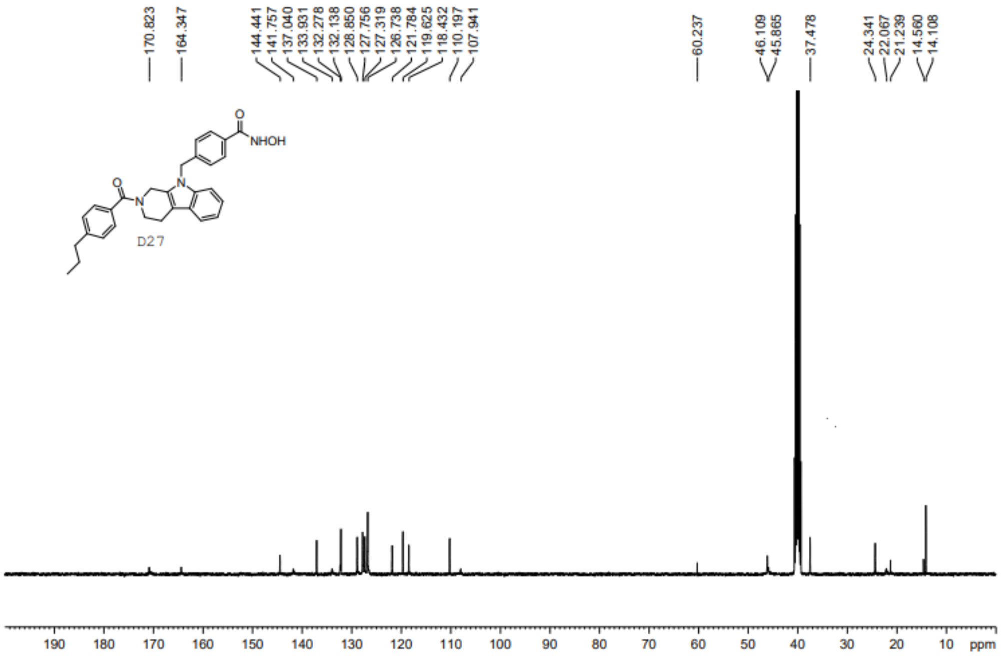

^{13}C NMR spectrum of **D27**

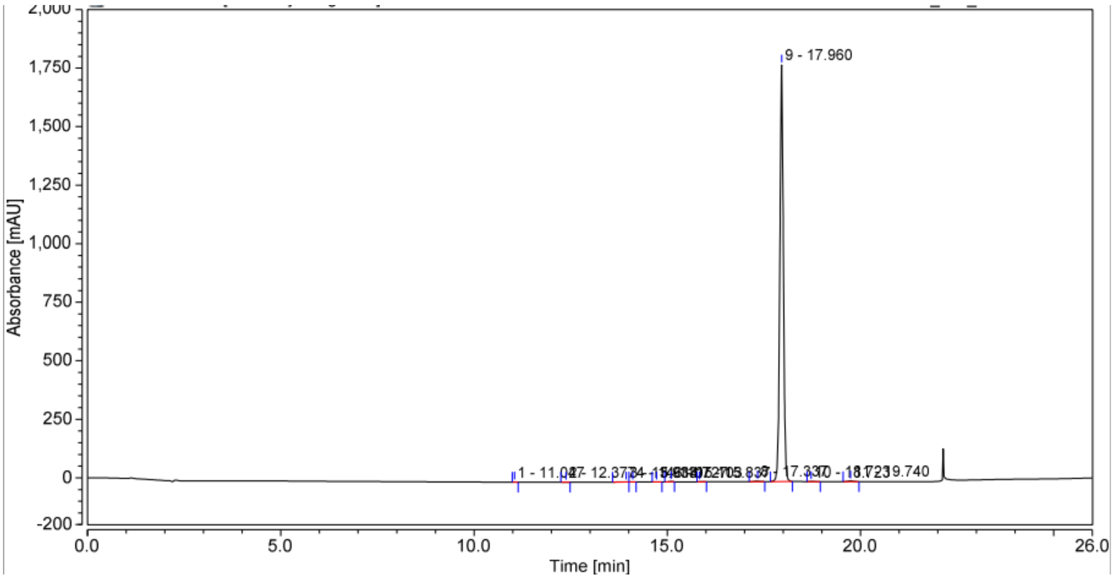

HPLC spectrum of **D27**

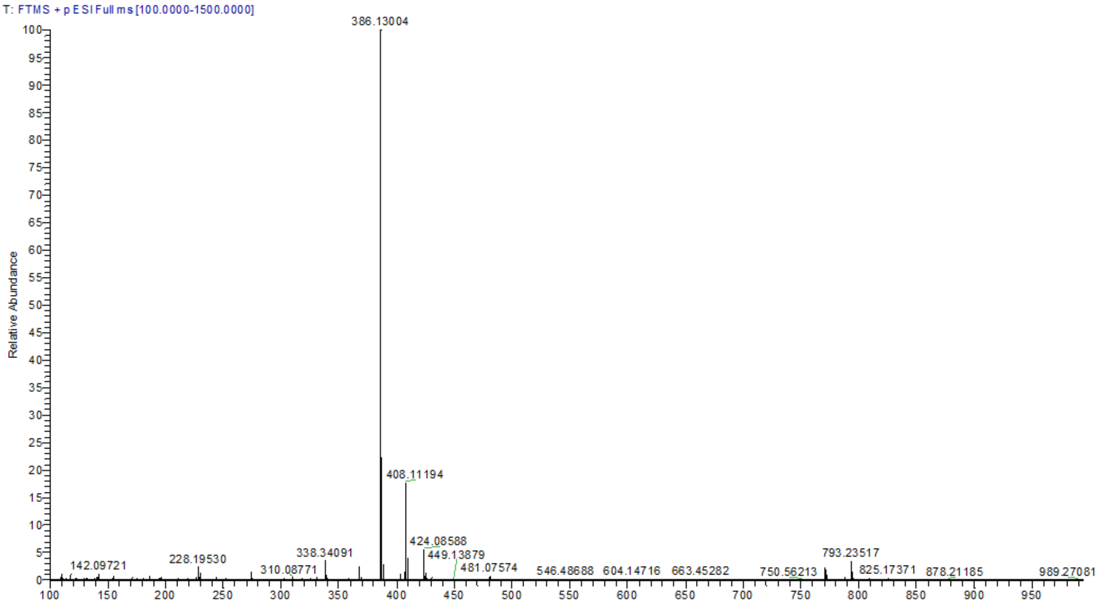

HRMS spectrum of **D28**

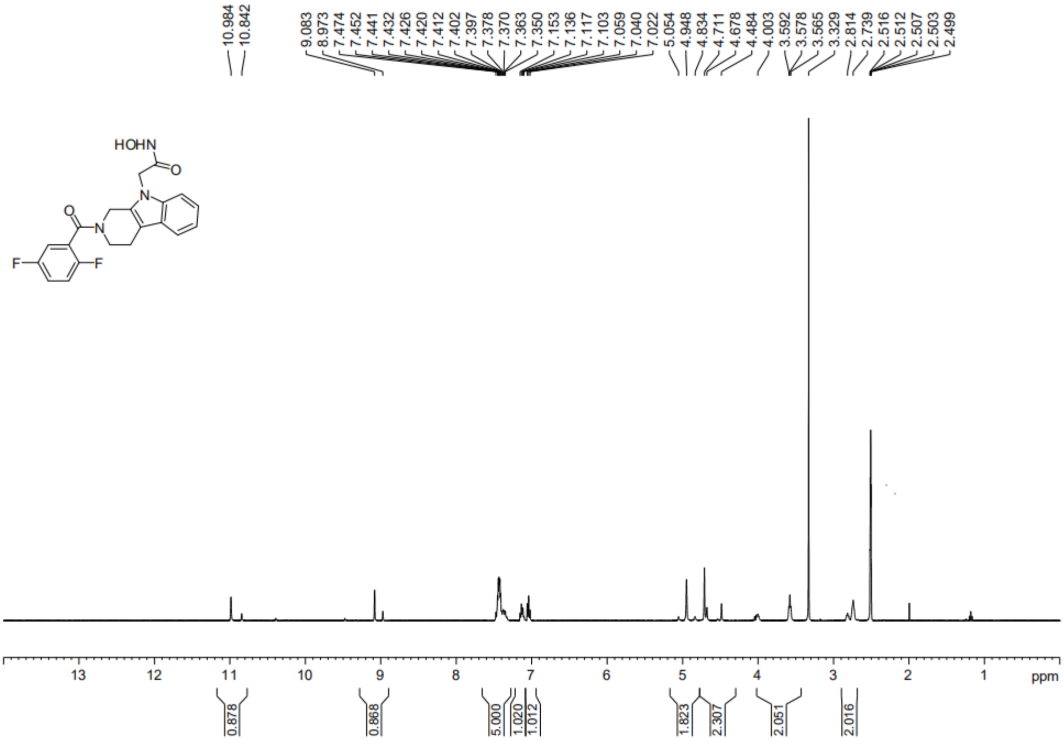

^{1}H NMR spectrum of **D28**

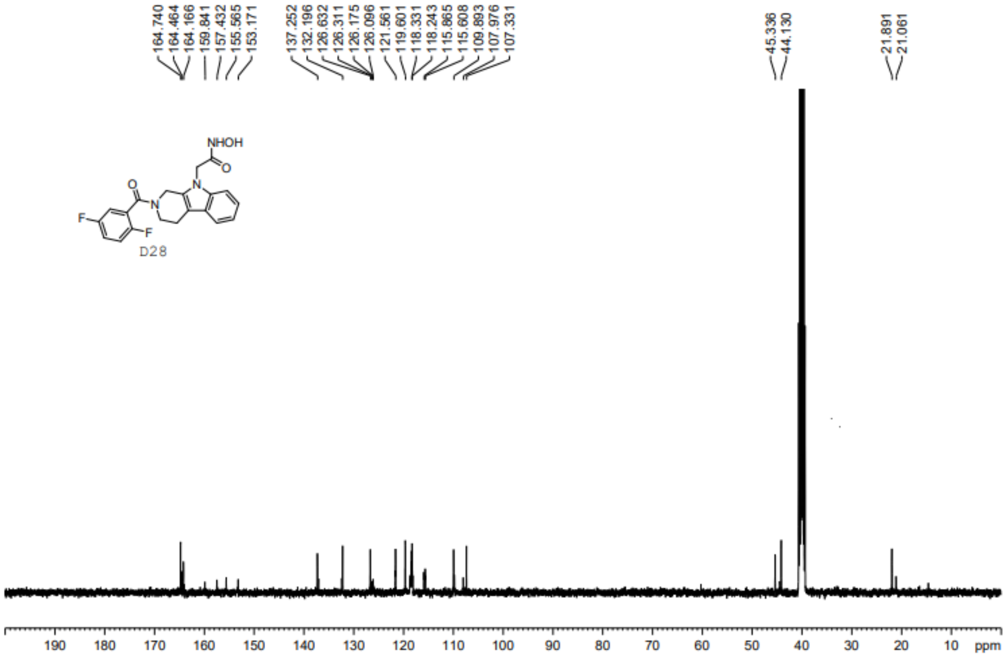

^{13}C NMR spectrum of **D28**

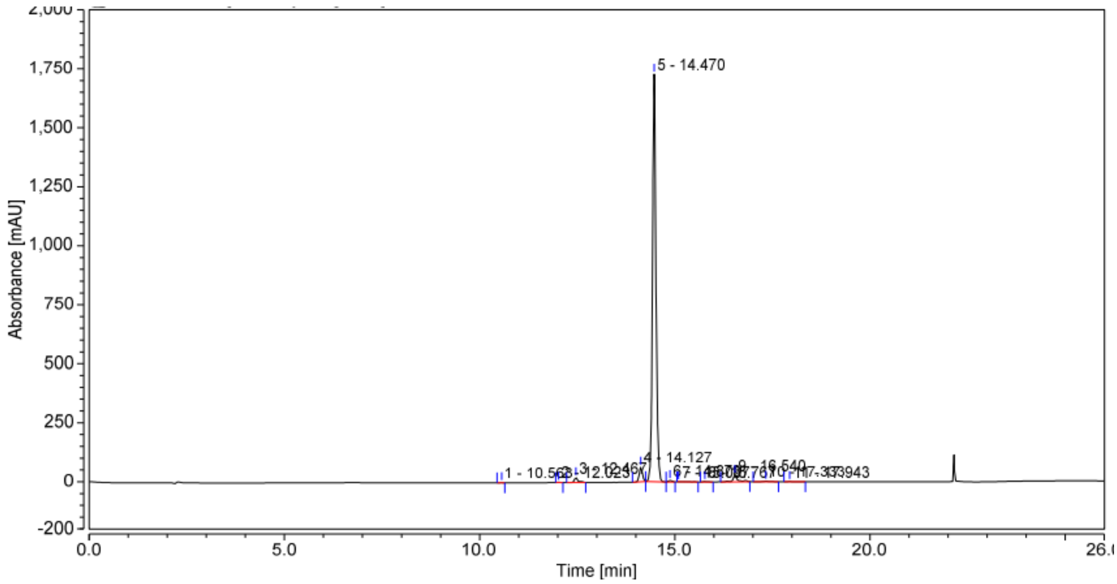

HPLC spectrum of **D28**

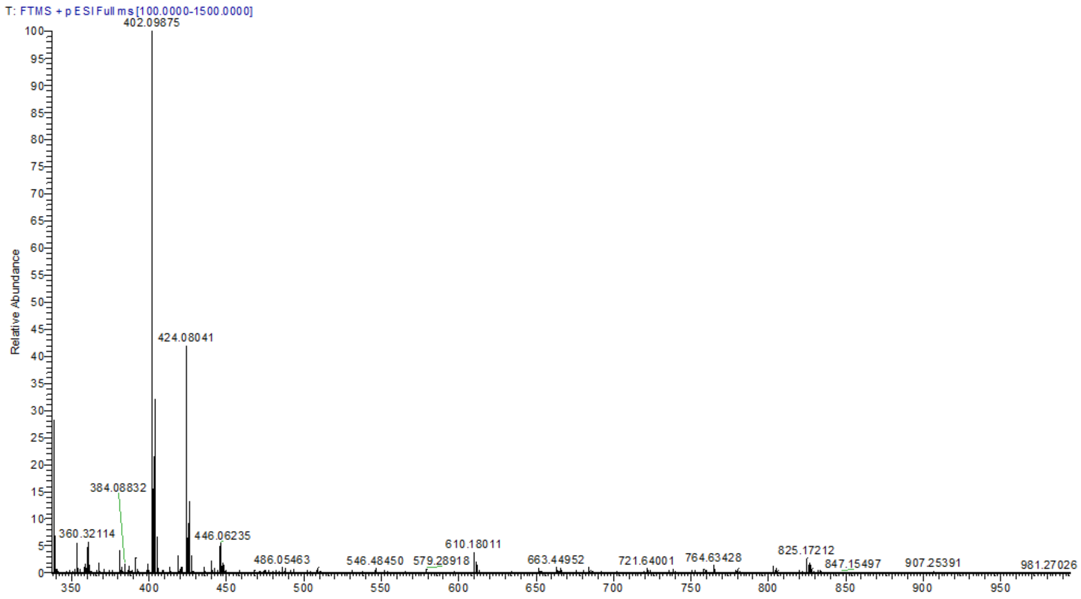

HRMS spectrum of **D29**

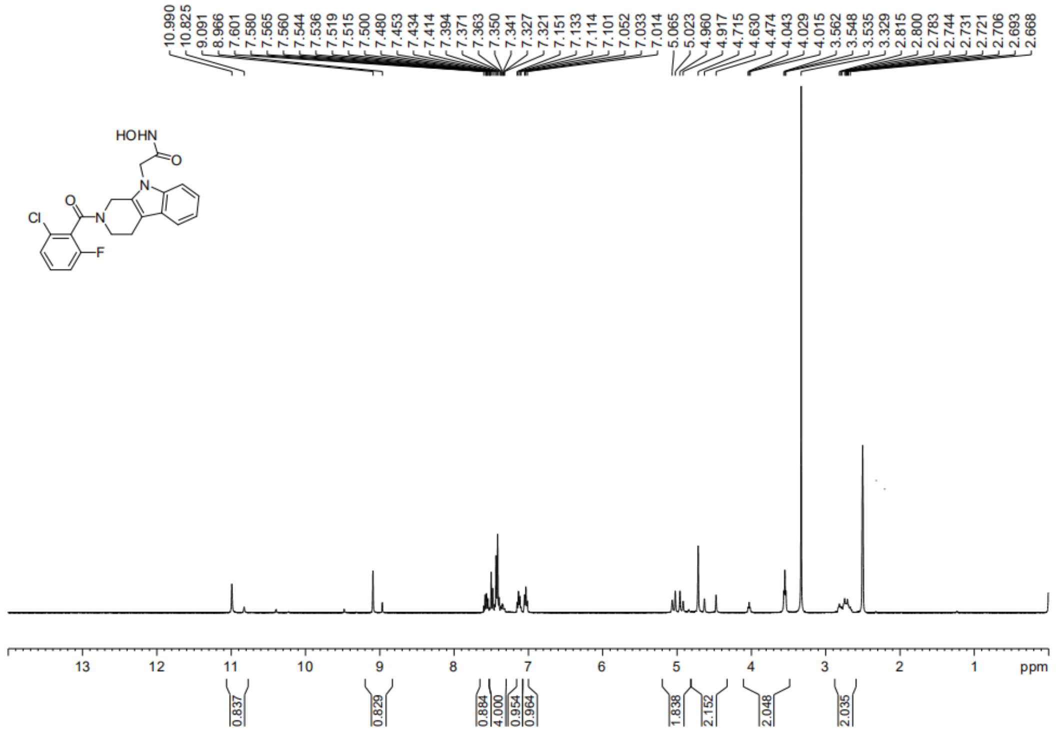

^{1}H NMR spectrum of **D29**

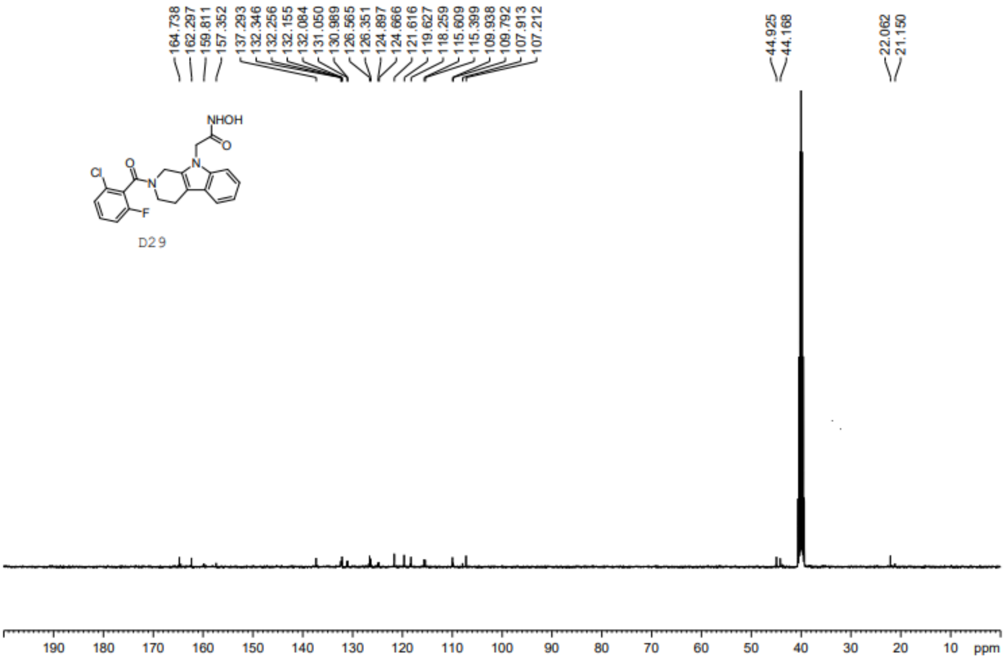

^{13}C NMR spectrum of **D29**

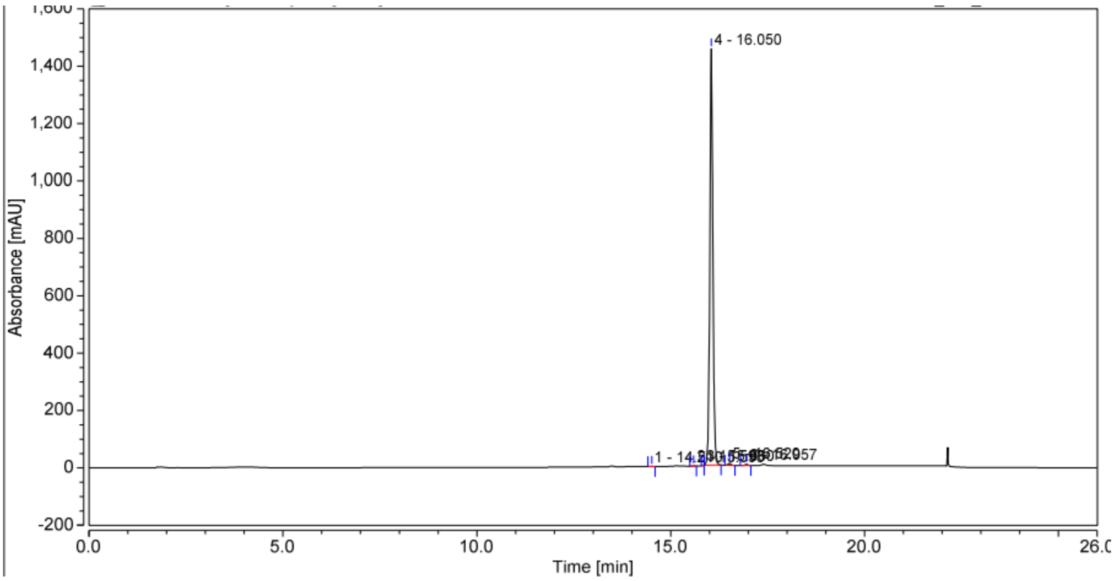

HPLC spectrum of **D29**
